# Access to benzo-fused nine-membered heterocyclic alkenes with a trifluoromethyl carbinol moiety *via* a double decarboxylative formal ring-expansion process under palladium catalysis†
†Electronic supplementary information (ESI) available: CCDC 1575063, 1575065, 1575062, 1589030. For ESI and crystallographic data in CIF or other electronic format see DOI: 10.1039/c7sc05447e


**DOI:** 10.1039/c7sc05447e

**Published:** 2018-02-23

**Authors:** Pulakesh Das, Satoshi Gondo, Punna Nagender, Hiroto Uno, Etsuko Tokunaga, Norio Shibata

**Affiliations:** a Department of Nanopharmaceutical Sciences , Department of Life Science and Applied Chemistry , Nagoya Institute of Technology , Gokiso, Showa-ku , Nagoya 466-8555 , Japan . Email: nozshiba@nitech.ac.jp; b Institute of Advanced Fluorine-Containing Materials , Zhejiang Normal University , 688 Yingbin Avenue , 321004 Jinhua , China

## Abstract

Direct access to pharmaceutically attractive benzo-fused nine-membered heterocyclic alkenes **3** with a trifluoromethyl carbinol moiety was achieved *via* a palladium-catalyzed double-decarboxylative formal ring-expansion process from six-membered trifluoromethyl benzo[*d*][1,3]oxazinones **1** to nine-membered trifluoromethyl benzo[*c*][1,5]oxazonines **3** in the presence of vinylethylene carbonates **2**. Generation of a Pd-π-allyl zwitterionic intermediate was proposed in the catalytic cycle. The trifluoromethyl group in the benzoxazinanones **1** plays an important role throughout the transformation. Diastereoselective chemical transformations of products **3** were also demonstrated.

## Abstract 2

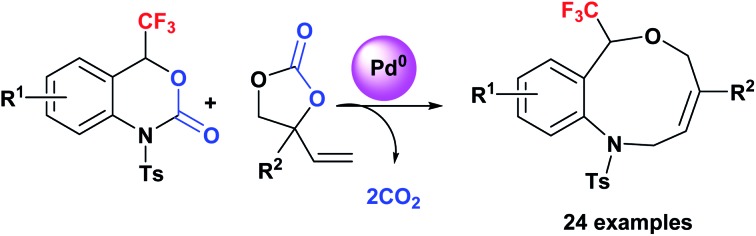
Direct access to benzo-fused nine-membered heterocyclic alkenes with a trifluoromethyl carbinol moiety was achieved *via* a palladium-catalysis.

## Introduction

**Fig. 1:**
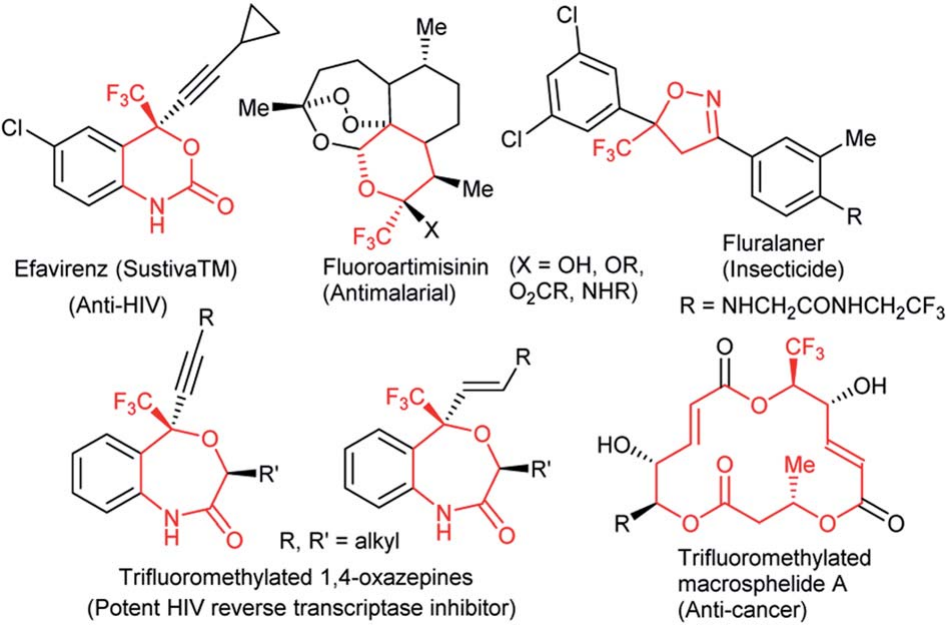
Biologically active heterocycles containing a trifluoromethyl carbinol moiety.

Fluoro-functionalized heterocycles with diverse ring sizes and ring systems have been well studied in pharmaceuticals and agrochemicals.^1^ Thus, a remarkable number of publications have been dedicated to the development of efficient synthetic methods to construct fluoro-functionalized heterocycles.^1^,^2^ In particular, heterocyclic molecules with a trifluoromethyl carbinol moiety, *i.e.*, CF_3_C(OR^1^)R^2^R^3^, have gathered much attention^3^–^6^ on account of their promising biological properties. Efavirenz^4^ (anti-HIV), trifluoromethylated artemisinins^5^ (anti-malarial), and fluralaner^6^ (insecticide and acaricide) are representative examples (Fig. 1).

**Scheme 1:**
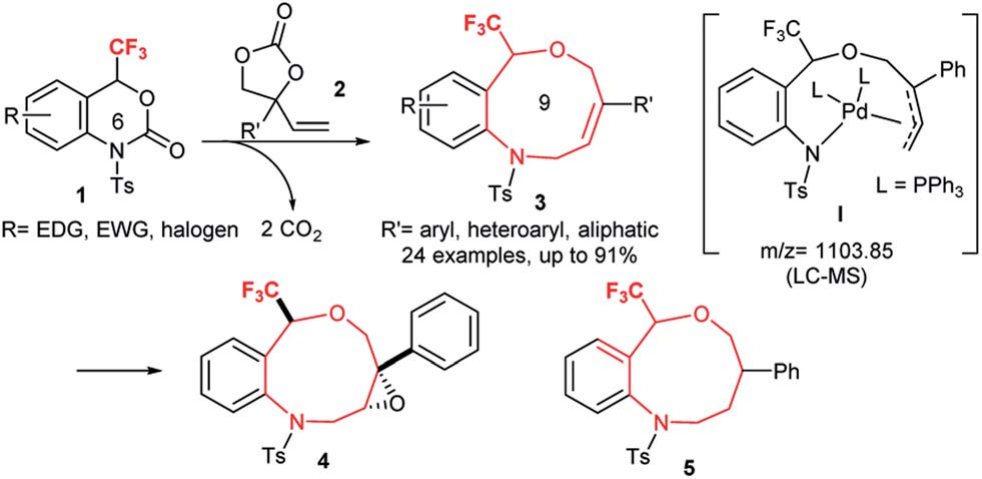
Direct access to benzo-fused nine-membered heterocyclic alkenes **3** with a trifluoromethyl carbinol moiety from six-membered oxazinones **1** and vinylethylene carbonates **2***via* palladium-catalyzed double decarboxylative cycloaddition and the further diastereoselective chemical transformations of **3**.

In this context, our group has been engaged in the development of novel synthetic methodologies for fluorine-containing heterocycles for decades.^7^ Including our reports,^7^ the present synthetic strategies for fluorinated heterocyclic molecules are mostly limited to the construction of five- and six-membered ring systems,^1^,^2^,^7^ while the synthesis of medium- to large-sized fluoro-functionalized heterocycles such as derivatives of benzo-oxazepine^8^ and macrosphelide A^9^ (Fig. 1) is extremely rare, despite the pharmaceutical importance of medium-sized heterocyclic compounds (non-fluorinated)^10^ and biologically active natural products.^11^ Very recently, Liu and co-workers reported an elegant method for the construction of fluoroalkyl-functionalized medium-/large-sized carbocyclic alkenes *via* an intramolecular radical trifluoromethylation–cyclization process.^12^ Recently, Zhao and co-workers successfully reported the palladium-catalyzed [5 + 4] and [6 + 4] cycloaddition reactions of azadienes with vinylethylene carbonates and vinyl oxetanes respectively in good yields and selectivities.^13^ We disclose herein the first synthesis of benzo-fused nine-membered heterocyclic alkenes **3** with a trifluoromethyl carbinol moiety and vinylethylene carbonates **2**^14^ (Scheme 1).

The resulting trifluoromethylated heterocycles **3** have a benzo[*c*][1,5]oxazonine skeleton, and are not only medicinally attractive fluorine-containing heterocycles,^1^ but also expanded variants of well-known [1,4]oxazepine pharmaceuticals.^15^ Synthesis of the titled nine-membered compounds **3** were achieved from previously unknown trifluoromethylated benzoxazinanones **1** (six-membered ring) *via* a formal ring-expansion pathway under palladium catalysis. The reaction proceeded *via* the double decarboxylation (DDC)^16^ of **1** and vinylethylene carbonates **2** followed by a [5 + 4] cycloaddition reaction. The formation of Pd-complex **I** as an intermediate was proposed by LC-MS spectrometric analysis. This method provides an expedient access to trifluoromethylated benzo[*c*][1,5]oxazonines **3** with diverse functional groups in the aromatic moiety, including electron-donating, electron-deficient, and halogenic groups. Moreover, the alkene moiety in products **3** was further functionalized by conventional chemical transformations such as epoxidation to **4** and reduction to **5** (Scheme 1) with high diastereoselectivities which make this novel trifluoromethylated nine-membered skeleton more attractive as a template for drug discovery research. The presence of a trifluoromethyl group on **1** plays a pivotal role for their successful transformation to **3** based on comparative studies using non-CF_3_-varients of **1**.

## Results and discussion

We started a preliminary investigation with the reaction of trifluoromethyl (CF_3_) benzoxazinanone **1a** and phenyl vinylethylene carbonate **2a** in the presence of suitable palladium precursors and/or phosphine ligands (Table 1). We first attempted our reaction of **1a** using similar Pd_2_(dba)_3_·CHCl_3_ conditions in the presence or absence of phosphine ligands, but the results were disappointing (entries 1–4). Moving on to Pd(PPh_3_)_4_ as a palladium precursor at 50 °C in THF furnished exclusively a nine-membered ring in good yield of 70% (entry 5). Motivated by this result, further optimization was carried out in different solvents. In toluene, a slight decrease in yield was observed, at 66% (entry 6), while in dichloroethane yield improved to 79% (entry 7). Lowering the temperature to room temperature (rt) furnished good yield (70%), but 40 hours were required to complete the reaction (entry 8). An excellent yield of 91% (89%) was observed by increasing the temperature to 80 °C (entry 9). Increasing the temperature further decreased yield dramatically (entry 10, see ESI for more details†).

**Table 1:** Optimization conditions^*a*^

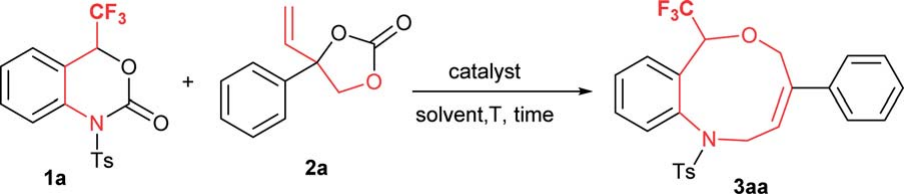					
Entry	Pd catalyst (with or without ligand)	Solvent	*T* [°C]	*t* [h]	Yield^*c*^ [%]
1^*b*^	10 mol% Pd_2_(dba)_3_·CHCl_3_	DCM	rt	24	—
2^*b*^	5 mol% Pd_2_(dba)_3_·CHCl_3_	THF	40	24	—
3	5 mol% Pd_2_(dba)_3_·CHCl_3_/10 mol% PCy_3_	THF	40	24	—
4	5 mol% Pd_2_(dba)_3_·CHCl_3_/10 mol% d*t*bpmb	THF	40	24	—
5	5 mol% Pd(PPh_3_)_4_	THF	50	7	75(70)
6	5 mol% Pd(PPh_3_)_4_	Toluene	50	36	68(66)
7	5 mol% Pd(PPh_3_)_4_	DCE	50	12	83(79)
8	5 mol% Pd(PPh_3_)_4_	DCE	rt	40	79(70)
9	5 mol% Pd(PPh_3_)_4_	DCE	80	12	91(89)
10	5 mol% Pd(PPh_3_)_4_	DCE	Reflux	12	40(34)

^*a*^Experiments were performed with **1a** (0.1 mmol), **2a** (0.15 mmol), 5 mol% Pd(PPh_3_)_4_ (0.05 mmol) in 1.0 mL solvent.

^*b*^ **2a** (0.12 mmol) was used.

^*c*^Yields are ^19^F NMR yields with internal standard PhCF_3_ and yields (isolated) are also given in parentheses. d*t*bpmb = 1,2-bis(di-*tert*-butylphosphinomethyl)benzene. DCE = 1,2-dichloroethane.

Based on the optimized reaction conditions, the flexibility of the DDC reaction was scrutinized by using a broad array of vinylethylene carbonates (VECs) **2a–m** with **1a**. The results are summarized in Table 2. Both electron-withdrawing and electron-donating groups on the phenyl ring of **2** furnished good to excellent yields. VECs **2b–c**, which have electron-donating groups (Me and OMe) at the *p*-position, reacted efficiently to afford the desired products **3** in excellent yields (**3ab**: 83%; **3ac**: 78%) whereas VEC **2g**, which contains an electron-withdrawing group (CF_3_) at the *p*-position, furnished moderate yield (**3ag**: 56%). Furthermore, halogen-substituted VECs (**2d**: F; **2e**: Cl; **2f**: Br) also underwent the DDC reaction very smoothly to furnish good to excellent yields (**3ad**: 69%; **3ae**: 86%; **3af**: 91%). Similarly, a highly electronegative atom (**2h**: F) and an electron-donating group (**2i**: OMe) at the *o*-position afforded excellent yields (**3ah**: 84% and **3ai**: 88%). Noticeably, substrates bearing an electron-withdrawing group (F) and an electron-donating group (OMe) at the *o*-position furnished higher yields than *p*-substituted substrates. Moreover, the scope of VECs **2** was extended to heteroaryl systems (**2j**: 2-furyl; **2k**: 2-thiophenyl) and the reaction proceeded smoothly to afford the desired products **3** in good yields (**3aj**: 76%; **3ak**: 79%). Gratifyingly, non-aromatic substituent VEC **2l** and extended π conjugate naphthalene-derived VEC **2m** also underwent the cycloaddition reaction to furnish **3al** and **3am** in moderate to good yield (53% and 65%, respectively), thus significantly broadening the scope of substrate **2** of this DDC system (Table 2).

**Table 2:** Scope of vinylethylene carbonates (VECs) **2**^*a*^

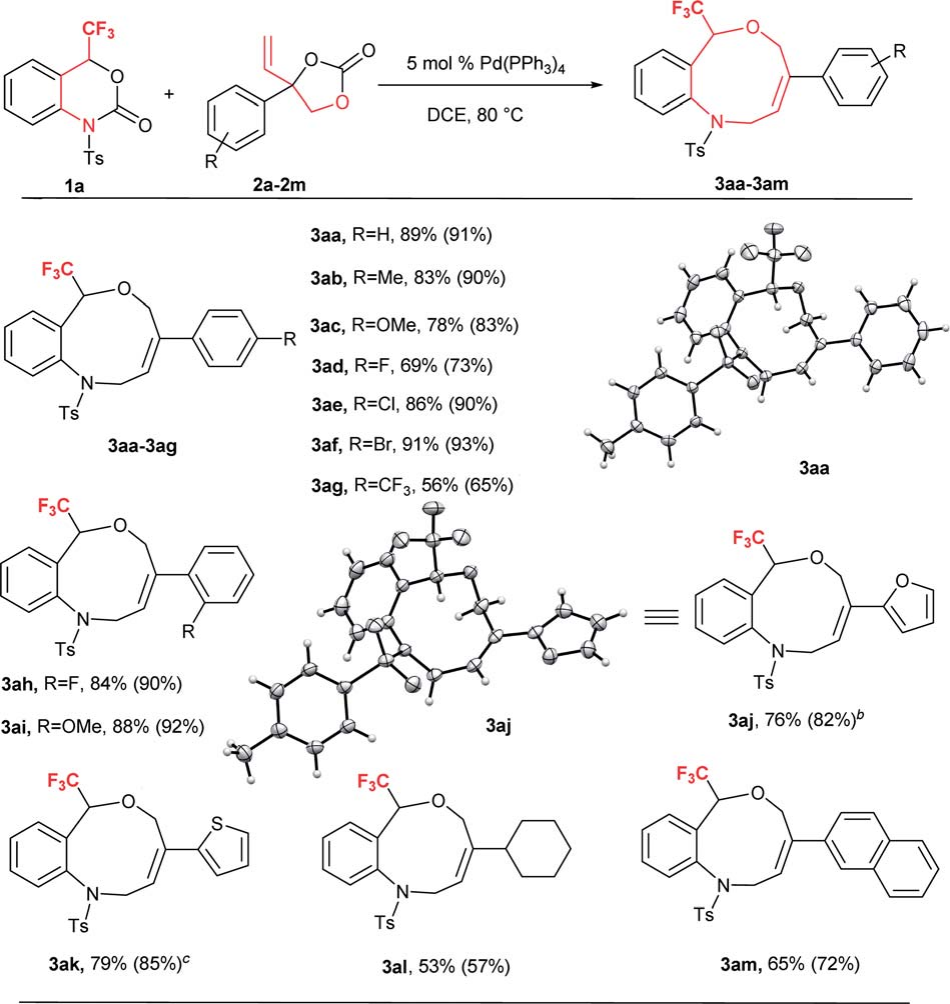

^*a*^Experiments were performed with **1a** (0.1 mmol), **2a–m** (0.15 mmol), Pd(PPh_3_)_4_ (0.05 mmol) in 1.0 mL dry DCE with stirring at 80 °C for 12–16 h. Yields are isolated yields and ^19^F NMR yields with internal standard PhCF_3_ also shown in parentheses. **3aa**: CCDC 1575063; **3aj**: CCDC 1575065.

^*b*^0.20 mmol of **2j** was used.

^*c*^0.20 mmol of **2k** was used.

Spurred by this interesting result, a range of differently substituted CF_3_-benzoxazinanones **1b–e** were further examined to better understand the DDC reaction (Table 3). Substituents on **1** with electronically dissimilar properties at different positions on the benzene ring were well tolerated to provide **3** in moderate to good yields. The substrate-bearing electron-donating methyl group on the benzene ring, **1b** produced CF_3_-tetrahydrobenzoxazonine **3ba** in 81% yield. The halogen-substituted CF_3_-benzoxazinanones **1c** and **1e** (F and Br) produced DDC products **3** in moderate to good yields (**3ca**: 69% and **3ea**: 78%) (Table 3).

**Table 3:** Scope of benzoxazinanones **1**^*a*^

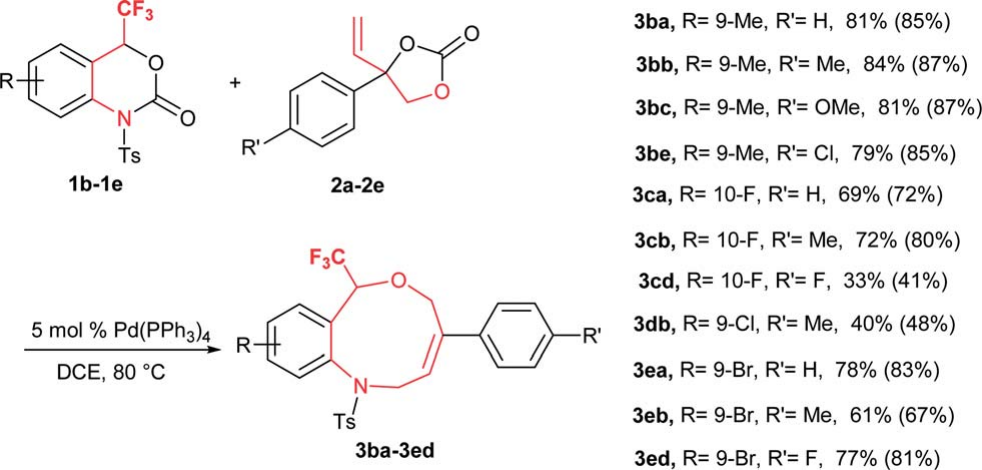

^*a*^Unless noted otherwise, the reaction was performed with 0.10 mmol of **1b–e** as mentioned in Table 1. Yields are isolated yields and ^19^F NMR yields with internal standard (PhCF_3_) also shown in parentheses.

**Scheme 2:**
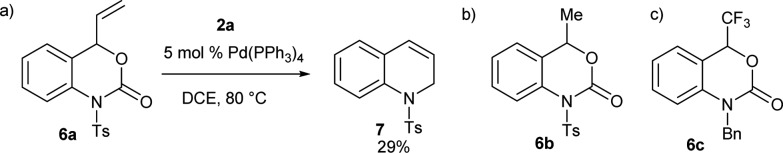
Reaction of benzoxazinanones **6a–c** which contain different substituents at the C-4 position and N-protected group, with **2a** under optimized conditions gave different results.

To ensure the effect of the CF_3_ group at the C-4 position, next we examined the reaction of benzoxazinanones **6**, which contain different substituents at the C-4 position, with **2a** (Scheme 2). In recent years, palladium-catalyzed cyclization reactions using vinyl benzoxazinanone **6a** with a variety of substrates have been actively investigated by several groups.^17^ We thus first attempted the reaction of **6a** with **2a**. Interestingly, substrate **6a** with a vinyl at the C-4 position produced a very different result. Under our best conditions, a vinyl-substituted benzoxazinanone **6a** was converted to an intramolecular cyclization product **7** in 29% yield but no desired nine-membered cyclized product was observed (Scheme 2a). We next examined the reaction using **6b** with a methyl group at the C-4 position instead, but were unable to furnish the desired product and the starting material **6b** remained (Scheme 2b). Similar no conversion was obtained when we carried out the reaction of **6c** having protected *N*-benzyl group (Scheme 2c). Although the reasons for the high reactivity of **1a** are not clear, it might be due to the higher electrophilicity value of **1a** induced by the strong electronegativity of the CF_3_ group (group electronegativity of CF_3_ is 3.45).^18^ To ensure the effect of the CF_3_ group at the C-4 position of **1a**, we performed a DFT calculation. The electrophilic value of **1a** having CF_3_ at the C-4 position was estimated to be 3.67 (*ω* (eV)) while that of **5b** containing CH_3_ at the C-4 position (3.33) was lower (Table S6, Fig. S1 in ESI for details†).

**Fig. 2:**
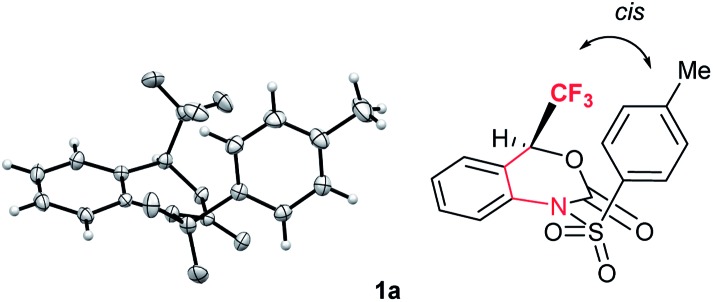
X-ray crystallographic analysis of **1a** (CCDC ; 1575062 †) revealed a sterically unfavourable *cis*-configuration between CF_3_ and tosyl groups.

Interestingly, the X-ray crystallographic analysis of starting substrate **1a** revealed that **1a** has a sterically unfavourable *cis*-configuration between CF_3_ and tosyl groups (Fig. 2). Although the reasons for the stabilization of **1a** in this configuration are not sure,^19^ the steric repulsion might be the additional factor for the high reactivity of **1a** for decarboxylation reaction.

**Scheme 3:**
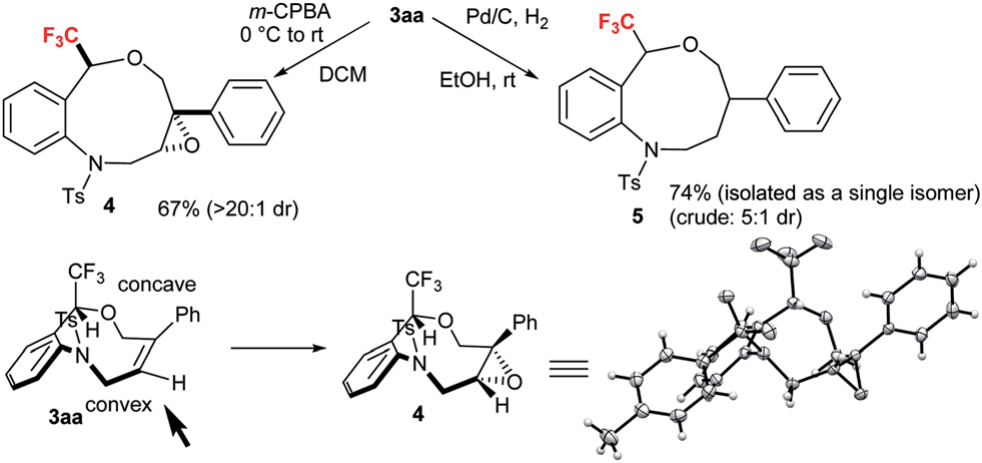
Diastereoselective derivatizations of tetrahydrobenzoxazonine **3aa**.

To demonstrate the synthetic applicability of CF_3_-substituted tetrahydrobenzoxazonines **3**, epoxidation and hydrogenation reactions were carried out as displayed in Scheme 3 based on the classical work of Still and Hoveyda.^20^ By using the Zhao's condition13*a* we performed the epoxidation of **3aa** in the presence of *m*-CPBA at 0 °C to rt successfully transformed to epoxide **4** with 67% yield and >20 : 1 diastereoselectivity through the peripheral attack. The X-ray crystallographic structure of **4** (CCDC ; 1589030 †) suggested that epoxidation proceeded *via* a less hindered convex approach. Hydrogenation of **3aa** with H_2_ in the presence of Pd–C at rt furnished the desired product **5** (5 : 1 dr) in 74% yield (isolated as a single isomer) (Scheme 3).

**Scheme 4:**
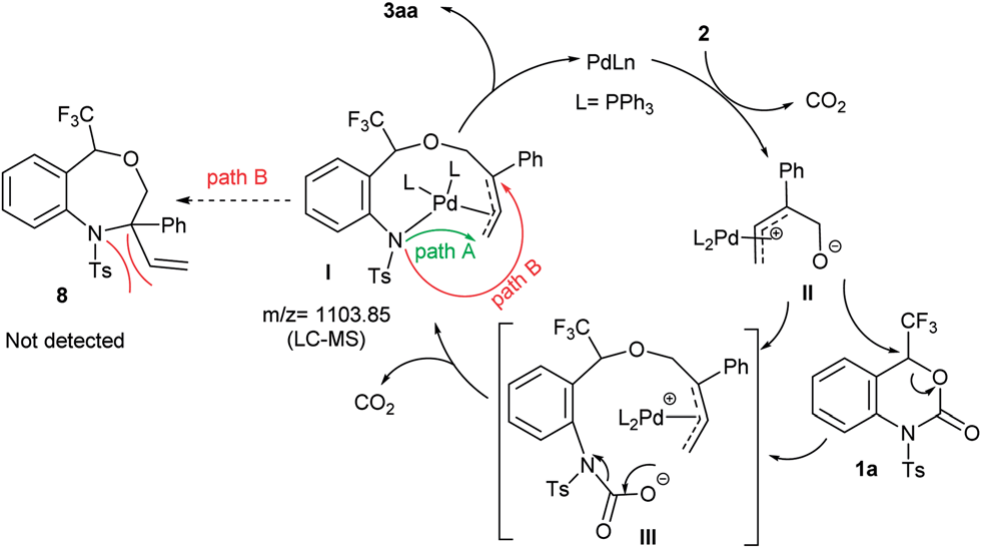
Plausible mechanism.

A plausible reaction mechanism of the palladium-catalyzed DDC reaction of **1a** with **2a** to **3aa** is portrayed in Scheme 4. The catalytic cycle is first initiated by the oxidative addition of Pd(0) with **2** followed by decarboxylation, which generates the π-allyl-Pd(ii) complex **II**. The extremely nucleophilic nature of the alkoxide oxygen of **II** attacks the most electrophilic carbon atom attached to the CF_3_ group of **1a** which triggers the opening of benzoxazinanone ring to generate reactive species **III**. Due to its highly reactive nature, species **III** immediately transforms into Pd-complex **I***via* decarboxylation. Recently, Kleij *et al.* disclosed the similar kind of six membered Pd-complex with the support of DFT calculations.^21^ In our case, the formation of Pd-complex **I** was confirmed by LC-MS spectrometry (Fig. S2 in ESI for detail†) but we could not detect it by NMR (Fig. S3, in ESI for detail†).

From complex **I**, there might be two possible pathways for the formation of two different cyclized products. Attack at the terminal position of the Pd-complex (path A) would generate the [5 + 4] cycloaddition product **3aa** while internal attack (*i.e.*, path B) of Pd-complex could result in [4 + 3] cycloaddition to furnish a seven-membered heterocycle **8**. However, we did not obtain the [4 + 3] cycloaddition adduct **8**. This may be attributed to steric hindrance of **8**, *i.e.*, the NTs group as well as the tetrasubstituted tertiary carbon center on **8**.

## Conclusions

In conclusion, we have established a novel and highly efficient methodology for the synthesis of benzo-fused nine-membered heterocyclic alkenes with a trifluoromethyl carbinol moiety through a palladium-catalyzed double decarboxylative formal ring expansion process. A combination of trifluoromethylated six-membered benzoxazinanones with vinylethylene carbonates resulted in direct access to previously unknown trifluoromethyl-functionalized nine-membered heterocycles. The trifluoromethyl substituent at the C-4 position of benzoxazinanones plays an important role in this transformation. Diastereoselective transformations of the benzo-fused nine-membered heterocyclic alkene were also achieved to demonstrate the synthetic utility of the products. Investigation of the formation of other medium-sized rings as well as enantioselective variants of the reaction are presently under way in our laboratory.

## Conflicts of interest

There are no conflicts to declare.

## Supplementary Material

Supplementary information

Crystal structure data
