# Supplementary material for: Access to benzo-fused nine-membered heterocyclic alkenes with a trifluoromethyl carbinol moiety via a double decarboxylative formal ring-expansion process under palladium catalysis
Source: Chem Sci. 2018 Feb 23;9(13):3276–81. doi: 10.1039/c7sc05447e (PMC5915791; doi:10.1039/c7sc05447e)
Supplement: Supplementary file 1 [file SC-009-C7SC05447E-s001.pdf]

Electronic Supplementary Material (ESI) for Chemical Science.
This journal is © The Royal Society of Chemistry 2018

Electronic Supplementary Information

Access to benzo-fused nine-membered heterocyclic alkenes with a trifluoromethyl carbinol moiety via a double decarboxylative formal ring-expansion process under palladium catalysis

Pulakesh Das,# Satoshi Gondo,# Punna Nagender,# Hiroto Uno,# Etsuko Tokunaga# and Norio Shibata*#+

#Department of Nanopharmaceutical Sciences & Department of Frontier Materials, Nagoya Institute of Technology, Gokiso, Showa-ku, Nagoya 466-8555, Japan.

+Institute of Advanced Fluorine-Containing Materials, Zhejiang Normal University 688 Yingbin Avenue, 321004 Jinhua (China).

E-mail: nozshiba@nitech.ac.jp

Table of Contents:

1. Optimization Data of Double Decarboxylative (DDC) reaction…..........................S3
Table S1. Preliminary Investigation……………......................................S3
Table S2. Solvent screening……………….. S4
Table S3. Temperature screening…………………………………………….S4
Table S4. Catalyst and Additive screening…………………………………..S4
Table S5. Catalyst amount screening………………………………………...S5
2. Density functional theory (DFT) calculation………………………………………S5
3. Structural and electronic properties………………………………………………...S6
Figure S1. Frontier molecular orbitals of benzoxazinanone **1** derivatives…...S6
Table S6. Comparative studies of global chemical reactivity indices of benzoxazinanone **1** derivatives……………………………………………….…S7
4. LC-MS analysis of Pd-π-allyl complex **I**…………….…………………………..S7
Figure S2. LC-MS spectra of Pd-π-allyl complex **I**……...……......................S8
5. ^{19}F NMR studies ………………………………………………………………..…...S9
Figure S2. ^{19}F NMR investigation in between the reaction of **1a** and **2a** in DCE-d_4……………………………………………………………………...S10
6. General Information…………………………………………………………………S11
7. General experimental procedure for the synthesis of compound **S1**(**method A**)...S12
8. General experimental procedure for the synthesis of compound **S2** (**method B**)..S15
9. General experimental procedure for the synthesis of compound **S3** (**method C**).S18
10. General experimental procedure for the synthesis of substituted 4-trifluoromethyl benzoxazinanone **1** (**method D**)…………………………………………………S21
11. Typical procedure for the preparation of DDC products **3** (**method E**)……………………………………………………………….......S24
12. Characterization Data of Products **3**……..…………………………………….S25
13. Application of CF_3- benzoxazonine **3aa**………………………………….....S39
General procedure for preparation of **4**...S39
General procedure for preparation of **5**...S40
14. Preparation of compound **6b, 6c**..………………………………………...…S41
15. X-Ray crystallographic structures of **1a, 3aa, 3aj**, **4**…………...…………..……S42
16. References…………………………………………………………………........S43
17. NMR Data……………………………………………………………………..S44

1. Optimization Data of Double Decarboxylative (DDC) reaction

Table S1: Preliminary Investigation:[a]

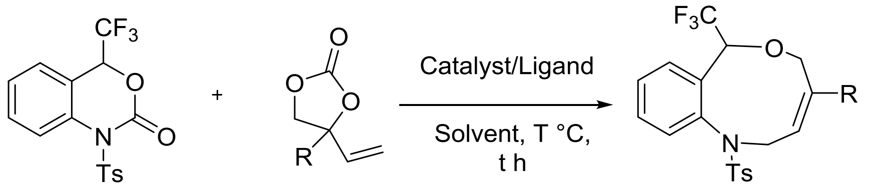

Entry	Catalyst	Ligand	Solvent	T (°C)	t (h)	Yield[b]
1[c]	10 mol % $Pd_2(dba)_3{\cdot}CHCl_3$	-	DCM	rt	24	-
2[d]	5 mol % $Pd_2(dba)_3{\cdot}CHCl_3$	-	THF	40	24	-
3	5 mol % $Pd_2(dba)_3{\cdot}CHCl_3$	10 mol % PCy_3	THF	40	24	-
4	5 mol % $Pd_2(dba)_3{\cdot}CHCl_3$	10 mol % dtbpmb	THF	40	24	-
5	5 mol % $Pd(PPh_3)_4$	-	Tol	50	24	47 (42)
**6**	**5 mol % $Pd(PPh_3)_4$**	**-**	**Tol**	**50**	**36**	**68 (66)**
7	5 mol % $Pd(PPh_3)_4$	-	Tol	80	12	55 (45)
8	5 mol % $Pd(PPh_3)_4$	-	Tol	100	12	16 (10)

[a] Experiments were performed with **1a** (0.1 mmol), **2a** (0.15 mmol) in 1mL solvent. [b] ^{19}F NMR yields with reference $PhCF_3$ and isolated yields are given in the parenthesis. [c,d] **2a** (0.12 mmol) was used.

PCy_3: Tricyclohexyl phosphine; dtbpmb: 1,2-Bis(di-^{t}butyl phosphinomethyl)benzene;

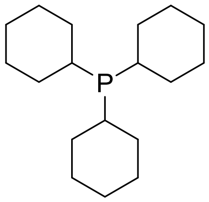

PCy_3

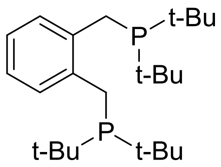

dtbpmb

Table S2: Solvent screening[a]

Entry	Catalyst	Solvent	T (°C)	t (h)	Yield[b]
1	5 mol % $Pd(PPh_3)_4$	Tol	50	36	68 (66)
2	5 mol % $Pd(PPh_3)_4$	THF	50	7	75 (70)
**3**	**5 mol % $Pd(PPh_3)_4$**	**DCE**	**50**	**12**	**83 (79)**
4	5 mol % $Pd(PPh_3)_4$	1,4-Dioxane	50	20	67 (62)
5	5 mol % $Pd(PPh_3)_4$	Xylene	50	24	78 (74)
6	5 mol % $Pd(PPh_3)_4$	Benzene	50	20	68 (64)
7	5 mol % $Pd(PPh_3)_4$	Acetonitrile	50	15	81 (74)

[a] Experiments were performed with **1a** (0.1 mmol), **2a** (0.15 mmol) in 1mL solvent [b] ^{19}F NMR yields with reference $PhCF_3$ and isolated yields are given in the parenthesis.

Table S3: Temperature screening[a]

Entry	Catalyst	Solvent	T (°C)	t (h)	Yield[b]
1	5 mol % $Pd(PPh_3)_4$	DCE	50	12	83 (79)
**2**	**5 mol % $Pd(PPh_3)_4$**	**DCE**	**80**	**12**	**91 (89)**
3	5 mol % $Pd(PPh_3)_4$	DCE	Reflux	12	40 (34)

[a] Experiments were performed with **1a** (0.1 mmol), **2a** (0.15 mmol) in 1mL DCE [b] ^{19}F NMR yields with reference $PhCF_3$ and isolated yields are given in the parenthesis.

Table S4: Catalyst and Additive screening[a]

Entry	Catalyst	Additive	Solvent	T (°C)	t (h)	Yield[b]
1	2 mol % $Pd(PPh_3)_4$	Et_3N (0.5 equiv)	DCE	50	12	34 (27)
2	2 mol % $Pd(PPh_3)_4$	K_2CO_3 (1.0 equiv)	DCE	50	12	75 (72)
3	2 mol % $Pd(PPh_3)_4$	Cs_2CO_3 (1.0 equiv)	DCE	50	12	72 (68)
4	2 mol % $Pd(PPh_3)_4$	K_2HPO_4 (1.0 equiv)	DCE	50	12	81 (79)
5	2 mol % $Pd(PPh_3)_4$	K_2HPO_4 (1.0 equiv)	DCE	40	12	68 (63)
6	2 mol % $Pd(PPh_3)_4$	K_2HPO_4 (1.0 equiv)	DCE	60	12	75 (71)
7	2 mol % $Pd(PPh_3)_4$	K_2HPO_4 (1.0 equiv)	DCE	reflux	12	44 (36)

[a] Experiments were performed with **1a** (0.1 mmol), **2a** (0.15 mmol) in 1mL DCE. [b] ^{19}F NMR yields with reference $PhCF_3$ and isolated yields are given in the parenthesis.

Table S5: Amount of catalyst screening[a]

Entry	Catalyst	Solvent	T (°C)	t (h)	Yield[b]
1	2.5 mol % $Pd(PPh_3)_4$	DCE	80	12	64 (59)
2	**5 mol % $Pd(PPh_3)_4$**	**DCE**	**80**	**12**	**91 (89)**
3	10 mol % $Pd(PPh_3)_4$	DCE	80	12	11

[a] Experiments were performed with **1a** (0.1 mmol), **2a** (0.15 mmol) in 1mL DCE [b] ^{19}F NMR yields with reference $PhCF_3$ and isolated yields are given in the parenthesis.

2. Density functional theory (DFT) calculation[1]

In order to explore the theoretical-experimental consistency, quantum chemical calculations were performed with complete geometry optimizations using standard Spartan' 14 software. Geometry optimization was carried out by B3LYP/6-311+G** level of theory. The chemical reactivity descriptors calculated using DFT are: total energy (E), chemical hardness (η), electronic chemical potential (μ) and electrophilicity (ω).
Chemical hardness (η) measures the resistance to change in the electron distribution or charge transfer and it associates with the stability and reactivity of a chemical system. On the basis of frontier molecular orbitals, chemical hardness corresponds to the gap between the HOMO and LUMO. Chemical hardness is approximated using equation 1

$$\eta = (E_{LUMO} - E_{HOMO})/2 \qquad (1)$$

where E_{LUMO} and E_{HOMO} are the LUMO and HOMO energies.
Electronic chemical potential (μ) is defined as the negative of electronegativity of a molecule and calculated using equation 2.

$$\mu = (E_{LUMO} + E_{HOMO})/2 \qquad (2)$$

Physically, μ describes the escaping tendency of electrons from an equilibrium system.
Global electrophilicity index (ω), is calculated using the electronic chemical potential and chemical hardness as shown in equation 3.

$$\omega = \mu^2/2\eta \qquad (3)$$

This index measures the propensity or capacity of a species to accept electrons. It is a measure of the stabilization in energy after a system accepts additional amount of electronic charge from the environment.

3. Structural and electronic properties

DFT calculations were performed for benzoxazinanone **1** derivatives. The contour plots of the frontier orbitals for the ground state are shown in Figure S1 including the Highest Occupied Molecular Orbital (HOMO) and Lowest Unoccupied Molecular Orbital (LUMO).

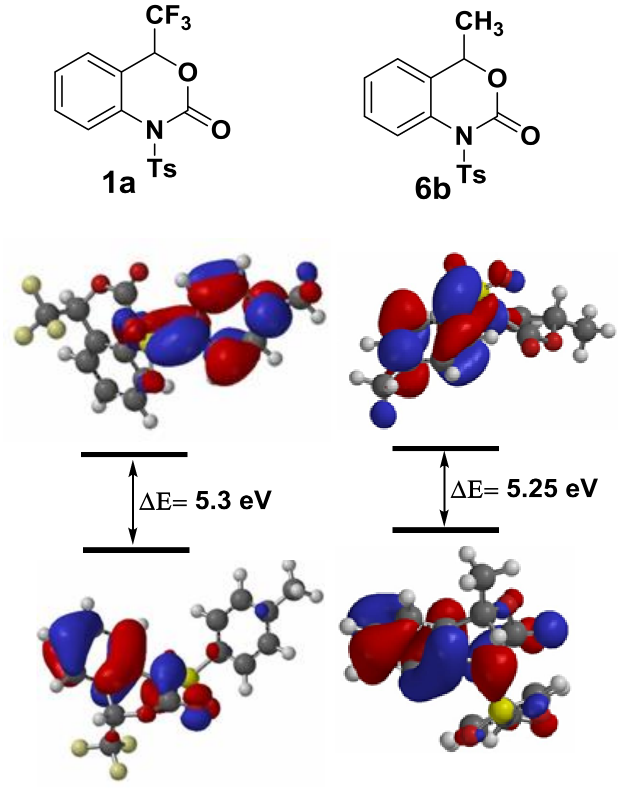

Figure S1. Frontier molecular orbitals of benzoxazinanone **1** derivatives.

Table S6 contains the computed global chemical reactivity indices for compounds **1a** and **6b**. The electrophilicity (ω) values for the derivatives **1a** and **6b** are 3.67 eV and 3.33 eV respectively. Among the derivatives, derivative **1a** is the strongest electrophilic compared to **6b**.

Table S6. Comparative studies of global chemical reactivity indices of benzoxazinanone **1** derivatives.

	**1a; R=CF3**	**6b; R=CH3**
Total Energy, E (au)	-1670.62	-1372.81
E_{HOMO} (eV)	-7.06	-6.81
E_{LUMO} (eV)	-1.76	-1.56
Dipole moment (debye)	6.83	4.54
Energy gap (Δ) (eV)	5.30	5.25
Chemical hardness, η(eV)	2.65	2.63
Electronic chemical potential, μ(eV)	- 4.41	- 4.18
Global electrophilicity index, ω(eV)	3.67	3.33

4. LC-MS analysis of Pd-π-allyl complex I

LC-MS analysis confirmed that the Pd-π-allyl complex **I** was formed during the progress of the reaction. LC-MS (ESI, m/z): $[M]^+$ 1103.85 (isotopic pattern) (Figure S2)

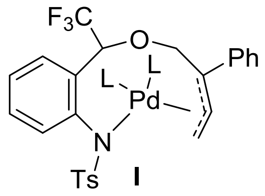

I

m/z= 1103.85
(LC-MS)

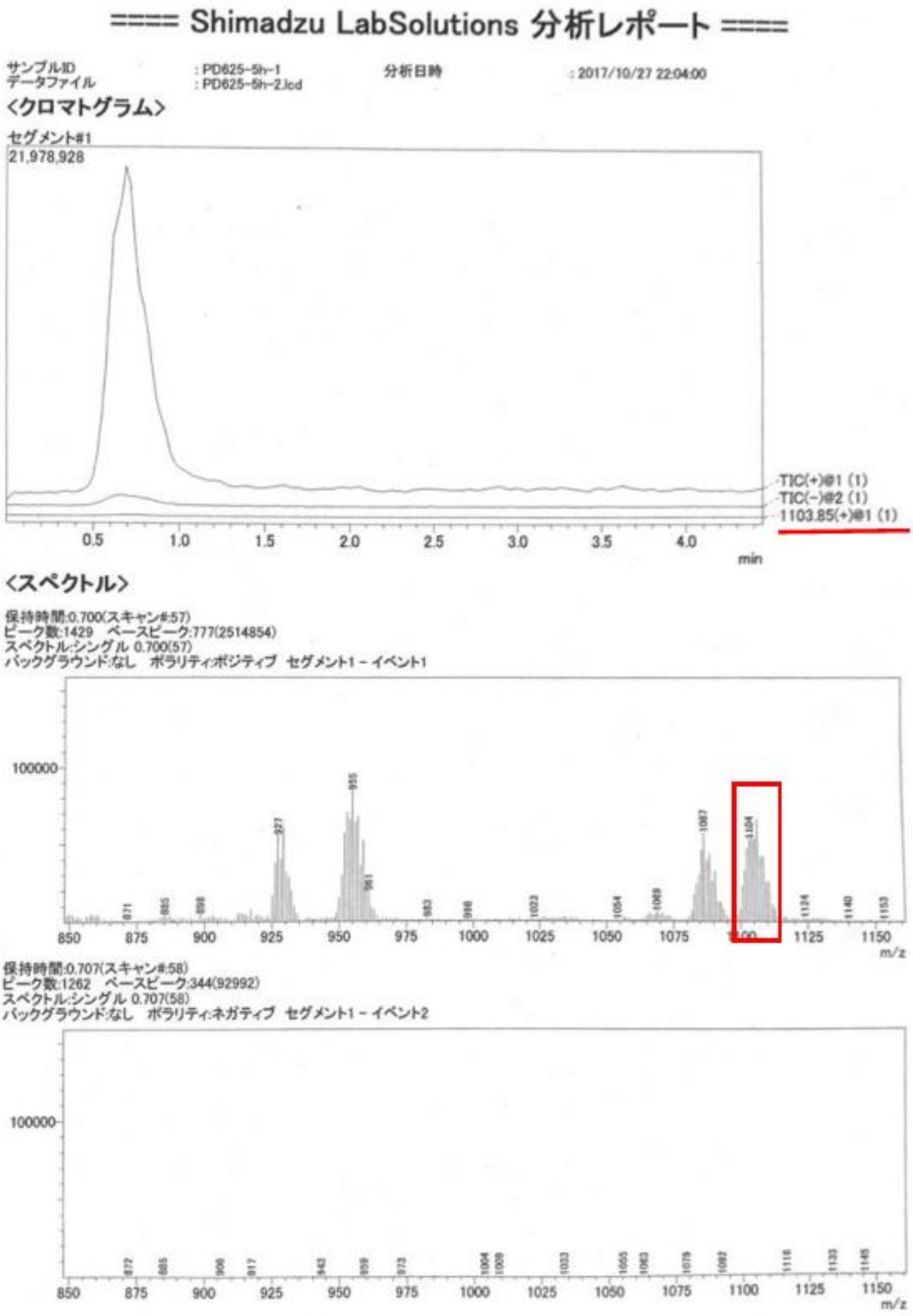

Figure S2. LC-MS spectra of Pd-π-allyl complex **I.**

5. ^{19}F NMR studies to observe the progress of the DDC reaction

To an oven dried NMR tube **1a** (0.03 mmol, 0.011 g, 1.0 equiv) was transferred inside the glove box. To it 0.5 mL DCE-d_4 was added and recorded the ^{19}F NMR [Figure S3 a)]. To the above solution, $Pd(PPh_3)_4$ (0.009 mmol, 0.0104 g, 0.3 equiv) was added and stirred at room temperature and recorded the NMR spectra after 4 h [Figure S3 b)]. **2a** was added to the above reaction mixture inside the glove box and recorded the crude ^{19}F NMR spectra after 10 min, 1 h, 12 h and 18 h [Figure S3 c)-f)] respectively. After 10 min of addition of **2a**, progress of the reaction was observed through the NMR spectra. After 18 h, i.e. completion of the reaction, we had added D_2O to the above reaction mixture and stirred vigorously to ensure complete mixing and recorded the NMR spectra [Figure S3 g)].

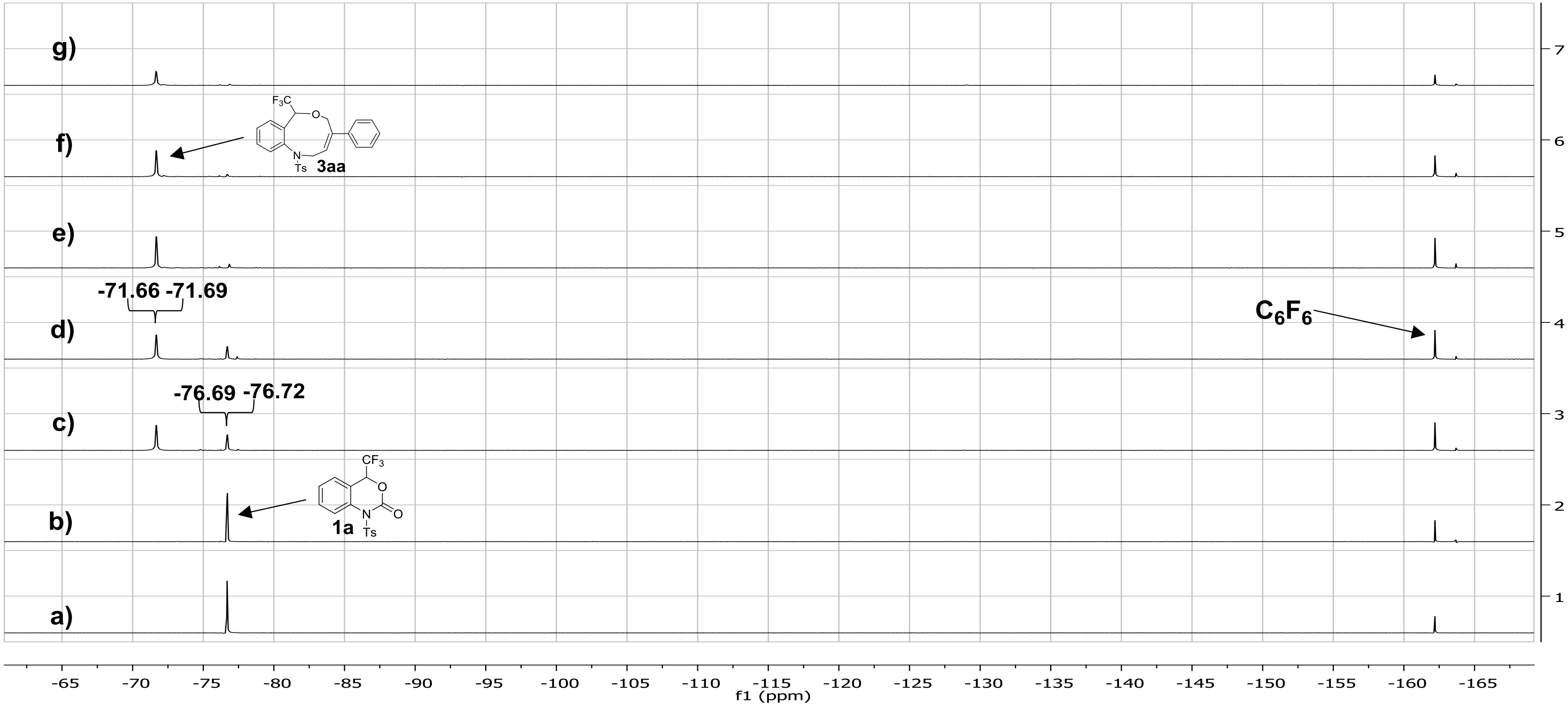

Figure S3. ^{19}F NMR investigation in between the reaction of 1a and 2a in DCE-d_4.

6. General information

All reactions were performed in oven-dried glassware under a positive pressure of nitrogen or argon. Solvents were transferred via syringe and were introduced into the reaction vessels through a rubber septum. All solvents were dried by standard method. All of the reactions were monitored by thin-layer chromatography (TLC) carried out on 0.25 mm Merck silica gel (60-F254). The TLC plates were visualized with UV light and 7% phosphomolybdic acid or $KMnO_4$ in water/heat or *p*-anisaldehyde solution/heat. All of the reaction products were purified by column chromatography. Column chromatography was carried out on a column packed with silica gel 60N spherical neutral size 63-210 mm. The ^{1}H NMR (300 MHz, 400 MHz and 500 MHz) and ^{19}F NMR (282 MHz) spectra (with Hexafluorobenzene (δ ppm −162.2) as an internal standard) as for solution in $CDCl_3$ were recorded on a Varian Mercury 300. ^{13}C NMR (126 MHz) spectra for solution in $CDCl_3$ was recorded on a BRUKER 500 UltraShieldTR . Chemical shifts (δ) are expressed in ppm downfield from internal TMS or C_6F_6. Chemical shifts (δ) are reported in ppm, and coupling constants (J) are in hertz (Hz). The following abbreviations were used to explain the multiplicities: s = singlet, d = doublet, t = triplet, q = quartet, m = multiplet, br = broad. Mass spectra were recorded on a SHIMADZU GCMS-QP5050A (EI-MS) and SHIMAZU LCMS-2020(ESI-MS). Infrared spectra were recorded on JASCO FT/IR-200 or a JASCO FT/IR-4100 spectrometer. Melting points were measured on Buchi M-565 device.

Commercially available chemicals were obtained from Aldrich Chemical Co., Alfa Aesar, TCI, Ark Farm and used as received unless otherwise stated. The residual solvent signals were used as references (TMS: δH = 0.00 ppm, δC =77.16 ppm; and C_6F_6: δF = −162.2

ppm). High resolution mass spectrometry (HRMS) was carried out on an electron impact ionization mass spectrometer with a micro-TOF analyzer.

7. General experimental procedure for the preparation of substituted ethyl (2-formylphenyl)carbamate S1 (method A)

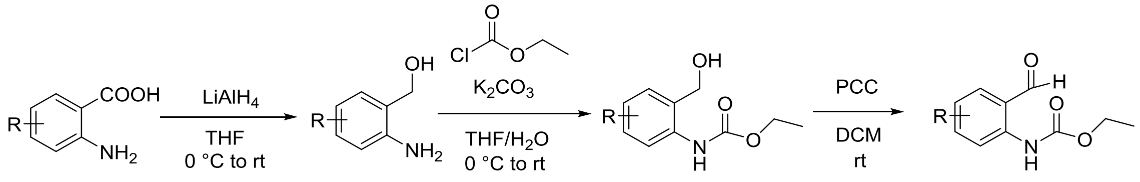

The substituted ethyl (2-formylphenyl)carbamate **S1** were prepared according to literature procedures[2,3,4] in three steps starting from substituted 2-aminobenzoic acid.

To a solution of substituted 2-amino-benzoic acid (20 mmol) in dry THF (30 mL) was added dropwise a solution of $LiAlH_4$ in THF (1M, 30 mL) while the temperature was maintained at 0 °C. The resulting mixture was allowed to warm to room temperature and was stirred for 2 h. The mixture was then hydrolyzed by dropwise addition of water (5 mL) and 5% NaOH (7 mL). The resulting suspension was filtered and the precipitate was washed with ethyl acetate. Then the combined organic phase was evaporated. The residue was recrystallized from ethyl acetate and petroleum ether, affording the corresponding alcohols quantitatively as a fine white or pale yellow solid.

To a solution of substituted 2-amino benzyl alcohol in saturated K_2CO_3 aq. and THF (v:v= 2:1, 45 mL) was added ethyl chloroformate (25 mmol). The mixture was stirred at room temperature for 3 h and extracted with EtOAc. The organic phase was dried over Na_2SO_4 and concentrated under reduced pressure to afford the desired substituted *N*-protected 2-aminobenzyl alcohol without purification.

In a flame dried 200 mL two neck round bottom flask, pyridinium chlorochromate (4.85 g, 22.5 mmol, 1.5 equiv) was suspended in anhydrous DCM (60 mL) fitted with a 100-mL addition funnel. A solution of substituted *N*-protected 2-aminobenzyl alcohol (3.13 g, 15 mmol, 1.0 equiv) in anhydrous DCM (60 mL) was placed in the addition funnel and dropwise added to the flask. As the addition progressed, the reaction became black and opaque. The reaction was stirred for 2.5 h at ambient temperature. Upon completion, the black solution was decanted from the black precipitate and filtered through a short pad of silica gel. The precipitate was washed with DCM (15 mL × 3) to ensure complete transfer.

The DCM was removed *in vacuo* and the resulting residue was purified by flash chromatography on silica gel (using 8:2 hexane/ethyl acetate).
Starting materials **S1a** was reported in previous articles.[5]
Stabilized vinylethylene carbonates **2** were prepared according to the known procedure.[6]

Ethyl (2-formyl-4-methylphenyl)carbamate (S1b)

Cream colored solid, 90% yield, mp. 39.7-42.2 °C. Using the general method A, in a flame dried 200 mL two neck round bottom flask, pyridinium chlorochromate (4.85 g, 22.5 mmol, 1.5 equiv) was suspended in anhydrous DCM (60 mL) fitted with a 100-mL addition funnel. A solution of substituted *N*-protected 2-amino-5-methylbenzyl alcohol (3.13 g, 15 mmol, 1.0 equiv) in anhydrous DCM (60 mL) was placed in the addition funnel and added dropwise to the flask. The reaction was stirred for 2.5 h at ambient temperature. After completion of the reaction DCM was removed *in vacuo* and the resulting residue was purified by flash chromatography on silica gel (using 8:2 hexane/ethyl acetate) to obtain the pure product.

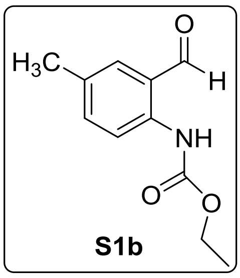

S1b

^{1}H NMR (300 MHz, $CDCl_3$) δ 10.46 (s, 1H, -CHO), 9.86 (s, 1H, -NH), 8.45 – 8.26 (m, 2H, ArH), 7.42 (s, 1H, ArH), 4.24 (q, *J* = 7.1 Hz, 2H, -O-CH_2), 2.37 (s, 3H, Ar-CH_3), 1.33 (t, *J* = 7.1 Hz, 3H, CH_3). **^{13}C NMR** (126 MHz, $CDCl_3$) δ 195.28, 153.92, 139.11, 136.98, 136.23, 131.49, 121.36, 118.43, 61.42, 20.46, 14.62. **IR** (KBr): 2975, 2927, 2873, 1737, 1660, 1590, 1529, 1396, 1319, 1058, 887, 767. cm^{-1}. **HRMS** (ESI) calculated for $C_{11}H_{13}NO_3Na$ $[M+Na]^+$ 230.0793, found 230.0790.

Ethyl (5-fluoro-2-formylphenyl)carbamate (S1c)

Cream colored solid, 67% yield, mp 30.6-36.8 °C. Using the general method A, in a flame dried 200 mL two neck round bottom flask, pyridinium chlorochromate (3.23 g, 15 mmol, 1.5 equiv) was suspended in anhydrous DCM (50 mL) fitted with a 100-mL addition funnel. A solution of substituted *N*-protected 2-amino-4-fluorobenzyl alcohol (2.1 g, 10 mmol, 1.0 equiv) in anhydrous DCM (50 mL) was placed in the addition funnel and added dropwise to the flask. The reaction was stirred for 2.5 h at ambient temperature. After completion of the reaction, DCM was removed *in vacuo* and the resulting residue was purified by flash chromatography on silica gel (using 8:2 hexane/ethyl acetate) to obtain the pure product. **^{1}H NMR** (300 MHz, $CDCl_3$) δ 10.75 (s, 1H,-CHO), 9.84 (s, 1H, NH), 8.23 (d, *J* = 12.0 Hz, 1H, ArH), 7.74 – 7.53 (m, 1H, ArH), 6.95 – 6.72 (m, 1H, ArH), 4.24 (q, *J* = 7.1 Hz, 2H, -OCH_2), 1.33 (t, *J*

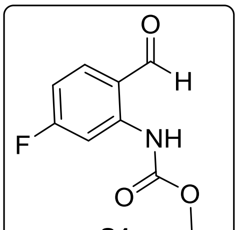

S1c

= 7.1 Hz, 3H,CH_3). **^{13}C NMR** (126 MHz, $CDCl_3$) δ 193.70, 167.53 (d, *J* = 256.2 Hz), 153.66, 144.05 (d, *J* = 13.7 Hz), 138.71 (d, *J* = 12.0 Hz), 118.30 (d, *J* = 1.8 Hz), 109.59 (d, *J* = 23.4 Hz), 105.93 (d, *J* = 28.8 Hz), 61.85, 14.56. **IR** (KBr): 3250, 3117, 2991, 1737, 1678, 1536, 1401, 1324, 1198, 1114, 872, 770, 725 cm^{-1}. **HRMS** (ESI) calculated for $C_{10}H_9FNO_3$ $[M-H]^-$ 210.0566, found 210.0558.

Ethyl (4-chloro-2-formylphenyl)carbamate (S1d)

Cream colored solid, 84% yield, mp. 73.4-75.1 °C. Using the general method A, in a flame dried 200 mL two neck round bottom flask, pyridinium chlorochromate (3.6 g, 16.5 mmol, 1.5 equiv) was suspended in anhydrous DCM (40 mL) fitted with a 100-mL addition funnel. A solution of substituted *N*-protected 2-amino-5-chlorobenzyl alcohol (2.52 g, 11 mmol, 1.0 equiv) in anhydrous DCM (40 mL) was placed in the addition funnel and added dropwise to the flask. The reaction was stirred for 2.5 h at ambient temperature. After completion of the reaction DCM was removed *in vacuo* and the resulting residue was purified by flash chromatography on silica gel (using 8:2 hexane/ethyl acetate) to obtain the pure product. **^{1}H NMR** (300 MHz, $CDCl_3$) δ 10.47 (s, 1H, CHO), 9.85 (s, 1H, NH), 8.46 (d, *J* = 9.0 Hz, 1H, ArH), 7.61 (d, *J* = 2.5 Hz, 1H, ArH), 7.53 (dd, *J* = 9.0, 2.5 Hz, 1H, ArH), 4.25 (q, *J* = 7.1 Hz, 2H, -OCH_2), 1.34 (t, *J* = 7.1 Hz, 3H, CH_3). **^{13}C NMR** (126 MHz, $CDCl_3$) δ 193.97, 153.67, 140.02, 135.96, 135.06, 127.00, 122.29, 120.14, 61.77, 14.56. **IR** (KBr): 2985, 2911, 2871, 2765, 1727, 1590, 1309, 1093, 728, 651 cm^{-1}. **HRMS** (ESI) calculated for $C_{10}H_9ClNO_3$ $[M-H]^-$ 226.0271, found 226.0269.

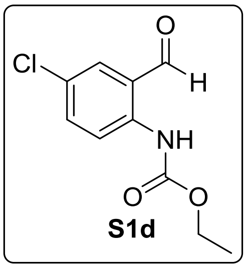

Ethyl (4-bromo-2-formylphenyl)carbamate (S1e)

White powder, 91% yield, mp. 90.5-91.5 °C. Using the general method A, in a flame dried 200 mL two neck round bottom flask, pyridinium chlorochromate (3.4 g, 15.9 mmol, 1.5 equiv) was suspended in anhydrous DCM (40 mL) fitted with a 100-mL addition funnel. A solution of substituted *N*-protected 2-amino-5-bromobenzyl alcohol (2.9 g, 10.6 mmol, 1.0 equiv) in anhydrous DCM (40 mL) was placed in the addition funnel and added dropwise to the flask. The reaction was stirred for 2.5 h at ambient temperature. After completion of the reaction DCM was removed *in vacuo* and the resulting residue was purified by flash chromatography on silica gel (using 8:2 hexane/ethyl acetate) to obtain the pure product. **^{1}H NMR** (500 MHz, $CDCl_3$) δ 10.47 (s, 1H, -CHO), 9.84 (s, 1H, NH), 8.40 (d, *J* = 9.0 Hz,

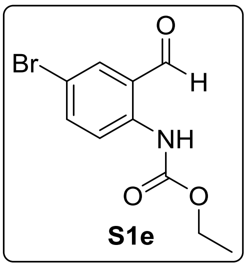

1H, ArH), 7.75 (d, J = 2.4 Hz, 1H, ArH), 7.69 – 7.65 (m, 1H, ArH), 4.25 (q, J = 7.1 Hz, 2H, $-OCH_2$), 1.34 (t, J = 7.1 Hz, 3H, CH_3). **^{13}C NMR** (126 MHz, $CDCl_3$) δ 193.91, 153.64, 140.49, 138.80, 138.10, 122.69, 120.42, 114.00, 61.80, 14.56. **IR** (KBr): 2983, 2867, 1729, 1585, 1386, 1309, 1058, 885, 808, 767, 715 cm^{-1}. **HRMS** (ESI) calculated for $C_{10}H_9BrNO_3$ $[M-H]^-$ 269.9766, found 269.9768.

8. General experimental procedure for the synthesis of CF_3 substituted alcohol S2 (method B)[7]

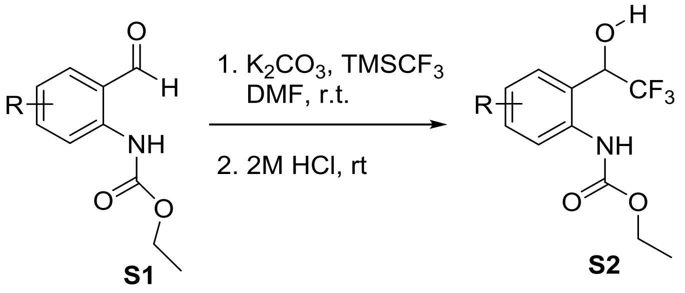

In a flame dried 100 mL round bottom flask, aldehyde **S1a** (10 mmol, 1.932 g, 1.0 equiv) and $TMSCF_3$ (neat, 20 mmol, 3 mL, 2.0 equiv) was suspended in anhydrous DMF (20 mL). To this solution dry K_2CO_3 (10 mol %, 0.138 g, 0.1 equiv) was added and the mixture was stirred vigorously at room temperature under N_2 atmosphere. Completion of the reaction was monitored by TLC. To this reaction mixture, 2M HCl solution (4 mL) was added and stirred for 3 h at room temperature. The reaction mixture was then extracted with ethyl acetate (3×30 mL). Combined organic layers were finally washed with brine solution, dried over anhydrous Na_2SO_4, and then solvent was removed under reduced pressure. The crude product was further purified by column chromatography (using 8:2 hexane/ethyl acetate) to afford pure product **S2a**. The characterization data of **S2a** are summarized below.

Ethyl (2-(2,2,2-trifluoro-1-hydroxyethyl)phenyl)carbamate (S2a)

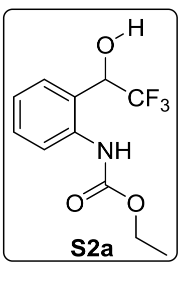

White solid, 62% yield, mp. 104.2-106.9 °C. **^{1}H NMR** (500 MHz, $CDCl_3$) δ 7.83 (d, J = 25.1 Hz, 1H, ArH), 7.40 – 7.28 (m, 1H, ArH), 7.24 (d, J = 7.7 Hz, 1H, ArH), 7.10 (t-like, J = 7.5 Hz, 1H, ArH), 5.08 – 5.01 (m, 1H, $CH-CF_3$), 4.42 (d-like, J = 4.6 Hz, 1H, -OH), 4.16 (q, J = 7.1 Hz, 2H, $-OCH_2$), 1.28 (t, J = 7.1 Hz, 3H, CH_3). **^{13}C NMR** (126 MHz, $CDCl_3$) δ 154.93, 137.23, 130.26, 129.64, 124.58 (q, J = 282.8 Hz), 124.31, 123.71, 123.04, 72.64 (q, J = 33.6 Hz), 61.79, 14.52. **^{19}F NMR** (282 MHz, $CDCl_3$) δ -77.69 (d, J = 7.0 Hz, 3F). **IR** (KBr): 3334, 2991, 1702, 1594, 1452, 1357, 1261, 1170, 1068, 838,

754 cm^{-1}. **HRMS** (ESI) calculated for $C_{11}H_{12}F_3NO_3Na$ $[M+Na]^+$ 286.0667, found 286.0673.

Ethyl (4-methyl-2-(2,2,2-trifluoro-1-hydroxyethyl)phenyl)carbamate (S2b)

Light yellow solid, 61% yield, mp. 131.5-133.0 °C. Using the general method B, in a flame dried 100 mL round bottom flask, aldehyde **S1b** (5 mmol, 1.03 g, 1.0 equiv) and $TMSCF_3$ (neat, 10 mmol, 1.5 mL, 2.0 equiv) was suspended in anhydrous DMF (15 mL). To this solution dry K_2CO_3 (10 mol %, 0.07 g, 0.1 equiv) was added and the mixture was stirred vigorously at room temperature under N_2 atmosphere. After completion of the reaction, 2M HCl solution (2 mL) was added and stirred for 3 h at room temperature. The reaction mixture was then extracted with ethyl acetate (3×30 mL) and purified by column chromatography (using 8:2 hexane/ethyl acetate) to afford pure product **S2b**. **^{1}H NMR** (500 MHz, $CDCl_3$) δ 7.19 (d, *J* = 1.9 Hz, 1H, ArH), 7.17 (d, *J* = 1.5 Hz, 1H, ArH), 7.15 (s, 1H, ArH), 5.14 – 5.06 (m, 1H, -CH-CF_3), 4.19 (q, *J* = 7.1 Hz, 2H, -OCH_2), 3.62 (d-like, *J* = 4.6 Hz, 1H, OH), 2.33 (s, 3H, Ph-CH_3), 1.30 (t, *J* = 7.1 Hz, 3H, CH_3). **^{13}C NMR** (126 MHz, $CDCl_3$) δ 154.99, 134.65, 134.34, 131.02, 129.73, 124.62, 124.61 (q, *J* = 282.5 Hz), 123.86, 71.88 (q, *J* = 28.5 Hz), 61.72, 20.94, 14.63. **^{19}F NMR** (282 MHz, $CDCl_3$) δ -77.46. **IR** (KBr): 3307, 2992, 2744, 1693, 1602, 1531, 1348, 1261, 1166, 1068, 840, 775 cm^{-1}. **HRMS** (ESI) calculated for $C_{12}H_{14}F_3NO_3Na$ $[M+Na]^+$ 300.0823, found 300.0825.

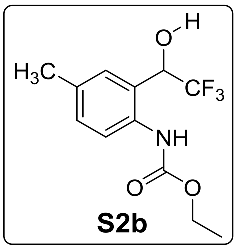

S2b

Ethyl (5-fluoro-2-(2,2,2-trifluoro-1-hydroxyethyl)phenyl)carbamate (S2c)

White solid, 77% yield, mp. 115.7-117.5 °C. Using the general method B, in a flame dried 100 mL round bottom flask, aldehyde **S1c** (3.8 mmol, 0.8 g, 1.0 equiv) and $TMSCF_3$ (neat, 5.3 mmol, 0.8 mL, 1.4 equiv) was suspended in anhydrous DMF (10 mL). To this solution dry K_2CO_3 (10 mol %, 0.05 g, 0.1 equiv) was added and the mixture was stirred vigorously at room temperature under N_2 atmosphere. After completion of the reaction, 2M HCl solution (1 mL) was added and stirred for 3 h at room temperature. The reaction mixture was then extracted with ethyl acetate (3×30 mL) and purified by column chromatography (using 8:2 hexane/ethyl acetate) to afford pure product **S2c**. **^{1}H NMR** (500 MHz, $CDCl_3$) δ 8.08 (br s, 1H, -NH), 7.79 (d, *J* = 8.7 Hz, 1H, ArH), 7.23 – 7.13 (m, 1H, ArH), 6.79 – 6.75 (m, 1H, ArH), 5.18 – 4.99 (m, 1H,-CH-CF_3), 4.21 (q, *J* = 7.1 Hz, 2H, -CH_2), 3.72 (br s, 1H, -OH), 1.31 (t, *J* = 7.1 Hz, 3H, -CH_3). **^{13}C NMR** (126 MHz, $CDCl_3$) δ 163.59 (d, *J* = 247.8 Hz), 153.98, 139.49 (d, *J* = 11.5 Hz), 131.16

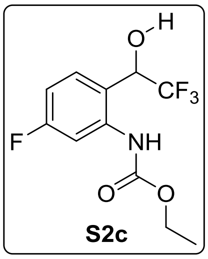

S2c

(d, J = 10.0 Hz), 124.26 (q, J = 282.7 Hz), 117.36, 110.40 (d, J = 22.0 Hz), 109.24 (d, J = 27.1 Hz), 73.07 (q, J = 33.0 Hz), 61.74, 14.41. **^{19}F NMR** (282 MHz, $CDCl_3$) δ -77.92 (d, J = 7.0 Hz, 3F), -109.93 (s, 1F). **IR** (KBr): 3322, 1700, 1544, 1444, 1270, 1171, 1125, 1063, 811 cm^{-1}. **HRMS** (ESI) calculated for $C_{11}H_{11}F_4NO_3Na$ $[M+Na]^+$ 304.0573, found 304.0578.

Ethyl (4-chloro-2-(2,2,2-trifluoro-1-hydroxyethyl)phenyl)carbamate (S2d)

White solid, 62% yield, mp. 95.2-98.7 °C. Using the general method B, in a flame dried 100 mL round bottom flask, aldehyde **S1d** (9 mmol, 2.0 g, 1.0 equiv) and $TMSCF_3$ (neat, 18 mmol, 2.66 mL, 2.0 equiv) was suspended in anhydrous DMF (20 mL). To this solution dry K_2CO_3 (10 mol %, 0.12 g, 0.1 equiv) was added and the mixture was stirred vigorously at room temperature under N_2 atmosphere. After completion of the reaction, 2M HCl solution (2 mL) was added and stirred for 3 h at room temperature. The reaction mixture was then extracted with ethyl acetate (3 × 30 mL) and purified by column chromatography (using 8:2 hexane/ethyl acetate) to afford pure product **S2d**. **^{1}H NMR** (300 MHz, $CDCl_3$) δ 7.78 (d, J = 8.1 Hz, 1H, ArH), 7.70 (s, 1H, ArH), 7.33 (d, J = 8.8 Hz, 1H, ArH), 5.11 – 5.00 (m, 1H, -CH-CF_3), 4.19 (q, J = 7.0 Hz, 2H, -CH_2), 1.30 (t, J = 7.4 Hz, 3H, CH_3). **^{13}C NMR** (126 MHz, $CDCl_3$) δ 154.59, 135.83, 130.34, 129.63, 129.41, 125.22, 124.48, 124.28 (q, J = 283.0 Hz), 72.08 (q, J = 33.1 Hz), 61.99, 14.55. **^{19}F NMR** (282 MHz, $CDCl_3$) δ -77.78 (d, J = 7.4 Hz, 3F). **IR** (KBr): 3289, 2992, 1693, 1589, 1529, 1407, 1303, 1174, 910, 840, 746 cm^{-1}. **HRMS** (ESI) calculated for $C_{11}H_{11}ClF_3NO_3Na$ $[M+Na]^+$ 320.0277, found 320.0273.

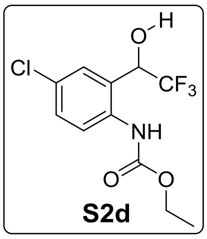

Ethyl (4-bromo-2-(2,2,2-trifluoro-1-hydroxyethyl)phenyl)carbamate (S2e)

White solid, 79% yield, mp. 86.0-89.2 °C. Using the general method B, in a flame dried 100 mL round bottom flask, aldehyde **S1e** (9.5 mmol, 2.58 g, 1.0 equiv) and $TMSCF_3$ (neat, 19 mmol, 2.81 mL, 2.0 equiv) was suspended in anhydrous DMF (25 mL). To this solution dry K_2CO_3 (10 mol %, 0.131 g, 0.1 equiv) was added and the mixture was stirred vigorously at room temperature under N_2 atmosphere. After completion of the reaction, 2M HCl solution (2 mL) was added and stirred for 3 h at room temperature. The reaction mixture was then extracted with ethyl acetate (3 × 30 mL) and purified by column chromatography (using 8:2 hexane/ethyl acetate) to afford pure product **S2e**. **^{1}H NMR** (500 MHz, $CDCl_3$) δ 7.79 (br s, 1H, NH), 7.75 (s, 1H, ArH), 7.47 (d, J = 2.3 Hz, 1H, ArH), 7.40 (d, J = 1.9 Hz, 1H, ArH), 5.07 – 5.00 (m, 1H,

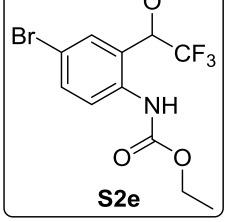

-CH-CF_3), 4.37 (br d, J = 4.5 Hz, 1H, -OH), 4.18 (q, J = 7.1 Hz, 2H, -CH_2), 1.30 (t, J = 7.1 Hz, 3H, CH_3). **^{13}C NMR** (126 MHz, $CDCl_3$) δ 154.58, 138.90, 138.20, 136.42, 133.25, 132.36, 124.55, 123.17 (q, J = 283.5 Hz), 72.19 (q, J = 32.5 Hz), 62.01, 14.53. **^{19}F NMR** (282 MHz, $CDCl_3$) δ -71.94 (d, J = 7.9 Hz, 3F). **IR** (KBr): 3340, 2992, 1708, 1587, 1513, 1398, 1178, 885, 838, 728 cm^{-1}. **HRMS** (ESI) calculated for $C_{11}H_{10}BrF_3NO_3$ [M-H]$^-$ 339.9796, found 339.9790.

9. General experimental procedure for the synthesis of substituted 4-(trifluoromethyl)-1,4-dihydro-benzoxazin-2-one S3 (method C)

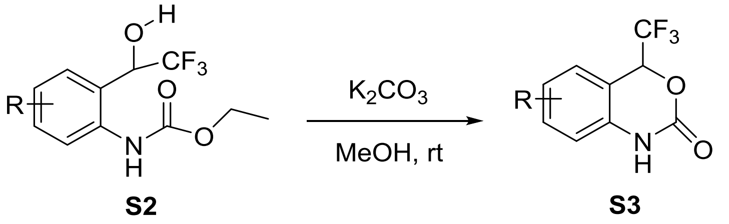

In a flame dried 100 mL round bottom flask, compound **S2a** (5 mmol, 1.316 g, 1.0 equiv) was suspended in anhydrous MeOH (30 mL). To this solution dry K_2CO_3 (1 mmol, 1.037 g, 1.5 equiv) was added and the mixture was stirred overnight at room temperature under Ar atmosphere. After completion of the reaction, the reaction mixture was concentrated under reduced pressure. The reaction mixture was then extracted with ethyl acetate (3 × 30 mL). Combined organic layers were finally washed with brine solution, dried over anhydrous Na_2SO_4, and then solvent was removed under reduced pressure. The crude product was purified by flash column chromatography (using 8:2 hexane/ethyl acetate) to obtain the pure product **S3a.** The characterization data of **S3a** are summarized below.

4-(Trifluoromethyl)-1,4-dihydro-2*H*-benzo[*d*][1,3]oxazin-2-one (S3a)

White solid, 98% yield, mp. 179.9-182.1 °C. **^{1}H NMR** (300 MHz, $CDCl_3$) δ 7.46 – 7.37 (m, 1H, ArH), 7.27 (d, J = 0.8 Hz, 1H, ArH), 7.20 – 7.11 (m, 1H, ArH), 6.92 (d, J = 8.0 Hz, 1H, ArH), 5.66 (q, J = 6.5 Hz, 1H, -CH-CF_3). **^{13}C NMR** (126 MHz, $CDCl_3$) δ 149.83, 135.36, 131.44, 127.07, 124.04, 122.82 (q, J = 284.3 Hz), 115.00, 110.89, 76.61 (q, J = 34.4 Hz). **^{19}F NMR** (282 MHz, $CDCl_3$) δ -79.56 (d, J = 6.5 Hz, 3F). **IR** (KBr):3170, 3114, 3004, 2944, 1995, 1725, 1608, 1502, 1384, 1274, 1186, 1081, 854, 755 cm^{-1}. **HRMS** (ESI) calculated for $C_9H_6F_3NO_2Na$ [M+Na]$^+$ 240.0248, found 240.0238.

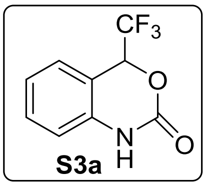

6-Methyl-4-(trifluoromethyl)-1,4-dihydro-2*H*-benzo[*d*][1,3]oxazin-2-one (S3b)

Dirty white solid, 78% yield, mp. 207.9-210.2 °C. Using the general method C, in a flame dried 100 mL round bottom flask, compound **S2b** (2.85 mmol, 0.79 g, 1.0 equiv) was suspended in anhydrous MeOH (25 mL). To this solution dry K_2CO_3 (2.85 mmol, 0.4 g, 1.0 equiv) was added and the mixture was stirred overnight at room temperature under Ar atmosphere. After completion of the reaction, the reaction mixture was concentrated, extracted with ethyl acetate and the crude product was purified by flash column chromatography (using 6:4 hexane/ethyl acetate) to obtain the pure product **S3b**. **^{1}H NMR** (300 MHz, $CDCl_3$) δ 8.37 (br s, 1H, NH), 7.19 (d, *J* = 8.1 Hz, 1H, ArH), 7.04 (s, 1H, ArH), 6.79 (d, *J* = 8.1 Hz, 1H, ArH), 5.58 (q, *J* = 6.5 Hz, 1H, -CH-CF_3), 2.34 (s, 3H, CH_3). **^{13}C NMR** (126 MHz, $CDCl_3$) δ 149.73, 133.85, 132.93, 132.01, 127.36, 122.86 (q, *J* = 284.76 Hz), 114.79, 110.78, 76.48 (q, *J* = 34.02 Hz), 20.91. **^{19}F NMR** (282 MHz, $CDCl_3$) δ -79.42 (d, *J* = 6.5 Hz, 3F). **IR** (KBr): 2950, 2346, 1724, 1621, 1425, 1299, 1187, 1135, 931, 734 cm^{-1}. **HRMS** (ESI) calculated for $C_{10}H_8F_3NO_2Na$ $[M+Na]^+$ 254.0405, found 254.0405.

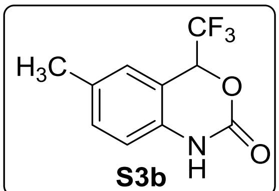

S3b

7-Fluoro-4-(trifluoromethyl)-1,4-dihydro-2*H*-benzo[*d*][1,3]oxazin-2-one (S3c)

White solid, 74% yield, mp. 170.5-174.5 °C. Using the general method C, in a flame dried 100 mL round bottom flask, compound **S2c** (2.85 mmol, 0.8 g, 1.0 equiv) was suspended in anhydrous EtOH (15 mL). To this solution dry K_2CO_3 (2.85 mmol, 0.39 g, 1.0 equiv) was added and the mixture was stirred overnight at room temperature under Ar atmosphere. After completion of the reaction, the reaction mixture was concentrated, extracted with ethyl acetate and the crude product was purified by flash column chromatography (using 6:4 hexane/ethyl acetate) to obtain the pure product **S3c**. **^{1}H NMR** (300 MHz, $CDCl_3$) δ 8.80 (br s, 1H, NH), 7.22 (d, *J* = 6.5 Hz, 1H, ArH), 6.95 – 6.77 (m, 1H, ArH), 6.67 (dd, *J* = 8.8, 2.1 Hz, 1H, ArH), 5.63 (q, *J* = 6.4 Hz, 1H, -CH-CF_3). **^{13}C NMR** (126 MHz, $CDCl_3$) δ 164.39 (d, *J* = 250.8 Hz), 149.38, 137.17 (d, *J* = 11.3 Hz), 128.99 (d, *J* = 10.0 Hz), 122.68 (q, *J* = 283.9 Hz), 111.31 (d, *J* = 22.6 Hz), 106.71, 102.75 (d, *J* = 26.5 Hz), 76.29 (q, *J* = 34.8 Hz). **^{19}F NMR** (282 MHz, $CDCl_3$) δ -79.75 (d, *J* = 6.4 Hz, 3F), -108.16 – -108.28 (m, 1F). **IR** (KBr): 3114, 3089, 2924, 1618, 1522, 1499, 1421, 1378, 1266, 1199, 1137, 1113, 858, 736 cm^{-1}. **HRMS** (ESI) calculated for $C_9H_4F_4NO_2$ $[M-H]^-$ 234.0178, found 234.0162.

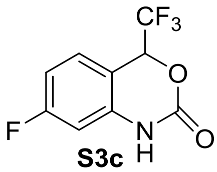

S3c

6-Chloro-4-(trifluoromethyl)-1,4-dihydro-2*H*-benzo[*d*][1,3]oxazin-2-one (S3d)

Cream color solid, 82% yield, mp. 216.8-220.4 °C. Using the general method C, in a flame dried 100 mL round bottom flask, compound **S2d** (5.4 mmol, 1.61 g, 1.0 equiv) was suspended in anhydrous MeOH (50 mL). To this solution dry K_2CO_3 (6.5 mmol, 0.89 g, 1.2 equiv) was added and the mixture was stirred overnight at room temperature under Ar atmosphere. After completion of the reaction, the reaction mixture was concentrated, extracted with ethyl acetate and the crude product was purified by flash column chromatography (using 8:2 hexane/ethyl acetate) to obtain the pure product **S3d**. **^{1}H NMR** (300 MHz, $CDCl_3$) δ 8.42 (br s, 1H, NH), 7.38 (d, *J* = 8.3 Hz, 1H, ArH), 7.26 (s, 1H, ArH), 6.85 (d, *J* = 8.5 Hz, 1H, ArH), 5.60 (q, *J* = 6.3 Hz, 1H, -CH-CF_3). **^{13}C NMR** (126 MHz, $CDCl_3$) δ 148.99, 134.04, 131.62, 129.33, 127.14, 122.57 (q, *J* = 284.3 Hz), 116.20, 112.44, 76.10 (q, *J* = 34.6 Hz). **^{19}F NMR** (282 MHz, $CDCl_3$) δ -79.30 (d, *J* = 6.3 Hz, 3F). **IR** (KBr): 2977, 1727, 1600, 1423, 1367, 1270, 1189, 1083, 854, 767 cm^{-1}. **HRMS** (ESI) calculated for $C_9H_4ClF_3NO_2$ $[M-H]^-$ 249.9883, found 249.9892.

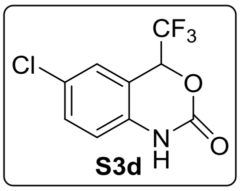

6-Bromo-4-(trifluoromethyl)-1,4-dihydro-2*H*-benzo[*d*][1,3]oxazin-2-one (S3e)

Brown color solid, 68% yield, mp. 213.8-215.6 °C. Using the general method C, in a flame dried 100 mL round bottom flask, compound **S2e** (7.2 mmol, 2.45 g, 1.0 equiv) was suspended in anhydrous MeOH (50 mL). To this solution dry K_2CO_3 (8.64 mmol, 1.2 g, 1.2 equiv) was added and the mixture was stirred overnight at room temperature under Ar atmosphere. After completion of the reaction, the reaction mixture was concentrated, extracted with ethyl acetate and the crude product was purified by flash column chromatography (using 8:2 hexane/ethyl acetate) to obtain the pure product **S3e**. **^{1}H NMR** (500 MHz, $CDCl_3$) δ 8.27 (br s, 1H, NH), 7.53 – 7.50 (m, 1H, ArH), 7.40 (s, 1H, ArH), 6.79 (d, *J* = 8.5 Hz, 1H, ArH), 5.60 (q, *J* = 6.6 Hz, 1H, -CH-CF_3). **^{13}C NMR** (126 MHz, $CDCl_3$) δ 148.93, 134.51, 134.49, 129.97, 122.56 (q, *J* = 285.6 Hz), 116.48, 116.39, 112.81, 75.98 (q, *J* = 34.8 Hz). **^{19}F NMR** (282 MHz, $CDCl_3$) δ -71.94 (d, *J* = 6.6 Hz, 3F). **IR** (KBr): 3151, 2003, 1710, 1600, 1417, 1365, 1272, 1191, 1139, 1081, 854, 767 cm^{-1}. **HRMS** (ESI) calculated for $C_9H_4BrF_3NO_2$ $[M-H]^-$ 293.9378. found 293.9403.

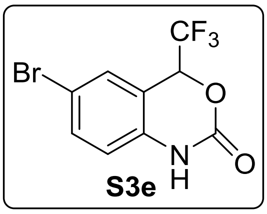

4-Methyl-1,4-dihydro-2*H*-benzo[*d*][1,3]oxazin-2-one (S3f)

White solid, 80% yield, mp. 100.6-104.8 °C. Using the general method B, in a flame dried 100 flame dried 100 mL round bottom flask, aldehyde **S1a** (2.59 mmol, 0.5 g, 1.0 equiv) and CH_3MgBr in THF solution (3.0 M, 1 mL) was suspended in anhydrous THF (10 mL) at 0 °C and the mixture was stirred vigorously at room temperature under N_2 atmosphere. After completion of the reaction, mixture was concentrated on reduced pressure and re-dissolve in EtOH (15 mL). To this solution dry K_2CO_3 (2.59 mmol, 0.357 g, 1.0 equiv) was added and the mixture was stirred overnight at room temperature under Ar atmosphere. After completion of the reaction, the reaction mixture was concentrated, extracted with ethyl acetate and the crude product was purified by flash column chromatography (using 6:4 hexane/ethyl acetate) to obtain the pure product **S3f**. **^{1}H NMR** (500 MHz, $CDCl_3$) δ 8.84 (br s, 1H, NH), 7.28 – 7.24 (m, 1H, ArH), 7.16 – 6.99 (m, 2H, ArH), 6.87 (d, *J* = 7.8 Hz, 1H, ArH), 5.53 (q, *J* = 6.6 Hz, 1H, CH-CH_3), 1.72 (d, *J* = 6.6 Hz, 3H, CH_3). **^{13}C NMR** (126 MHz, $CDCl_3$) δ 153.46, 134.85, 129.09, 123.75, 123.43, 122.57, 114.31, 75.88, 20.29. **IR** (KBr): 3162, 3102, 2990, 2934, 1600, 1495, 1453, 1431, 1397, 1261, 1073, 1046, 761 cm^{-1}. **HRMS** (ESI) calculated for $C_9H_9NO_2Na$ [M+Na]$^+$ 186.0531, found 186.0525.

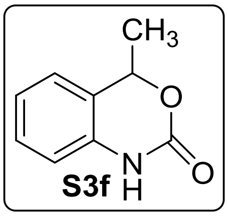

10. General experimental procedure for the synthesis of substituted 4-trifluoromethyl benzoxazinanone 1 (method D)

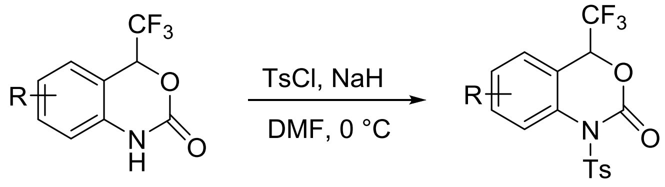

S3 1

In a flame dried 100 mL round bottom flask, compound **S3a** (3.1 mmol, 0.673 g, 1.0 equiv) was suspended in dry DMF (30 mL) and allowed to cool to 0 °C. To this solution NaH (60% dispersion in mineral oil, 4.65 mmol, 0.112 g, 1.5 equiv) was added and the mixture was allowed to stir for 1 h under Ar atmosphere. After 1 h, a solution of *p*-toluenesulfonyl chloride (3.41 mmol, 0.648 g, 1.1 equiv) in dry DMF (3 mL) was added dropwise to the reaction mixture. Completion of the reaction was monitored by TLC. After completion, the reaction mixture was poured into crushed ice followed by extraction with ethyl acetate (3×30 mL). Combined organic layers were finally washed with brine solution, dried over anhydrous Na_2SO_4 and then solvent was removed under reduced

pressure. The crude product was purified by flash column chromatography (using 8:2 hexane/ethyl acetate) to obtain the pure product **1a**. The characterization data of **1a** are summarized below.

1-Tosyl-4-(trifluoromethyl)-1,4-dihydro-2*H*-benzo[*d*][1,3]oxazin-2-one (1a)
White solid, 48% yield, mp.162.1-165.9 °C. **^{1}H NMR** (500 MHz, $CDCl_3$) δ 8.07 (d, *J* = 8.4 Hz, 2H, ArH), 7.78 (d, *J* = 8.4 Hz, 1H, ArH), 7.56 – 7.51 (m, 1H, ArH), 7.39 (d, *J* = 8.1 Hz, 2H, ArH), 7.35 – 7.30 (m, 2H, ArH), 5.42 (q, *J* = 7.0 Hz, 1H, $-CHCF_3$), 2.47 (s, 3H, CH_3). **^{13}C NMR** (126 MHz, $CDCl_3$) δ 146.65, 146.30, 134.90, 134.37, 131.03, 129.81, 129.55, 127.38, 126.34, 122.19 (q, *J* = 284.3 Hz), 121.14, 117.22, 75.91 (q, *J* = 35.3 Hz), 21.90. **^{19}F NMR** (282 MHz, $CDCl_3$) δ -77.27 (d, *J* = 7.0 Hz,3F). **IR** (KBr): 3118, 1756, 1594, 1496, 1465, 1367, 1299, 1191, 1016, 971, 958, 840, 815, 769 cm^{-1}. **HRMS** (ESI) calculated for $C_{16}H_{12}F_3NO_4SNa$ $[M+Na]^+$ 394.0337, found 394.0333.

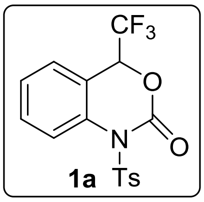

6-Methyl-1-tosyl-4-(trifluoromethyl)-1,4-dihydro-2*H*-benzo[*d*][1,3]oxazin-2-one (1b) Dirty white solid, 67% yield, mp. 144.6-148.9 °C. Using the general method D, in a flame dried 100 mL round bottom flask, compound **S3b** (2.0 mmol, 0.462 g, 1.0 equiv) was suspended in dry DMF (18 mL) and allowed to cool to 0 °C. To this solution NaH (60% dispersion in mineral oil, 3.0 mmol, 0.072 g, 1.5 equiv) was added and the mixture was allowed to stir for 1 h under Ar atmosphere. After 1 h, a solution of *p*-toluenesulfonyl chloride (2.2 mmol, 0.42 g, 1.1 equiv) in dry DMF (2 mL) was added dropwise to the reaction mixture. Completion of the reaction was monitored by TLC After completion, the reaction mixture was poured into crushed ice followed by extraction with ethyl acetate. The crude product was purified by flash column chromatography (using 6:4 hexane/ethyl acetate) to obtain the pure product **1b**. **^{1}H NMR** (500 MHz, $CDCl_3$) δ 8.05 (d, *J* = 8.4 Hz, 2H, ArH), 7.66 (d, *J* = 8.5 Hz, 1H, ArH), 7.38 (dd, *J* = 8.6, 0.6 Hz, 2H, ArH), 7.32 (dd, *J* = 8.5, 1.6 Hz, 1H, ArH), 7.10 (s, 1H, ArH), 5.36 (q, *J* = 6.8 Hz, 1H, $-CHCF_3$), 2.47 (s, 3H, CH_3), 2.39 (s, 3H, CH_3). **^{13}C NMR** (126 MHz, $CDCl_3$) δ 146.74, 146.19, 136.53, 134.97, 131.90, 131.67, 129.76, 129.53, 127.66, 122.21 (q, *J* = 284.3 Hz), 121.00, 117.05, 75.97 (q, *J* = 35.2 Hz), 21.90, 20.86. **^{19}F NMR** (282 MHz, $CDCl_3$) δ -77.30 (d, *J* = 6.8 Hz, 3F). **IR** (KBr): 2931, 1762, 1594, 1500, 1454, 1371, 1295, 1265, 1238, 1170, 1083, 970, 912, 833, 744 cm^{-1}. **HRMS** (ESI) calculated for $C_{17}H_{14}F_3NO_4SNa$ $[M+Na]^+$ 408.0493, found 408.0490.

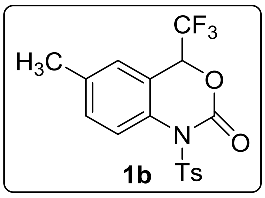

7-Fluoro-1-tosyl-4-(trifluoromethyl)-1,4-dihydro-2*H*-benzo[*d*][1,3]oxazin-2-one (1c)
White solid, 61% yield, mp. 178.4-182.6 °C. Using the general method D, in a flame dried 100 mL round bottom flask, compound **S3c** (1.5 mmol, 0.35 g, 1.0 equiv) was suspended

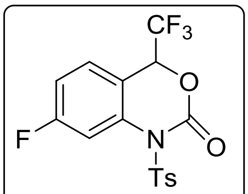
1c

in dry DMF (10 mL) and allowed to cool to 0 °C. To this solution NaH (60% dispersion in mineral oil, 2.2 mmol, 0.0536 g, 1.5 equiv) was added and the mixture was allowed to stir for 1 h under Ar atmosphere. After 1 h, a solution of *p*-toluenesulfonyl chloride (1.8 mmol, 0.342 g, 1.2 equiv) in dry DMF (2 mL) was added dropwise to the reaction mixture. Completion of the reaction was monitored by TLC After completion, the reaction mixture was poured into crushed ice followed by extraction with ethyl acetate. The crude product was purified by flash column chromatography (using 8:2 hexane/ethyl acetate) to obtain the pure product **1c**. **^{1}H NMR** (500 MHz, $CDCl_3$) δ 8.05 (d, *J* = 8.4 Hz, 2H, ArH), 7.58 (dd, *J* = 10.2, 2.4 Hz, 1H, ArH), 7.40 (d, *J* = 8.1 Hz, 2H, ArH), 7.29 (dd, *J* = 8.5, 5.6 Hz, 1H, ArH), 7.08 – 7.02 (m, 1H, ArH), 5.40 (q, *J* = 6.8 Hz, 1H, -$CHCF_3$), 2.48 (s, 3H, CH_3). **^{13}C NMR** (126 MHz, $CDCl_3$) δ 163.65 (d, *J* = 251.2 Hz), 146.64, 146.12, 135.86 (d, *J* = 11.6 Hz), 134.50, 129.92, 129.63, 128.92 (d, *J* = 9.9 Hz), 122.07 (q, *J* = 284.3 Hz), 113.64 (d, *J* = 22.6 Hz), 112.89 (d, *J* = 3.1 Hz), 109.37 (d, *J* = 28.3 Hz), 75.52 (q, *J* = 35.5 Hz), 21.95. **^{19}F NMR** (282 MHz, $CDCl_3$) δ -77.61 (d, *J* = 6.8 Hz, 3F), -106.78 – -106.92 (m, 1F). **IR** (KBr):3110, 3084, 1777, 1509, 1379, 1326, 1294, 1187, 1113, 1019, 877, 736 cm^{-1}. **HRMS** (ESI) calculated for $C_{16}H_{11}F_4NO_4SNa$ $[M+Na]^+$ 412.0228, found 412.0243.

6-Chloro-1-tosyl-4-(trifluoromethyl)-1,4-dihydro-2*H*-benzo[*d*][1,3]oxazin-2-one (1d): White powder, 60% yield, mp. 142.5-145.8 °C. Using the general method D, in a flame dried 100 mL round bottom flask, compound **S3d** (4.5 mmol, 1.13 g, 1.0 equiv) was suspended in dry DMF (40 mL) and allowed to cool to 0 °C. To this solution NaH

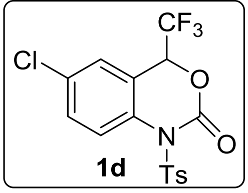
1d

(60% dispersion in mineral oil, 6.75 mmol, 0.162 g, 1.5 equiv) was added and the mixture was allowed to stir for 1 h under Ar atmosphere. After 1 h, a solution of *p*-toluenesulfonyl chloride (4.95 mmol, 0.941 g, 1.1 equiv) in dry DMF (4 mL) was added dropwise to the reaction mixture. Completion of the reaction was monitored by TLC After completion, the reaction mixture was poured into crushed ice followed by extraction with ethyl acetate. The crude product was purified by flash column chromatography (using 8:2 hexane/ethyl acetate) to obtain the pure product **1d**. **^{1}H NMR** (500 MHz, $CDCl_3$) δ 8.03 (d, *J* = 8.4 Hz, 2H, ArH), 7.75 (d, *J* = 8.9 Hz, 1H, ArH), 7.50 (dd, *J* = 8.9, 2.4 Hz, 1H, ArH), 7.39 (d, *J* = 8.1 Hz, 2H, ArH), 7.31 (d, *J* = 2.2 Hz, 1H,

ArH), 5.38 (q, J = 6.5 Hz, 1H, -CHCF$_3$), 2.47 (s, 3H, CH$_3$). **^{13}C NMR** (126 MHz, CDCl$_3$) δ 146.61, 146.12, 134.46, 132.92, 132.11, 131.16, 129.87, 129.63, 127.23, 122.61, 121.92 (q, J = 284.4 Hz), 118.81, 75.31 (q, J = 35.5 Hz), 21.92. **^{19}F NMR** (282 MHz, CDCl$_3$) δ -77.23 (d, J = 6.5 Hz, 3F). **IR** (KBr): 3122, 2969, 1764, 1594, 1486, 1428, 1369, 1290, 1267, 1172, 1085, 970, 842, 750 cm^{-1}. **HRMS** (ESI) calculated for $C_{16}H_{11}ClF_3NO_4SNa$ [M+Na]$^+$ 427.9947, found 427.9946.

6-bromo-1-tosyl-4-(trifluoromethyl)-1,4-dihydro-2*H*-benzo[*d*][1,3]oxazin-2-one(1e)

Light yellow solid, 48% yield, mp. 127.7-131.8 °C. Using the general method D, in a flame dried 50 mL round bottom flask, compound **S3e** (2.35 mmol, 0.695 g, 1.0 equiv) was suspended in dry DMF (20 mL) and allowed to cool to 0 °C. To this solution NaH (60% dispersion in mineral oil, 3.53 mmol, 0.085 g, 1.5 equiv) was added and the mixture was allowed to stir for 1 h under Ar atmosphere. After 1 h, a solution of *p*-toluenesulfonyl chloride (2.6 mmol, 0.494 g, 1.1 equiv) in dry DMF (2 mL) was added dropwise to the reaction mixture. Completion of the reaction was monitored by TLC After completion, the reaction mixture was poured into crushed ice followed by extraction with ethyl acetate. The crude product was purified by flash column chromatography (using 8:2 hexane/ethyl acetate) to obtain the pure product **1e**. **^{1}H NMR** (500 MHz, CDCl$_3$) δ 8.03 (d, J = 8.3 Hz, 2H, ArH), 7.72 – 7.63 (m, 2H, ArH), 7.45 (d, J = 1.6 Hz, 1H, ArH), 7.39 (d, J = 8.1 Hz, 2H, ArH), 5.38 (q, J = 7.6 Hz, 1H, CF$_3$-CH) , 2.47 (s, 3H, CH$_3$). **^{13}C NMR** (126 MHz, CDCl$_3$) δ 146.64, 146.10, 134.46, 134.13, 133.49, 130.15, 129.89, 129.67, 122.85, 121.95 (q, J = 284.6 Hz), 119.58, 119.08, 75.23 (q, J = 35.7 Hz), 21.96. **^{19}F NMR** (282 MHz, CDCl$_3$) δ -77.24 (d, J = 7.6 Hz, 3F). **IR** (KBr): 2987, 1764, 1594, 1486, 1427, 1367, 1292, 1186, 1085, 970, 842, 692 cm^{-1}. **HRMS** (ESI) calculated for $C_{16}H_{11}BrF_3NO_4SNa$ [M+Na]$^+$ 471.9442, found 471.9425.

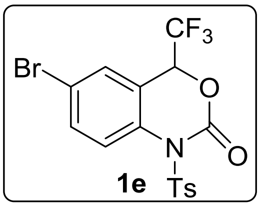

11.Typical procedure for the preparation of DDC products 3 (method E)

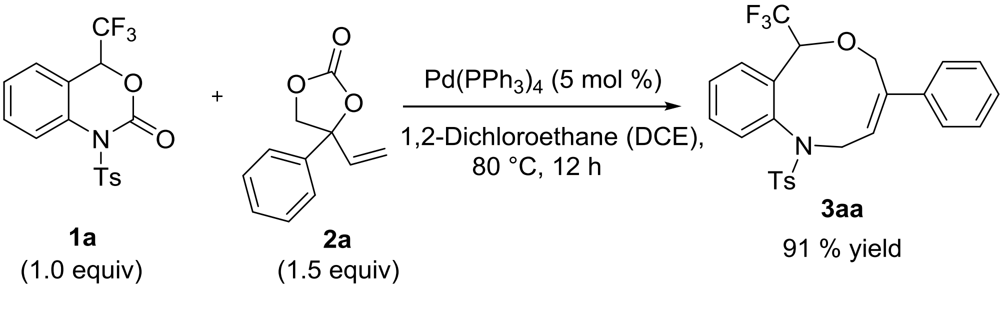

In a flame dried Schlenk tube 1 mL DCE was taken. The solvent was degassed by using

standard “freeze-pump-thaw” method. The process was repeated for three times and finally the Schlenk tube was filled with argon gas. After that, $Pd(PPh_3)_4$ (5 mol %; 0.0058 g) was taken in the schlenk tube inside the glove box and allowed to stir for few minutes. To the above mixture 1-tosyl-4-(trifluoromethyl)-1,4-dihydro-2*H*-benzo[*d*][1,3]oxazin-2-one **1a** (0.1 mmol, 0.037 g, 1.0 equiv) and 4-phenyl-4-vinyl-1,3-dioxolan-2-one **2a** (0.15 mmol, 0.029 g, 1.5 equiv) were added and the mixture was allowed to stir at 80 °C until complete conversion of **1a** (reaction time 12 h for the formation of **3aa**) under Ar atmosphere. Completion of the reaction was monitored by TLC. After completion, crude product was purified by flash column chromatography (using 8:2 hexane/ethyl acetate) to obtain the pure product **3aa** in 89% isolated yield as light yellow solid. The characterization data of **3aa** are summarized below.

12.Characterization Data of Products 3

4-Phenyl-1-tosyl-7-(trifluoromethyl)-1,2,5,7-tetrahydrobenzo[*c*][1,5]oxazonine (3aa) Light yellow solid, mp. 150.0-152.4 °C, 89% isolated yield. **^{1}H NMR** (400 MHz, $CDCl_3$) δ 7.77 (d, *J* = 7.4 Hz, 1H, ArH), 7.65 (d, *J* = 12 Hz, 2H, ArH), 7.51 – 7.48 (m, 2H, ArH), 7.47 – 7.39 (m, 2H, ArH), 7.39 – 7.31 (m, 3H, ArH), 7.29-7.26 (m, 2H, ArH), 7.00 (dd, *J* = 7.6, 1.6 Hz, 1H, ArH), 6.58 (dd, *J* = 8.4, 7.2 Hz, 1H, -CH-alkene), 5.15 (q, *J* = 6.8 Hz, 1H, CF_3-CH), 4.76 (dd, *J* = 15.2, 7.2 Hz, 1H, -N-CH-), 4.50, 3.94 (J_{AB} = 12.6, 2H, -O-CH_2-), 3.65 (dd, *J* = 15.2, 8.4 Hz, 1H, -N-CH-), 2.42 (s, 3H, CH_3). **^{13}C NMR** (126 MHz, $CDCl_3$) δ 144.21, 142.28, 140.37, 140.06, 136.61, 133.52, 131.33, 129.99, 129.95, 129.92, 129.37, 129.19, 128.64, 128.23, 127.82, 126.57, 124.42 (q, *J* = 281.40 Hz), 71.04 (q, *J* = 32.3 Hz), 65.19, 49.15, 21.69. **^{19}F NMR** (282 MHz, $CDCl_3$) δ -72.36 (d, *J* = 6.8 Hz, 3F). **IR** (KBr): 3060, 2940, 1590, 1344, 1280, 1168, 1128, 1060, 862, 765, 709 cm^{-1}. **HRMS** (ESI) calculated for $C_{25}H_{22}F_3NO_3SNa$ $[M+Na]^+$ 496.1170, found 496.1175.

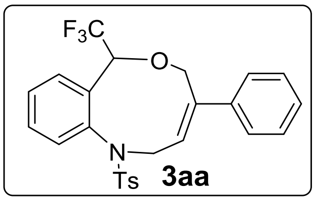

4-(p-Tolyl)-1-tosyl-7-(trifluoromethyl)-1,2,5,7-tetrahydrobenzo[*c*][1,5]oxazonine (3ab) White solid, 83% isolated yield, mp. 155.9-163.3 °C. By using method E, in DCE solution $Pd(PPh_3)_4$ (5 mol %; 0.0058 g) was taken. To it **1a** (0.1 mmol, 0.037 g, 1.0 equiv) and **2b** (0.15 mmol, 0.031 g, 1.5 equiv) were added. After being stirred at 80 °C for 12 h, the mixture was dried in vacuo. The crude product was purified by flash column chromatography (using 8:2

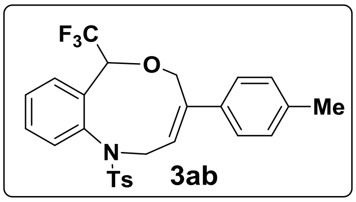

hexane/ethyl acetate) to obtain the pure product **3ab**. **^{1}H NMR** (400 MHz, $CDCl_3$) δ 7.76 (d, *J* = 7.4 Hz, 1H, ArH), 7.65 (d, *J* = 8.0 Hz, 2H, ArH), 7.47 – 7.41 (m, 2H, ArH), 7.39 (d, *J* = 8.4 Hz, 2H, ArH), 7.27 (d, *J* = 8.8 Hz, 2H, ArH), 7.16 (d, *J* = 7.9 Hz, 2H, ArH), 7.01 (dd, *J* = 7.8, 1.4 Hz, 1H, ArH), 6.56 (dd, *J* = 8.4, 7.2 Hz, 1H, -CH-alkene), 5.12 (q, *J* = 7.1 Hz, 1H, CF_3-CH), 4.75 (dd, *J* = 15.0, 7.2 Hz, 1H, -N-CH-), 4.48, 3.92 (J_{AB} = 12.6 Hz, 2H, -O-CH_2-), 3.64 (dd, *J* = 15.0, 8.4 Hz, 1H, -N-CH-), 2.42 (s, 3H, CH_3), 2.36 (s, 3H, CH_3). **^{13}C NMR** (126 MHz, $CDCl_3$) δ 144.17, 142.28, 139.87, 138.14, 137.41, 136.66, 133.53, 131.30, 129.95, 129.91, 129.45, 129.35, 129.21, 129.05, 127.82, 126.43, 127.64 – 120.91 (q, *J* = 281.93 Hz), 70.98 (q, *J* = 32.6 Hz), 65.15, 49.21, 21.70, 21.31. **^{19}F NMR** (282 MHz, $CDCl_3$) δ -72.43 (d, *J* = 7.1 Hz, 3F). **IR** (KBr): 3045, 2944, 1587, 1336, 1272, 1164, 873, 825, 723 cm^{-1}. **HRMS** (ESI) calculated for $C_{26}H_{24}F_3NO_3SNa$ $[M+Na]^+$ 510.1327, found 510.1318.

4-(4-Metoxypheny1-tosyl-7-(trifluoromethyl)-1,2,5,7-tetrahydrobenzo[*c*][1,5] oxazonine (3ac) White solid, 78% isolated yield, mp. 153.6-166.3 °C. By using method E, in DCE solution $Pd(PPh_3)_4$ (5 mol %; 0.006 g) was taken. To it **1a** (0.1 mmol, 0.037 g, 1.0 equiv) and **2c** (0.15 mmol, 0.033 g, 1.5 equiv) were added. After being stirred at 80 °C for 12 h, the mixture was dried in vacuo. The crude product was purified by flash column chromatography (using 8:2 hexane/ethyl acetate) to obtain the pure product **3ac**. **^{1}H NMR** (300 MHz, $CDCl_3$) δ 7.77 (d, *J* = 7.4 Hz, 1H, ArH), 7.65 (d, *J* = 8.3 Hz, 2H, ArH), 7.49 – 7.44 (m, 2H, ArH), 7.44 – 7.36 (m, 2H, ArH), 7.33 – 7.24 (m, 2H, ArH), 6.99 (dd, *J* = 7.7, 1.5 Hz, 1H, ArH), 6.92 – 6.85 (m, 2H, ArH), 6.54 (t, *J* = 8.0 Hz, 1H, -CH-alkene), 5.14 (q, *J* = 7.2 Hz, 1H, CF_3-CH), 4.74 (dd, *J* = 15.1, 7.3 Hz, 1H, -N-CH), 4.47, 3.90 (J_{AB} = 12.6 Hz, 2H, -O-CH_2-), 3.83 (s, 3H, OCH_3), 3.63 (dd, *J* = 15.0, 8.6 Hz, 1H, -N-CH-), 2.42 (s, 3H, CH_3). **^{13}C NMR** (126 MHz, $CDCl_3$) δ 159.71, 144.14, 142.27, 139.33, 136.66, 133.52, 132.72, 131.30, 129.94, 129.89, 129.41, 128.25, 127.79, 127.75, 126.67, 124.41 (q, *J* = 279.3 Hz), 113.96, 70.92 (q, *J* = 32.2 Hz), 65.08, 55.43, 49.27, 21.68. **^{19}F NMR** (282 MHz, $CDCl_3$) δ -72.41 (d, *J* = 7.1 Hz, 3F). **IR** (KBr): 3068, 2937, 1602, 1348, 1280, 1164, 1128, 1022, 865, 775, 701 cm^{-1}. **HRMS** (ESI) calculated for $C_{26}H_{24}F_3NO_4SNa$ $[M+Na]^+$ 526.1276, found 526.1276.

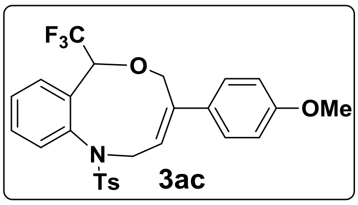

4-(4-Fluorophenyl)-1-tosyl-7-(trifluoromethyl)-1,2,5,7-tetrahydrobenzo[*c*][1,5] oxazonine (3ad) Light yellow solid, 69% isolated yield, mp. 142.5-144.6 °C. By using method E, in DCE solution $Pd(PPh_3)_4$ (5 mol %; 0.0058 g) was taken. To it **1a** (0.1 mmol,

0.037 g, 1.0 equiv) and **2d** (0.15 mmol, 0.031 g, 1.5 equiv) were added. After being stirred at 80 °C for 14 h, the mixture was dried in vacuo. The crude product was purified by flash column chromatography (using 8:2 hexane/ethyl acetate) to obtain the pure product **3ad**. **^{1}H NMR** (400 MHz, $CDCl_3$) δ 7.78 (d, *J* = 7.7 Hz, 1H, ArH), 7.65 (d, *J* = 8.4 Hz, 2H, ArH), 7.50 – 7.46 (m, 2H, ArH), 7.46 – 7.42 (m, 1H, ArH), 7.42 – 7.38 (m, 1H, ArH), 7.28 (d, *J* = 7.9 Hz, 2H, ArH), 7.07 – 7.00 (m, 2H, ArH), 6.97 (dd, *J* = 7.9, 1.3 Hz, 1H, ArH), 6.55 (dd, *J* = 8.4, 7.4 Hz, 1H, -CH-alkene), 5.15 (q, *J* = 7.1 Hz, 1H, CF_3-CH), 4.74 (dd, *J* = 15.2, 7.4 Hz, 1H, - N-CH-), 4.45, 3.91 (J_{AB} = 12.8 Hz, 2H, -O-CH_2-), 3.63 (dd, *J* = 15.2, 8.4 Hz, 1H, -N-CH-), 2.43 (s, 3H, CH_3). **^{13}C NMR** (126 MHz, $CDCl_3$) δ 162.87 (d, *J* = 247.6 Hz), 144.26, 142.26, 139.05, 136.48 (d, *J* = 18.0 Hz), 136.44., 133.45, 131.39, 130.02 (d, *J* = 9.5 Hz), 129.93, 129.31, 129.22 (d, *J* = 2.3 Hz), 128.34, 128.28, 127.82, 124.41 (q, *J* = 277.1 Hz), 115.53 (d, *J* = 21.4 Hz), 71.03 (q, *J* = 32.7 Hz), 65.11, 49.12, 21.70. **^{19}F NMR** (282 MHz, $CDCl_3$) δ -72.43 (d, *J* = 7.1 Hz, 3F), -113.86 – -115.46 (m, 1F). **IR** (KBr): 3060, 2944, 1594, 1355, 1160, 877, 765, 730 cm^{-1}. **HRMS** (ESI) calculated for $C_{25}H_{21}F_4NO_3SNa$ $[M+Na]^+$ 514.1076, found 514.1080.

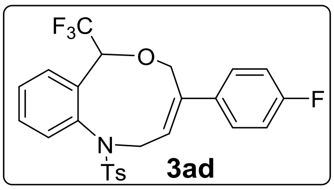

4-(4-Chlorophenyl)-1-tosyl-7-(trifluoromethyl)-1,2,5,7-tetrahydrobenzo[*c*][1,5] oxazonine (3ae) Light yellow solid, 86% isolated yield, mp. 125.4-130.9 °C. By using method E, in DCE solution $Pd(PPh_3)_4$ (5 mol %; 0.0058 g) was taken. To it **1a** (0.1 mmol, 0.037 g, 1.0 equiv) and **2e** (0.15 mmol, 0.034 g, 1.5 equiv) were added. After being stirred at 80 °C for 12 h, the mixture was dried in vacuo. The crude product was purified by flash column chromatography (using 8:2 hexane/ethyl acetate) to obtain the pure product **3ae**. **^{1}H NMR** (400 MHz, $CDCl_3$) δ 7.77 (d, *J* = 7.6 Hz, 1H, ArH), 7.65 (d, *J* = 8.3 Hz, 2H, ArH), 7.48 – 7.43 (m, 2H, ArH), 7.43 – 7.37 (m, 2H, ArH), 7.34 – 7.30 (m, 2H, ArH), 7.31 – 7.28 (m, 1H, ArH), 7.29 – 7.27 (m, 1H, ArH), 6.96 (dd, *J* = 7.9, 1.3 Hz, 1H, ArH), 6.58 (dd, *J* = 8.4, 7.4 Hz, 1H, -CH-alkene), 5.14 (q, *J* = 6.8 Hz, 1H, CF_3-CH), 4.74 (dd, *J* = 15.0, 7.4 Hz, 1H, - N-CH-), 4.45, 3.91 (J_{AB} = 12.8 Hz, 2H, -O-CH_2-), 3.63 (dd, *J* = 15.0, 8.4 Hz, 1H, -N-CH-), 2.43 (s, 3H, CH_3). **^{13}C NMR** (126 MHz, $CDCl_3$) δ 144.30, 142.23, 138.94, 138.74, 136.49, 134.23, 133.42, 131.42, 130.47, 130.08, 129.94, 129.27, 129.21, 128.80, 127.88, 127.81, 124.37 (q, *J* = 281.2 Hz), 71.06 (q, *J* = 32.4 Hz), 64.94, 49.06, 21.70. **^{19}F NMR** (282 MHz, $CDCl_3$) δ -72.41 (d, *J* = 6.8 Hz, 3F). **IR** (KBr): 3056, 2956, 1587, 1344, 1276, 1157, 1097, 1068, 873, 765, 719 cm^{-1}. **HRMS** (ESI) calculated for $C_{25}H_{21}ClF_3NO_3SNa$ $[M+Na]^+$

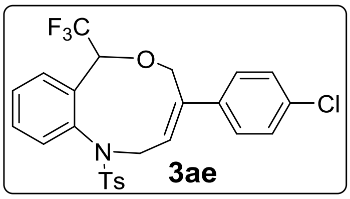

530.0780, found 530.0780.

4-(4-Bromophenyl)-1-tosyl-7-(trifluoromethyl)-1,2,5,7-tetrahydrobenzo[*c*][1,5] oxazonine (3af) White solid, 91% isolated yield, mp. 153.3-154.6 °C. By using method E, in DCE solution Pd(PPh_3)$_4$ (5 mol %; 0.0058 g) was taken. To it **1a** (0.1 mmol, 0.037 g, 1.0 equiv) and **2f** (0.15 mmol, 0.0404 g, 1.5 equiv) were added. After being stirred at 80 °C for 12 h, the mixture was dried in vacuo. The crude product was purified by flash column chromatography (using 8:2 hexane/ethyl acetate) to obtain the pure product **3af**. **^{1}H NMR** (400 MHz, $CDCl_3$) δ 7.77 (d, *J* = 7.6 Hz, 1H, ArH), 7.65 (d, *J* = 8.4 Hz, 2H, ArH), 7.49 – 7.46 (m, 2H, ArH), 7.46 – 7.39 (m, 2H, ArH), 7.38 – 7.36 (m, 2H, ArH), 7.29 – 7.26 (m, 2H, ArH), 6.95 (dd, *J* = 7.9, 1.3 Hz, 1H, ArH), 6.58 (dd, *J* = 8.4, 7.4 Hz, 1H, -CH-alkene), 5.14 (q, *J* = 7.1 Hz, 1H, CF_3-CH), 4.73 (dd, *J* = 15.0, 7.4 Hz, 1H, -N-CH-), 4.44, 3.91 (J_{AB} = 12.8 Hz, 2H, -O-CH_2-), 3.63 (dd, *J* = 15.0, 8.4 Hz, 1H, -N-CH-), 2.43 (s, 3H, CH_3). **^{13}C NMR** (126 MHz, $CDCl_3$) δ 144.30, 142.22, 139.20, 138.99, 136.47, 133.40, 131.76, 131.42, 130.52, 130.08, 129.94, 129.25, 129.18, 128.18, 127.80, 124.36 (q, *J* = 281.4 Hz), 122.46, 71.07 (q, *J* = 32.5 Hz), 64.90, 49.05, 21.70. **^{19}F NMR** (282 MHz, $CDCl_3$) δ -72.40 (d, *J* = 7.1 Hz, 3F). **IR** (KBr): 3052, 2952, 1590, 1344, 1276, 1157, 1086, 1068, 877, 723 cm^{-1}. **HRMS** (ESI) calculated for $C_{25}H_{21}BrF_3NO_3SNa$ $[M+Na]^+$ 574.0275, found 574.0292.

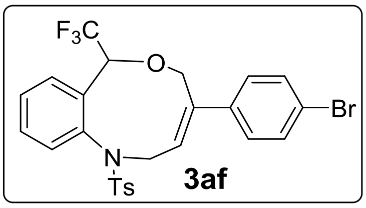

1-Tosyl-7-(trifluoromethyl)-4-(4-(trifluoromethyl)phenyl)-1,2,5,7-tetrahydrobenzo [*c*][1,5]oxazonine (3ag) Light yellow solid, 56% isolated yield, mp. 142.0-151.7 °C. By using method E, in DCE solution Pd(PPh_3)$_4$ (5 mol %; 0.0058 g) was taken. To it **1a** (0.1 mmol, 0.037 g, 1.0 equiv) and **2g** (0.15 mmol, 0.0387 g, 1.5 equiv) were added. After being stirred at 80 °C for 12 h, the mixture was dried in vacuo. The crude product was purified by flash column chromatography (using 8:2 hexane/ethyl acetate) to obtain the pure product **3ag**. **^{1}H NMR** (400 MHz, $CDCl_3$) δ 7.79 (d, *J* = 7.7 Hz, 1H, ArH), 7.66 (d, *J* = 8.4 Hz, 2H, ArH), 7.65 – 7.61 (m, 4H, ArH), 7.49 – 7.38 (m, 2H, ArH), 7.30 – 7.26 (m, 2H, ArH), 6.96 (dd, *J* = 7.9, 1.3 Hz, 1H, ArH), 6.65 (dd, *J* = 8.4, 7.2 Hz, 1H, -CH-alkene), 5.18 (q, *J* = 7.1 Hz, 1H, CF_3-CH), 4.76 (dd, *J* = 15.2, 7.2 Hz, 1H, -N-CH-), 4.48, 3.95 (J_{AB} = 12.6 Hz, 2H, -O-CH_2-), 3.66 (dd, *J* = 15.2, 8.4 Hz, 1H,-N-CH-), 2.43 (s, 3H, CH_3). **^{13}C NMR** (126 MHz, $CDCl_3$) δ 144.38, 143.85, 142.23, 139.00, 136.39, 133.40, 131.94, 131.48, 130.34, 130.16, 130.08, 129.98, 129.22, 127.84, 126.90, 125.63 (q, *J* = 4.1 Hz), 124.36 (q, *J* = 281.2 Hz),

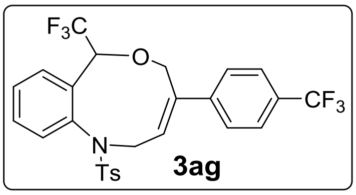

124.25(q, *J* = 272.7 Hz), 71.19 (q, *J* = 32.4 Hz), 64.94, 48.97, 21.71. **^{19}F NMR** (282 MHz, $CDCl_3$) δ -63.07 (s, 3F), -72.37 (d, *J* = 7.1 Hz, 3F). **IR** (KBr): 2960, 1542, 1332, 1276, 1157, 1120, 1060, 873, 738, 715 cm^{-1}. **HRMS** (ESI) calculated for $C_{26}H_{21}F_6NO_3SNa$ $[M+Na]^+$ 564.1044, found 564.1059.

4-(2-Fluorophenyl)-1-tosyl-7-(trifluoromethyl)-1,2,5,7-tetrahydrobenzo[*c*][1,5] oxazonine (3ah) White solid, 84% isolated yield, mp. 166.1-168.0 °C. By using method E, in DCE solution $Pd(PPh_3)_4$ (5 mol %; 0.0058 g) was taken. To it **1a** (0.1 mmol, 0.037 g, 1.0 equiv) and **2h** (0.15 mmol, 0.031 g, 1.5 equiv) were added. After being stirred at 80 °C for 13 h, the mixture was dried in vacuo. The crude product was purified by flash column chromatography (using 8:2 hexane/ethyl acetate) to obtain the pure product **3ah**. **^{1}H NMR** (400 MHz, $CDCl_3$) δ 7.75 (d, *J* = 7.4 Hz, 1H, ArH), 7.69 (d, *J* = 8.3 Hz, 2H, ArH), 7.47 – 7.39 (m, 2H, ArH), 7.34 – 7.29 (m, 2H, ArH), 7.29 – 7.24 (m, 1H, ArH), 7.21 – 7.17 (m, 1H, ArH), 7.13 – 7.08 (m, 1H, ArH), 7.07 – 7.02 (m, 1H, ArH), 7.01 – 6.98 (m, 1H, ArH), 6.40 (br t, *J* = 7.8 Hz, 1H, -CH-alkene), 5.20 (q, *J* = 7.1 Hz, 1H, CF_3-CH), 4.71 (dd, *J* = 15.0, 7.2 Hz, 1H, -N-CH-), 4.47, 3.94 (J_{AB} = 12.8 Hz, 2H, -O-CH_2-), 3.72 (dd, *J* = 15.0, 8.4 Hz, 1H, -N-CH-), 2.45 (s, 3H, CH_3). **^{13}C NMR** (126 MHz, $CDCl_3$) δ 159.74 (d, *J* = 247.7 Hz), 144.29, 142.25, 136.53, 136.26, 133.45 (d, *J* = 3.3 Hz), 131.35, 130.57 (d, *J* = 3.4 Hz), 130.00, 129.97, 129.73 (d, *J* = 8.3 Hz), 129.70, 129.34, 129.11, 128.31 (d, *J* = 13.5 Hz), 127.84, 124.41 (d, *J* = 3.6 Hz), 124.40 (q, *J* = 281.7 Hz), 115.85 (d, *J* = 22.6 Hz), 71.47 (q, *J* = 32.3 Hz), 65.70, 48.71, 21.70. **^{19}F NMR** (282 MHz, $CDCl_3$) δ -72.32 (d, *J* = 7.1 Hz, 3F), -116.65 (d, *J* = 4.5 Hz, 1F). **IR** (KBr): 3060, 2948, 1587, 1340, 1284, 1160, 873, 761, 715 cm^{-1}. **HRMS** (ESI) calculated for $C_{25}H_{21}F_4NO_3SNa$ $[M+Na]^+$ 514.1076, found 514.1082.

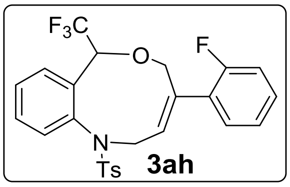

4-(2-Methoxyphenyl)-1-tosyl-7-(trifluoromethyl)-1,2,5,7-tetrahydrobenzo[*c*][1,5] oxazonine (3ai) White solid, 88% isolated yield, mp. 197.3-199.4 °C. By using method E, in DCE solution $Pd(PPh_3)_4$ (5 mol %; 0.0058 g) was taken. To it **1a** (0.1 mmol, 0.037 g, 1.0 equiv) and **2i** (0.15 mmol, 0.033 g, 1.5 equiv) were added. After being stirred at 80 °C for 13 h, the mixture was dried in vacuo. The crude product was purified by flash column chromatography (using 8:2 hexane/ethyl acetate) to obtain the pure product **3ai**. **^{1}H NMR** (400 MHz, $CDCl_3$) δ 7.73 (d, *J* = 1.0 Hz, 1H, ArH), 7.71 (d, *J* = 8.3 Hz, 2H, ArH), 7.47 – 7.43 (m, 1H, ArH), 7.43 – 7.39 (m, 1H, ArH), 7.33 (dd, *J* = 8.5, 0.6 Hz, 2H, ArH), 7.30 – 7.26 (m, 1H, ArH), 7.05 – 7.02 (m, 1H,

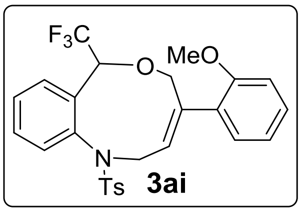

ArH), 6.91 – 6.87 (m, 1H, ArH), 6.84 (dd, J = 8.3, 0.7 Hz, 1H, ArH), 6.80 (dd, J = 7.4, 1.8 Hz, 1H, ArH), 6.17 (dd, J = 8.4, 7.2 Hz, 1H, -CH-alkene), 5.16 (q, J = 7.1 Hz, 1H, CF_3-CH), 4.68 (dd, J = 15.2, 7.2 Hz, 1H, -N-CH-), 4.58, 3.95 (J_{AB} = 12.8 Hz, 2H, -O-CH_2-), 3.76 (s, 3H, OCH_3), 3.79 – 3.72 (dd, J = 15.2, 8.4 Hz, 1H, -N-CH-), 2.46 (s, 3H, CH_3). **^{13}C NMR** (126 MHz, $CDCl_3$) δ 156.34, 144.15, 142.27, 140.64, 136.87, 133.66, 131.39, 131.18, 130.69, 129.96, 129.83, 129.61, 129.60, 129.44, 129.07, 127.83, 124.40 (q, J = 271.4 Hz), 120.78, 110.52, 71.59 (q, J = 32.1 Hz), 65.28, 55.31, 48.70, 21.71. **^{19}F NMR** (282 MHz, $CDCl_3$) δ -72.27 (d, J = 7.1 Hz, 3F). **IR** (KBr): 3048, 2937, 1606, 1359, 1272, 1172, 1128, 1064, 881, 782, 746, 646 cm^{-1}. **HRMS** (ESI) calculated for $C_{26}H_{24}F_3NO_4SNa$ [M+Na]$^+$ 526.1276, found 526.1290.

4-(Furan-2-yl)-1-tosyl-7-(trifluoromethyl)-1,2,5,7-tetrahydrobenzo[*c*][1,5] oxazonine (3aj) White solid, 76% isolated yield, mp. 97.6-106.8 °C. By using method E, in DCE solution $Pd(PPh_3)_4$ (5 mol %; 0.0058 g) was taken. To it **1a** (0.1 mmol, 0.037 g, 1.0 equiv) and **2j** (0.2 mmol, 0.036 g, 2.0 equiv) were added. After being stirred at 80 °C for 16 h, the mixture was dried in vacuo. The crude product was purified by flash column chromatography (using 8:2 hexane/ethyl acetate) to obtain the pure product **3aj**. **^{1}H NMR** (400 MHz, $CDCl_3$) δ 7.74 (d, J = 7.6 Hz, 1H, ArH), 7.66 (d, J = 8.3 Hz, 2H, ArH), 7.46 – 7.42 (m, 1H, ArH), 7.40 – 7.35 (m, 2H, ArH), 7.29 – 7.26 (m, 2H, ArH), 6.93 (dd, J = 7.8, 1.6 Hz, 1H, furyl-H), 6.77 (dd, J = 8.6, 7.4 Hz, 1H, -CH-alkene), 6.52 (br d, J = 3.6 Hz, 1H, furyl-H), 6.42 (dd, J = 3.4, 1.6 Hz, 1H, furyl-H), 5.09 (q, J = 7.2 Hz, 1H, CF_3-CH), 4.77 (dd, J = 15.2, 7.4 Hz, 1H, -N-CH-), 4.47, 3.84 (J_{AB} = 12.8 Hz, 2H, -O-CH_2-), 3.66 (dd, J = 15.2, 8.6 Hz, 1H, -N-CH-), 2.42 (s, 3H,CH_3).

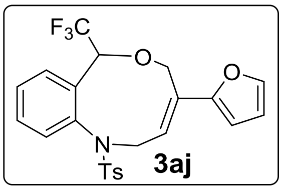

^{13}C NMR (126 MHz, $CDCl_3$) δ 152.83, 144.20, 142.80, 142.29, 136.51, 133.57, 131.33, 130.05, 130.02, 129.90, 129.38, 129.14, 127.85, 125.48, 124.29 (q, J = 281.5 Hz), 111.76, 108.59, 71.44 (q, J = 31.9 Hz), 62.97, 48.92, 21.69. **^{19}F NMR** (282 MHz, $CDCl_3$) δ -72.48 (d, J = 7.2 Hz, 3F). **IR** (KBr): 3100, 2948, 1594, 1348, 1276, 1168, 869, 769, 715, 651 cm^{-1}. **HRMS** (ESI) calculated for $C_{23}H_{20}F_3NO_4SNa$ [M+Na]$^+$486.0963, found 486.0970.

4-(Thiophen-2-yl)-1-tosyl-7-(trifluoromethyl)-1,2,5,7-tetrahydrobenzo[*c*][1,5] oxazonine (3ak) Light yellow solid, 79% isolated yield, mp. 167.3-175.6 °C. By using method E, in DCE solution $Pd(PPh_3)_4$ (5 mol %; 0.0058 g) was taken. To it **1a** (0.1 mmol, 0.037 g, 1.0 equiv) and **2k** (0.2 mmol, 0.0392 g, 2.0 equiv) were added. After being stirred at 80 °C for 16 h, the mixture was dried in vacuo. The crude product was purified by flash

column chromatography (using 8:2 hexane/ethyl acetate) to obtain the pure product **3ak**.

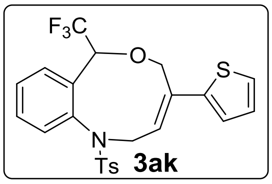

^{1}H NMR (400 MHz, $CDCl_3$) δ 7.76 (d, J = 7.4 Hz, 1H, ArH), 7.65 (d, J = 8.3 Hz, 2H, ArH), 7.48 – 7.42 (m, 1H, ArH), 7.42 – 7.38 (m, 1H, ArH), 7.28 (br d, J = 0.5 Hz, 1H, thienyl-H), 7.26 (d, J = 1.0 Hz, 1H, ArH), 7.24 – 7.21 (m, 2H, ArH), 7.02 (dd, J = 5.2, 3.6 Hz, 1H, thienyl-H), 6.99 (dd, J = 7.8, 1.4 Hz, 1H, thienyl-H), 6.63 (dd, J = 8.6, 7.6 Hz, 1H, -CH-alkene), 5.07 (q, J = 7.1 Hz, 1H, CF_3-CH), 4.74 (dd, J = 15.2, 7.6 Hz, 1H, -N-CH-), 4.51, 3.90 (J_{AB} = 12.8 Hz, 2H, -O-CH_2-), 3.64 (dd, J = 15.2, 8.6 Hz, 1H, -N-CH-), 2.42 (s, 3H, CH_3). **^{13}C NMR** (126 MHz, $CDCl_3$) δ 144.24, 143.27, 142.25, 136.53, 134.35, 133.39, 131.40, 130.07, 129.94, 129.53, 129.20, 129.18, 127.92, 127.80, 125.67, 125.35, 122.05 (q, J = 280.0 Hz), 71.24 (q, J = 32.6 Hz), 64.67, 49.17, 21.69. **^{19}F NMR** (282 MHz, $CDCl_3$) δ -72.38 (d, J = 7.1 Hz, 3F). **IR** (KBr): 3031, 2964, 2908, 1733, 1631, 1598, 1392, 1336, 1268, 1153, 1132, 1068, 865, 765, 719 cm^{-1}. **HRMS** (ESI) calculated for $C_{23}H_{20}F_3NO_3S_2Na$ $[M+Na]^+$ 502.0734, found 502.0737.

4-Cyclohexyl-1-tosyl-7-(trifluoromethyl)-1,2,5,7-tetrahydrobenzo[*c*][1,5]oxazonine (3al) White solid, 53% isolated yield, mp. 125.3-130.1 °C. By using method E, in DCE solution $Pd(PPh_3)_4$ (5 mol %; 0.0058 g) was taken. To it **1a** (0.1 mmol, 0.037 g, 1.0 equiv) and **2l** (0.15 mmol, 0.0294 g, 1.5 equiv) were added. After being stirred at 80 °C for 16 h, the mixture was dried in vacuo. The crude product was purified by flash column chromatography (using 8:2 hexane/ethyl acetate) to obtain the pure product **3al**. **^{1}H NMR**

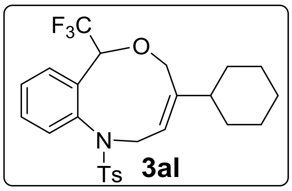

(300 MHz, $CDCl_3$) δ 7.74 – 7.68 (m, 1H, ArH), 7.65 (d, J = 8.3 Hz, 2H, ArH), 7.46 – 7.36 (m, 2H, ArH), 7.29 (d, J = 8.2 Hz, 2H, ArH), 7.07 – 7.00 (m, 1H, ArH), 5.97 (br t, J = 7.9 Hz, 1H, -CH-alkene), 4.89 (q, J = 7.2 Hz, 1H, CF_3-CH), 4.58 (dd, J = 15.0, 7.3 Hz, 1H, -N-CH-), 4.07, 3.59 (J_{AB} = 12.4 Hz, 2H, -O-CH_2-), 3.56 – 3.47 (m, 1H, -N-CH-), 2.43 (s, 3H, CH_3), 2.08 – 1.95 (m, 1H, cyclohex-H), 1.83 – 1.74 (m, 2H, cyclohex-H), 1.72 – 1.59 (m, 3H, cyclohex-H), 1.36 – 1.02 (m, 5H, cyclohex-H). **^{13}C NMR** (126 MHz, $CDCl_3$) δ 146.66, 144.05, 142.29, 136.93, 133.45, 131.17, 129.86, 129.77, 129.70, 128.96, 127.77, 126.04, 124.50 (q, J = 281.6 Hz), 71.08 (q, J = 31.9 Hz), 64.62, 48.74, 44.33, 32.62, 32.10, 26.82, 26.72, 26.23, 21.67. **^{19}F NMR** (282 MHz, $CDCl_3$) δ -72.17 (d, J = 7.1 Hz, 3F). **IR** (KBr): 3108, 2929, 2852, 1598, 1392, 1351, 1268, 1160, 1056, 869, 719 cm^{-1}. **HRMS** (ESI) calculated for $C_{25}H_{28}F_3NO_3SNa$ $[M+Na]^+$ 502.1640, found 502.1638.

4-(Naphthalen-2-yl)-1-tosyl-7-(trifluoromethyl)-1,2,5,7-tetrahydrobenzo[*c*][1,5] oxazonine (3am) White solid, 65% isolated yield, mp. 128.9-136.2 °C. By using method E, in DCE solution Pd(PPh_3)$_4$ (5 mol %; 0.0058 g) was taken. To it **1a** (0.1 mmol, 0.037 g, 1.0 equiv) and **2m** (0.15 mmol, 0.036 g, 1.5 equiv) were added. After being stirred at 80 °C for 15 h, the mixture was dried in vacuo. The crude product was purified by flash column chromatography (using 8:2 hexane/ethyl acetate) to obtain the pure product **3am**. **^{1}H NMR** (400 MHz, $CDCl_3$) δ 7.98 (d, J = 1.3 Hz, 1H, ArH), 7.88 – 7.83 (m, 2H, ArH), 7.83 – 7.79 (m, 2H, ArH), 7.69 – 7.63 (m, 2H, ArH), 7.61 (dd, J = 8.6, 1.9 Hz, 1H, ArH), 7.50 – 7.47 (m, 3H, ArH), 7.45 – 7.38 (m, 2H, ArH), 7.28 (d, J = 0.5 Hz, 1H, ArH), 7.05 (dd, J = 7.8, 1.5 Hz, 1H, ArH), 6.73 (dd, J = 8.6, 7.2 Hz, 1H, -CH-alkene), 5.18 (q, J = 7.9 Hz, 1H, CF_3-CH), 4.82 (dd, J = 15.2, 7.2 Hz, 1H, -N-CH-), 4.63, 4.03 (J_{AB} =12.6 Hz, 2H, -O-CH_2-), 3.72 (dd, J = 15.2, 8.6 Hz, 1H, -N-CH-), 2.41 (s, 3H, CH_3). **^{13}C NMR** (126 MHz, $CDCl_3$) δ 144.23, 142.28, 139.95, 137.47, 136.59, 133.50, 133.46, 133.11, 131.37, 130.28, 130.02, 129.94, 129.49, 129.23, 128.59, 128.24, 127.81, 127.67, 126.44, 126.41, 125.86, 124.37, 124.36 (q, J = 276.0 Hz), 71.11 (q, J = 32.3 Hz), 65.15, 49.22, 21.69. **^{19}F NMR** (282 MHz, $CDCl_3$) δ -72.35 (d, J = 7.9 Hz, 3F). **IR** (KBr): 3060, 2948, 1598, 1351, 1265, 1160, 1089, 1033, 869, 786, 734, 694 cm^{-1}. **HRMS** (ESI) calculated for $C_{29}H_{24}F_3NO_3SNa$ $[M+Na]^+$ 546.1327, found 546.1327.

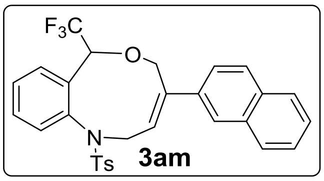

9-Methyl-4-phenyl-1-tosyl-7-(trifluoromethyl)-1,2,5,7-tetrahydrobenzo[*c*][1,5] oxazonine (3ba) White solid, 81% isolated yield, mp. 153.0-156.9 °C. By using method E, in DCE solution Pd(PPh_3)$_4$ (5 mol %; 0.0058 g) was taken. To it **1b** (0.1 mmol, 0.0385 g, 1.0 equiv) and **2a** (0.15 mmol, 0.029 g, 1.5 equiv) were added. After being stirred at 80 °C for 12 h, the mixture was dried in vacuo. The crude product was purified by flash column chromatography (using 8:2 hexane/ethyl acetate) to obtain the pure product **3ba**. **^{1}H NMR** (400 MHz, $CDCl_3$) δ 7.65 (d, J = 8.0 Hz, 2H, ArH), 7.55 (s, 1H, ArH), 7.53 – 7.48 (m, 2H, ArH), 7.39 – 7.34 (m, 2H, ArH), 7.34 – 7.30 (m, 1H, ArH), 7.28 (d, J = 0.6 Hz, 2H, ArH), 7.22 – 7.17 (m, 1H, ArH), 6.87 (d, J = 8.1 Hz, 1H, ArH), 6.61 (dd, J = 8.4, 7.4 Hz, 1H, -CH-alkene), 5.10 (q, J = 7.1 Hz, 1H, CF_3-CH), 4.74 (dd, J = 15.0, 7.2 Hz, 1H, -N-CH-), 4.48, 3.92 (J_{AB} = 12.6 Hz, 2H, -O-CH_2-), 3.62 (dd, J = 15.0, 8.4 Hz, 1H, -N-CH-), 2.42 (s, 3H, CH_3), 2.39 (s, 3H, CH_3). **^{13}C NMR** (126 MHz, $CDCl_3$) δ 144.08, 140.42, 140.20, 139.88, 139.75, 136.75, 133.01, 132.14, 130.29, 129.87, 129.54, 128.99, 128.62, 128.18, 127.79, 126.56, 124.45 (q, J = 280.3 Hz), 70.88 (q, J = 32.6 Hz),

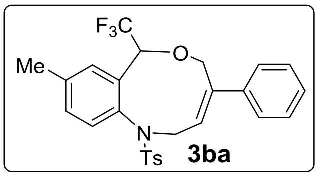

65.01, 49.25, 21.67, 21.53. **^{19}F NMR** (282 MHz, $CDCl_3$) δ -72.25 (d, *J* = 7.1 Hz, 3F). **IR** (KBr): 3083, 3052, 2933, 1590, 1336, 1276, 1157, 1060, 962, 873, 769, 686 cm^{-1}. **HRMS** (ESI) calculated for $C_{26}H_{24}F_3NO_3SNa$ $[M+Na]^+$ 510.1327, found 510.1330.

9-Methyl-4-(p-tolyl)-1-tosyl-7-(trifluoromethyl)-1,2,5,7-tetrahydrobenzo[*c*][1,5] oxazonine (3bb) White solid, 84% isolated yield, mp. 181.9-187.2 °C. By using method E, in DCE solution $Pd(PPh_3)_4$ (5 mol %; 0.0058 g) was taken. To it **1b** (0.1 mmol, 0.0385 g, 1.0 equiv) and **2b** (0.15 mmol, 0.031 g, 1.5 equiv) were added. After being stirred at 80 °C for 12 h, the mixture was dried in vacuo. The crude product was purified by flash column chromatography (using 8:2 hexane/ethyl acetate) to obtain the pure product **3bb**. **^{1}H NMR** (400 MHz, $CDCl_3$) δ 7.65 (d, *J* = 8.4 Hz, 2H, ArH), 7.54 (s, 1H, ArH), 7.43 – 7.38 (m, 2H, ArH), 7.24 (dd, *J* = 7.5, 1.1 Hz, 2H, ArH), 7.21 – 7.18 (m, 1H, ArH), 7.16 (d, *J* = 7.9 Hz, 2H, ArH), 6.87 (d, *J* = 8.1 Hz, 1H, ArH), 6.58 (dd, *J* = 8.4, 7.4 Hz, 1H, -CH-alkene), 5.08 (q, *J* = 7.1 Hz, 1H, CF_3-CH), 4.73 (dd, *J* = 15.2, 7.4 Hz, 1H, -N-CH-), 4.46, 3.90 (J_{AB} = 12.4 Hz, 2H, -O-CH_2-), 3.60 (dd, *J* = 15.2, 8.4 Hz, 1H, -N-CH-), 2.42 (s, 3H, CH_3), 2.38 (s, 3H, CH_3), 2.36 (s, 3H, CH_3). **^{13}C NMR** (126 MHz, $CDCl_3$) δ 144.04, 140.15, 139.75, 139.68, 138.07, 137.46, 136.79, 133.02, 132.11, 129.85, 129.54, 129.38, 129.32, 129.04, 127.78, 126.42, 124.46 (q, *J* = 282.9 Hz), 70.83 (q, *J* = 32.2 Hz), 64.98, 49.30, 21.67, 21.53, 21.29. **^{19}F NMR** (282 MHz, $CDCl_3$) δ -72.32 (d, *J* = 7.1 Hz, 3F). **IR** (KBr): 3072, 3052, 2929, 1594, 1388, 1344, 1280, 1149, 1068, 877, 809, 701 cm^{-1}. **HRMS** (ESI) calculated for $C_{27}H_{26}F_3NO_3SNa$ $[M+Na]^+$ 524.1483, found 524.1496.

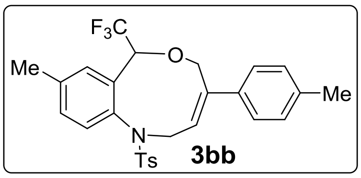

3bb

4-(4-Methoxyphenyl)-9-methyl-1-tosyl-7-(trifluoromethyl)-1,2,5,7-tetrahydrobenzo [*c*][1,5]oxazonine (3bc) Light yellow solid, 81% isolated yield, mp. 165.2-170.6 °C. By using method E, in DCE solution $Pd(PPh_3)_4$ (5 mol %; 0.0058 g) was taken. To it **1b** (0.1 mmol, 0.0385 g, 1.0 equiv) and **2c** (0.15 mmol, 0.033 g, 1.5 equiv) were added. After being stirred at 80 °C for 12 h, the mixture was dried in vacuo. The crude product was purified by flash column chromatography (using 8:2 hexane/ethyl acetate) to obtain the pure product **3bc**. **^{1}H NMR** (400 MHz, $CDCl_3$) δ 7.65 (d, *J* = 8.4 Hz, 2H, ArH), 7.54 (s, 1H, ArH), 7.49 – 7.43 (m, 2H, ArH), 7.28 – 7.25 (m, 2H, ArH), 7.20 – 7.18 (m, 1H, ArH), 6.91 – 6.87 (m, 3H, ArH), 6.55 (dd, *J* = 8.4, 7.6 Hz, 1H, -CH-alkene), 5.08 (q, *J* = 7.1 Hz, 1H, CF_3-CH), 4.73 (dd, *J* = 15.0, 7.6 Hz, 1H, -N-CH-), 4.45, 3.89 (J_{AB} = 12.4 Hz, 2H,- O-CH_2-), 3.83 (s, 3H, OCH_3), 3.59 (dd, *J* = 15.0,

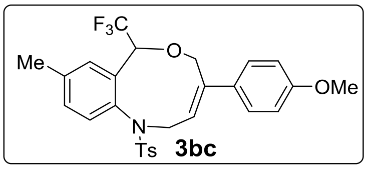

3bc

8.4 Hz, 1H, -N-CH-), 2.42 (s, 3H, CH_3), 2.39 (s, 3H, CH_3). **^{13}C NMR** (126 MHz, $CDCl_3$) δ 159.70, 144.03, 140.16, 139.76, 139.19, 136.82, 133.02, 132.80, 132.77, 132.11, 129.85, 129.54, 129.07, 128.57, 127.77, 123.34 (q, J = 271.7 Hz), 113.97, 70.77 (q, J = 32.7 Hz), 64.94, 55.44, 49.38, 21.65, 21.54. **^{19}F NMR** (282 MHz, $CDCl_3$) δ -72.32 (d, J = 7.1 Hz, 3F). **IR** (KBr): 3031, 2929, 1602, 1344, 1280, 1247, 1105, 1060, 917, 873, 817, 671 cm^{-1}. **HRMS** (ESI) calculated for $C_{27}H_{26}F_3NO_4SNa$ $[M+Na]^+$ 540.1432, found 540.1436.

4-(4-Chlorophenyl)-9-methyl-1-tosyl-7-(trifluoromethyl)-1,2,5,7-tetrahydrobenzo [*c*][1,5]oxazonine (3be) Light yellow solid, 79% isolated yield, mp. 205.6-212.5 °C. By using method E, in DCE solution $Pd(PPh_3)_4$ (5 mol %; 0.006 g) was taken. To it **1b** (0.1 mmol, 0.0385 g, 1.0 equiv) and **2e** (0.15 mmol, 0.034 g, 1.5 equiv) were added. After being stirred at 80 °C for 12 h, the mixture was dried in vacuo. The crude product was purified by flash column chromatography (using 8:2 hexane/ethyl acetate) to obtain the pure product **3be**. **^{1}H NMR** (400 MHz, $CDCl_3$) δ 7.64 (d, J = 8.4 Hz, 2H, ArH), 7.55 (s, 1H, ArH), 7.48 – 7.43 (m, 2H, ArH), 7.35 – 7.30 (m, 2H, ArH), 7.30 – 7.25 (m, 2H, ArH), 7.21 – 7.18 (m, 1H, ArH), 6.83 (d, J = 8.1 Hz, 1H, ArH), 6.61 (dd, J = 8.4, 7.2 Hz, 1H,-CH-alkene), 5.09 (q, J = 7.2 Hz, 1H, CF_3-CH), 4.72 (dd, J = 15.2, 7.2 Hz, 1H, -N-CH-), 4.43, 3.90 (J_{AB} = 12.6 Hz, 2H, -O-CH_2-), 3.60 (dd, J = 15.2, 8.4 Hz, 1H, -N-CH-), 2.43 (s, 3H, CH_3), 2.39 (s, 3H, CH_3). **^{13}C NMR** (126 MHz, $CDCl_3$) δ 144.17, 140.33, 139.72, 138.80, 136.65, 134.21, 132.91, 132.22, 130.78, 129.90, 129.58, 129.57, 128.95, 128.81, 127.89, 127.80, 124.41 (q, J = 281.5 Hz), 70.93 (q, J = 32.3 Hz), 64.79, 49.18, 21.70, 21.56. **^{19}F NMR** (282 MHz, $CDCl_3$) δ -72.32 (d, J = 7.2 Hz, 3F). **IR** (KBr): 3060, 2956, 2921, 1594, 1355, 1284, 1149, 1060, 881, 813, 715 cm^{-1}. **HRMS** (ESI) calculated for $C_{26}H_{23}ClF_3NO_3SNa$ $[M+Na]^+$ 544.0937, found 544.0941.

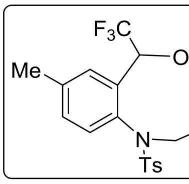

3be

10-Fluoro-4-phenyl-1-tosyl-7-(trifluoromethyl)-1,2,5,7-tetrahydrobenzo[*c*][1,5] oxazonine (3ca) Light yellow solid, 69% isolated yield, mp. 169.5-172.6 °C. By using method E, in DCE solution $Pd(PPh_3)_4$ (5 mol %; 0.006 g) was taken. To it **1c** (0.1 mmol, 0.039 g, 1.0 equiv) and **2a** (0.15 mmol, 0.029 g, 1.5 equiv) were added. After being stirred at 80 °C for 12 h, the mixture was dried in vacuo. The crude product was purified by flash column chromatography (using 8:2 hexane/ethyl acetate) to obtain the pure product **3ca**. **^{1}H NMR** (400 MHz, $CDCl_3$) δ 7.76 (dd, J = 8.8, 6.2 Hz, 1H, ArH), 7.69 – 7.66 (m, 1H, ArH), 7.66 – 7.64 (m, 1H, ArH),

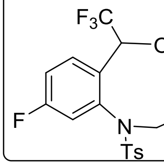

3ca

7.50 – 7.48 (m, 1H, ArH), 7.47 (dd, J = 2.0, 1.3 Hz, 1H, ArH), 7.39 – 7.35 (m, 1H, ArH), 7.35 – 7.32 (m, 2H, ArH), 7.31 (d, J = 0.6 Hz, 1H, ArH), 7.29 (d, J = 0.6 Hz, 1H, ArH), 7.19 – 7.15 (m, 1H, ArH), 6.77 (dd, J = 9.1, 2.7 Hz, 1H, ArH), 6.57 (dd, J = 8.8, 7.2 Hz, 1H,-CH-alkene), 5.07 (q, J = 7.1 Hz, 1H, CF_3-CH), 4.74 (dd, J = 15.2, 7.2 Hz, 1H, -N-CH-), 4.51, 3.95 (J_{AB} = 12.6 Hz, 2H, -O-CH_2-), 3.65 (dd, J = 15.2, 8.8 Hz, 1H, -N-CH-), 2.43 (s, 3H, CH_3). **^{13}C NMR** (126 MHz, $CDCl_3$) δ 163.55 (d, J = 253.6 Hz), 144.59, 143.73 (d, J = 9.5 Hz), 140.23 (d, J = 9.1 Hz), 136.15, 130.73 (d, J = 7.1 Hz), 130.08, 129.68, 129.66, 129.57, 128.69, 128.35, 127.80, 126.53, 124.26 (q, J = 281.5 Hz), 117.49 (d, J = 21.3 Hz), 116.54 (d, J = 21.4 Hz), 70.67 (q, J = 32.5 Hz), 65.20, 48.98, 21.71. **^{19}F NMR** (282 MHz, $CDCl_3$) δ -72.60 (d, J = 7.1 Hz, 3F), -108.41 (dd, J = 15.0, 7.8 Hz, 1F). **IR** (KBr): 3068, 2960, 2925, 1594, 1351, 1276, 1160, 1135, 1097, 1056, 842, 757, 694 cm^{-1}. **HRMS** (ESI) calculated for $C_{25}H_{21}F_4NO_3SNa$ $[M+Na]^+$ 514.1076, found 514.1082.

10-Fluoro-4-(p-tolyl)-1-tosyl-7-(trifluoromethyl)-1,2,5,7-tetrahydrobenzo[*c*][1,5] oxazonine (3cb) Light yellow solid, 72% isolated yield, mp. 152.7-160.6 °C. By using

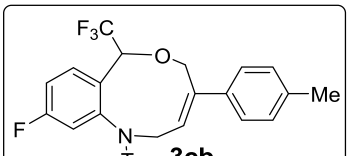

3cb

method E, in DCE solution $Pd(PPh_3)_4$ (5 mol %; 0.006 g) was taken. To it **1c** (0.1 mmol, 0.039 g, 1.0 equiv) and **2b** (0.15 mmol, 0.031 g, 1.5 equiv) were added. After being stirred at 80 °C for 14 h, the mixture was dried in vacuo. The crude product was purified by flash column chromatography (using 8:2 hexane/ethyl acetate) to obtain the pure product **3cb**. **^{1}H NMR** (400 MHz, $CDCl_3$) δ 7.74 (dd, J = 8.8, 6.2 Hz, 1H, ArH), 7.67 (d, J = 1.7 Hz, 1H, ArH), 7.66 – 7.63 (m, 1H, ArH), 7.38 (d, J = 1.7 Hz, 1H, ArH), 7.38 – 7.35 (m, 1H, ArH), 7.31 – 7.27 (m, 2H, ArH), 7.19 – 7.13 (m, 3H, ArH), 6.78 (dd, J = 9.1, 2.7 Hz, 1H, ArH), 6.54 (dd, J = 8.8, 7.2 Hz, 1H, -CH-alkene), 5.05 (q, J = 7.2 Hz, 1H, CF_3-CH), 4.73 (dd, J = 15.0, 7.2 Hz, 1H, -N-CH-), 4.49, 3.93 (J_{AB} = 12.6 Hz, 2H, -O-CH_2-), 3.64 (dd, J = 15.0, 8.8 Hz, 1H, -N-CH-), 2.43 (s, 3H, CH_3), 2.36 (s, 3H, CH_3). **^{13}C NMR** (126 MHz, $CDCl_3$) δ 163.52 (d, J = 253.2 Hz), 144.55, 143.71 (d, J = 9.7 Hz), 140.06, 138.25, 137.24, 136.18, 130.69 (d, J = 7.1 Hz), 130.05, 129.68 (d, J = 3.6 Hz), 129.37, 128.66, 127.78, 126.38, 124.26 (q, J = 281.6 Hz), 117.44 (d, J = 21.1 Hz), 116.56 (d, J = 21.4 Hz), 70.62 (q, J = 32.5 Hz), 65.16, 49.02, 21.69, 21.28. **^{19}F NMR** (282 MHz, $CDCl_3$) δ -72.63 (d, J = 7.2 Hz, 3F), -108.49 (dd, J = 14.8, 7.8 Hz, 1F). **IR** (KBr): 3031, 2948, 1594, 1375, 1355, 1284, 1164, 1060, 892, 809, 686 cm^{-1}. **HRMS** (ESI) calculated for $C_{26}H_{23}F_4NO_3SNa$ $[M+Na]^+$ 528.1232, found 528.1229.

10-Fluoro-4-(4-fluorophenyl)-1-tosyl-7-(trifluoromethyl)-1,2,5,7-tetrahydrobenzo [*c*][1,5]oxazonine (3cd) White solid, 33% isolated yield, mp. 159.2-166.6 °C. By using

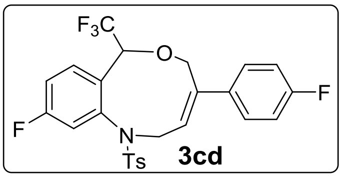

method E, in DCE solution $Pd(PPh_3)_4$ (5 mol %; 0.006 g) was taken. To it **1c** (0.1 mmol, 0.039 g, 1.0 equiv) and **2d** (0.15 mmol, 0.031 g, 1.5 equiv) were added. After being stirred at 80 °C for 14 h, the mixture was dried in vacuo. The crude product was purified by flash column chromatography (using 8:2 hexane/ethyl acetate) to obtain the pure product **3cd**. **^{1}H NMR** (400 MHz, $CDCl_3$) δ 7.76 (dd, *J* = 8.8, 6.2 Hz, 1H, ArH), 7.67 (d, *J* = 1.7 Hz, 1H, ArH), 7.65 (d, *J* = 1.7 Hz, 1H, ArH), 7.50 – 7.44 (m, 2H, ArH), 7.30 (d, *J* = 7.9 Hz, 2H, ArH), 7.20 – 7.16 (m, 1H, ArH), 7.08 – 7.01 (m, 2H, ArH), 6.74 (dd, *J* = 9.0, 2.7 Hz, 1H, ArH), 6.53 (dd, *J* = 8.4, 7.2 Hz, 1H, -CH-alkene), 5.08 (q, *J* = 7.0 Hz, 1H, CF_3-CH), 4.72 (dd, *J* = 15.0, 7.2 Hz, 1H, -N-CH-), 4.46, 3.92 (J_{AB} = 12.6 Hz, 2H, -O-CH_2-), 3.63 (dd, *J* = 15.0, 8.4 Hz, 1H,-N-CH-), 2.44 (s, 3H, CH_3). **^{13}C NMR** (126 MHz, $CDCl_3$) δ 163.57 (d, *J* = 253.9 Hz), 162.91 (d, *J* = 248.5 Hz), 144.64, 143.72 (d, *J* = 9.8 Hz), 139.22, 136.26 (d, *J* = 3.3 Hz), 136.09, 130.76 (d, *J* = 9.9 Hz), 130.09, 129.63, 129.57, 128.28 (d, *J* = 8.3 Hz), 127.79, 124.23 (q, *J* = 281.4 Hz), 117.56 (d, *J* = 21.2 Hz), 116.48 (d, *J* = 21.6 Hz), 115.58 (d, *J* = 21.3 Hz), 70.64 (q, *J* = 32.5 Hz), 65.10, 48.94, 21.72. **^{19}F NMR** (282 MHz, $CDCl_3$) δ -72.63 (d, *J* = 7.0 Hz, 3F), -108.25 (dd, *J* = 15.0, 7.6 Hz, 1F), -114.30 (dd, *J* = 8.9, 3.8 Hz, 1F). **IR** (KBr): 3060, 2948, 2308, 1598, 1355, 1276, 1160, 1097, 1056, 892, 817, 690 cm^{-1}. **HRMS** (ESI) calculated for $C_{25}H_{20}F_5NO_3SNa$ $[M+Na]^+$ 532.0982, found 532.0998.

9-Chloro-4-(p-tolyl)-1-tosyl-7-(trifluoromethyl)-1,2,5,7-tetrahydrobenzo[*c*] [1,5]oxazonine (3db) Light yellow solid, 40% isolated yield, mp. 61.2-79.9 °C. By using method E, in DCE solution $Pd(PPh_3)_4$ (5 mol %; 0.006 g) was taken. To it **1d** (0.1 mmol,

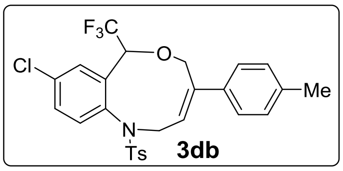

0.041 g, 1.0 equiv) and **2b** (0.15 mmol, 0.031 g, 1.5 equiv) were added. After being stirred at 80 °C for 15 h, the mixture was dried in vacuo. The crude product was purified by flash column chromatography (using 8:2 hexane/ethyl acetate) to obtain the pure product **3db**. **^{1}H NMR** (400 MHz, $CDCl_3$) δ 7.72 (d, *J* = 2.4 Hz, 1H, ArH), 7.63 (d, *J* = 8.4 Hz, 2H, ArH), 7.39 (d, *J* = 1.0 Hz, 1H, ArH), 7.36 (dd, *J* = 3.6, 2.1 Hz, 2H, ArH), 7.29 (d, *J* = 0.6 Hz, 1H, ArH), 7.26 (d, *J* = 3.0 Hz, 1H, ArH), 7.18 (d, *J* = 0.5 Hz, 1H, ArH), 7.16 (s, 1H, ArH), 6.97 (d, *J* = 8.6 Hz, 1H, ArH), 6.55 (dd, *J* = 8.4, 7.2 Hz, 1H, -CH-alkene), 5.05 (q, *J* = 7.1 Hz, 1H, CF_3-CH), 4.75 (dd, *J* = 15.0, 7.2 Hz, 1H, -N-CH-), 4.51, 3.93 (J_{AB} = 12.6 Hz, 2H, -O-CH_2-), 3.61 (dd, *J* = 15.0, 8.4 Hz, 1H, -N-CH-), 2.42 (s, 3H, CH_3), 2.36 (s, 3H, CH_3). **^{13}C NMR** (126 MHz, $CDCl_3$) δ 144.44, 140.65,

139.87, 138.29, 137.16, 136.30, 135.83, 135.34, 131.56, 130.81, 130.02, 129.40, 128.88, 127.98, 127.77, 126.37, 124.07 (q, J = 281.7 Hz), 70.80 (q, J = 32.5 Hz), 65.36, 49.18, 21.70, 21.30. **^{19}F NMR** (282 MHz, $CDCl_3$) δ -72.53 (d, J = 7.1 Hz, 3F). **IR** (KBr): 3100, 3027, 2929, 1695, 1511, 1344, 1276, 1176, 1068, 813, 734 cm^{-1}. **HRMS** (ESI) calculated for $C_{26}H_{23}ClF_3NO_3SNa$ $[M+Na]^+$ 544.0937, found 544.0953.

9-Bromo-4-phenyl-1-tosyl-7-(trifluoromethyl)-1,2,5,7-tetrahydrobenzo[*c*][1,5] oxazonine (3ea) Light yellow solid, 78% isolated yield, mp. 132.3-140.4 °C. By using method E, in DCE solution $Pd(PPh_3)_4$ (5 mol %; 0.006 g) was taken. To it **1e** (0.1 mmol, 0.045 g, 1.0 equiv) and **2a** (0.15 mmol, 0.029 g, 1.5 equiv) were added. After being stirred at 80 °C for 12 h, the mixture was dried in vacuo. The crude product was purified by flash column chromatography (using 8:2 hexane/ethyl acetate) to obtain the pure product **3ea**.

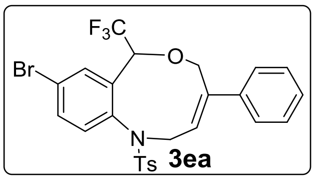

3ea

^{1}H NMR (300 MHz, $CDCl_3$) δ 7.89 (d, J = 2.1 Hz, 1H, ArH), 7.64 (d, J = 8.3 Hz, 2H, ArH), 7.53 (dd, J = 8.6, 2.3 Hz, 1H, ArH), 7.51 – 7.46 (m, 2H, ArH), 7.41 – 7.35 (m, 2H, ArH), 7.35 – 7.32 (m, 1H, ArH), 7.28 (d, J = 8.2 Hz, 1H, ArH), 7.27 (d, J = 3.5 Hz, 1H, ArH), 6.88 (d, J = 8.5 Hz, 1H, ArH), 6.59 (t, J = 7.9 Hz, 1H, -CH-alkene), 5.08 (q, J = 7.0 Hz, 1H, CF_3-CH), 4.75 (dd, J = 15.1, 7.3 Hz, 1H, -N-CH-), 4.52, 3.94 (J_{AB} = 12.7 Hz, 2H, -O-CH_2), 3.62 (dd, J = 15.0, 8.6 Hz, 1H, -N-CH-), 2.43 (s, 3H, CH_3). **^{13}C NMR** (126 MHz, $CDCl_3$) δ 144.49, 141.20, 140.11, 140.05, 136.26, 135.60, 134.60, 132.39, 131.00, 130.04, 129.81, 128.70, 128.37, 127.77, 126.52, 124.08 (q, J = 281.3 Hz), 123.96, 70.78 (q, J = 32.6 Hz), 65.39, 49.09, 21.70. **^{19}F NMR** (282 MHz, $CDCl_3$) δ -72.46 (d, J = 7.0 Hz, 3F). **IR** (KBr): 3079, 2956, 1805, 1602, 1348, 1280, 1164, 1060, 885, 769, 734 cm^{-1}. **HRMS** (ESI) calculated for $C_{25}H_{21}BrF_3NO_3SNa$ $[M+Na]^+$ 574.0275, found 574.0281.

9-Bromo-4-(p-tolyl)-1-tosyl-7-(trifluoromethyl)-1,2,5,7-tetrahydrobenzo[*c*][1,5] oxazonine (3eb) Light yellow solid, 61% isolated yield, mp. 122.0-129.2 °C. By using method E, in DCE solution $Pd(PPh_3)_4$ (5 mol %; 0.006 g) was taken. To it **1e** (0.1 mmol, 0.045 g, 1.0 equiv) and **2b** (0.15 mmol, 0.031 g, 1.5 equiv) were added. After being stirred at 80 °C for 15 h, the mixture was dried in vacuo. The crude product was purified by flash column chromatography (using 8:2 hexane/ethyl acetate) to obtain the pure product **3eb**.

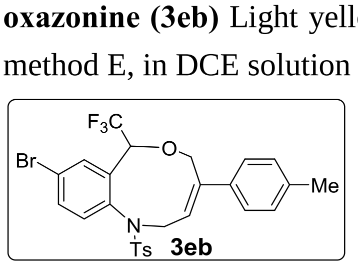

3eb

^{1}H NMR (300 MHz, $CDCl_3$) δ 7.88 (d, J = 2.1 Hz, 1H, ArH), 7.63 (d, J = 8.3 Hz, 2H, ArH), 7.53 (dd, J = 8.5, 2.3 Hz, 1H, ArH), 7.39 (d, J = 8.2 Hz, 2H, ArH), 7.29 (s, 1H, ArH), 7.25 (d, J = 6.4 Hz, 1H, ArH), 7.18 (d, J = 8.0 Hz, 2H, ArH),

6.90 (d, J = 8.5 Hz, 1H, ArH), 6.57 (dd, J = 8.4, 7.5 Hz, 1H, -CH-alkene), 5.05 (q, J = 7.0 Hz, 1H, CF_3-CH), 4.75 (dd, J = 15.1, 7.3 Hz, 1H, -N-CH-), 4.51, 3.92 (J_{AB} = 12.6 Hz, 2H, -O-CH_2), 3.61 (dd, J = 15.0, 8.6 Hz, 1H, -N-CH-), 2.43 (s, 3H, CH_3), 2.37 (s, 3H, CH_3). **^{13}C NMR** (126 MHz, $CDCl_3$) δ 144.45, 141.20, 139.87, 138.31, 137.16, 136.30, 135.61, 134.57, 132.39, 131.08, 130.03, 129.40, 128.88, 127.77, 126.38, 124.06 (q, J = 281.2 Hz), 123.92, 70.73 (q, J = 32.6 Hz), 65.37, 49.15, 21.71, 21.31. **^{19}F NMR** (282 MHz, $CDCl_3$) δ -72.48 (d, J = 6.8 Hz, 3F). **IR** (KBr): 3019, 2967, 1508, 1367, 1340, 1276, 1168, 1052, 877, 809, 734, 698 cm^{-1}. **HRMS** (ESI) calculated for $C_{26}H_{23}BrF_3NO_3SNa$ $[M+Na]^+$ 588.0432, found 588.0432.

9-Bromo-4-(4-fluorophenyl)-1-tosyl-7-(trifluoromethyl)-1,2,5,7-tetrahydrobenzo[*c*][1,5]oxazonine (3ed) Light yellow solid, 77% isolated yield, mp. 150.1-156.7 °C. By using method E, in DCE solution $Pd(PPh_3)_4$ (5 mol %; 0.006 g) was taken. To it **1e** (0.1 mmol, 0.045 g, 1.0 equiv) and **2d** (0.15 mmol, 0.031 g, 1.5 equiv) were added. After being stirred at 80 °C for 15 h, the mixture was dried in vacuo. The crude product was purified by flash column chromatography (using 8:2 hexane/ethyl acetate) to obtain the pure product **3ed**. **^{1}H NMR** (400 MHz, $CDCl_3$) δ 7.88 (d, J = 2.2 Hz, 1H, ArH), 7.63 (d, J = 8.0 Hz, 2H, ArH), 7.52 (dd, J = 8.5, 2.3 Hz, 1H, ArH), 7.49 – 7.44 (m, 2H, ArH), 7.29 (d, J = 7.9 Hz, 2H, ArH), 7.08 – 7.01 (m, 2H, ArH), 6.84 (d, J = 8.5 Hz, 1H, ArH), 6.55 (dd, J = 8.4, 7.2 Hz, 1H, -CH-alkene), 5.08 (q, J = 7.1 Hz, 1H, CF_3-CH), 4.73 (dd, J = 15.2, 7.2 Hz, 1H, -N-CH-), 4.47, 3.91 (J_{AB} = 12.6 Hz, 2H, -O-CH_2-), 3.60 (dd, J = 15.2, 8.4 Hz, 1H, -N-CH-), 2.43 (s, 3H, CH_3). **^{13}C NMR** (126 MHz, $CDCl_3$) δ 162.92 (d, J = 247.8 Hz), 144.55, 141.19, 141.19, 139.00, 139.00, 136.18 (d, J = 3.4 Hz), 135.53, 134.66, 132.40, 130.92, 130.05, 128.28 (d, J = 8.1 Hz), 127.76, 124.06 (q, J = 281.6 Hz), 124.03, 115.59 (d, J = 21.2 Hz), 70.76 (q, J = 32.7 Hz), 65.29, 49.06, 21.70. **^{19}F NMR** (282 MHz, $CDCl_3$) δ -72.48 (d, J = 7.1 Hz, 3F), -114.21 (d, J = 4.5 Hz, 1F). **IR** (KBr): 3045, 2944, 1594, 1344, 1280, 1164, 1060, 958, 838, 723 cm^{-1}. **HRMS** (ESI) calculated for $C_{25}H_{20}BrF_4NO_3SNa$ $[M+Na]^+$ 592.0181, found 592.0181.

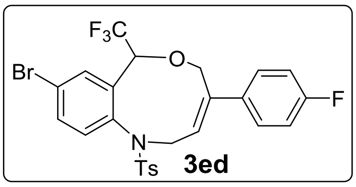

13. Application of CF_3-benzoxazonine 3aa

General procedure for the preparation of 1a-Phenyl-9-tosyl-4-(trifluoromethyl)-1a,2,4,9,10,10a-hexahydrobenzo[*c*]oxireno[2,3-*g*][1,5]oxazonine (4)

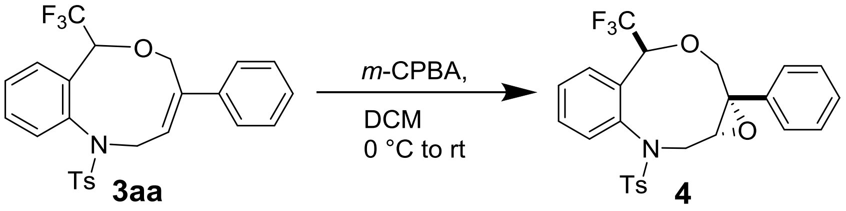

To a solution of nine-membered heterocycle **3aa** (0.10 mmol, 0.0473 g, 1.0 equiv) in DCM (2 mL) was added the DCM solution (2 mL) of *m*-CPBA (0.30 mmol, 0.0518 g, 3.0 equiv, 70 % wt/wt in water) dropwise at 0 °C under nitrogen. The reaction mixture was sealed under nitrogen and allowed to warm to room temperature and stirred for 11 h. The reaction was then quenched with saturated $NaHCO_3$ aq. solution and extracted with DCM. The combined organic phase was collected and dried with Na_2SO_4. After filtration and evaporation, the residue was purified by flash column chromatography (eluted with 8:2 hexane/ethyl acetate) to obtain the pure product **4** (32 mg, 67% yield) as white solid. This compound was observed as a mixture of two diastereoisomers in the NMR and the major diastereoisomer was isolated. Mp. 164.1-168.8 °C. **^{1}H NMR** (400 MHz, $CDCl_3$) δ 7.75 (d, J = 7.7 Hz, 2H, ArH), 7.73 – 7.71 (m, 1H, ArH), 7.59 – 7.57 (m, 1H, ArH), 7.57 – 7.55 (m, 1H, ArH), 7.49 – 7.44 (m, 1H, ArH), 7.43 – 7.40 (m, 1H, ArH), 7.39 (dd, J = 3.6, 1.7 Hz, 2H, ArH), 7.38 – 7.36 (m, 2H, ArH), 7.34 (d, J = 0.6 Hz, 1H, ArH), 6.81 (dd, J = 8.0, 1.2 Hz, 1H, ArH), 5.75 (q, J = 6.8 Hz, 1H, CF_3-CH), 4.70 (dd, J = 15.6, 5.2 Hz, 1H, -N-CH-), 4.31, 2.99 (J_{AB} = 13.0 Hz, 2H, -O-CH_2-), 3.96 (ddd, J = 8.8, 5.2, 1.0 Hz, 1H, -CH-epoxide), 2.83 (dd, J = 15.6, 8.8 Hz, 1H, -N-CH-), 2.47 (s, 3H, CH_3). **^{13}C NMR** (126 MHz, $CDCl_3$) δ 144.72, 143.41, 137.40, 135.92, 132.46, 131.64, 130.34, 130.14, 129.26, 128.91, 128.63, 128.38, 128.03, 127.42, 124.12 (q, J = 281.7 Hz), 71.44 (q, J = 32.4 Hz), 66.83, 61.48, 60.37, 53.44, 21.76. **^{19}F NMR** (282 MHz, $CDCl_3$) δ -72.61 (d, J = 6.8 Hz, 3F). **IR** (KBr): 3064, 2940, 1598, 1388, 1355, 1284, 1164, 1097, 966, 873, 817, 761, 727 cm^{-1}. **HRMS** (ESI) calculated for $C_{25}H_{22}F_3NO_4SNa$ $[M+Na]^+$ 512.1119, found 512.1122.

General procedure[9] for the preparation of 9-methyl-4-phenyl-1-tosyl-7-(trifluoromethyl)-1,2,3,4,5,7-hexahydrobenzo[*c*][1,5]oxazonine (5)

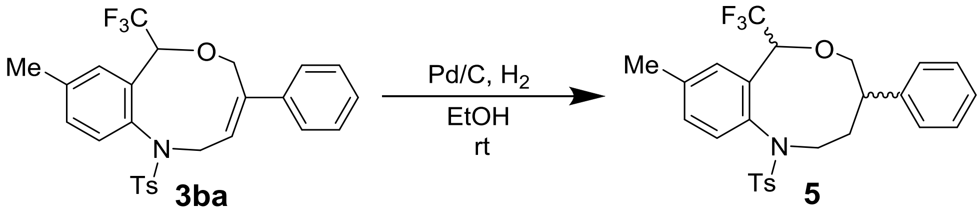

To a solution of nine-membered heterocycle **3ba** (0.03 mmol, 0.014g, 1.0 equiv.) in dry ethanol (2 mL). This mixture was degassed of dissolved air and purged with an argon atmosphere. To it 10% Pd/C (10 mol %, 0.0003g) was carefully added. The above reaction mixture was degassed and purged with hydrogen. The reaction is allowed to stir for 2 h at room temperature. After the completion of the reaction, the mixture was filtered through a celite pad and concentrated under reduced pressure and purified by flash column chromatography. (using 9:1 hexane/ethyl acetate) to obtain the pure product **5** as white solid 74% yield. This compound was observed as a mixture of two diastereoisomers in the NMR and the major diastereoisomer was isolated. mp. 73.1-76.5 °C. **^{1}H NMR** (300 MHz, $CDCl_3$) δ 7.57 (d, *J* = 8.3 Hz, 2H, ArH), 7.42 – 7.33 (m, 4H, ArH), 7.36 – 7.29 (m, 2H, ArH), 7.29 – 7.24 (m, 2H, ArH), 7.02 (d, *J* = 8.0 Hz, 1H, ArH), 6.35 (d, *J* = 8.0 Hz, 1H, ArH), 5.67 (q, *J* = 7.3 Hz, 1H, CF_3-CH), 4.68 (dd, *J* = 12.5, 10.6 Hz, 1H, -O-CH-), 4.50 (dd, *J* = 10.2, 5.4 Hz, 1H, -NCH-), 4.28 – 4.18 (m, 1H, -O-CH-), 3.25 – 3.13 (m, 1H, PhCH), 2.92 (ddd, *J* = 12.9, 5.4, 1.5 Hz, 1H, -NCH-), 2.46 (s, 3H, CH_3), 2.38 (s, 3H, CH_3), 1.97 – 1.83 (m, 1H, -CH-CPh), 1.64 – 1.55 (m, 1H, -CH-CPh). **^{13}C NMR** (126 MHz, $CDCl_3$) δ 144.26, 140.63, 139.33, 137.41, 137.36, 134.11, 130.03, 129.73, 128.94, 128.61, 128.51, 127.45, 127.10, 126.74, 125.08 (q, *J* = 271.0 Hz), 80.32 (q, *J* = 31.5 Hz), 78.26, 48.50, 44.70, 29.43, 21.77, 21.68. **^{19}F NMR** (282 MHz, $CDCl_3$) δ -72.83 (d, *J* = 7.3 Hz, 3F). **IR** (KBr): 3054, 2912, 1590, 1448, 1336, 1272, 1168, 1128, 1093, 898, 831, 765, 707 cm^{-1}. LC-MS (ESI, m/z): $[M+Na]^+$ 512.10.

14. Preparation of 4-Methyl-1-tosyl-1,4-dihydro-2*H*-benzo[*d*][1,3]oxazin-2-one (6b) Cream colored solid, 64% yield, mp. 107.4-111.4 °C. Using the general method D, in a flame dried 100 mL round bottom flask, compound **S3f** (2.1 mmol, 0.34 g, 1.0 equiv) was suspended in dry DMF (8 mL) and allowed to cool to 0 °C. To this solution NaH (60% dispersion in mineral oil, 3.1 mmol, 0.075 g, 1.5 equiv) was added and the mixture was allowed to stir for 1 h under Ar atmosphere. After 1 h, a solution of *p*-toluenesulfonyl chloride (2.7 mmol, 0.515 g, 1.3 equiv) in dry DMF (2 mL) was added dropwise to the reaction mixture. Completion of the reaction was monitored by TLC After completion, the reaction mixture was poured into crushed ice followed by extraction with ethyl acetate. The crude product was purified by flash column chromatography (using 6:2 hexane/ethyl acetate) to obtain the pure product **6b**. ^{1}**H NMR** (500 MHz, $CDCl_3$) δ 8.14 (d, *J* = 8.4 Hz, 2H, ArH), 7.59 (d, *J* = 8.2 Hz, 1H, ArH), 7.41 (dd, *J* = 11.4, 4.7 Hz, 2H, ArH), 7.31 – 7.18 (m, 3H, ArH), 5.36 (q, *J* = 6.8 Hz, 1H, CH_3-CH), 2.47 (s, 3H, Ts-CH_3), 1.72 (d, *J* = 6.8 Hz, 3H, CH_3). ^{13}**C NMR** (126 MHz, $CDCl_3$) δ 149.91, 145.73, 136.03, 134.42, 129.95, 129.14, 129.11, 129.06, 126.20, 123.88, 120.65, 75.21, 21.89, 18.23. **IR** (KBr): 1752, 1595, 1374, 1335, 1222, 1173, 1085, 1056, 761 cm^{-1}. **HRMS** (ESI) calculated for $C_{16}H_{15}NO_4SNa$ $[M+Na]^+$ 340.0619, found 340.0638.

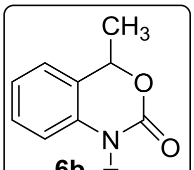

6b

Preparation of 1-benzyl-4-(trifluoromethyl)-1,4-dihydro-2H-benzo[d][1,3]oxazin-2-one (6c) White solid, 63% yield, mp. 133-134°C. Using the general method D, in a flame dried 20 mL round bottom flask, compound **S3a** (1.0 mmol, 0.217 g, 1.0 equiv) was suspended in dry DMF (5 mL) and allowed to cool to 0 °C. To this solution NaH (60% dispersion in mineral oil, 1.5 mmol, 0.036 g, 1.5 equiv) was added and the mixture was allowed to stir for 1 h under Ar atmosphere. After 1 h, a solution of benzyl bromide (1.1 mmol, 0.13 mL, 1.1 equiv) was added dropwise to the reaction mixture. Completion of the reaction was monitored by TLC After completion, the reaction mixture was poured into crushed ice followed by extraction with ethyl acetate. The crude product was purified by flash column chromatography (using 8:2 hexane/ethyl acetate) to obtain the pure product **6c**. ^{1}**H NMR** (300 MHz, $CDCl_3$) δ 7.38 – 7.30 (m, 3H, ArH), 7.29 – 7.21 (m, 4H, ArH), 7.16 – 7.08 (m, 1H, ArH), 6.89 (d, *J* = 8.3 Hz, 1H, ArH), 5.60 (q, *J* = 6.8 Hz, 1H, CF_3-CH), 5.18 (s, 2H, CH_2). ^{13}**C NMR** (126 MHz, $CDCl_3$) δ 149.88, 137.21, 135.51, 131.27, 129.07, 127.76, 127.34, 126.57, 123.61, 122.93 (q, *J* = 284.3 Hz), 114.85, 113.13, 75.57

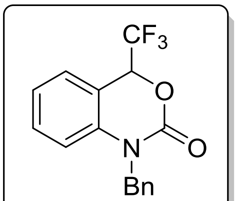

6c

(q, J = 34.1 Hz), 48.37. **^{19}F NMR** (282 MHz, $CDCl_3$) δ -79.29 (d, J = 6.7 Hz, 3F). **IR** (KBr): 3073, 2965, 1724, 1606, 1455, 1390, 1139, 1095, 914 cm^{-1}. LCMS (ESI^+) m/z: 308.25 $[M+H]^+$

15. X-Ray crystallographic structures are shown below

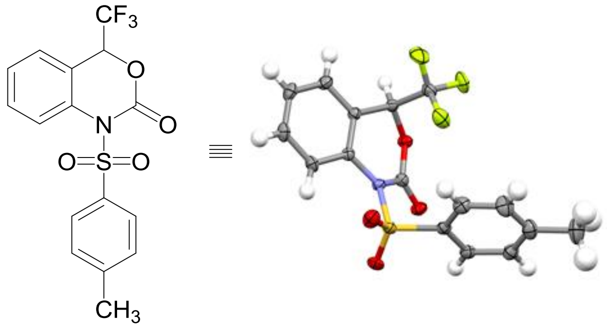

1a

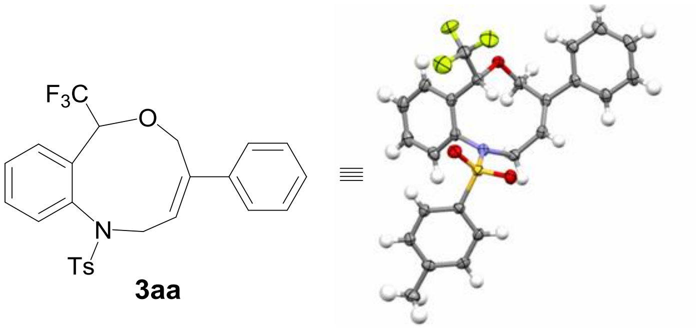

3aa

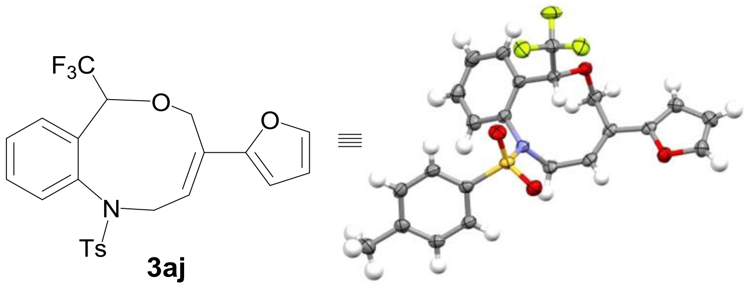

3aj

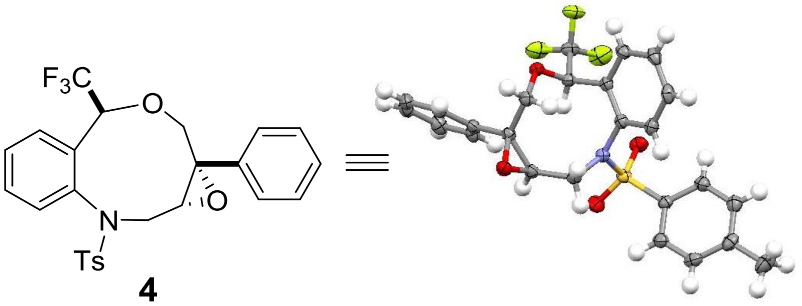

4

16.References

(1) S. Spirtovic-Halilovic, M. Salihovic, E. Veljovic, A. Osmanovic, S. Trifunovic and D. Zavrsnik, *Bulletin of the Chemists and Technologists of Bosnia and Herzegovina,* 2014, **43**, 57-60.

(2) L. Chen, G. M. Yang, J. Wang, Q. F. Jia, J. Wei and Z. Y. Du, *RSC Adv.*, 2015, **5**, 76696—76699.

(3) G. Zhan, M.-L. Shi, Q. He, W. Du and Y.-C. Chen, *Org. Lett.*, 2015, **17**, 4750—4753.

(4) A. M. Wagner, C. E. Knezevic, J. L. Wall, V. L. Sun, J. A. Buss, L. N. Allen and A. G. Wenzel, *Tetrahedron Letters,* 2012, **53**, 833—836.

(5) S. Chandrasekhar, C. Narsihmulu and V. Jagadeshwar, *Synlett*, 2002, **5**, 771—772.

(6) (a) A. Khan, R. Zheng, Y. Kan, J. Ye, J. Xing and Y. J. Zhang, *Angew. Chem. Int. Ed.*, 2014, **53**, 6439—6442; (b) W. Guo, L. M.- Rodriguez, E. Martin, E. C. E.- Adan and A. W. Kleij, *Angew. Chem. Int. Ed.*, 2016, **55**, 11037—11040; (c) L.-C. Yang, Z.-Q. Rong, Y.-N. Wang, Z. Y. Tan, M. Wang and Y. Zhao, *Angew. Chem. Int. Ed.*, 2017, **56**, 2927—2931.

(7) G. K. Surya Prakash, C. Panja, H. Vaghoo, V. Surampudi, R. Kultyshev, M. Mandal, G. Rasul, T. Mathew and G. A. Olah, *J. Org. Chem.*, 2006, **71**, 6806—6813.

(8) L. Ren and N. Jiao, *Chem. Commun.*, 2014, **50**, 3706—3709.

(9) V. Rauniyar, H. Zhai and D. G. Hall, *J. Am. Chem. Soc.*, 2008, **130**, 8481–8490.

17. NMR Data
(^{1}H-NMR, ^{13}C-NMR and ^{19}F-NMR)

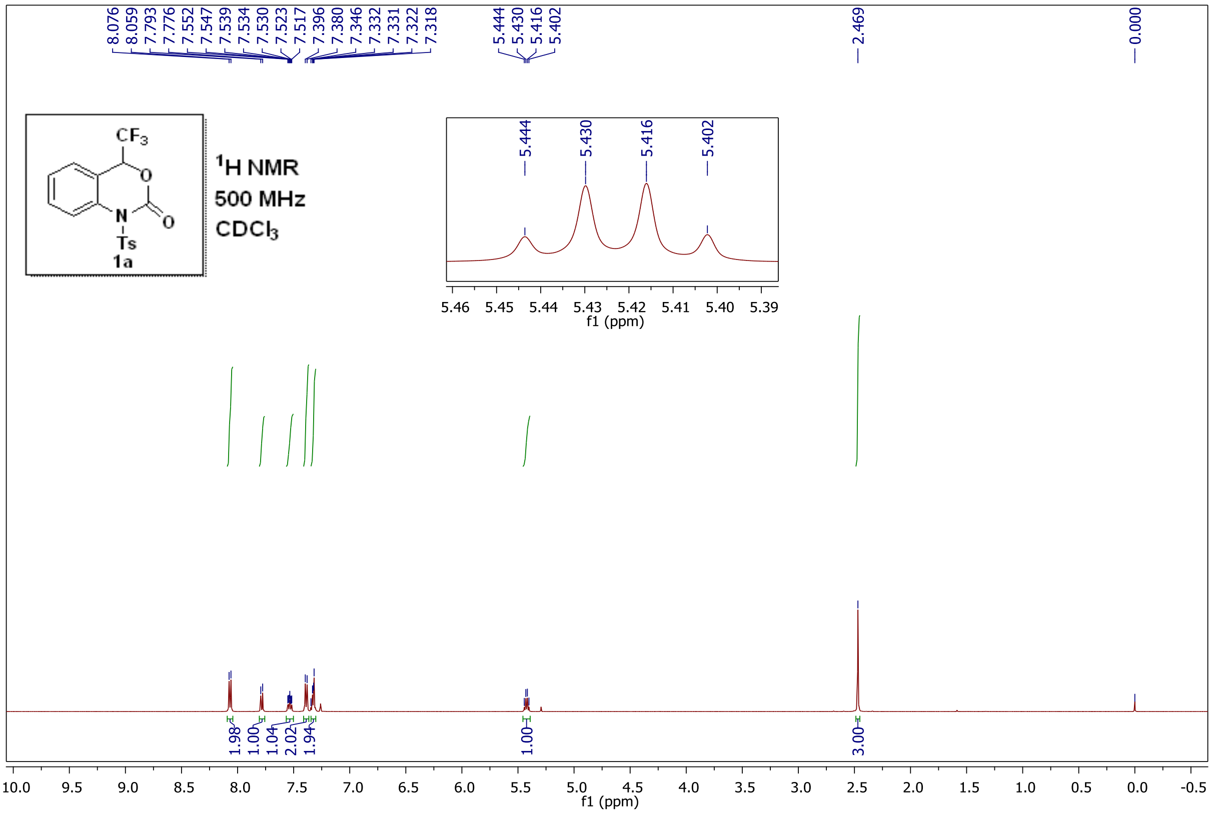
1H NMR
500 MHz
CDCl3
1a

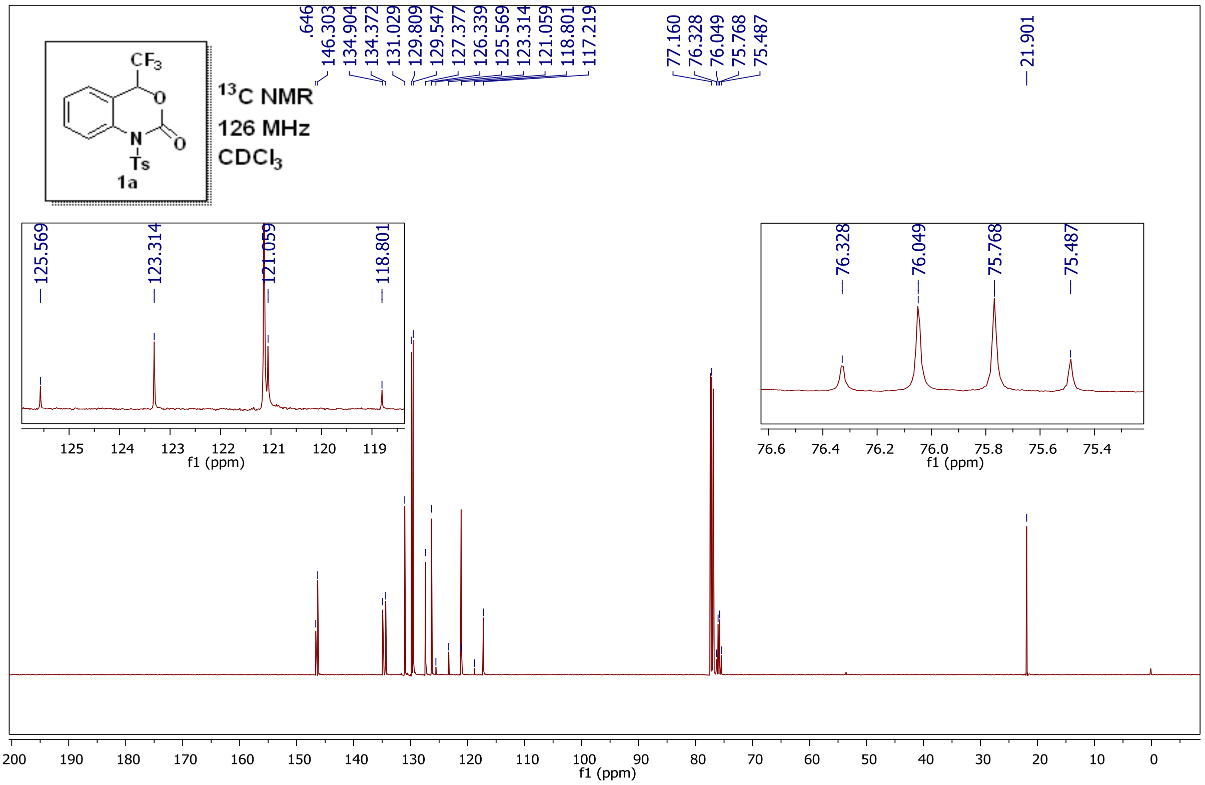
13C NMR
126 MHz
CDCl3
1a

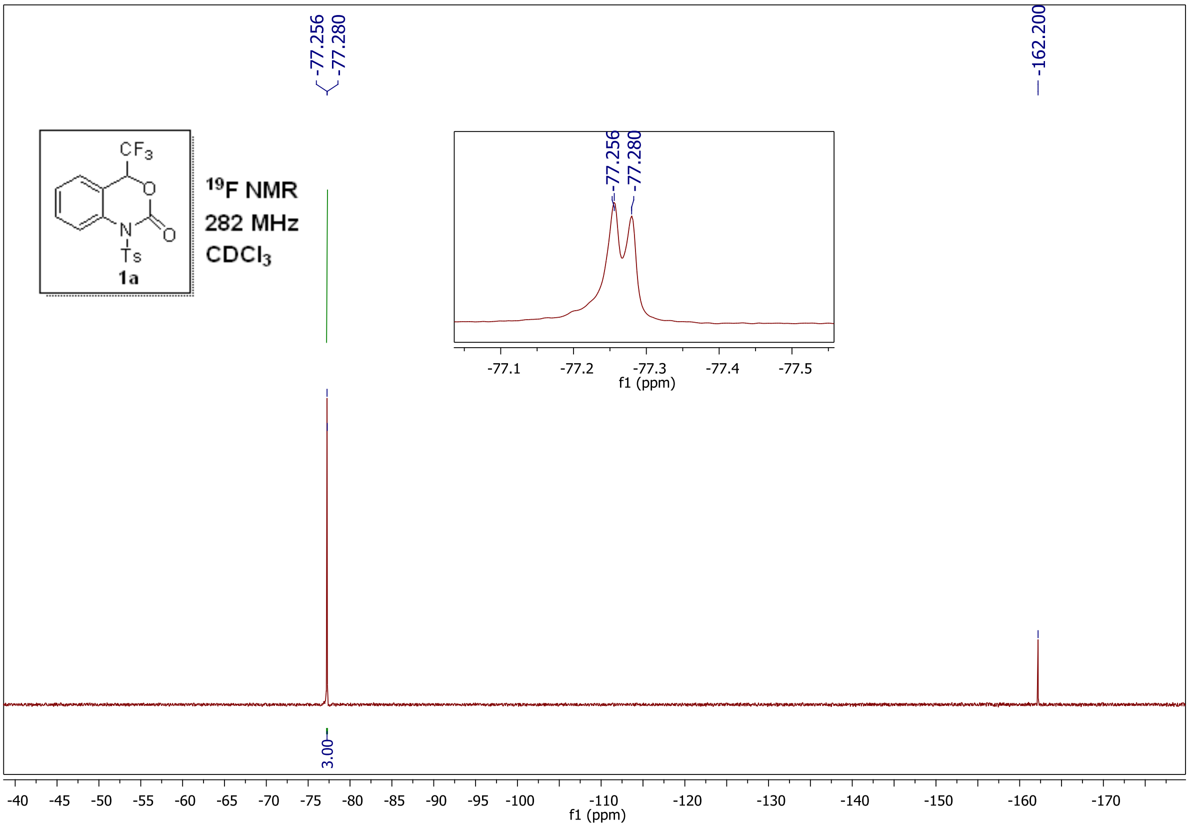

-77.256
-77.280
-162.200
CF3
O
N
O
Ts
1a
19F NMR
282 MHz
CDCl3
-77.1
-77.2
-77.3
-77.4
-77.5
f1 (ppm)
3.00
-40 -45 -50 -55 -60 -65 -70 -75 -80 -85 -90 -95 -100 -110 -120 -130 -140 -150 -160 -170
f1 (ppm)

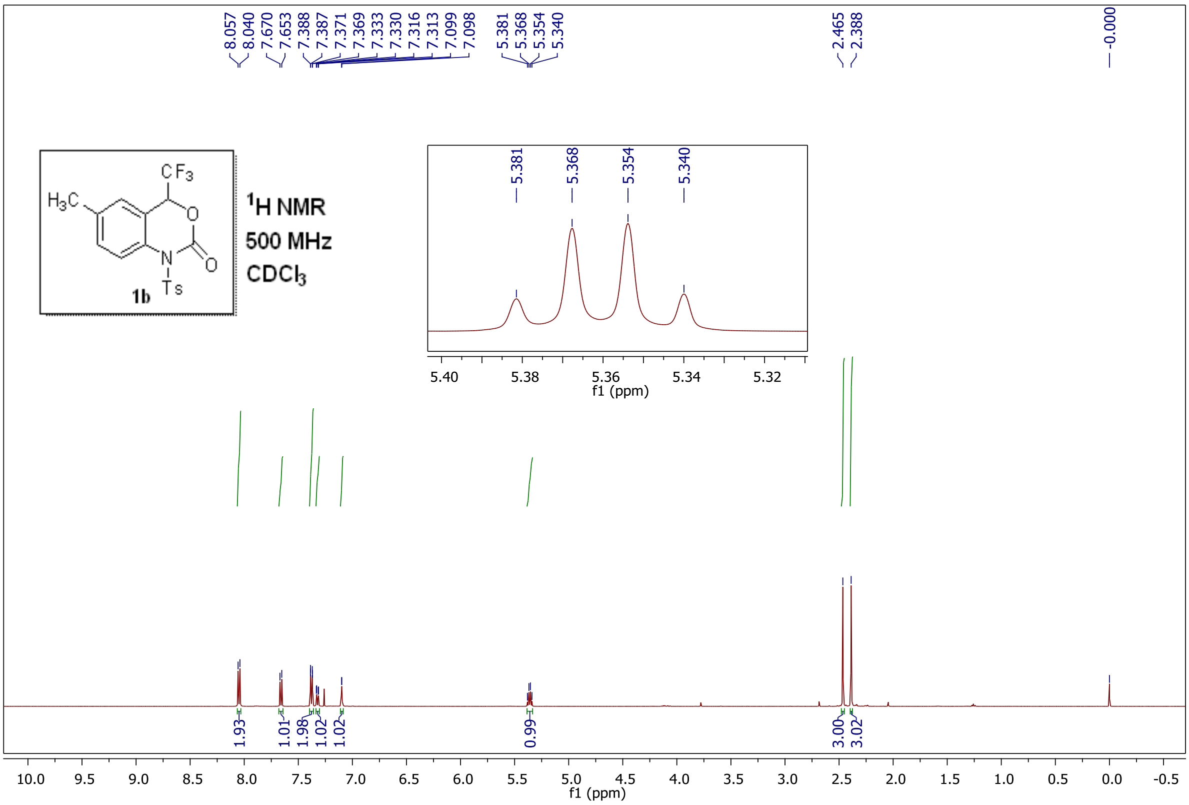

8.057 8.040 7.670 7.653 7.388 7.387 7.371 7.369 7.333 7.330 7.316 7.313 7.099 7.098 5.381 5.368 5.354 5.340 2.465 2.388 -0.000
CF3
H3C
O
N
O
Ts
1b
1H NMR
500 MHz
CDCl3
5.381
5.368
5.354
5.340
5.40 5.38 5.36 5.34 5.32
f1 (ppm)
1.93 1.01 1.98 1.02 1.02 0.99 3.00 3.02
10.0 9.5 9.0 8.5 8.0 7.5 7.0 6.5 6.0 5.5 5.0 4.5 4.0 3.5 3.0 2.5 2.0 1.5 1.0 0.5 0.0 -0.5
f1 (ppm)

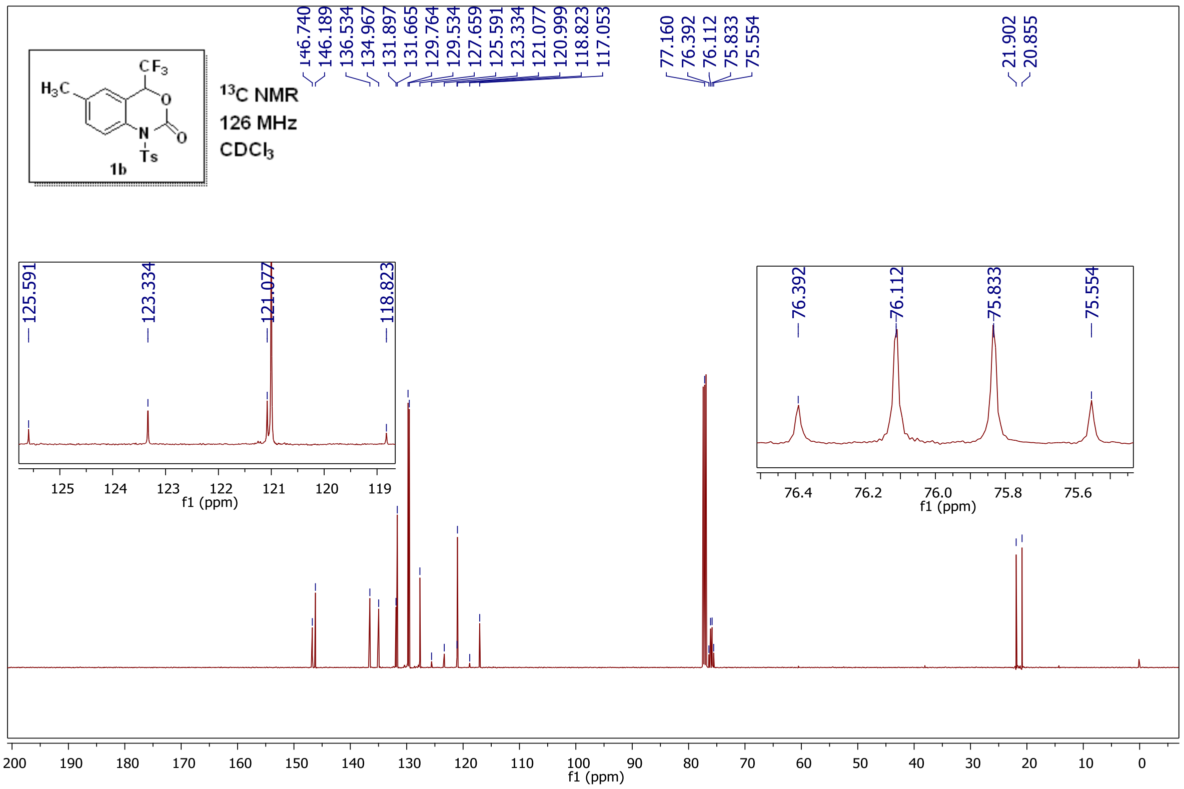

146.740
146.189
136.534
134.967
131.897
131.665
129.764
129.534
127.659
125.591
123.334
121.077
120.999
118.823
117.053
77.160
76.392
76.112
75.833
75.554
21.902
20.855
CF3
H3C
O
N
O
Ts
1b
13C NMR
126 MHz
CDCl3
f1 (ppm)

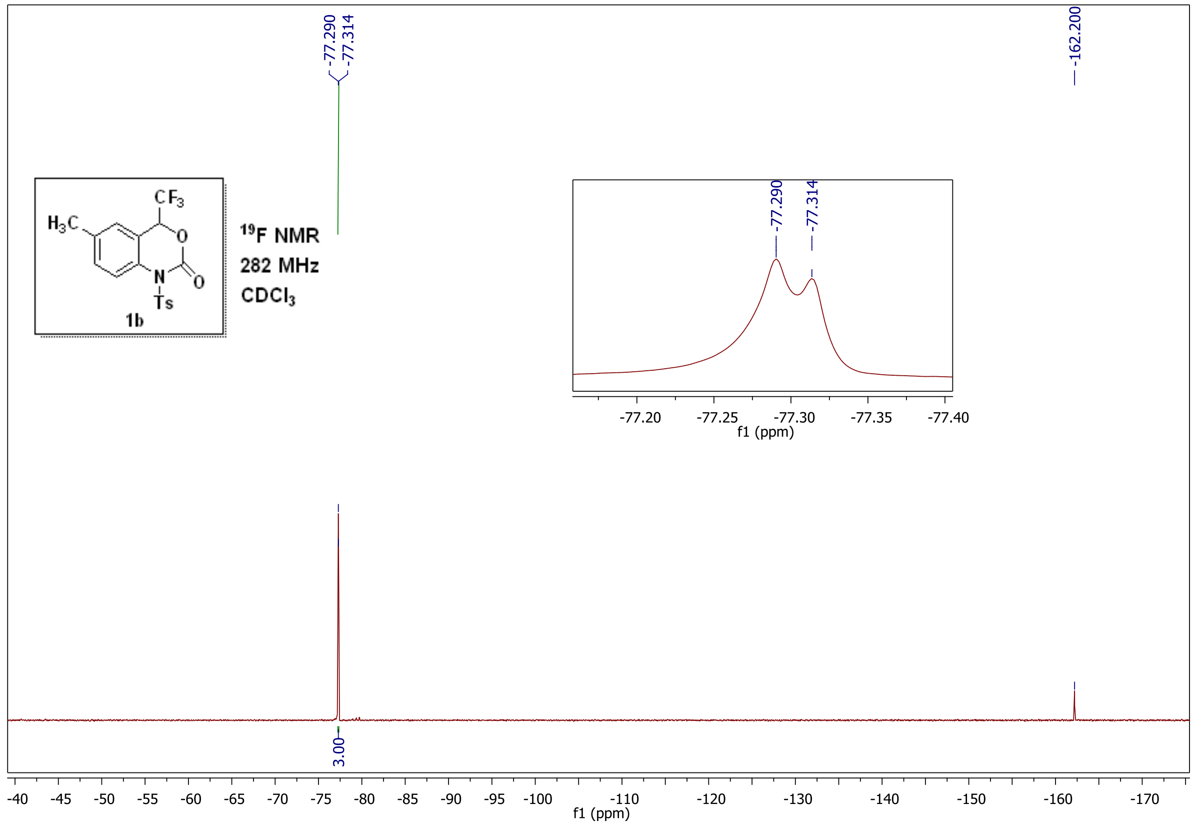

-77.290
-77.314
-162.200
CF3
H3C
O
N
O
Ts
1b
19F NMR
282 MHz
CDCl3
3.00
f1 (ppm)

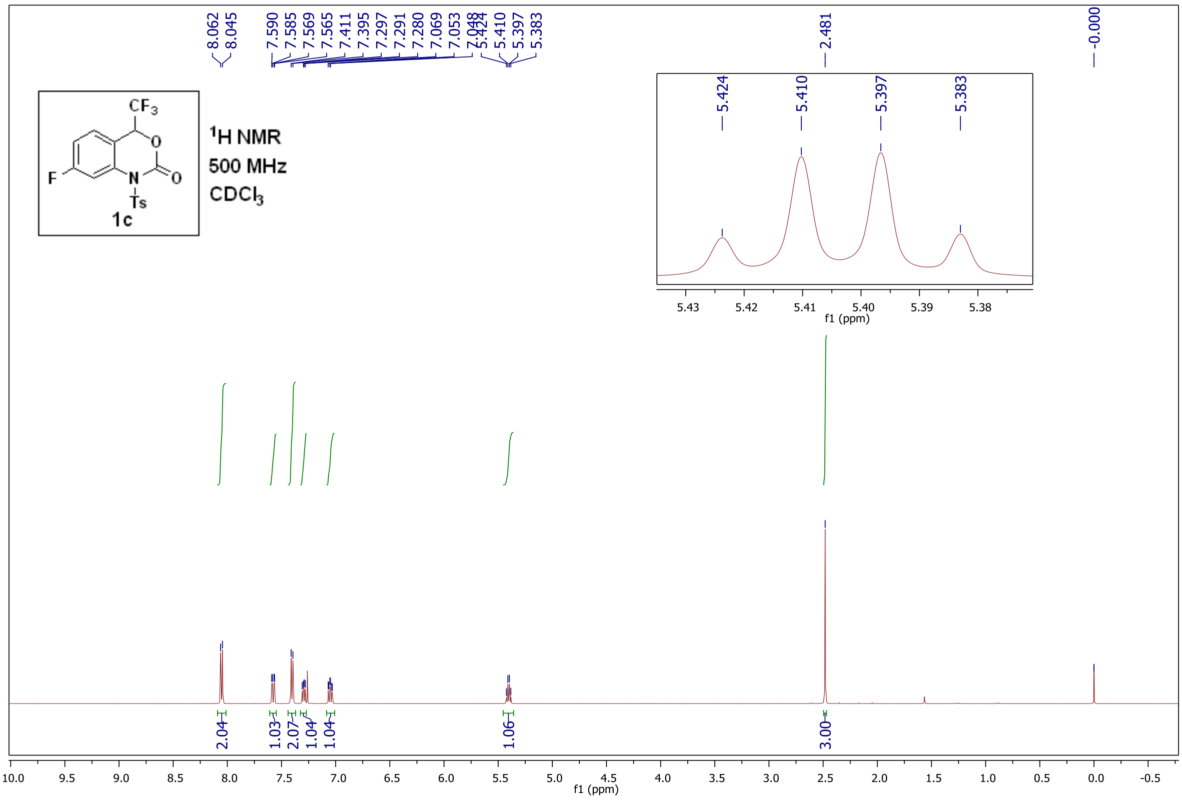

1H NMR
500 MHz
CDCl3
1c

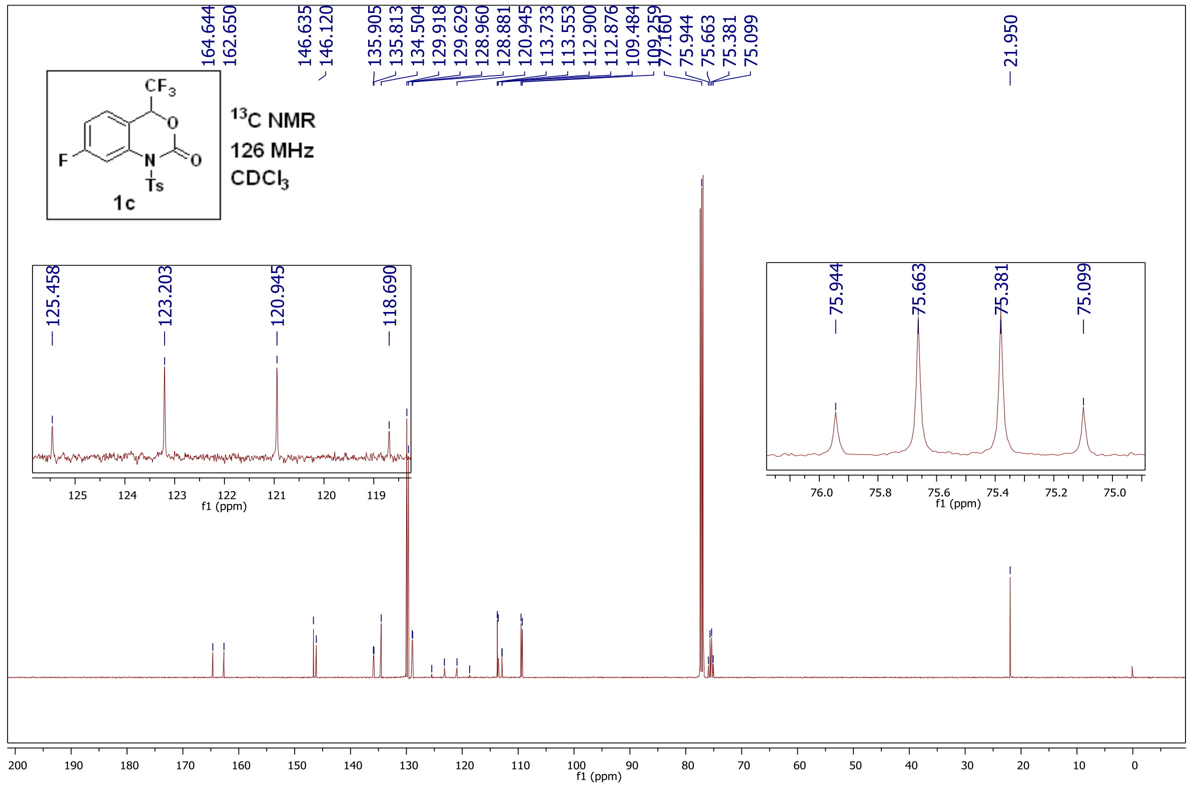

13C NMR
126 MHz
CDCl3
1c

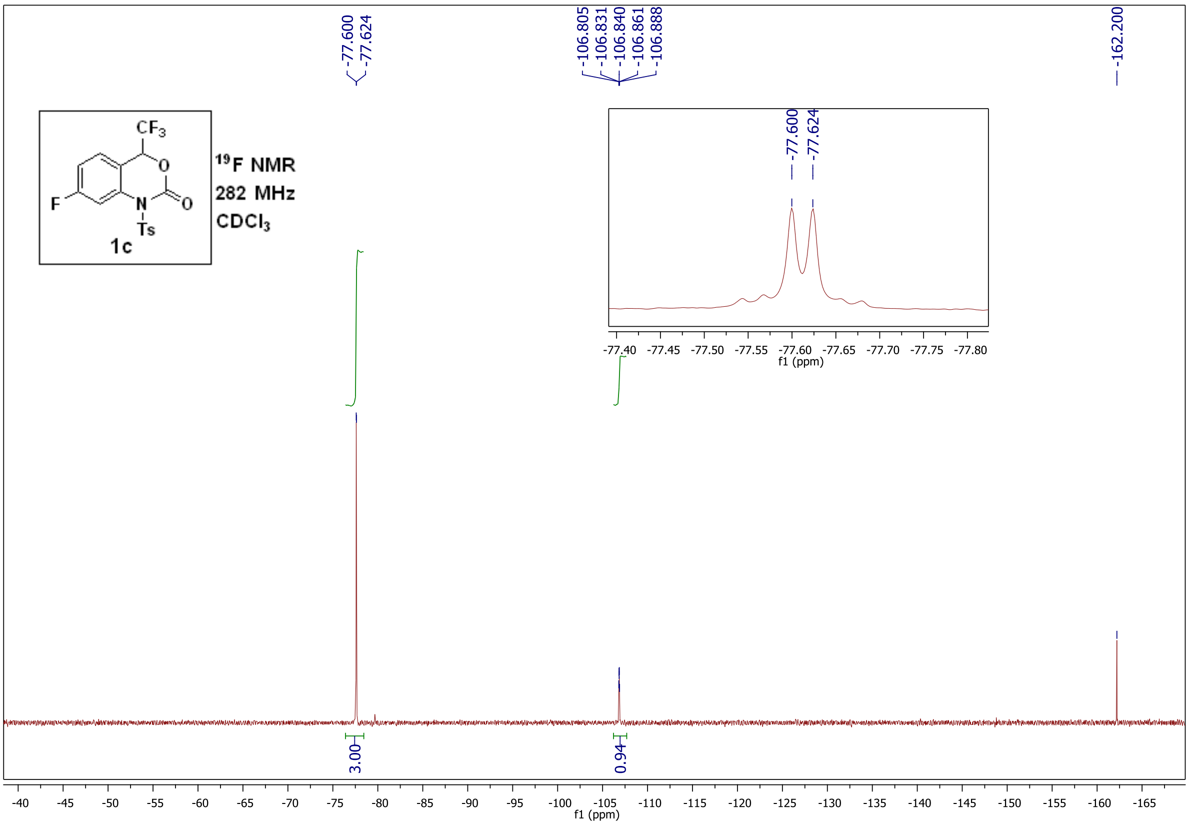

-77.600
-77.624
-106.805
-106.831
-106.840
-106.861
-106.888
-162.200
CF3
F
N
O
O
Ts
1c
19F NMR
282 MHz
CDCl3
f1 (ppm)
3.00
0.94

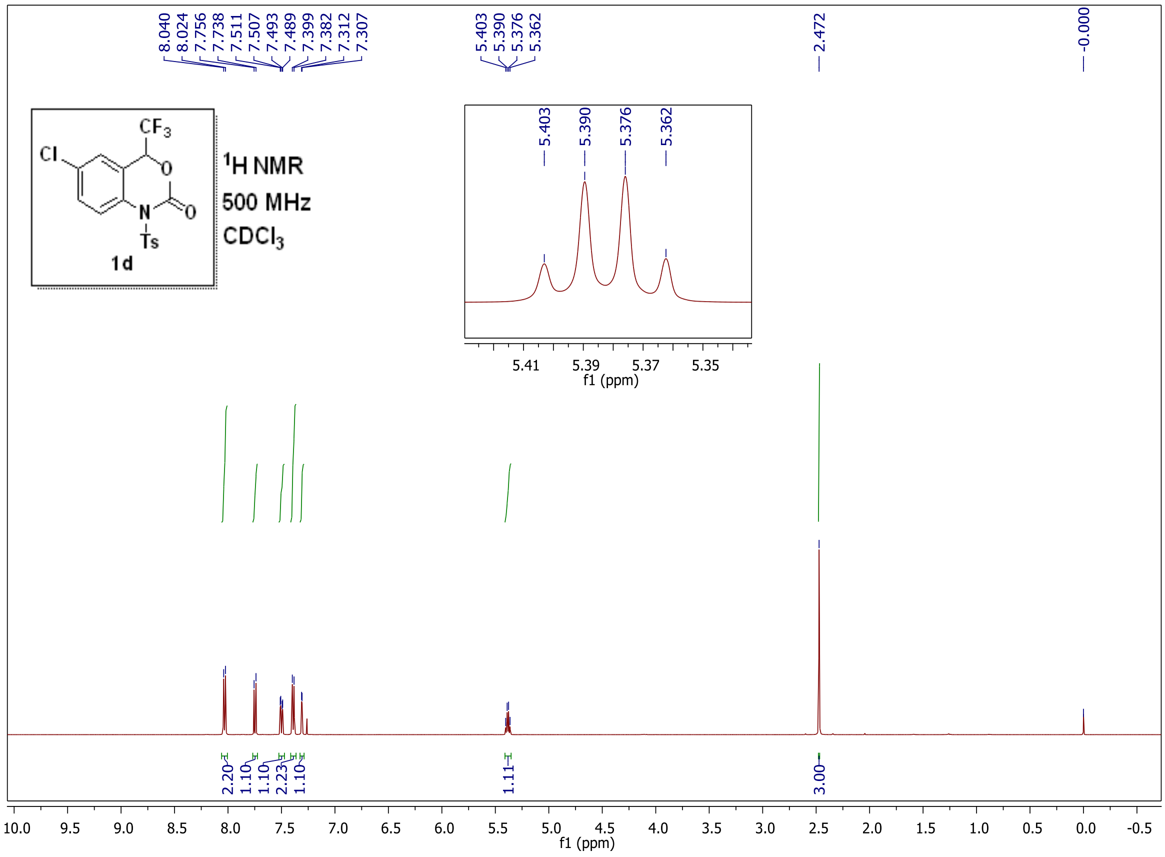

8.040
8.024
7.756
7.738
7.511
7.507
7.493
7.489
7.399
7.382
7.312
7.307
5.403
5.390
5.376
5.362
2.472
-0.000
CF3
Cl
N
O
O
Ts
1d
1H NMR
500 MHz
CDCl3
f1 (ppm)
2.20
1.10
1.10
2.23
1.10
1.11
3.00

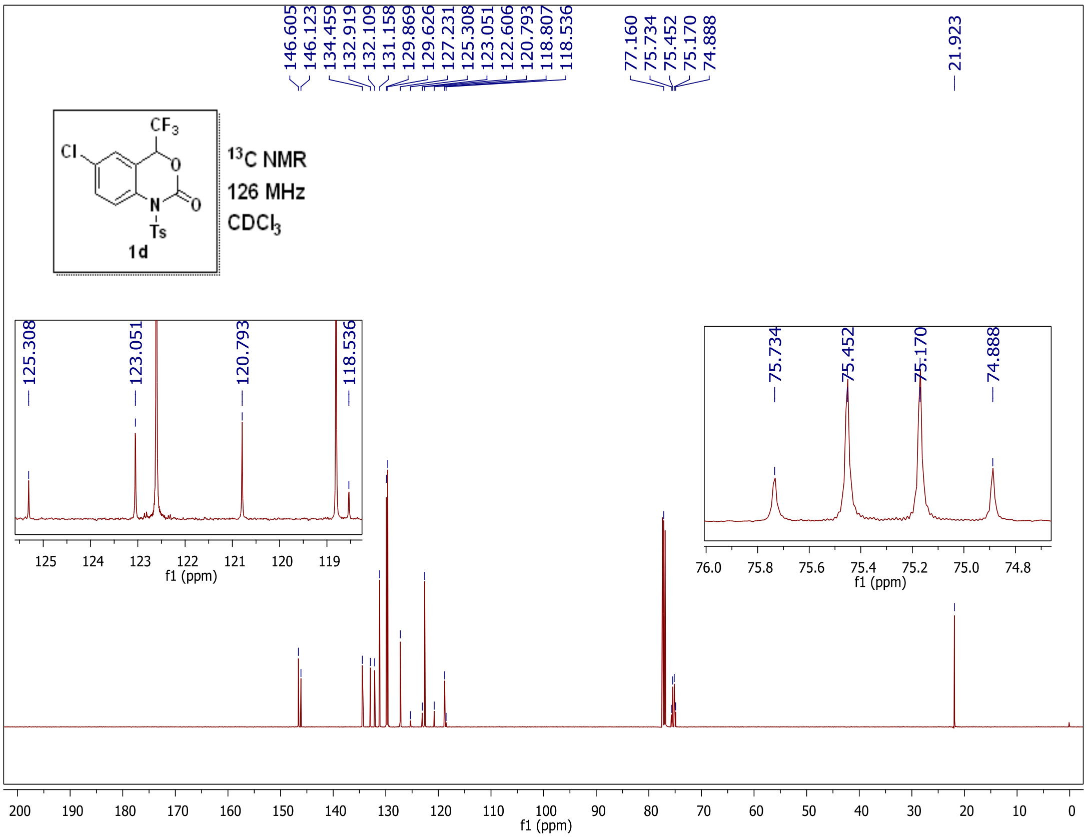
146.605
146.123
134.459
132.919
132.109
131.158
129.869
129.626
127.231
125.308
123.051
122.606
120.793
118.807
118.536
77.160
75.734
75.452
75.170
74.888
21.923
CF3
Cl
O
O
N
Ts
1d
13C NMR
126 MHz
CDCl3
f1 (ppm)

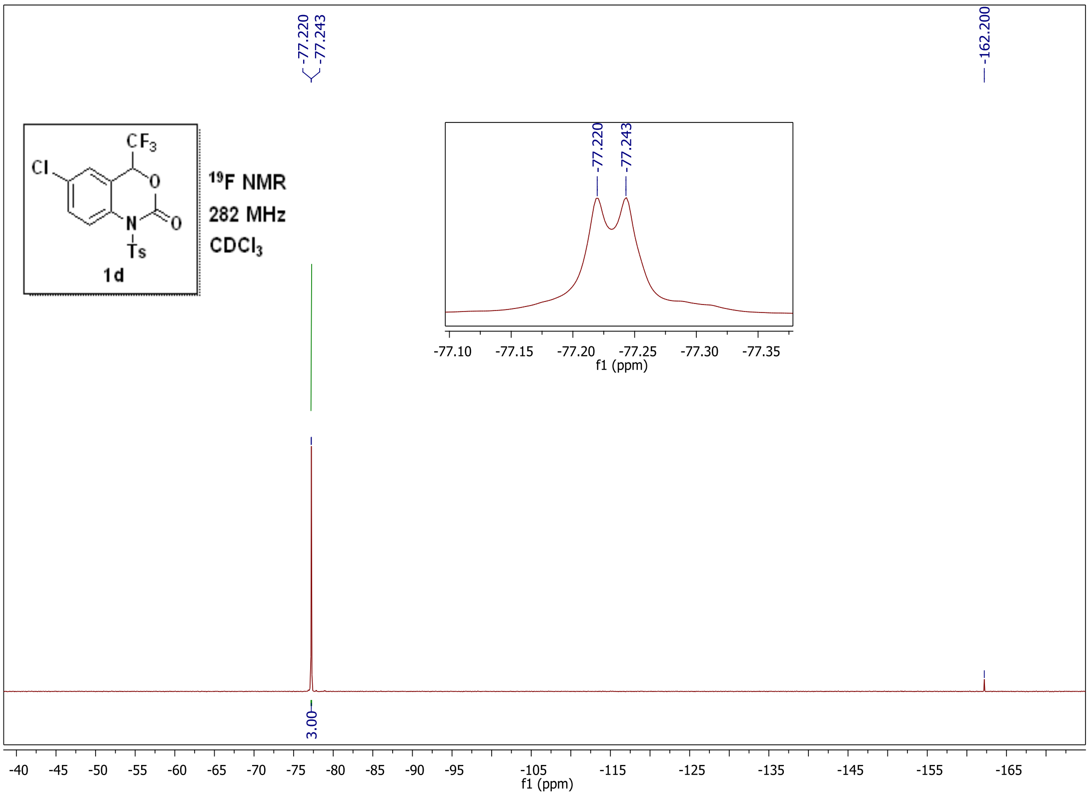
-77.220
-77.243
-162.200
CF3
Cl
O
O
N
Ts
1d
19F NMR
282 MHz
CDCl3
3.00
f1 (ppm)

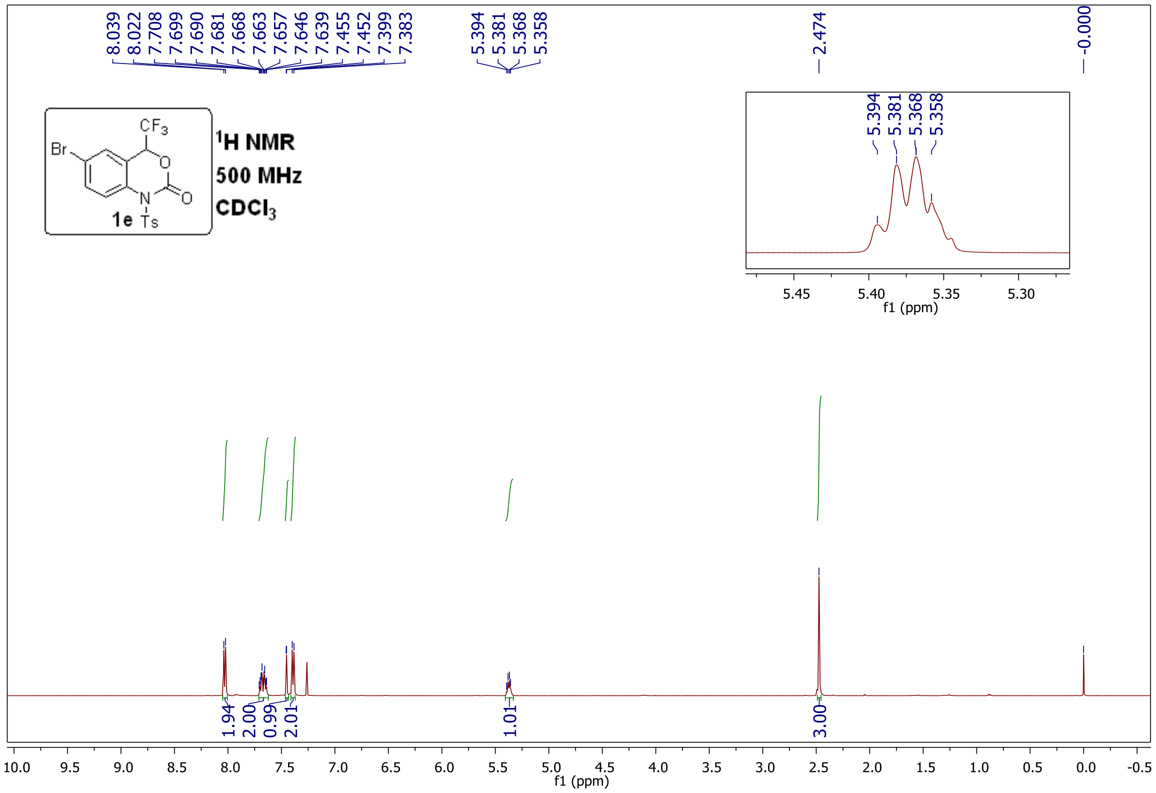

8.039
8.022
7.708
7.699
7.690
7.681
7.668
7.663
7.657
7.646
7.639
7.455
7.452
7.399
7.383
5.394
5.381
5.368
5.358
2.474
-0.000
Br
CF3
O
N
O
1e
Ts
^{1}H NMR
500 MHz
$CDCl_3$
1.94
2.00
0.99
2.01
1.01
3.00
f1 (ppm)

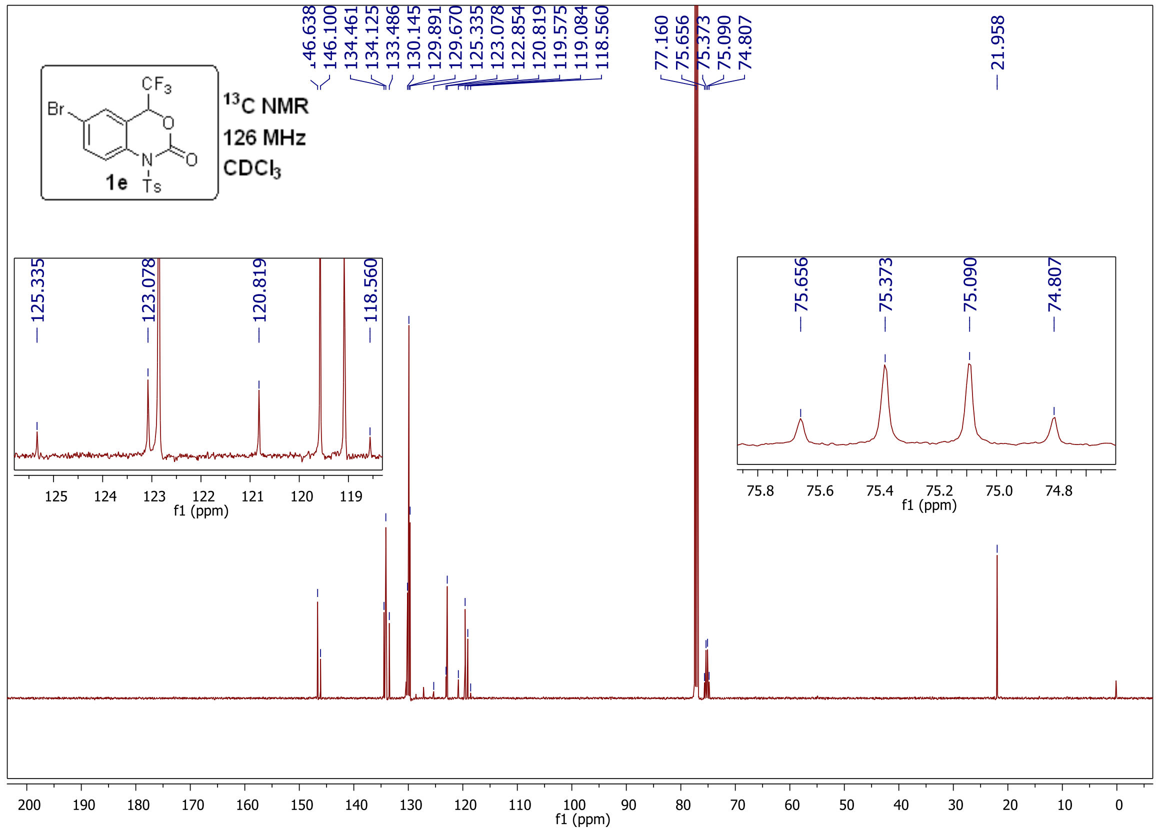

146.638
146.100
134.461
134.125
133.486
130.145
129.891
129.670
125.335
123.078
122.854
120.819
119.575
119.084
118.560
77.160
75.656
75.373
75.090
74.807
21.958
Br
CF3
O
N
O
1e
Ts
^{13}C NMR
126 MHz
$CDCl_3$
f1 (ppm)

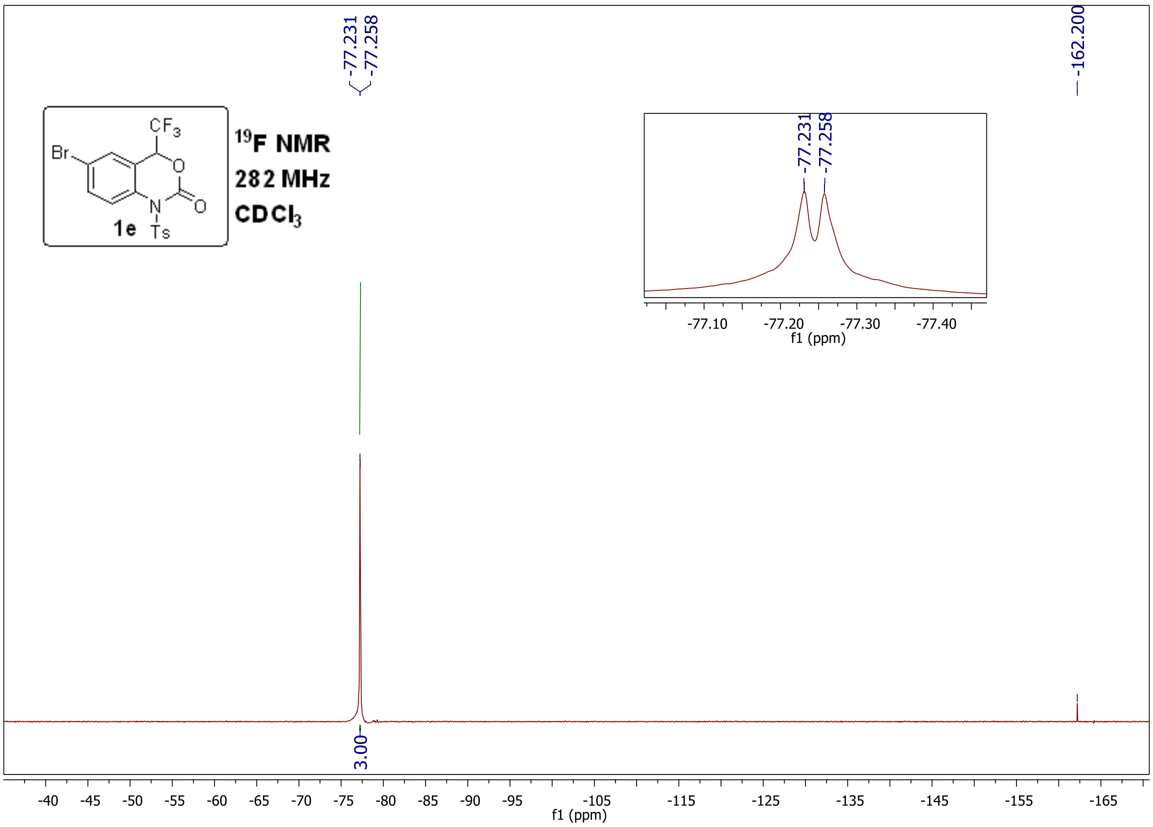
1e
19F NMR
282 MHz
CDCl3

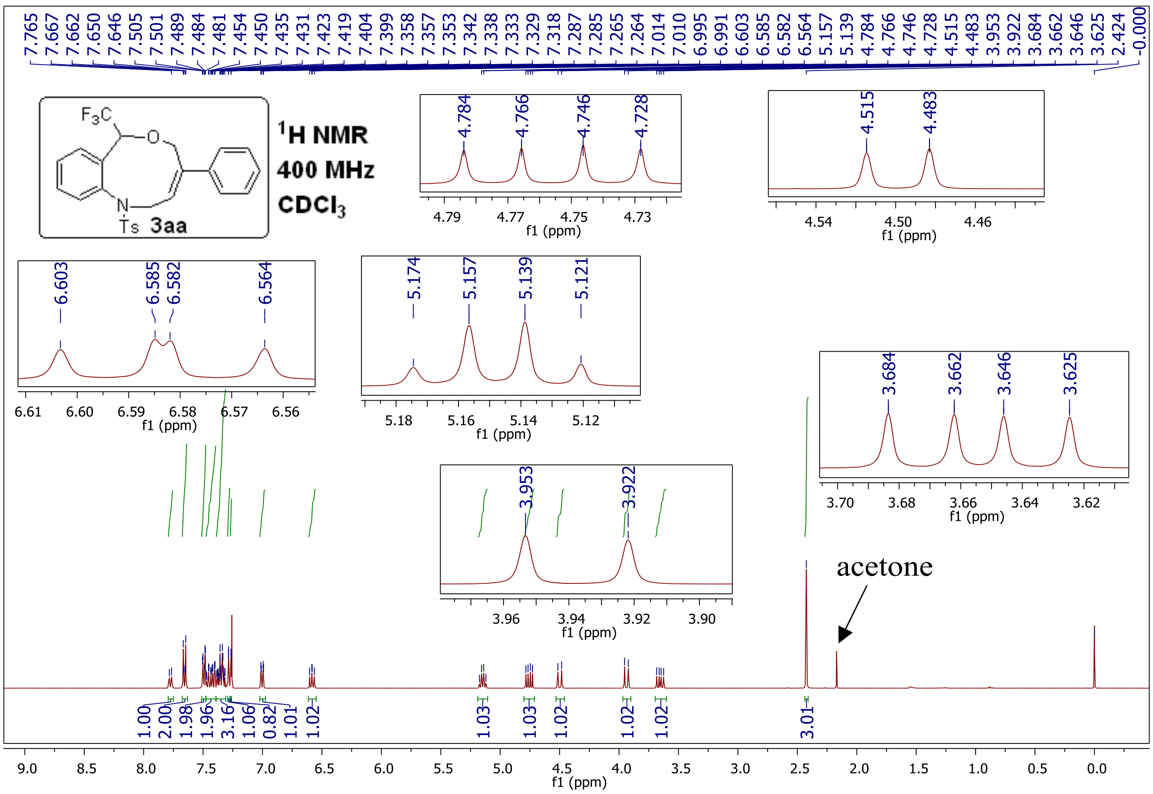
3aa
1H NMR
400 MHz
CDCl3
acetone

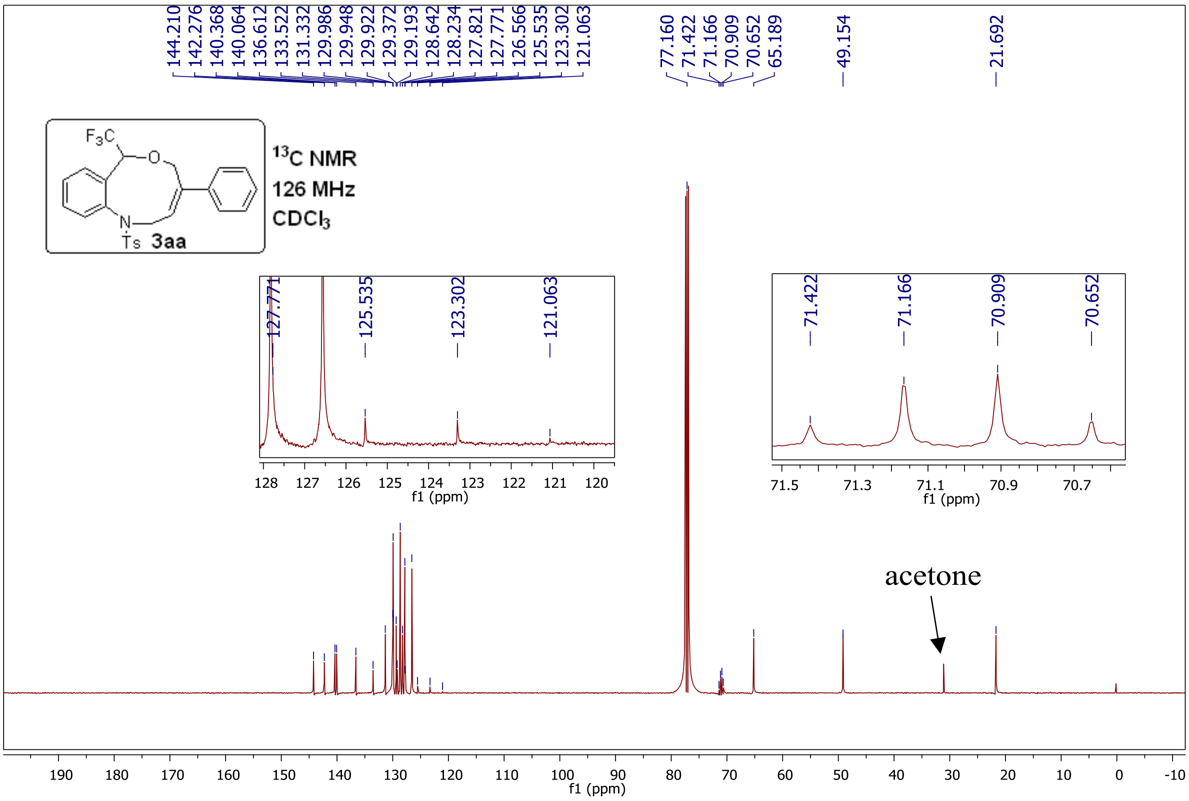
13C NMR
126 MHz
CDCl3
3aa
acetone
f1 (ppm)

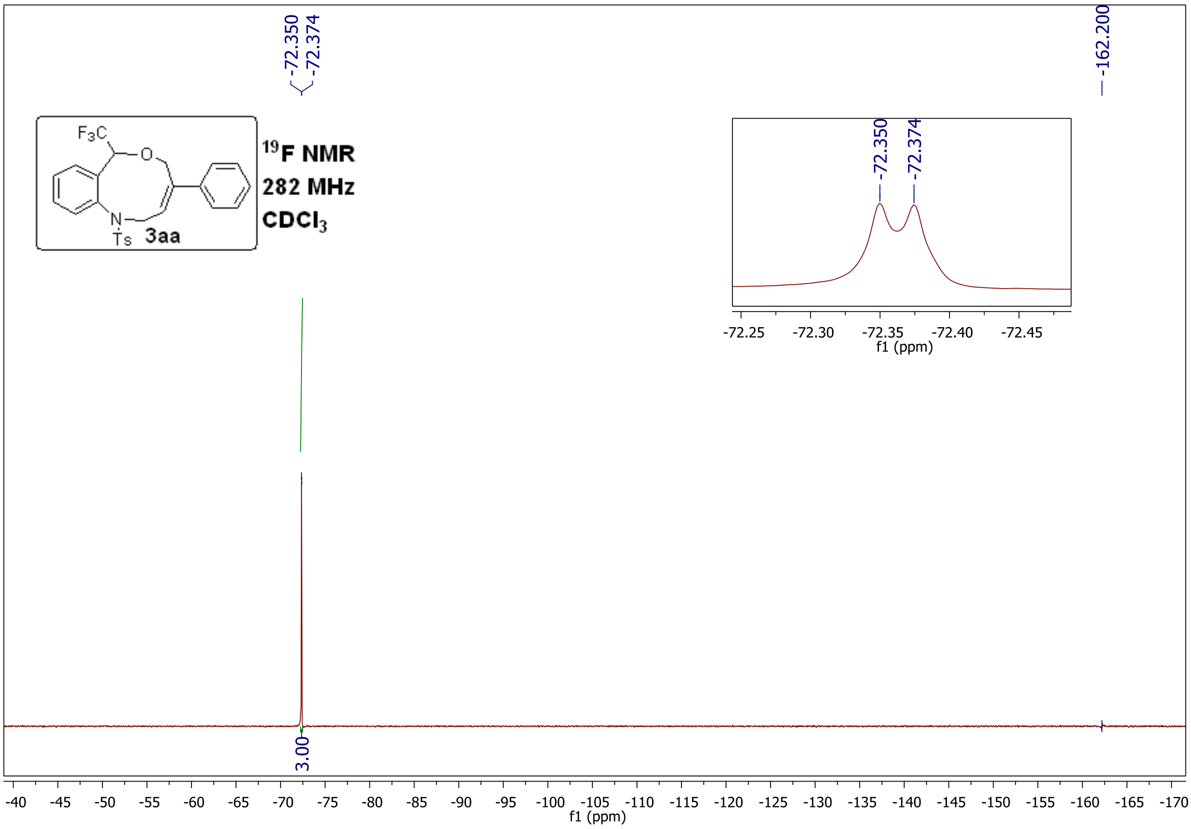
19F NMR
282 MHz
CDCl3
3aa
f1 (ppm)

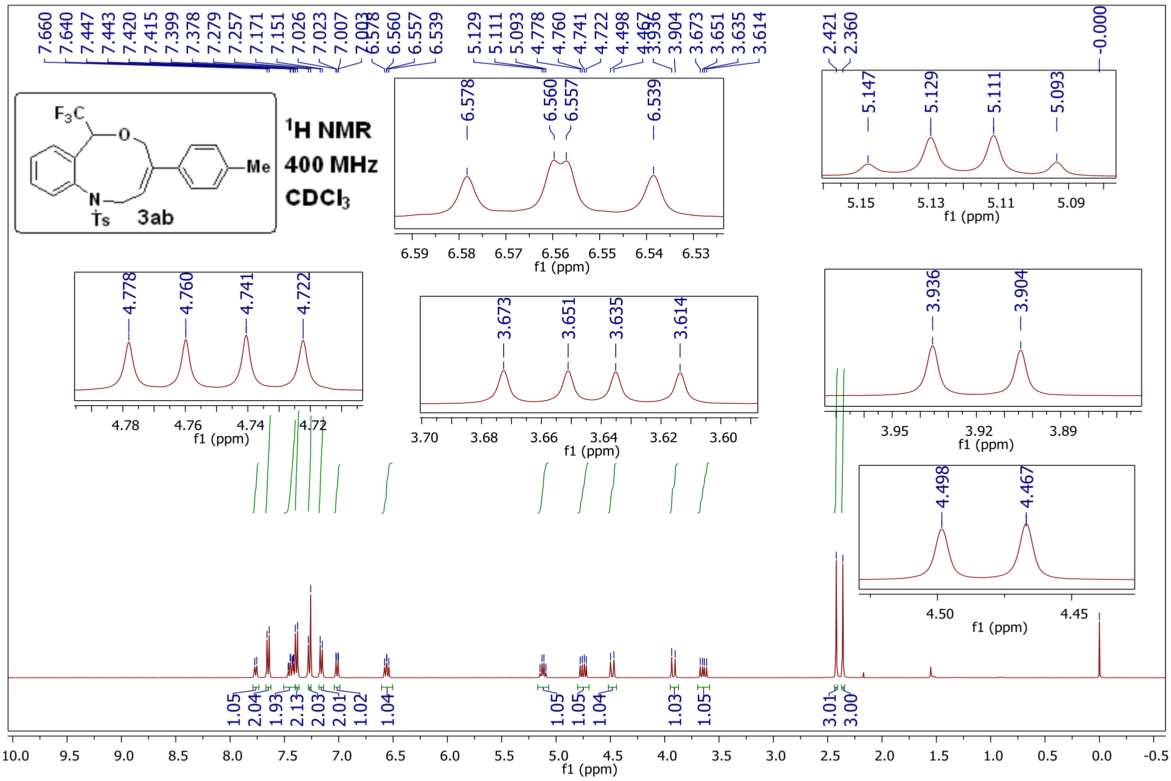

^{1}H NMR
400 MHz
CDCl$_3$
3ab

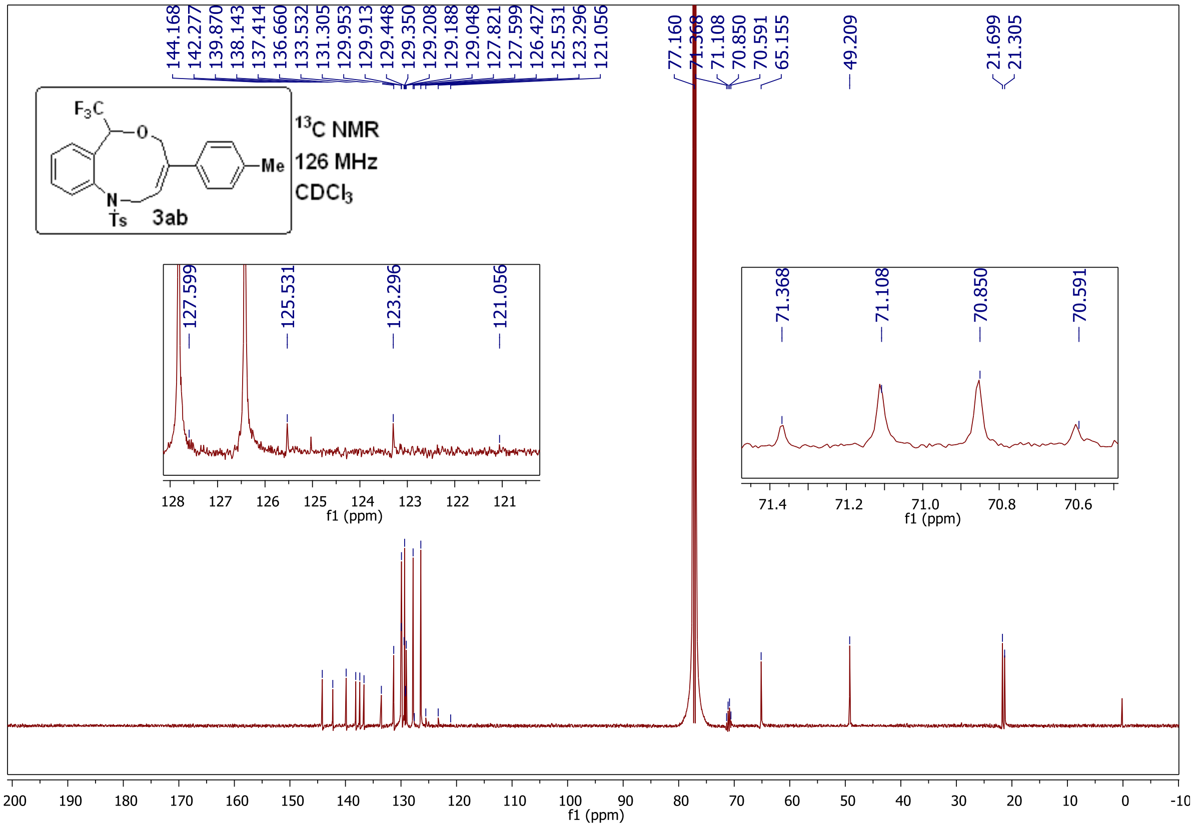

^{13}C NMR
126 MHz
CDCl$_3$
3ab

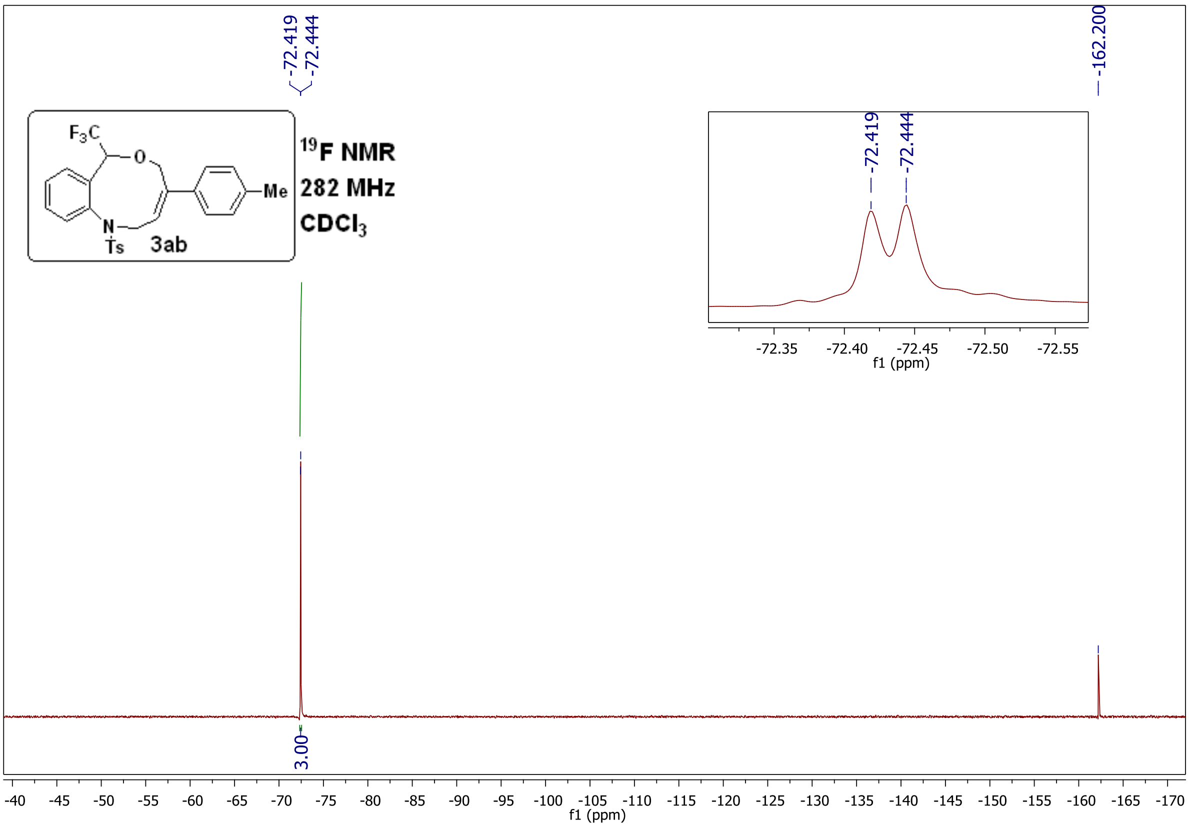
3ab
19F NMR
282 MHz
CDCl3
-72.419
-72.444
-162.200
3.00
f1 (ppm)

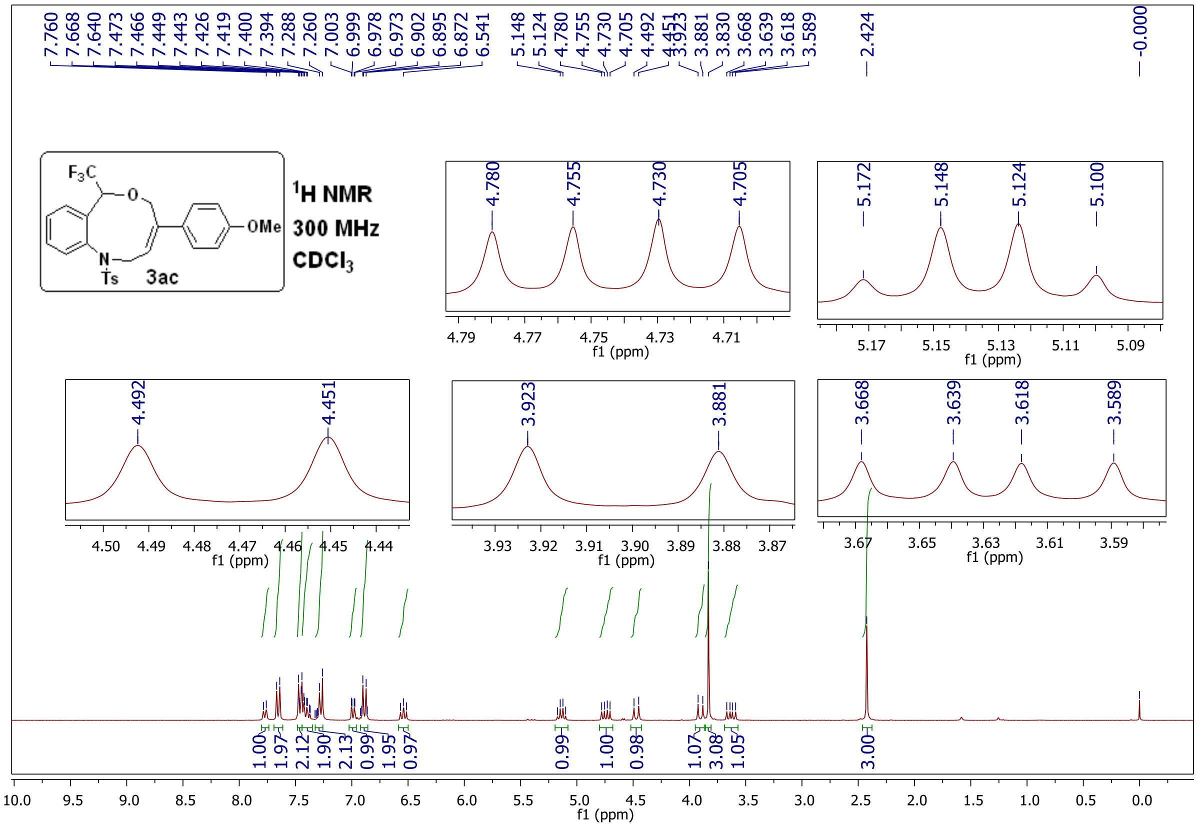
3ac
1H NMR
300 MHz
CDCl3
7.760 7.668 7.640 7.473 7.466 7.449 7.443 7.426 7.419 7.400 7.394 7.288 7.260 7.003 6.999 6.978 6.973 6.902 6.895 6.872 6.541
5.148 5.124 4.780 4.755 4.730 4.705 4.492 4.451 3.923 3.881 3.830 3.668 3.639 3.618 3.589
2.424
-0.000
5.172 5.148 5.124 5.100
1.00 1.97 2.12 1.90 2.13 0.99 1.95 0.97 0.99 1.00 0.98 1.07 3.08 1.05 3.00
f1 (ppm)

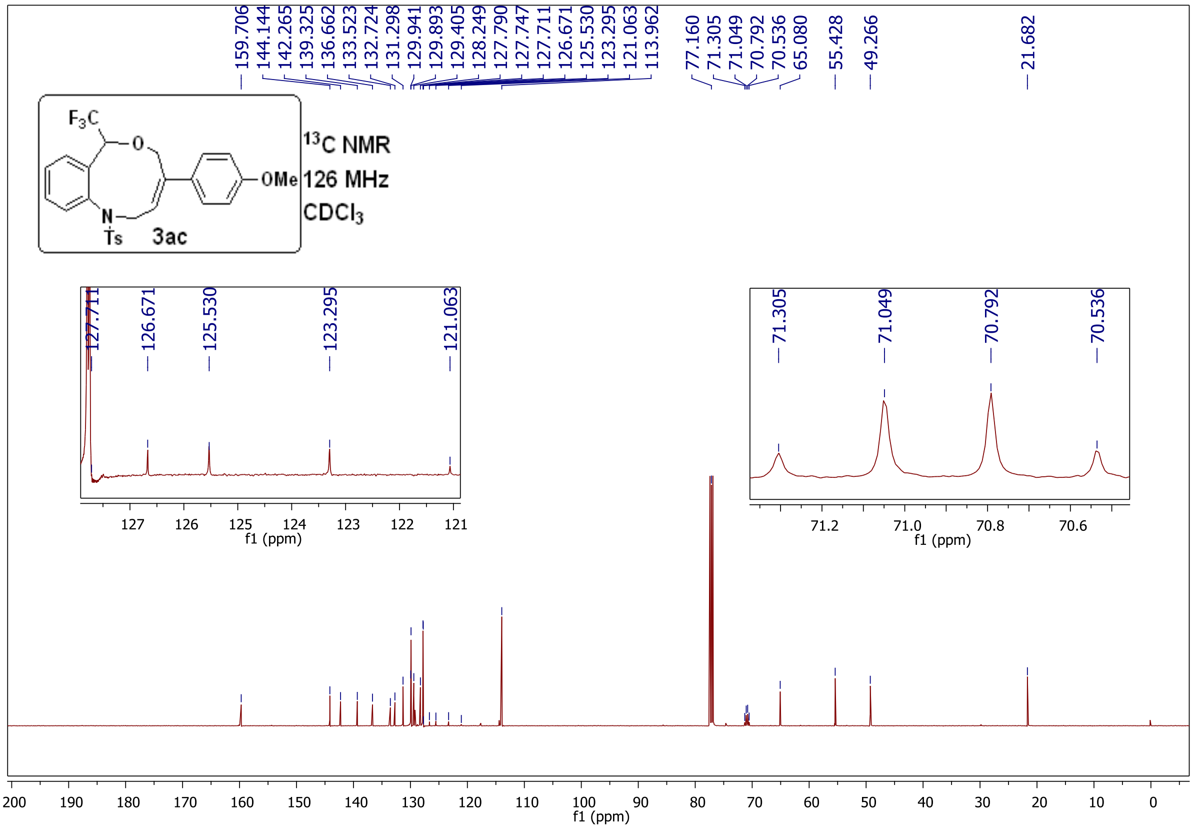

F3C
O
N
Ts
3ac
OMe
13C NMR
126 MHz
CDCl3
159.706
144.144
142.265
139.325
136.662
133.523
132.724
131.298
129.941
129.893
129.405
128.249
127.790
127.747
127.711
126.671
125.530
123.295
121.063
113.962
77.160
71.305
71.049
70.792
70.536
65.080
55.428
49.266
21.682
f1 (ppm)

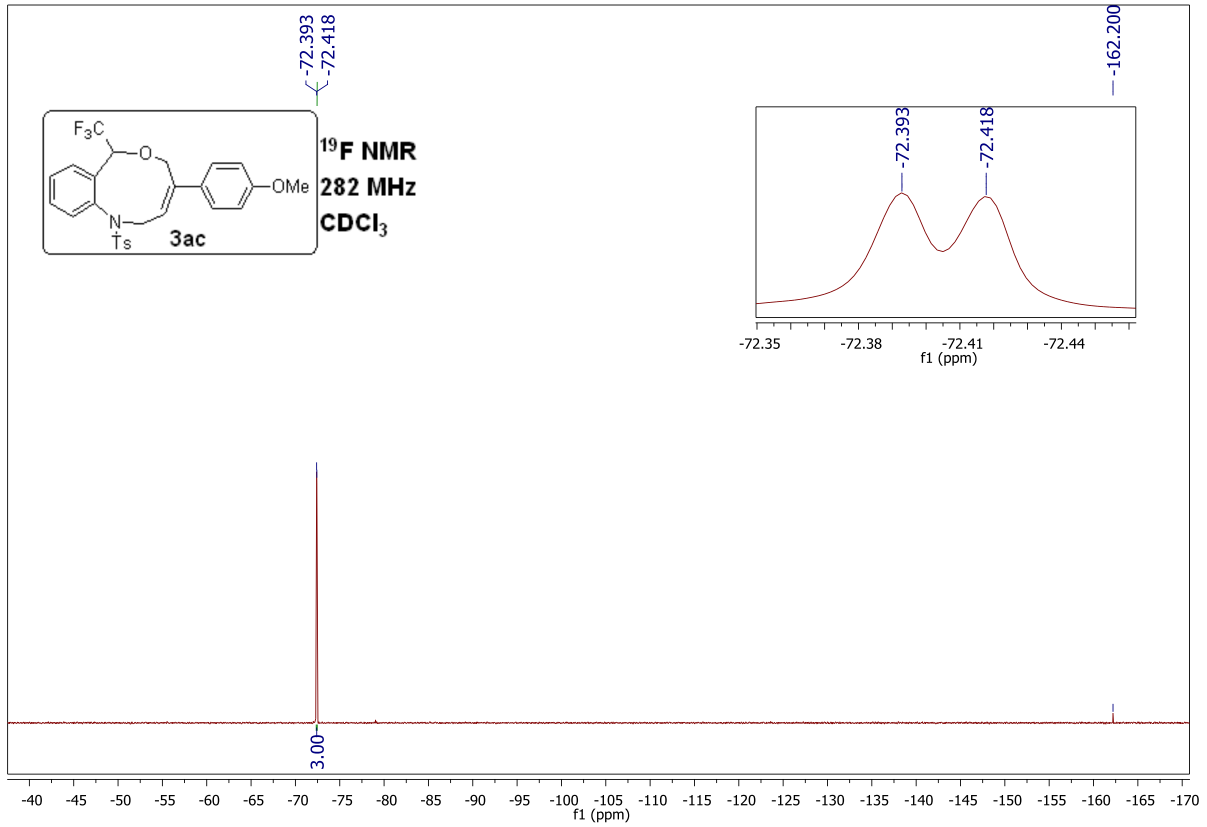

F3C
O
N
Ts
3ac
OMe
19F NMR
282 MHz
CDCl3
-72.393
-72.418
-162.200
3.00
f1 (ppm)

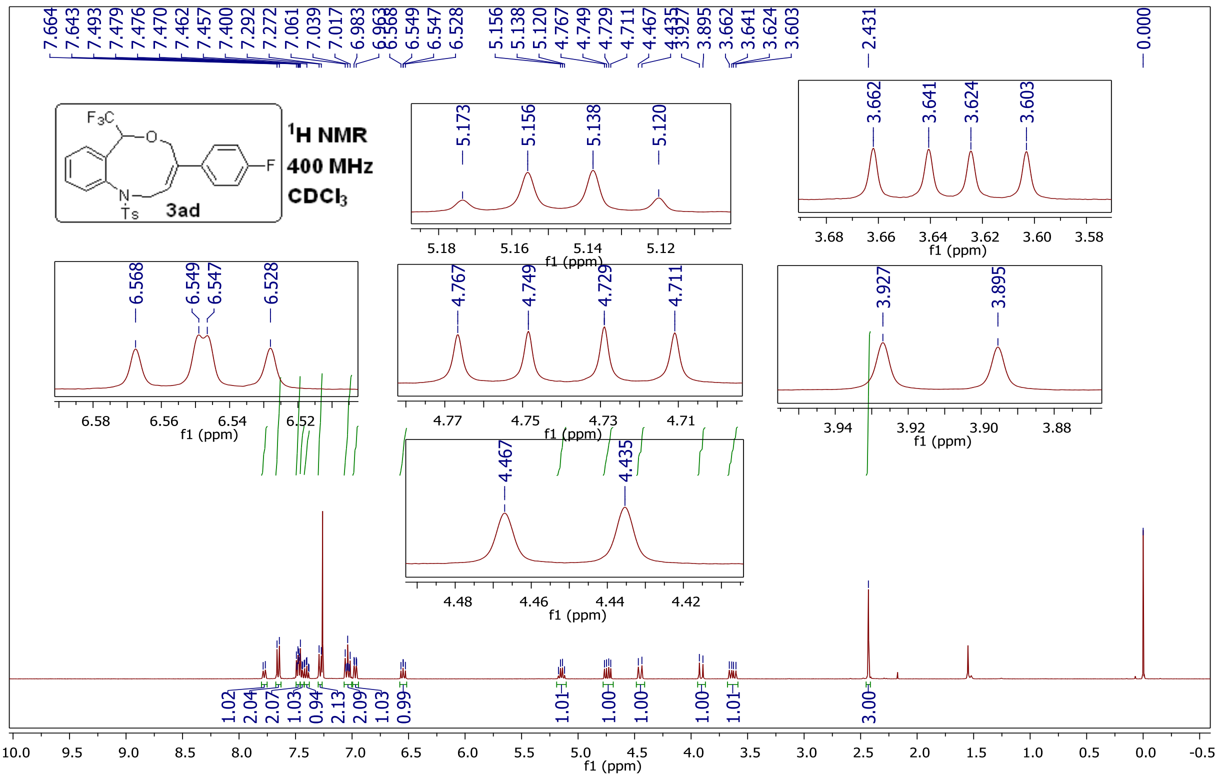

¹H NMR
400 MHz
CDCl₃
3ad
f1 (ppm)

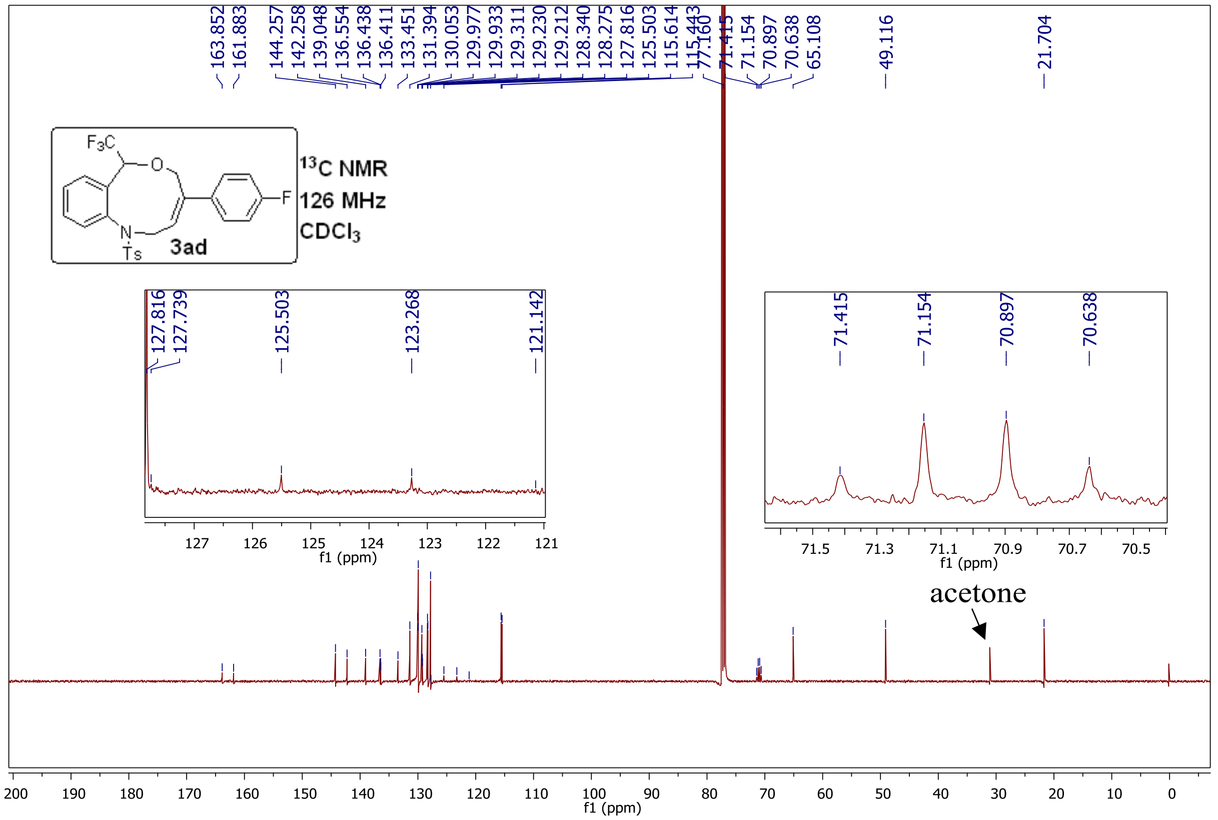

¹³C NMR
126 MHz
CDCl₃
3ad
acetone
f1 (ppm)

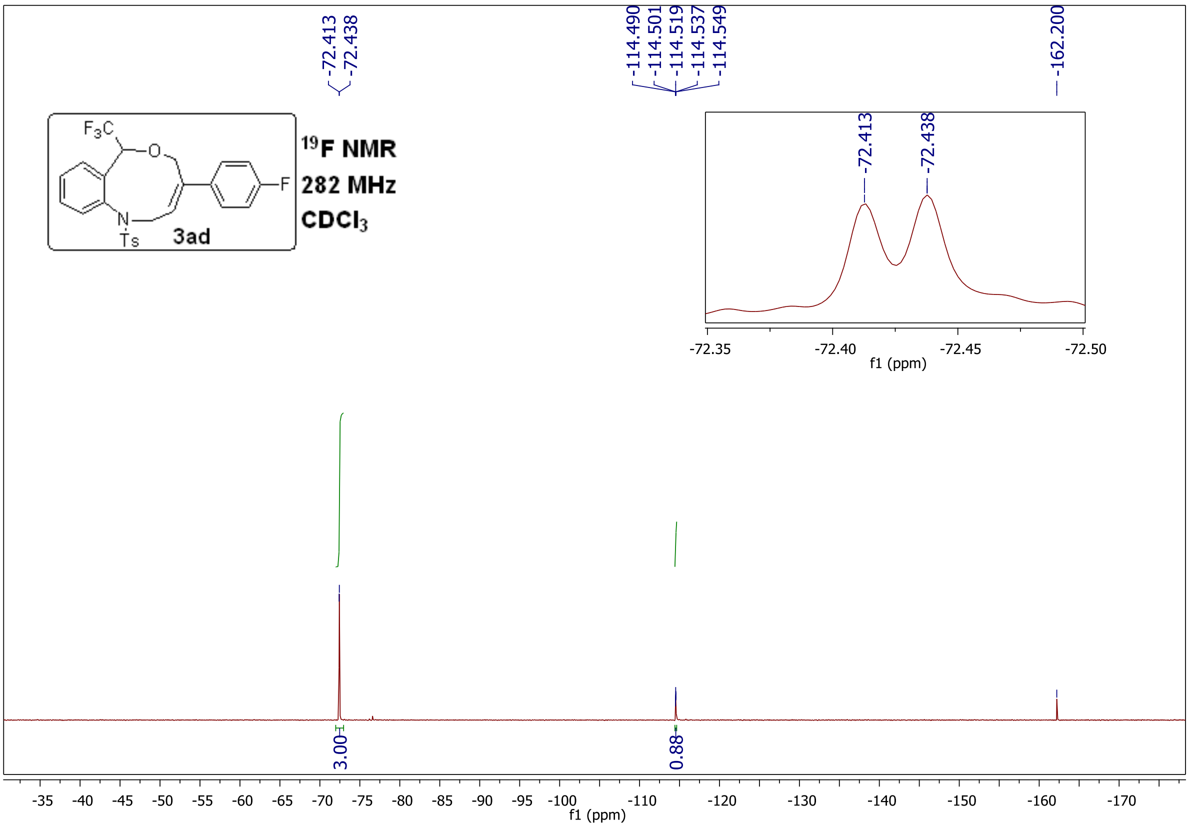

^{19}F NMR
282 MHz
CDCl$_3$
3ad
f1 (ppm)

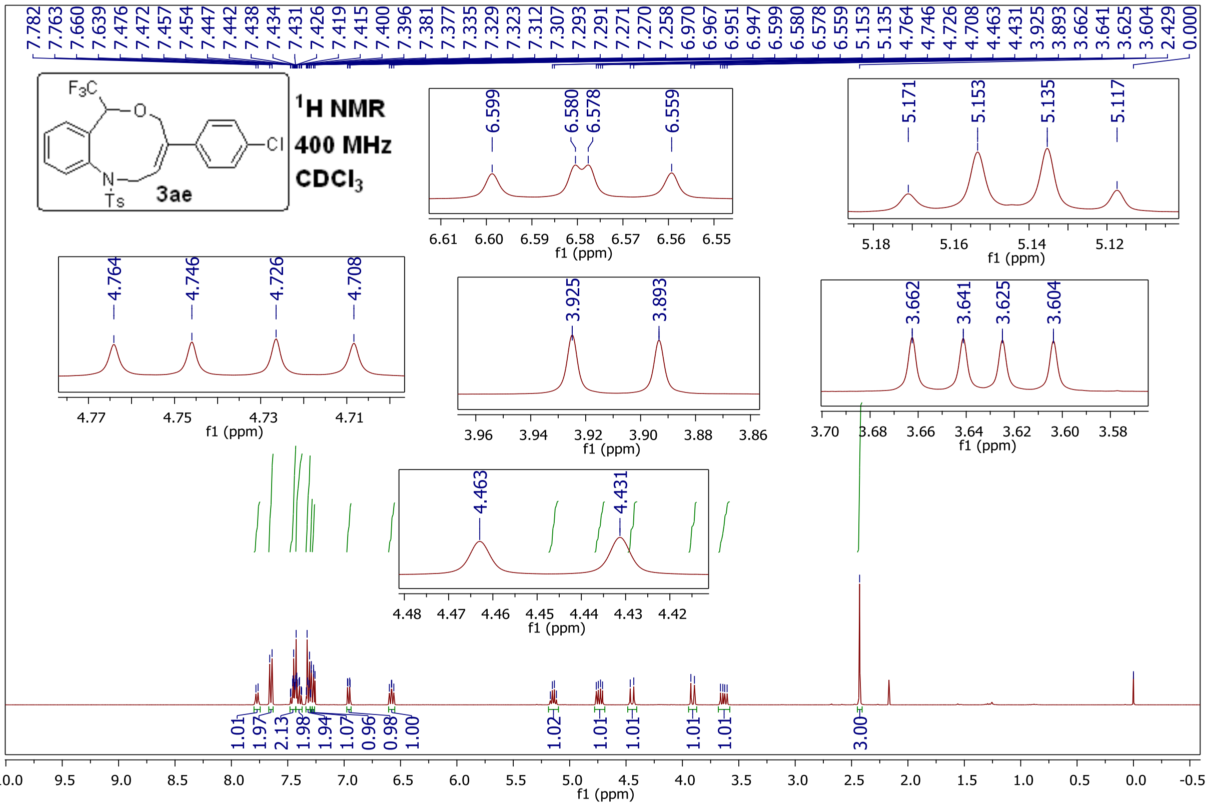

^{1}H NMR
400 MHz
CDCl$_3$
3ae
f1 (ppm)

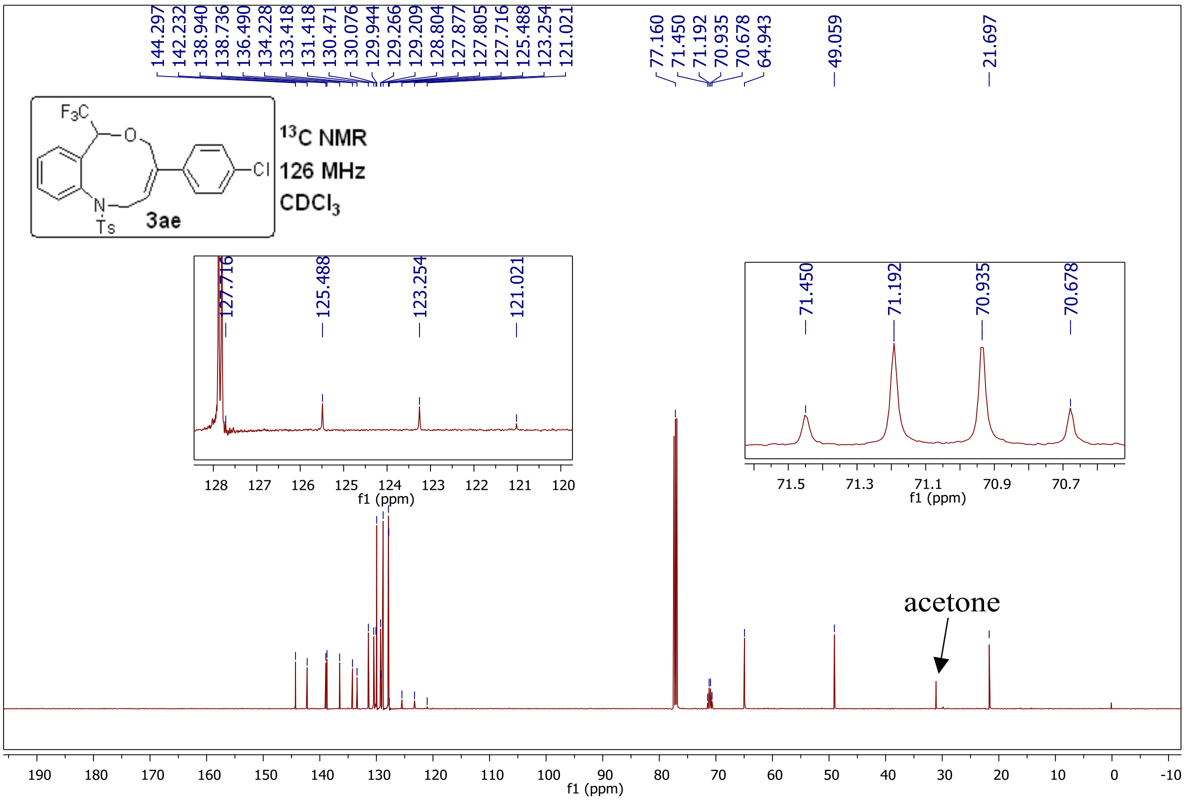

144.297
142.232
138.940
138.736
136.490
134.228
133.418
131.418
130.471
130.076
129.944
129.266
129.209
128.804
127.877
127.805
127.716
125.488
123.254
121.021
77.160
71.450
71.192
70.935
70.678
64.943
49.059
21.697
F3C
O
Cl
N
Ts
3ae
13C NMR
126 MHz
CDCl3
acetone
f1 (ppm)

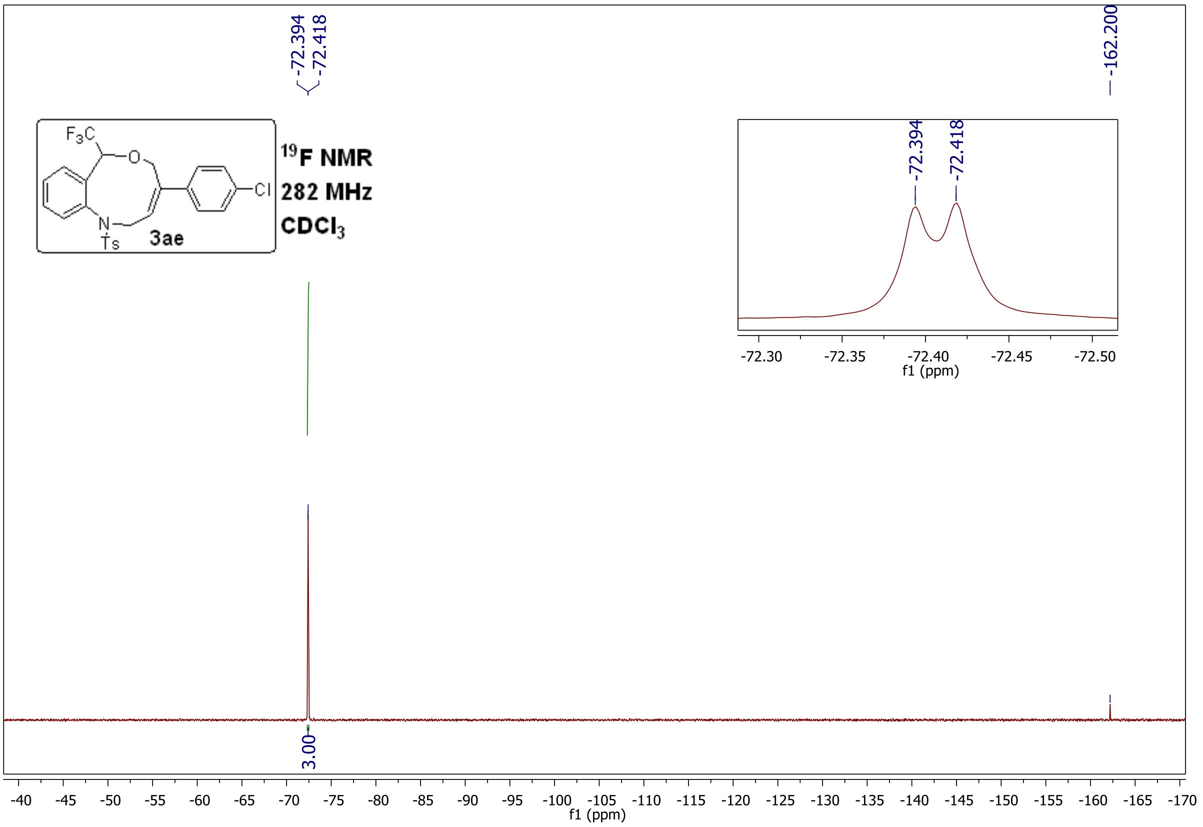

-72.394
-72.418
-162.200
F3C
O
Cl
N
Ts
3ae
19F NMR
282 MHz
CDCl3
3.00
f1 (ppm)

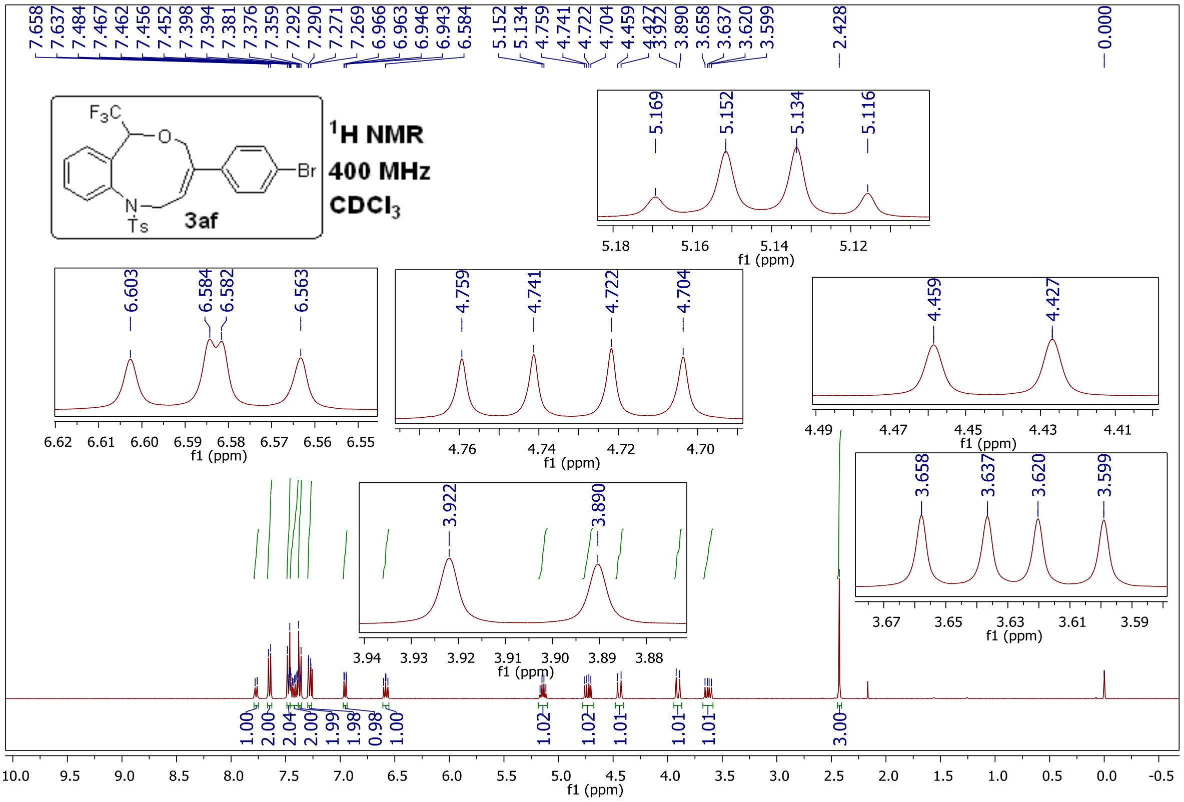
7.658 7.637 7.484 7.467 7.462 7.456 7.452 7.398 7.394 7.381 7.376 7.359 7.292 7.290 7.271 7.269 6.966 6.963 6.946 6.943 6.584 5.152 5.134 4.759 4.741 4.722 4.704 4.459 4.427 3.922 3.890 3.658 3.637 3.620 3.599 2.428 0.000
F3C
O
Br
N
Ts
3af
1H NMR
400 MHz
CDCl3
5.169 5.152 5.134 5.116
6.603 6.584 6.582 6.563
4.759 4.741 4.722 4.704
4.459 4.427
3.922 3.890
3.658 3.637 3.620 3.599
1.00 2.00 2.04 2.00 1.99 1.98 0.98 1.00 1.02 1.02 1.01 1.01 1.01 3.00
f1 (ppm)

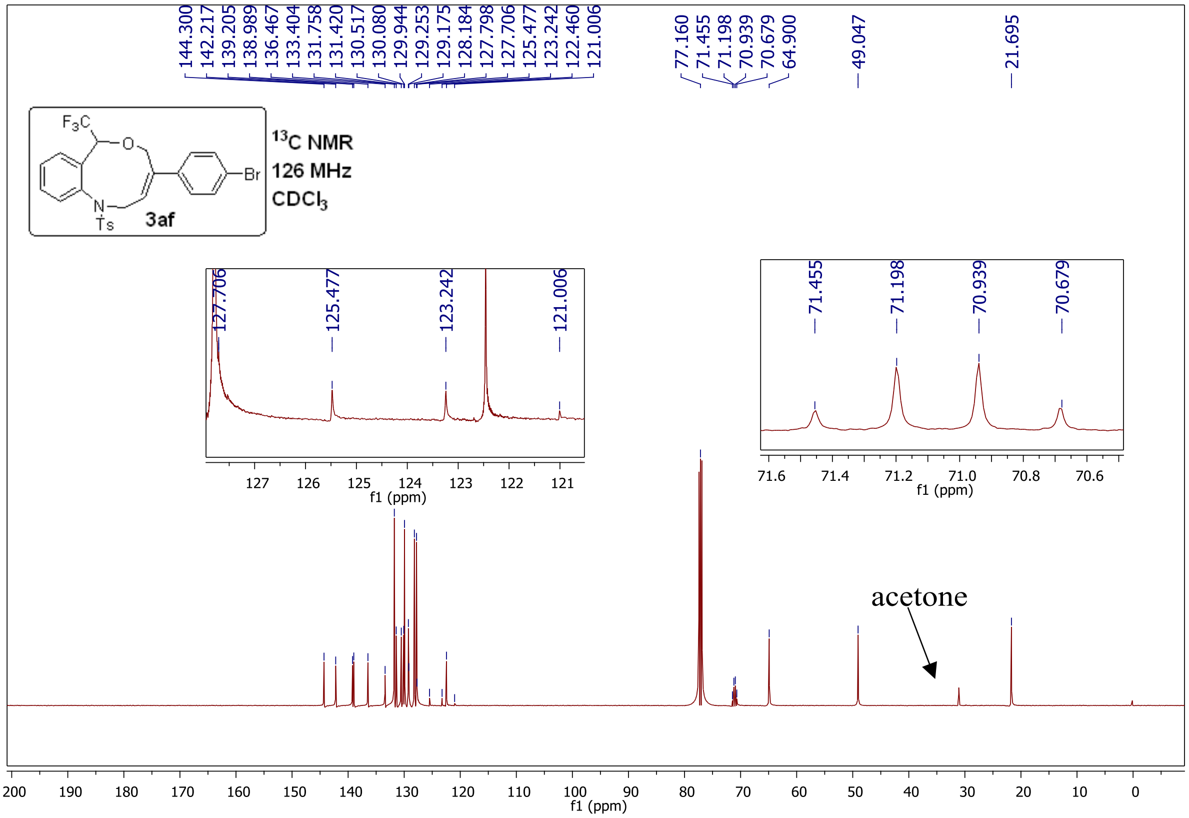
144.300 142.217 139.205 138.989 136.467 133.404 131.758 131.420 130.517 130.080 129.944 129.253 129.175 128.184 127.798 127.706 125.477 123.242 122.460 121.006 77.160 71.455 71.198 70.939 70.679 64.900 49.047 21.695
F3C
O
Br
N
Ts
3af
13C NMR
126 MHz
CDCl3
127.706 125.477 123.242 121.006
71.455 71.198 70.939 70.679
acetone
f1 (ppm)

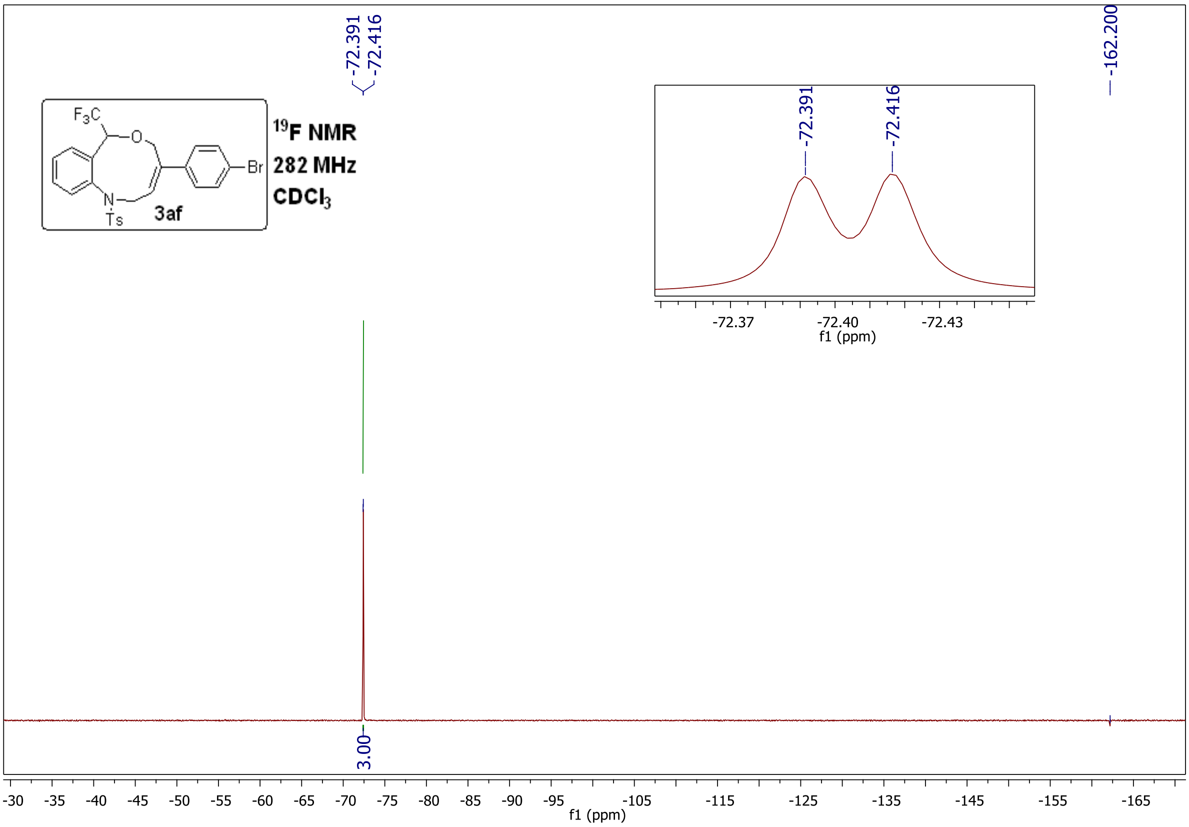

-72.391
-72.416
-162.200
F3C
O
Br
N
Ts
3af
19F NMR
282 MHz
CDCl3
3.00
f1 (ppm)

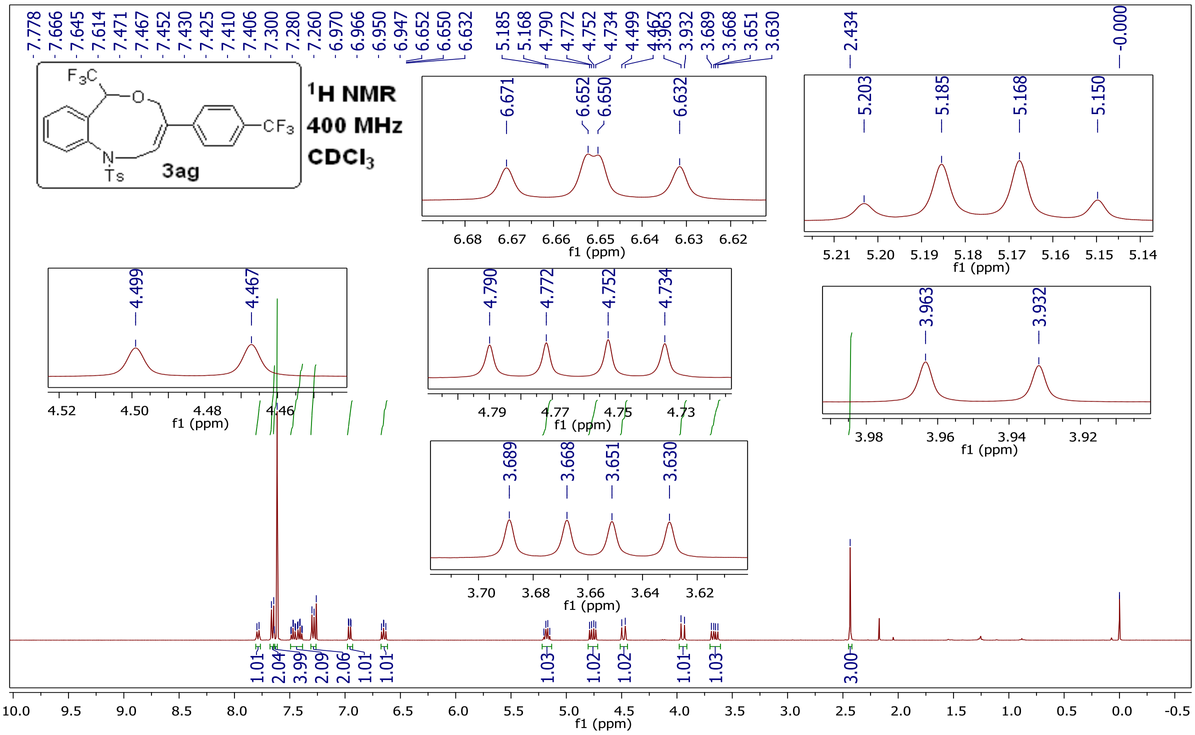

7.778
7.666
7.645
7.614
7.471
7.467
7.452
7.430
7.425
7.410
7.406
7.300
7.280
7.260
6.970
6.966
6.950
6.947
6.652
6.650
6.632
5.185
5.168
4.790
4.772
4.752
4.734
4.499
4.467
3.963
3.932
3.689
3.668
3.651
3.630
2.434
-0.000
F3C
O
CF3
N
Ts
3ag
1H NMR
400 MHz
CDCl3
1.01
2.04
3.99
2.09
2.06
1.01
1.01
1.03
1.02
1.02
1.01
1.03
3.00
f1 (ppm)

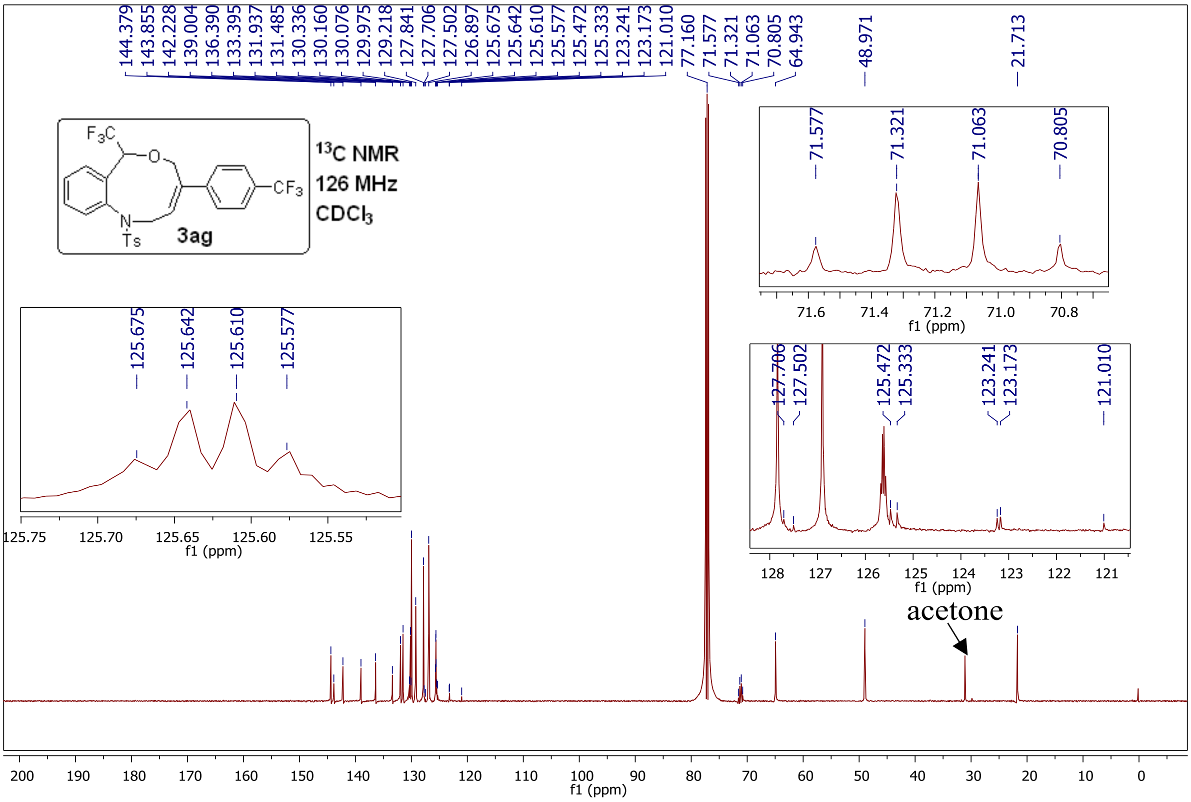

13C NMR
126 MHz
CDCl3
3ag
acetone
f1 (ppm)

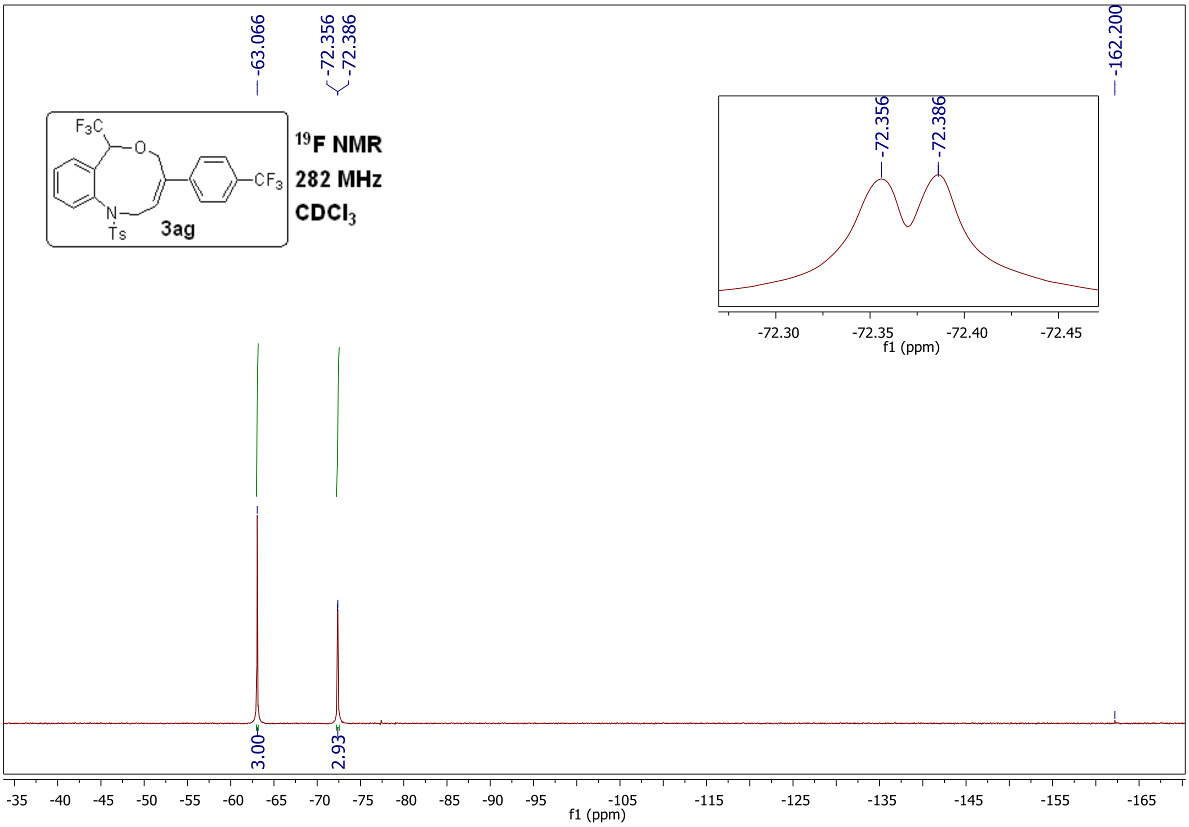

19F NMR
282 MHz
CDCl3
3ag
f1 (ppm)

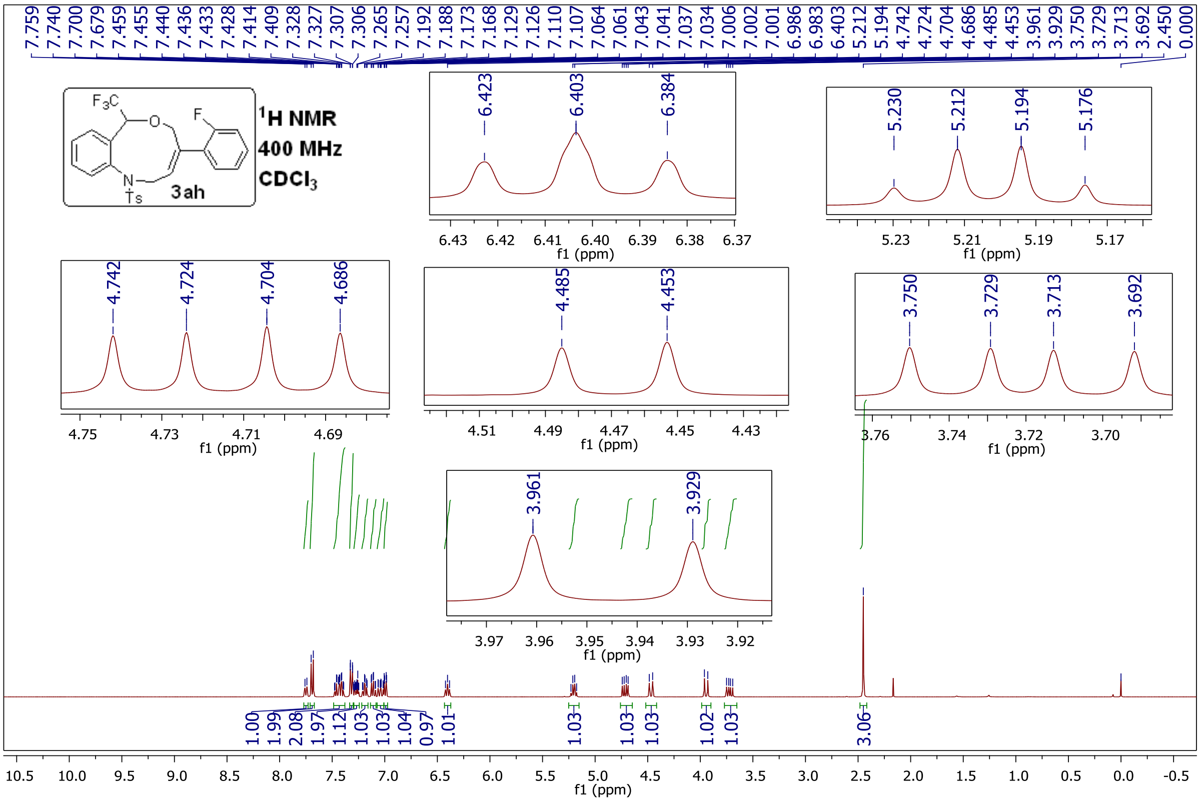
1H NMR
400 MHz
CDCl3
3ah
7.759 7.740 7.700 7.679 7.459 7.455 7.440 7.436 7.433 7.428 7.414 7.409 7.328 7.327 7.307 7.306 7.265 7.257 7.192 7.188 7.173 7.168 7.129 7.126 7.110 7.107 7.064 7.061 7.043 7.041 7.037 7.034 7.006 7.002 7.001 6.986 6.983 6.403 5.212 5.194 4.742 4.724 4.704 4.686 4.485 4.453 3.961 3.929 3.750 3.729 3.713 3.692 2.450 0.000
f1 (ppm)

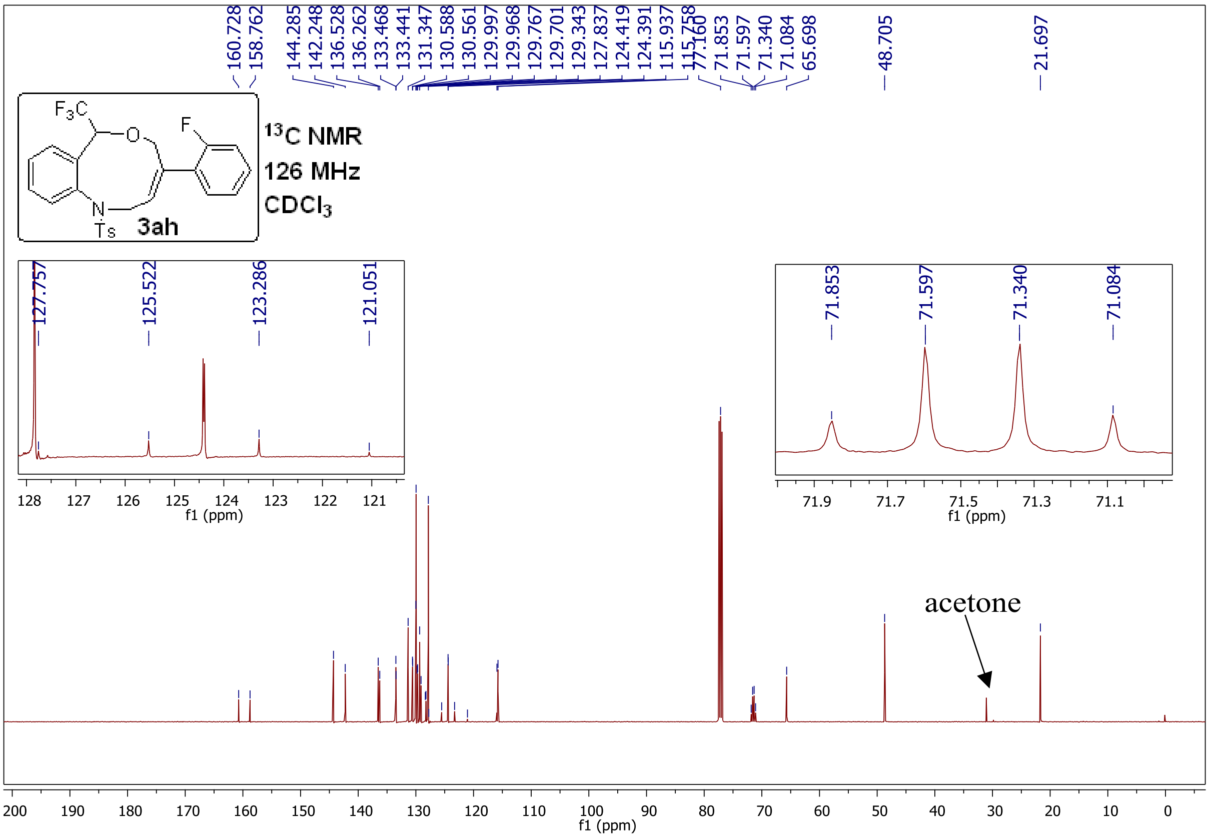
13C NMR
126 MHz
CDCl3
3ah
160.728 158.762 144.285 142.248 136.528 136.262 133.468 133.441 131.347 130.588 130.561 129.997 129.968 129.767 129.701 129.343 127.837 124.419 124.391 115.937 115.758 77.160 71.853 71.597 71.340 71.084 65.698 48.705 21.697
127.757 125.522 123.286 121.051
acetone
f1 (ppm)

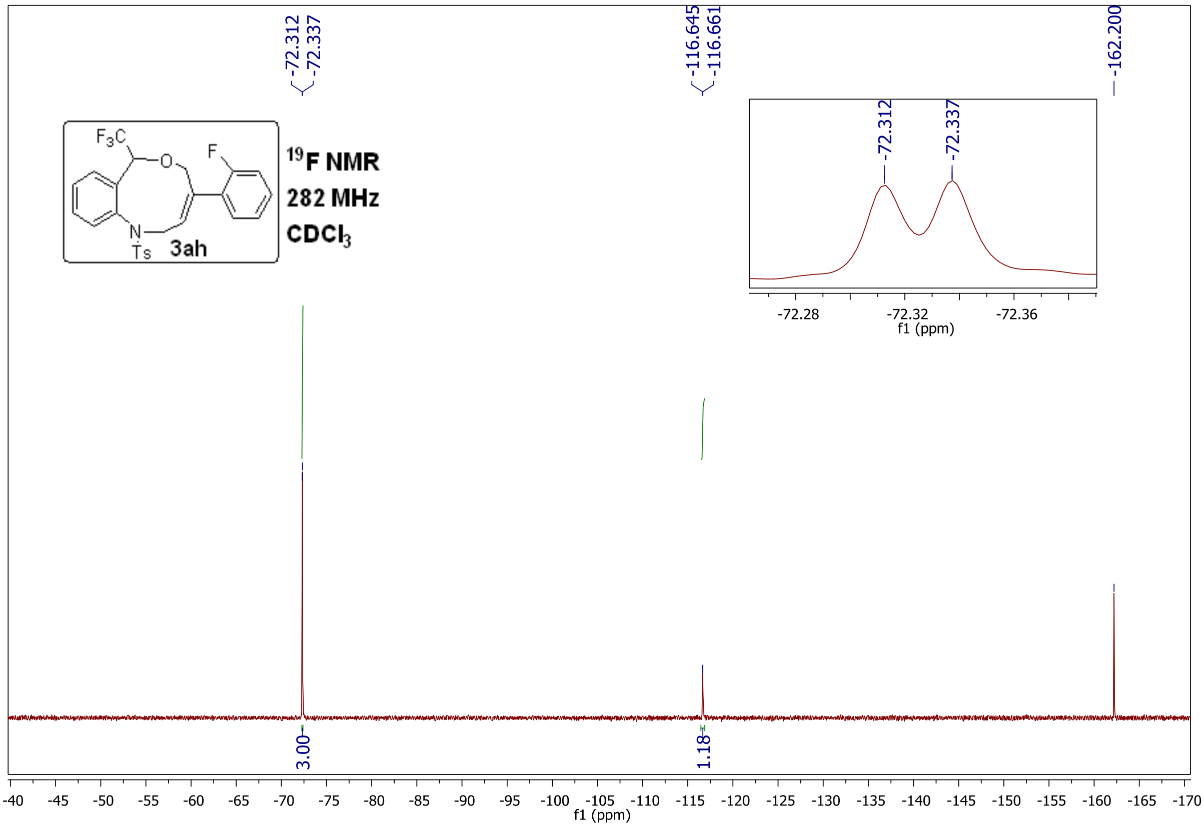

^{19}F NMR
282 MHz
CDCl$_3$
3ah
-72.312
-72.337
-116.645
-116.661
-162.200
3.00
1.18
f1 (ppm)

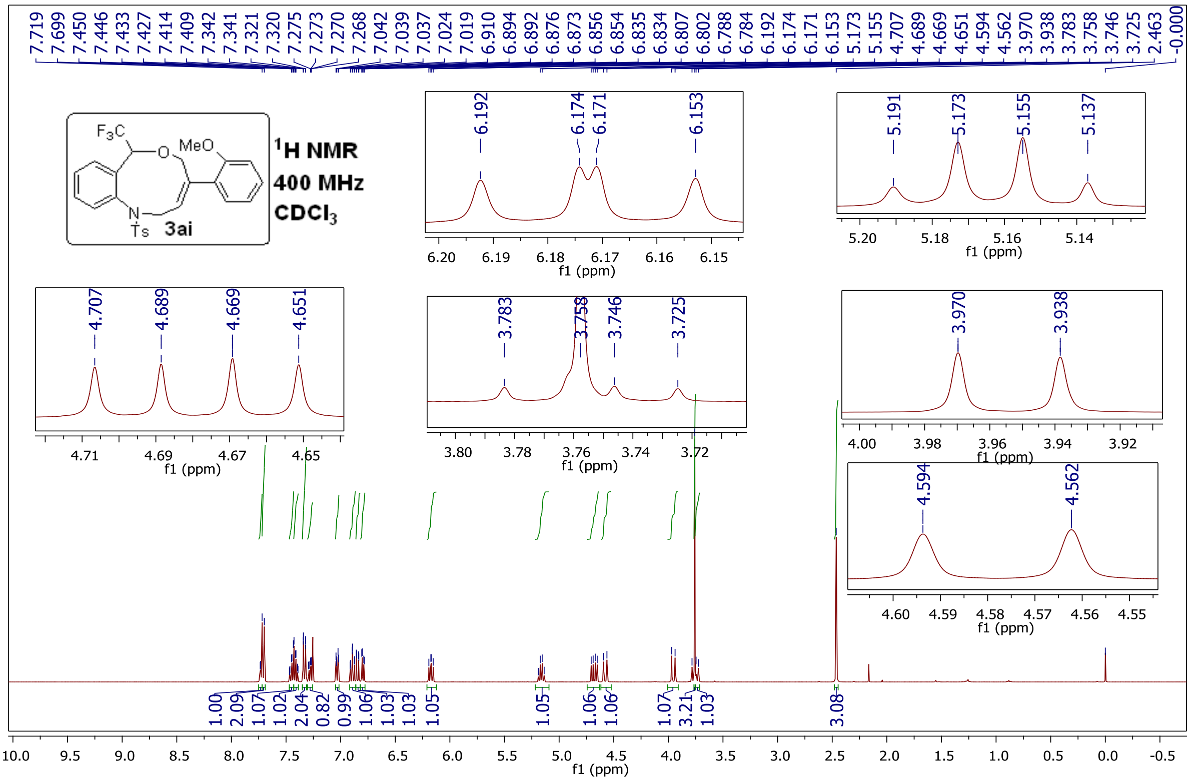

^{1}H NMR
400 MHz
CDCl$_3$
3ai
f1 (ppm)

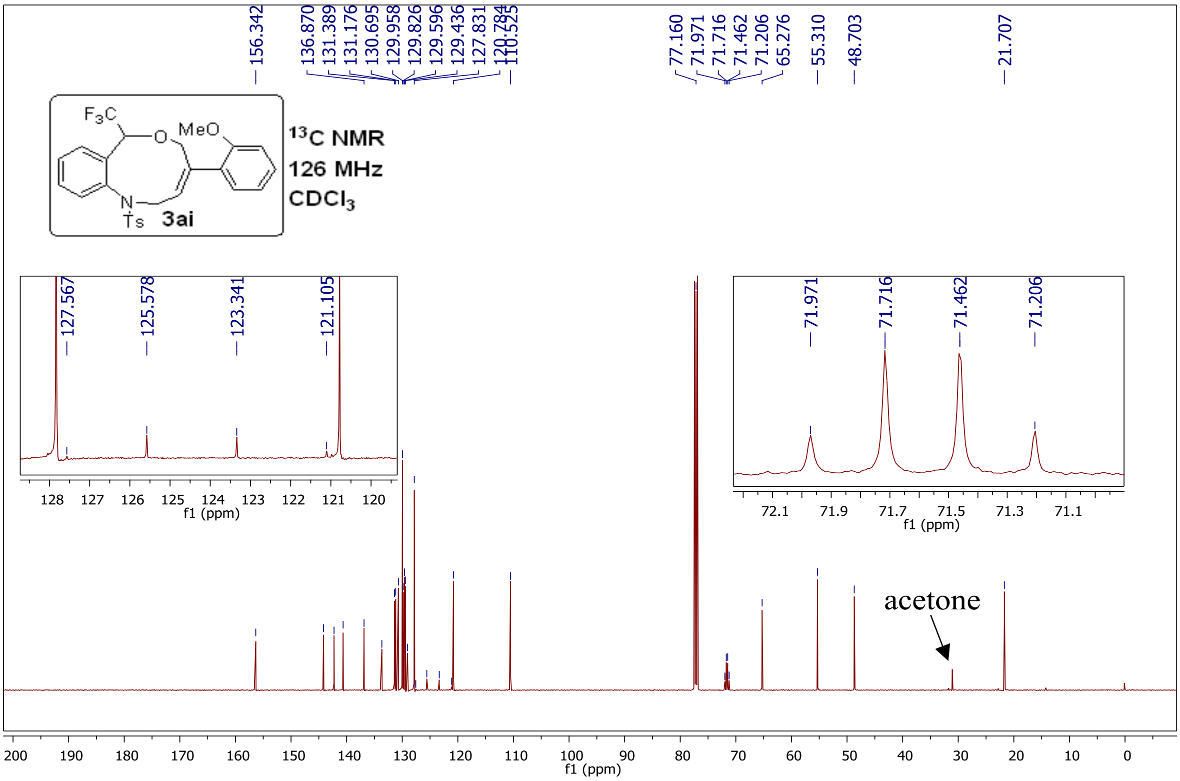
156.342
136.870
131.389
131.176
130.695
129.958
129.826
129.596
129.436
127.831
120.784
110.525
77.160
71.971
71.716
71.462
71.206
65.276
55.310
48.703
21.707
F3C
MeO
O
N
Ts
3ai
13C NMR
126 MHz
CDCl3
127.567
125.578
123.341
121.105
acetone
f1 (ppm)

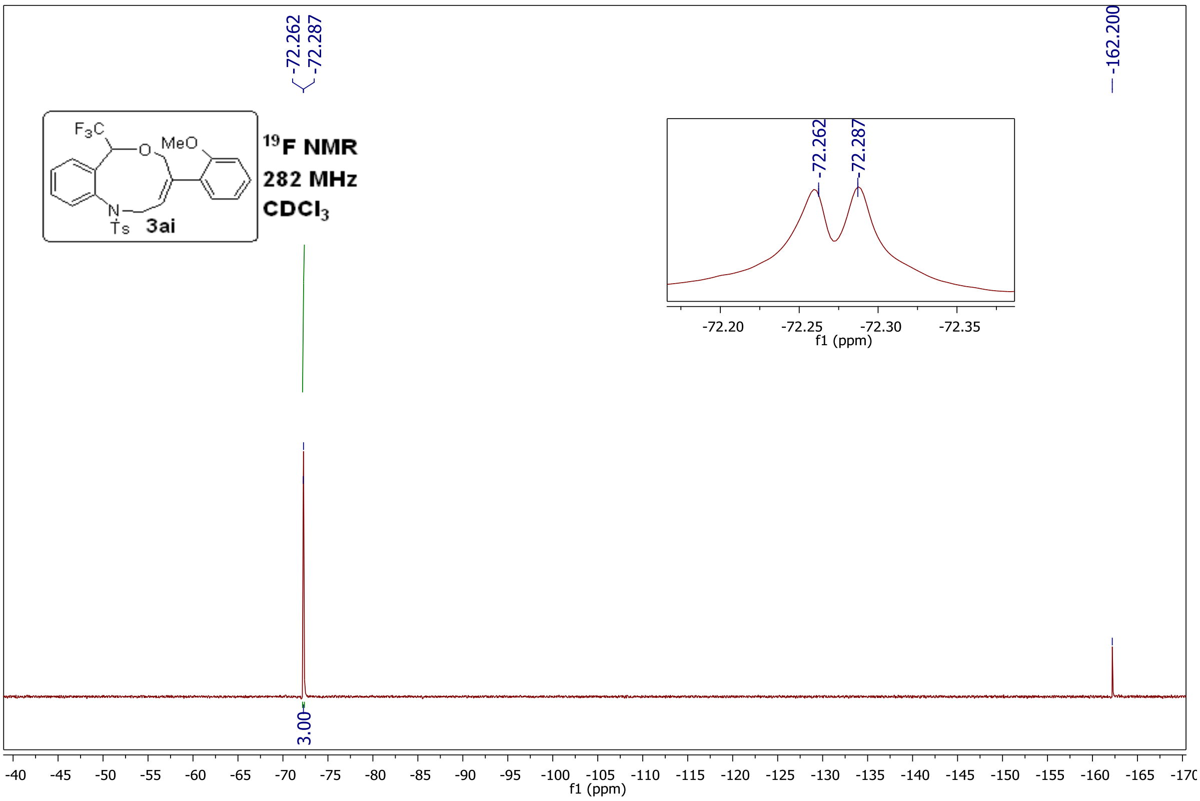
-72.262
-72.287
-162.200
F3C
MeO
O
N
Ts
3ai
19F NMR
282 MHz
CDCl3
3.00
f1 (ppm)

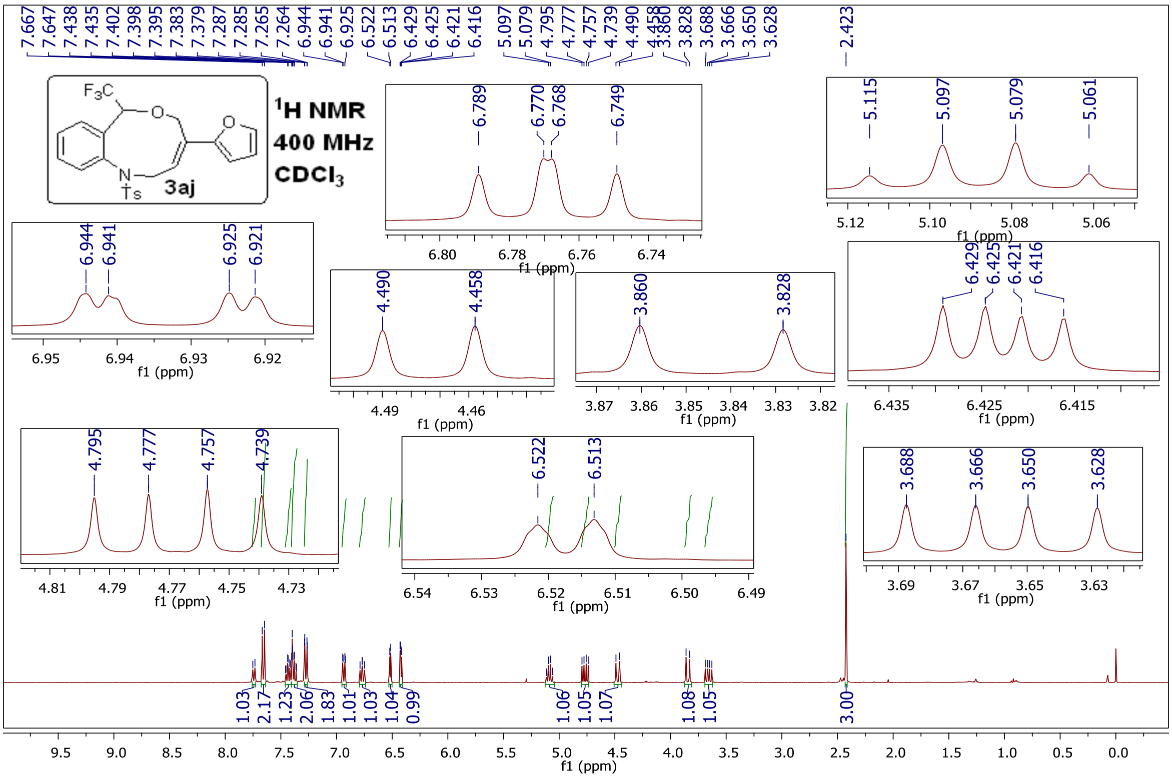

F3C
O
N
Ts
3aj
1H NMR
400 MHz
CDCl3
f1 (ppm)

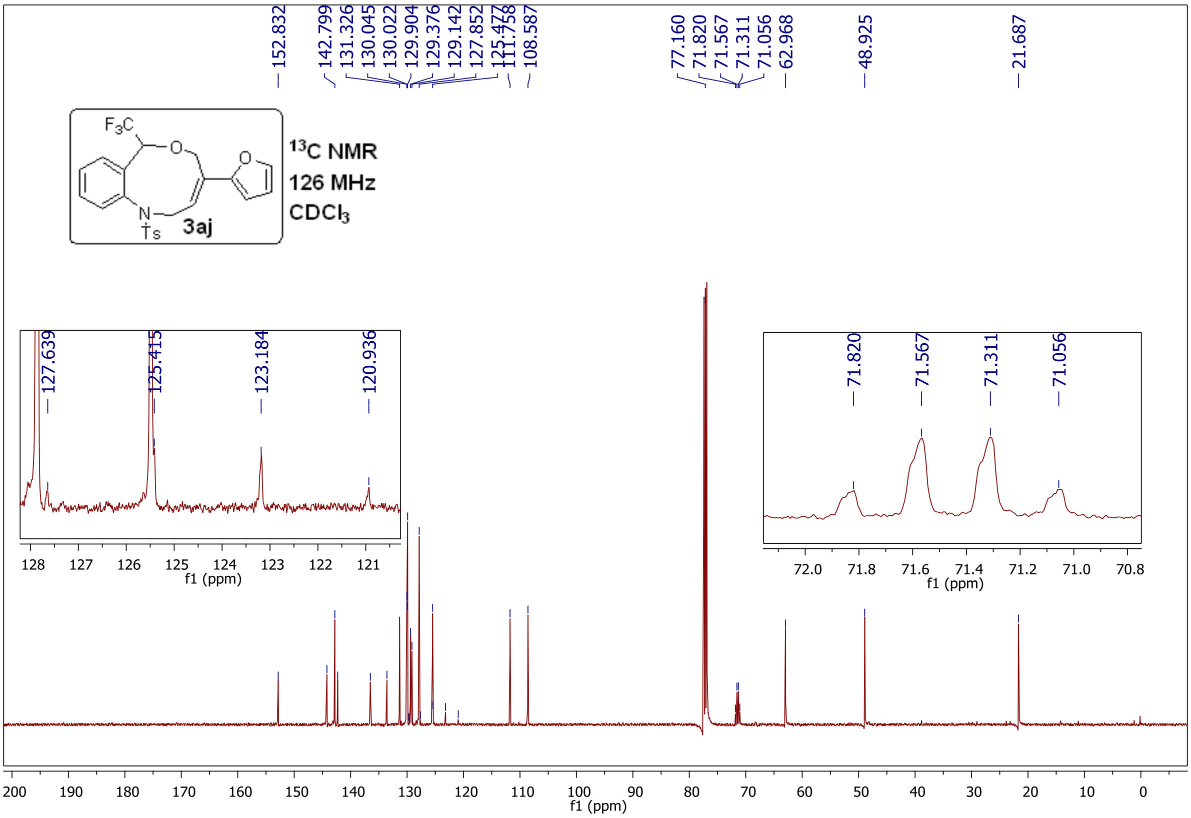

F3C
O
N
Ts
3aj
13C NMR
126 MHz
CDCl3
f1 (ppm)

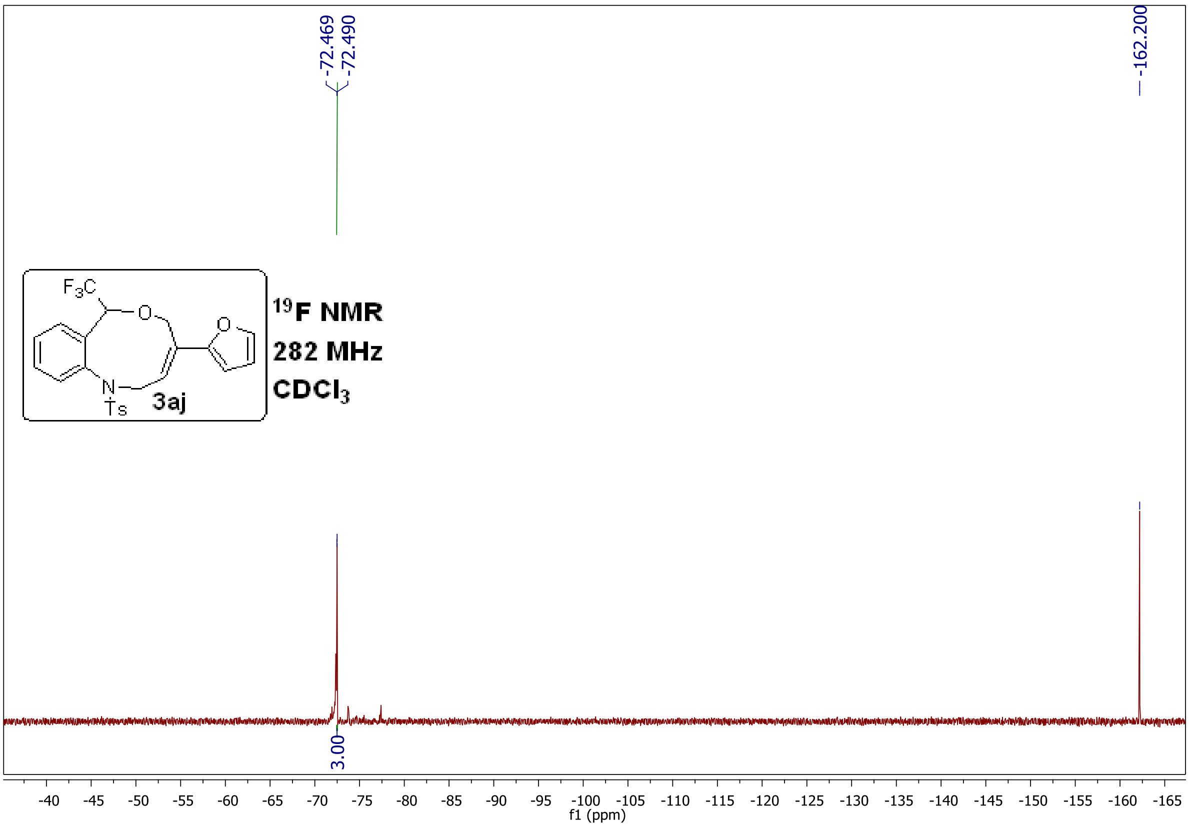

-72.469
-72.490
-162.200
F3C
3aj
19F NMR
282 MHz
CDCl3
3.00
f1 (ppm)

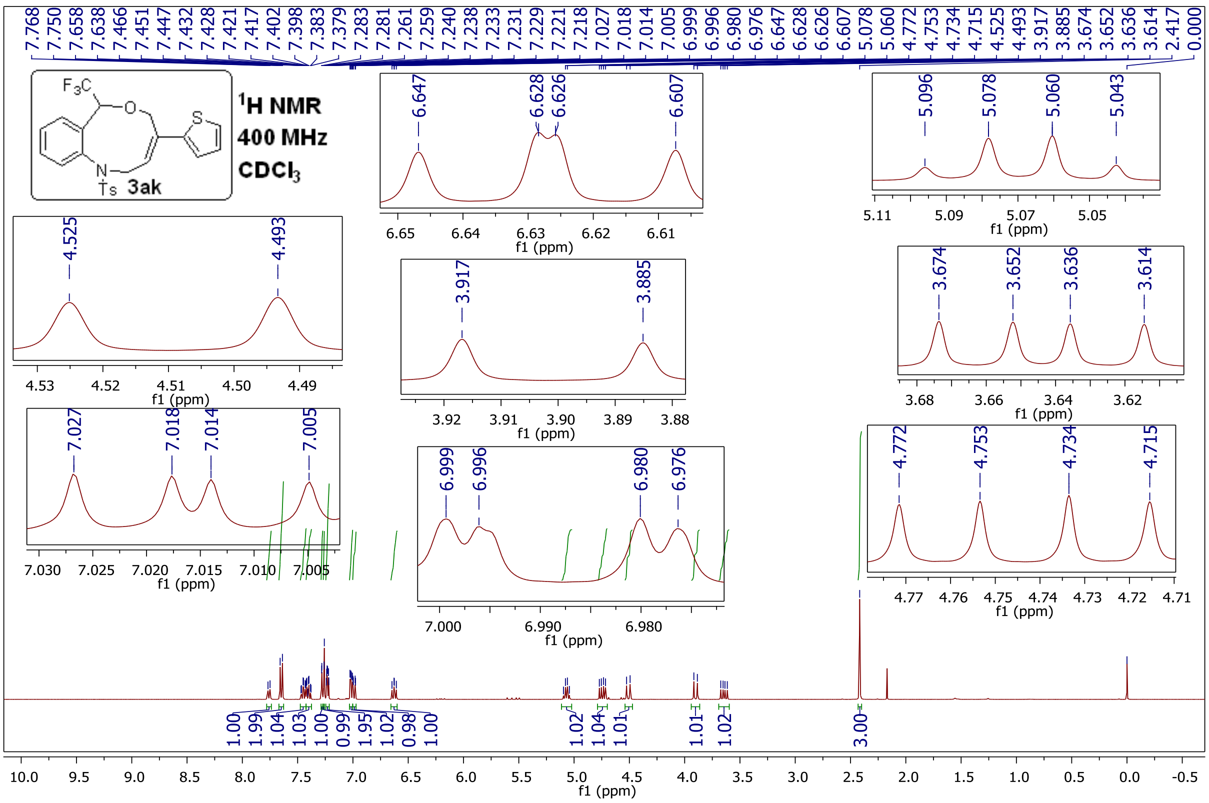

3ak
1H NMR
400 MHz
CDCl3
f1 (ppm)

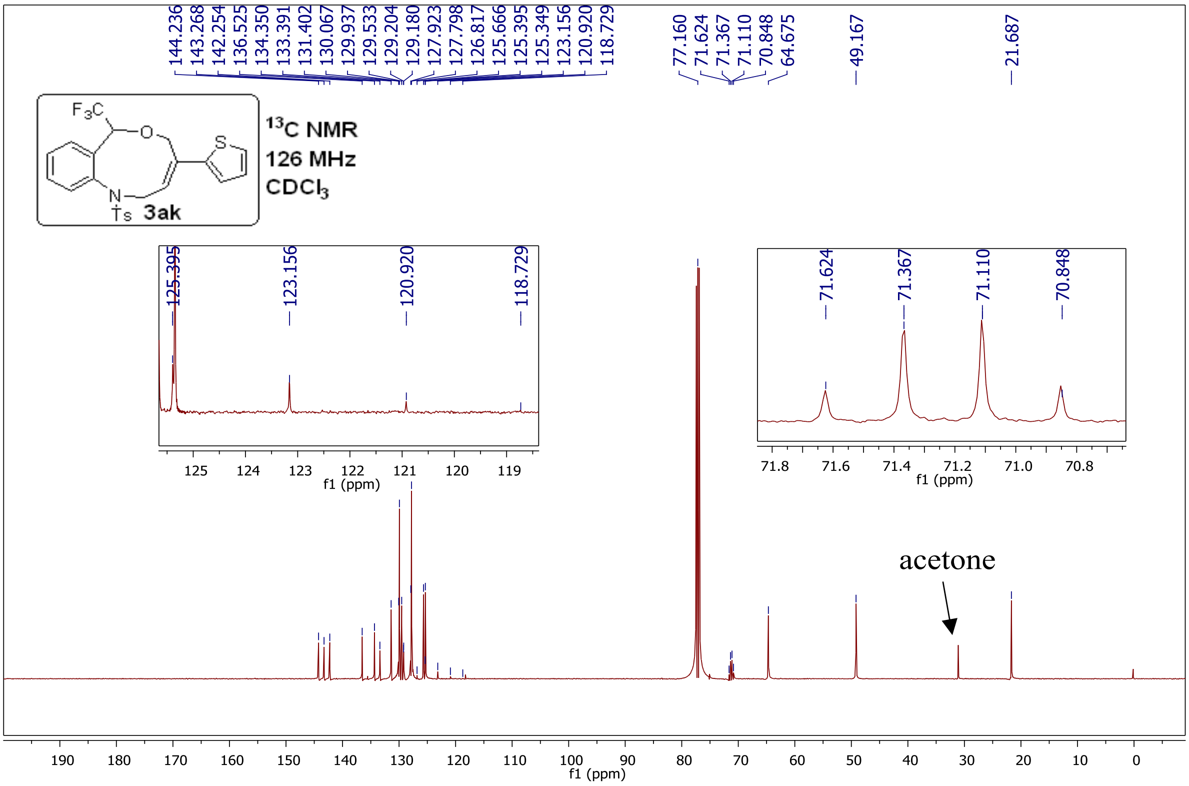
13C NMR
126 MHz
CDCl3
3ak
acetone
f1 (ppm)

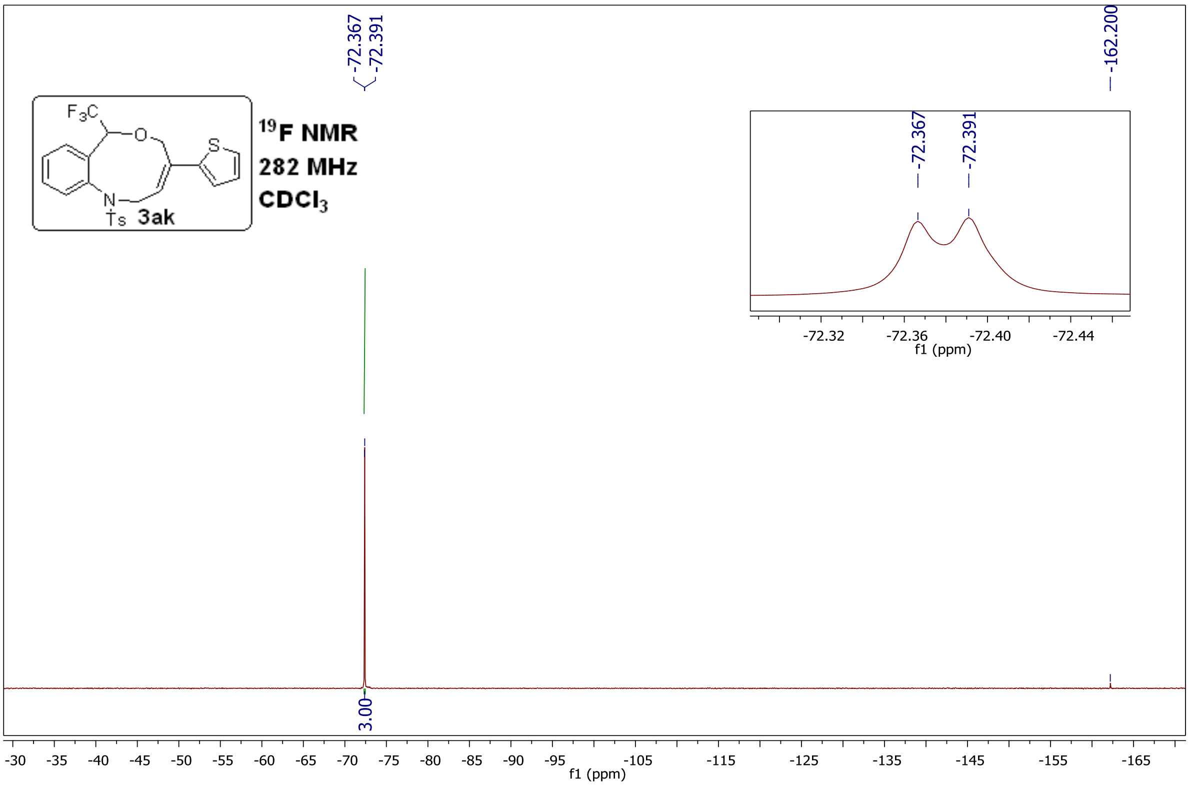
19F NMR
282 MHz
CDCl3
3ak
-72.367
-72.391
-162.200
3.00
f1 (ppm)

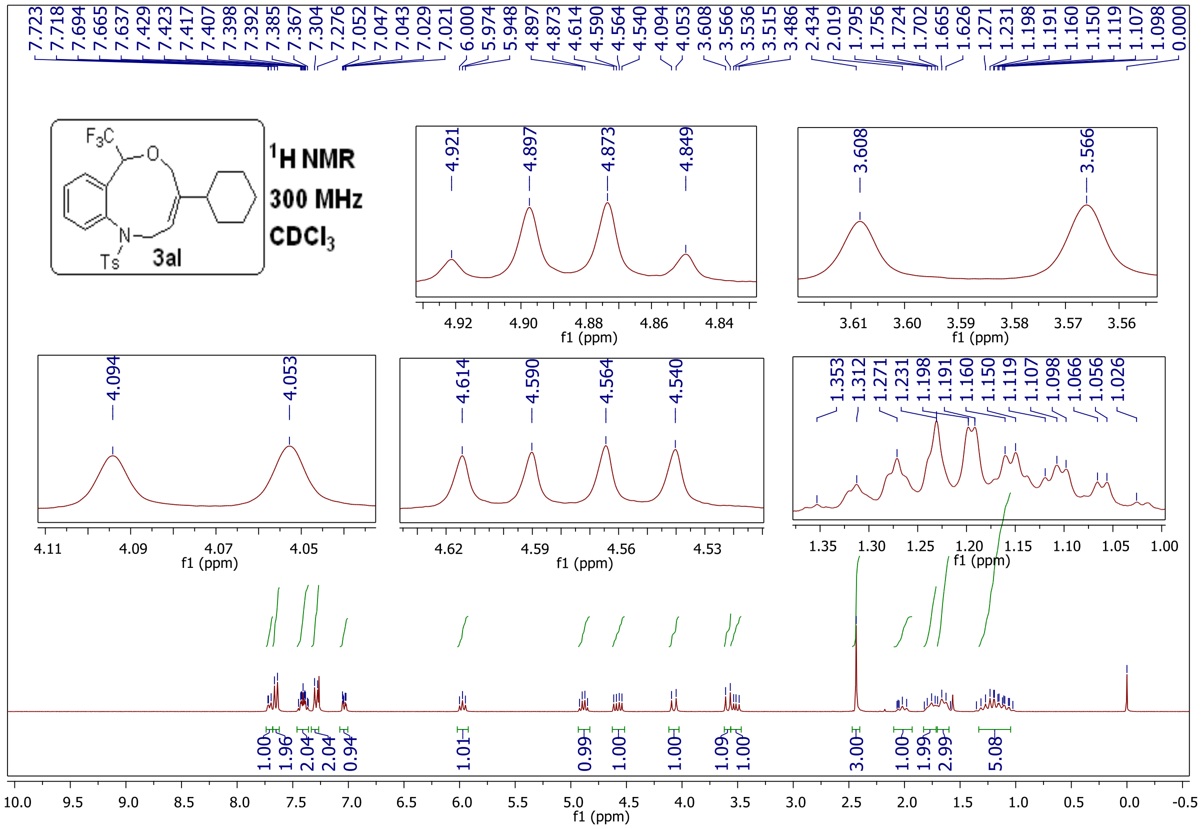

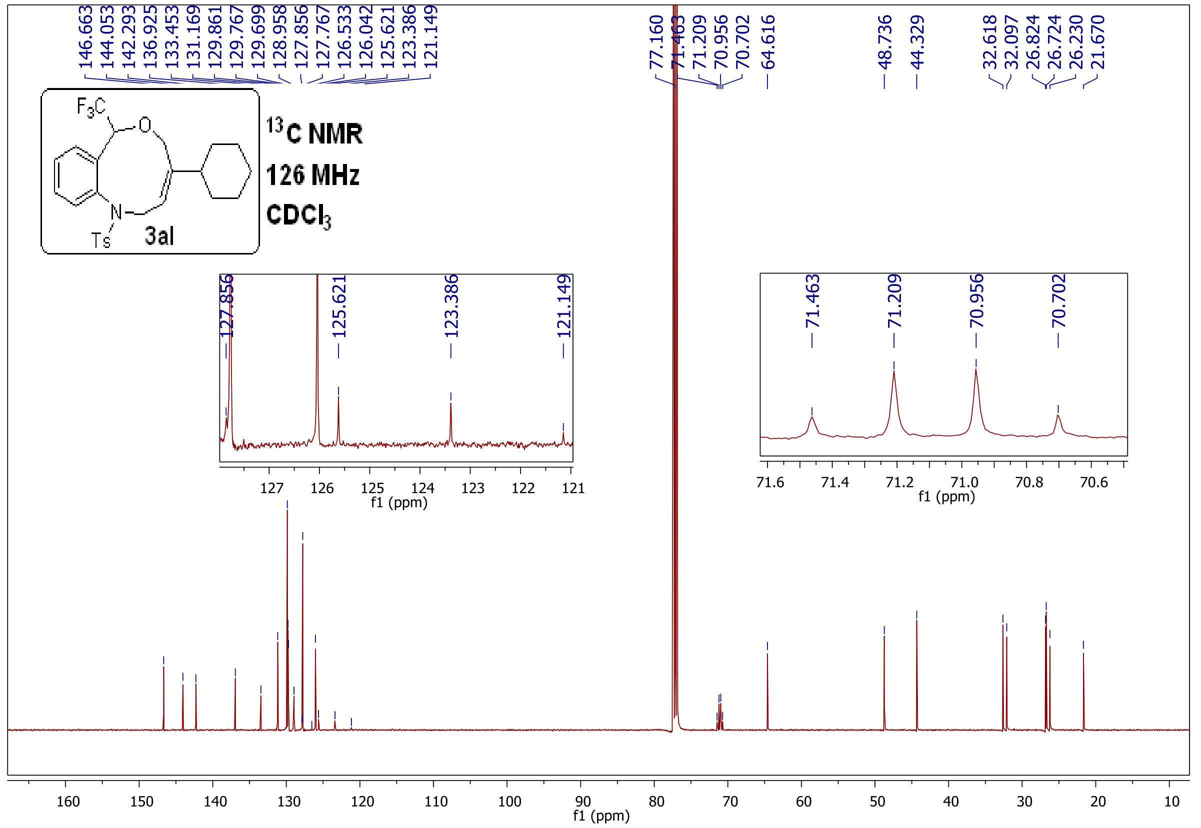

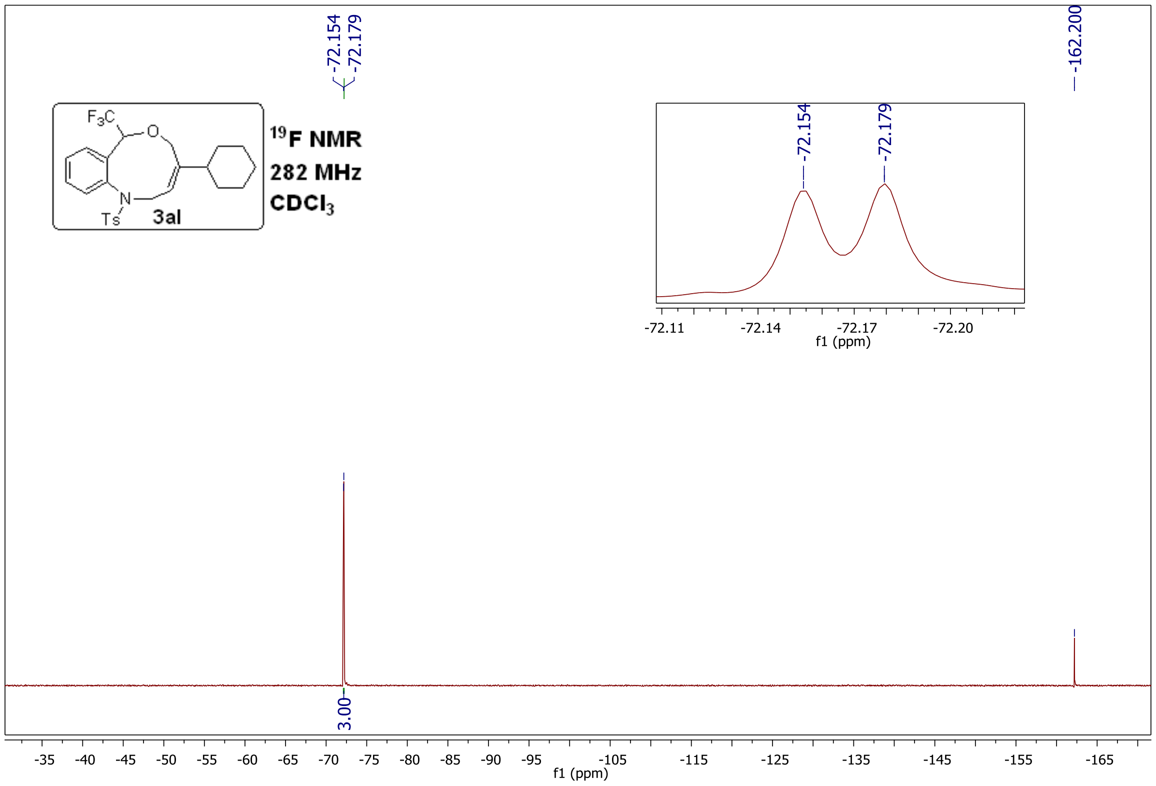

19F NMR
282 MHz
CDCl3
3al

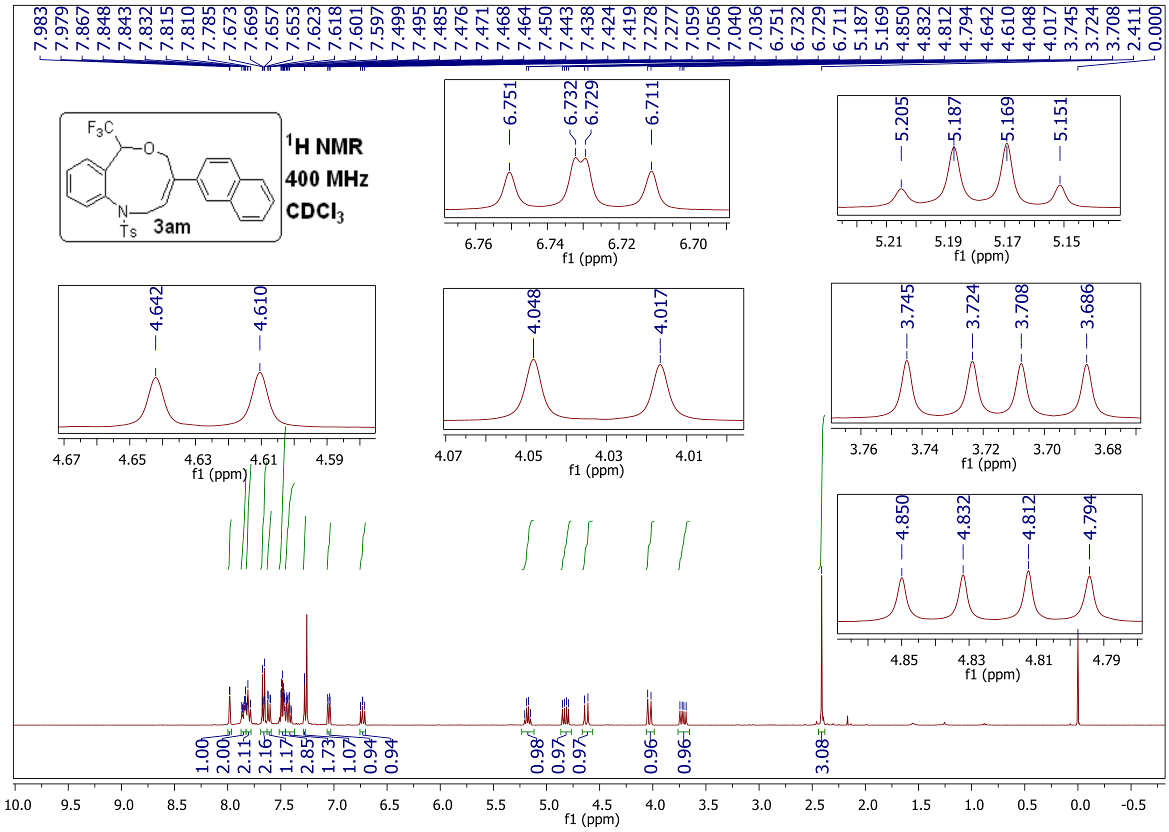

1H NMR
400 MHz
CDCl3
3am

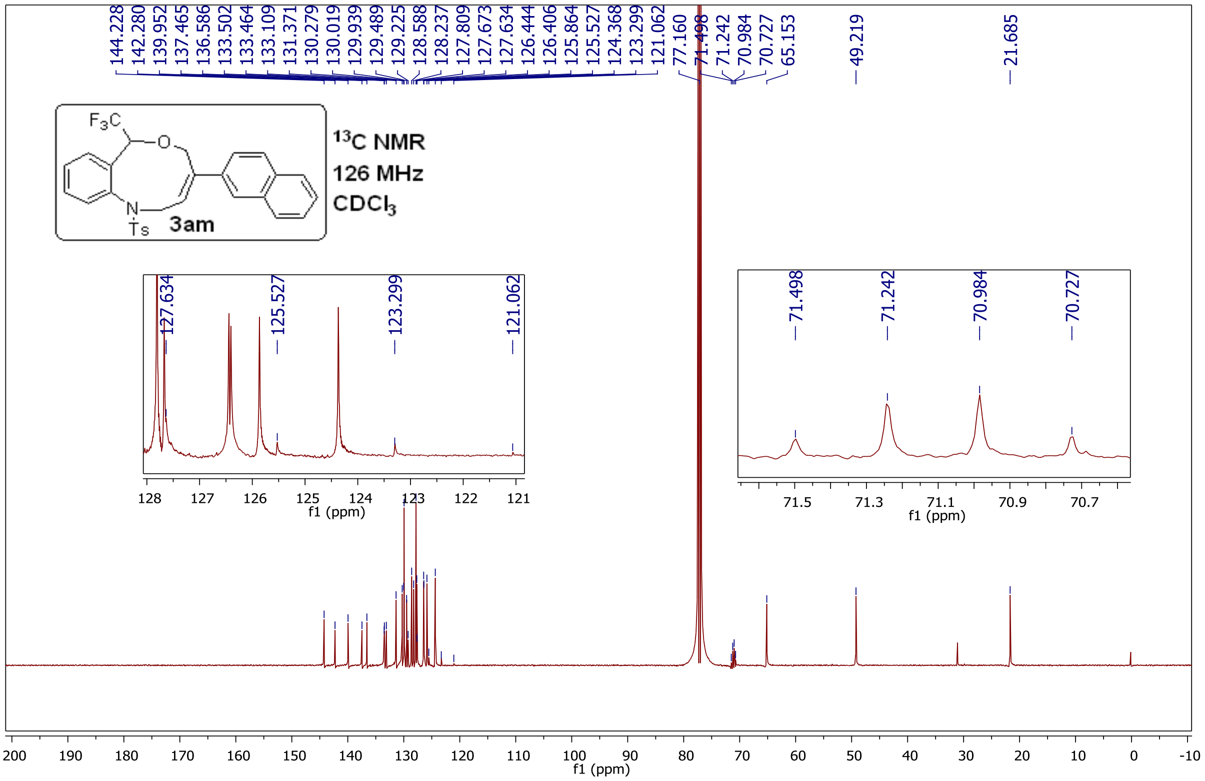

144.228
142.280
139.952
137.465
136.586
133.502
133.464
133.109
131.371
130.279
130.019
129.939
129.489
129.225
128.588
128.237
127.809
127.673
127.634
126.444
126.406
125.864
125.527
124.368
123.299
121.062
77.160
71.498
71.242
70.984
70.727
65.153
49.219
21.685
F3C
O
N
Ts
3am
13C NMR
126 MHz
CDCl3
f1 (ppm)

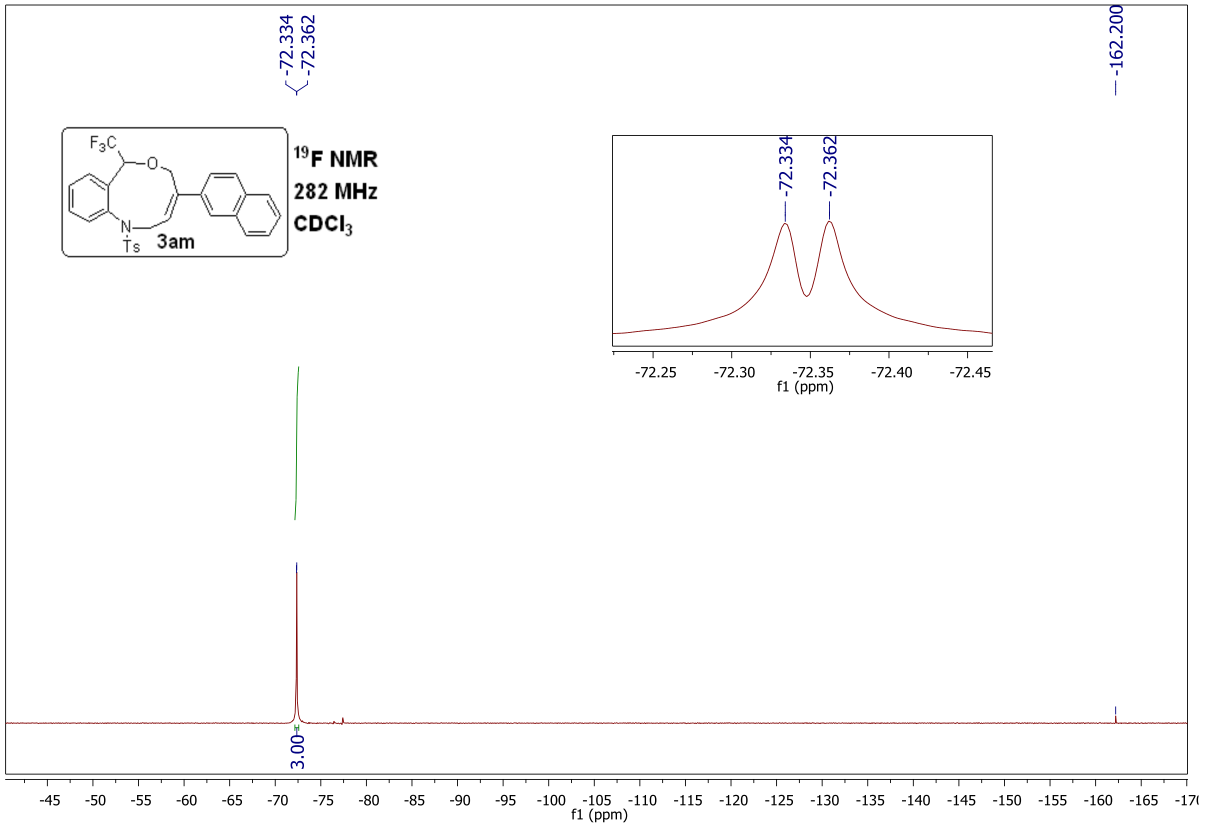

-72.334
-72.362
-162.200
F3C
O
N
Ts
3am
19F NMR
282 MHz
CDCl3
3.00
f1 (ppm)

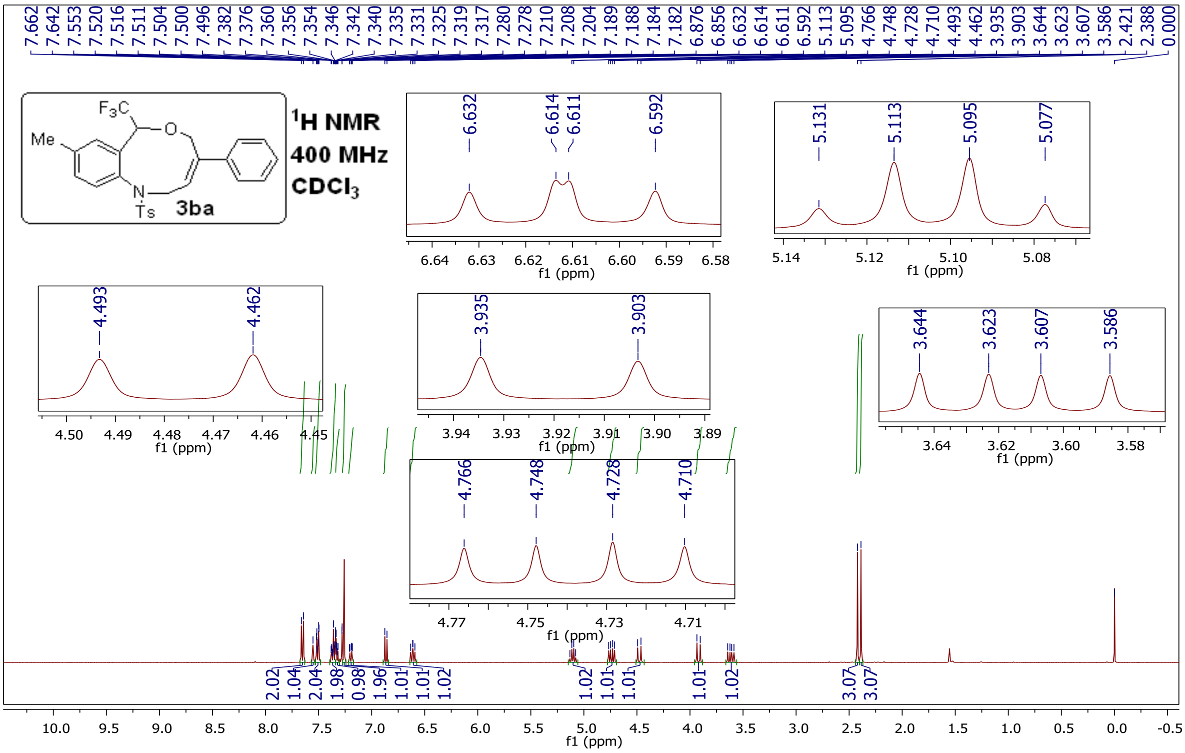

1H NMR
400 MHz
CDCl3
3ba

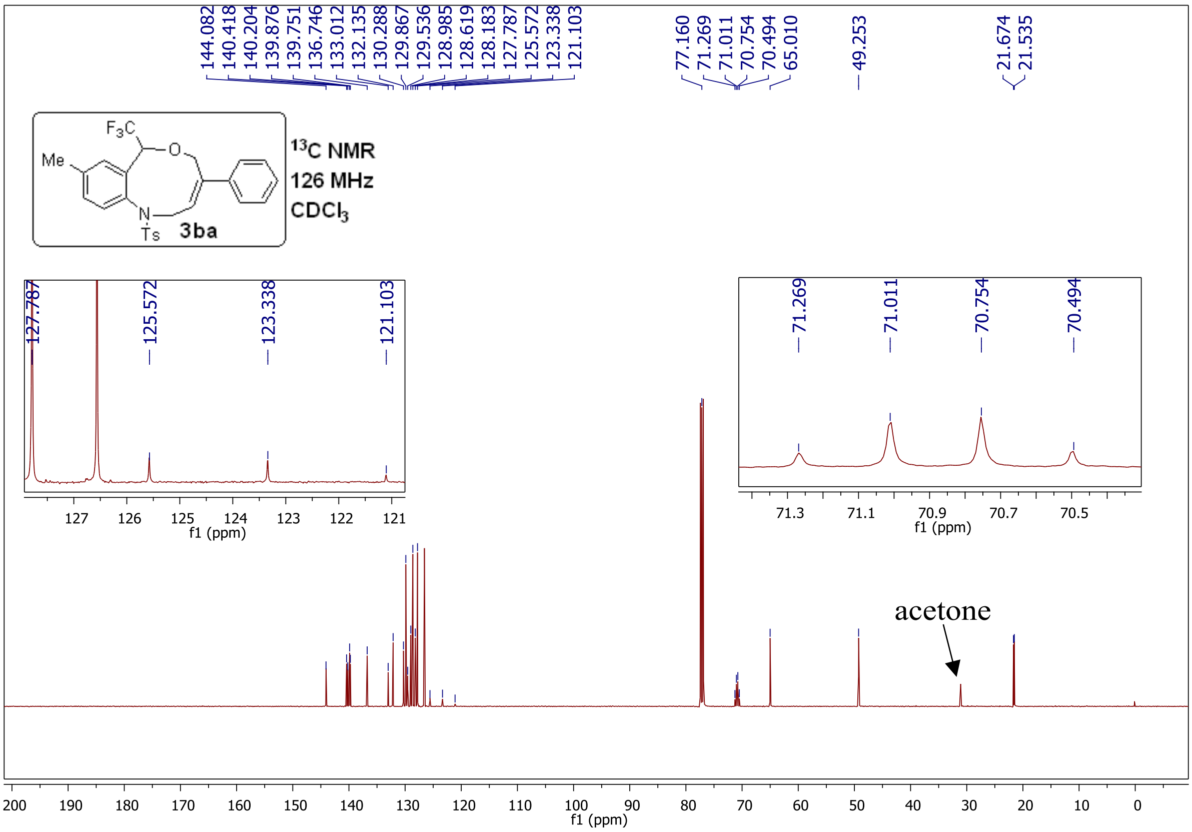

13C NMR
126 MHz
CDCl3
3ba
acetone

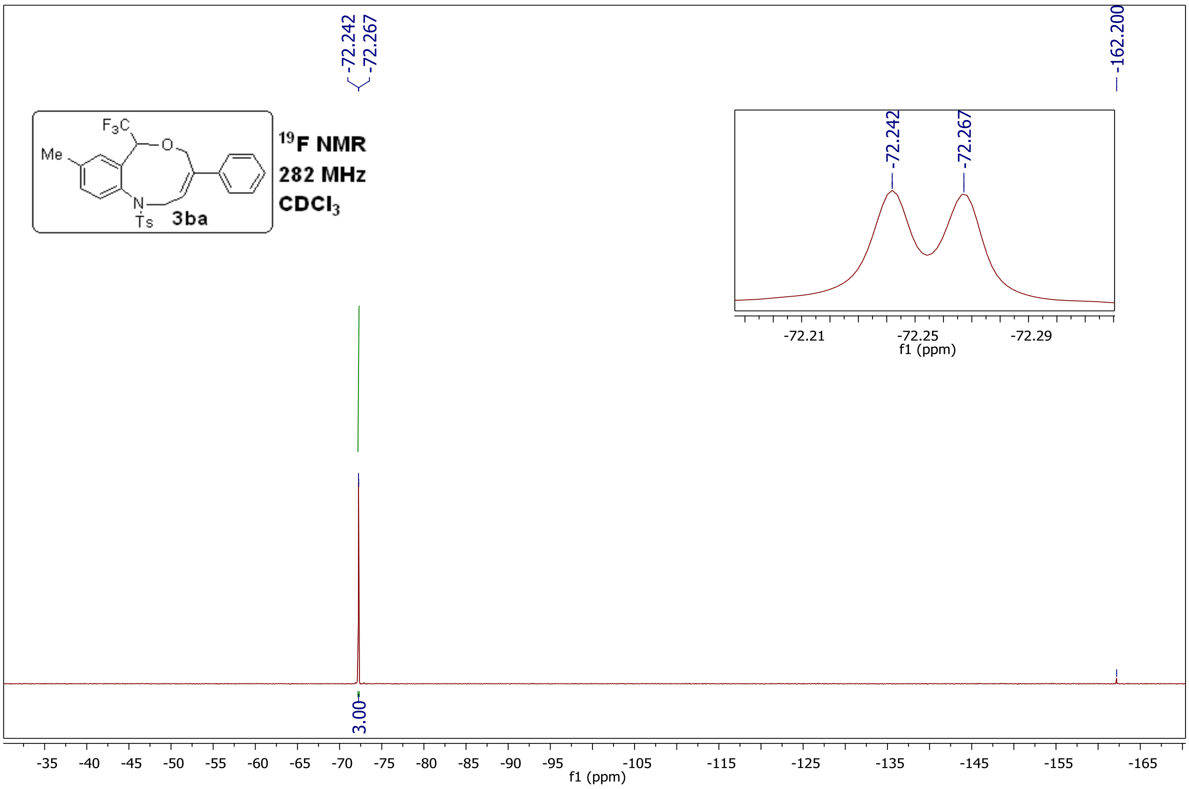

-72.242
-72.267
-162.200
F3C
O
Me
N
Ts
3ba
19F NMR
282 MHz
CDCl3
3.00
f1 (ppm)

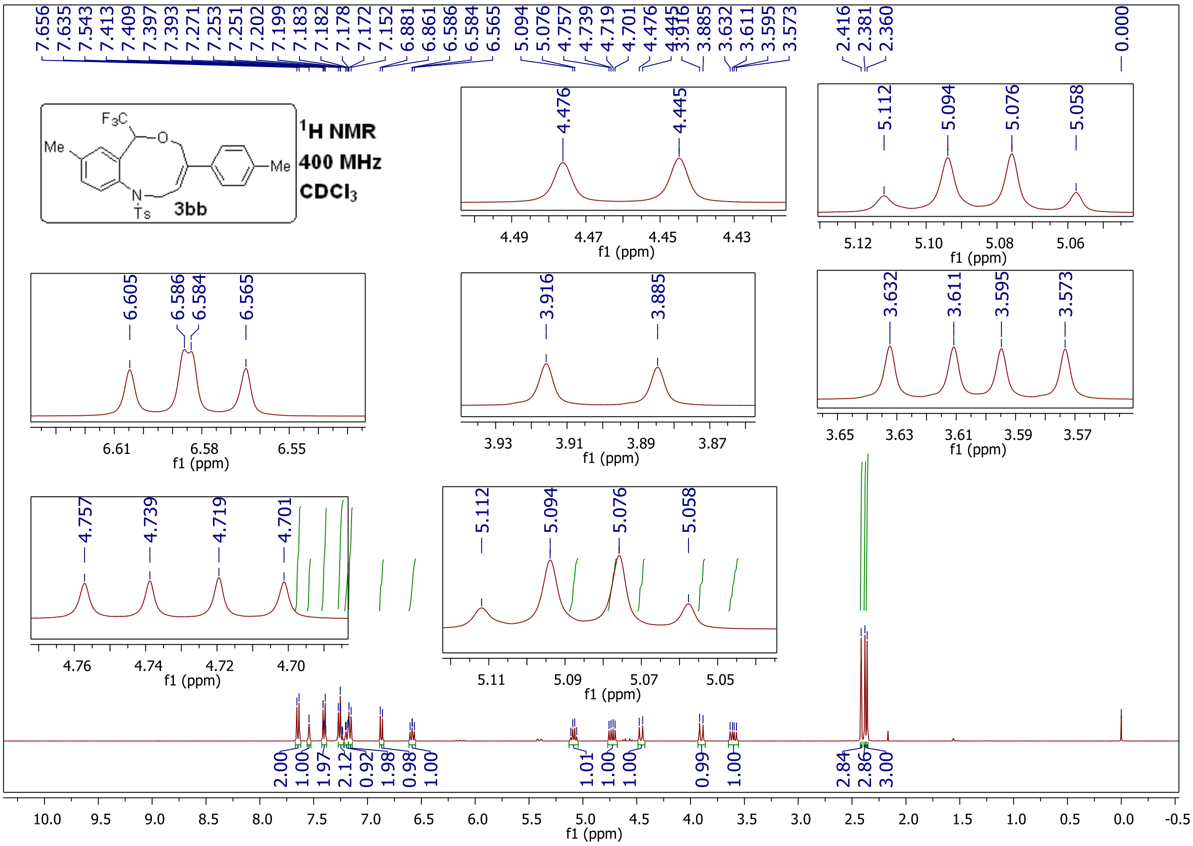

F3C
O
Me
Me
N
Ts
3bb
1H NMR
400 MHz
CDCl3
f1 (ppm)

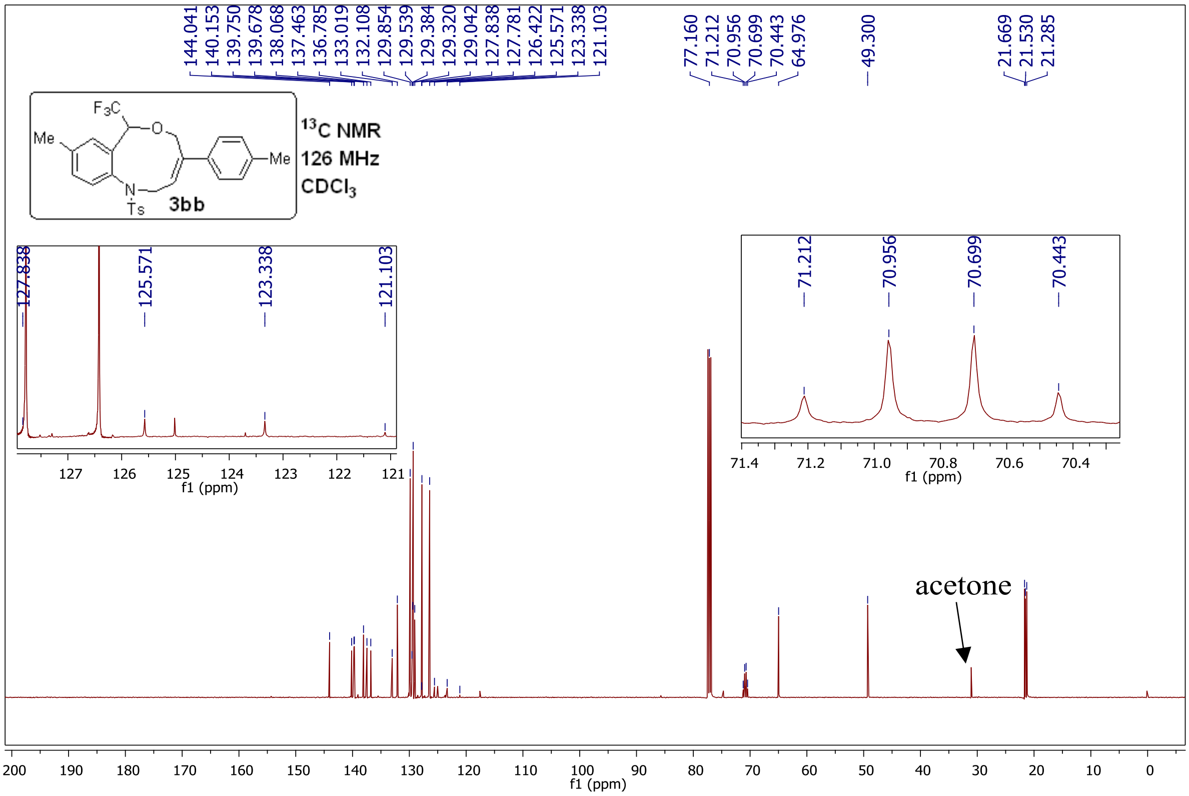

13C NMR
126 MHz
CDCl3
3bb
acetone
f1 (ppm)

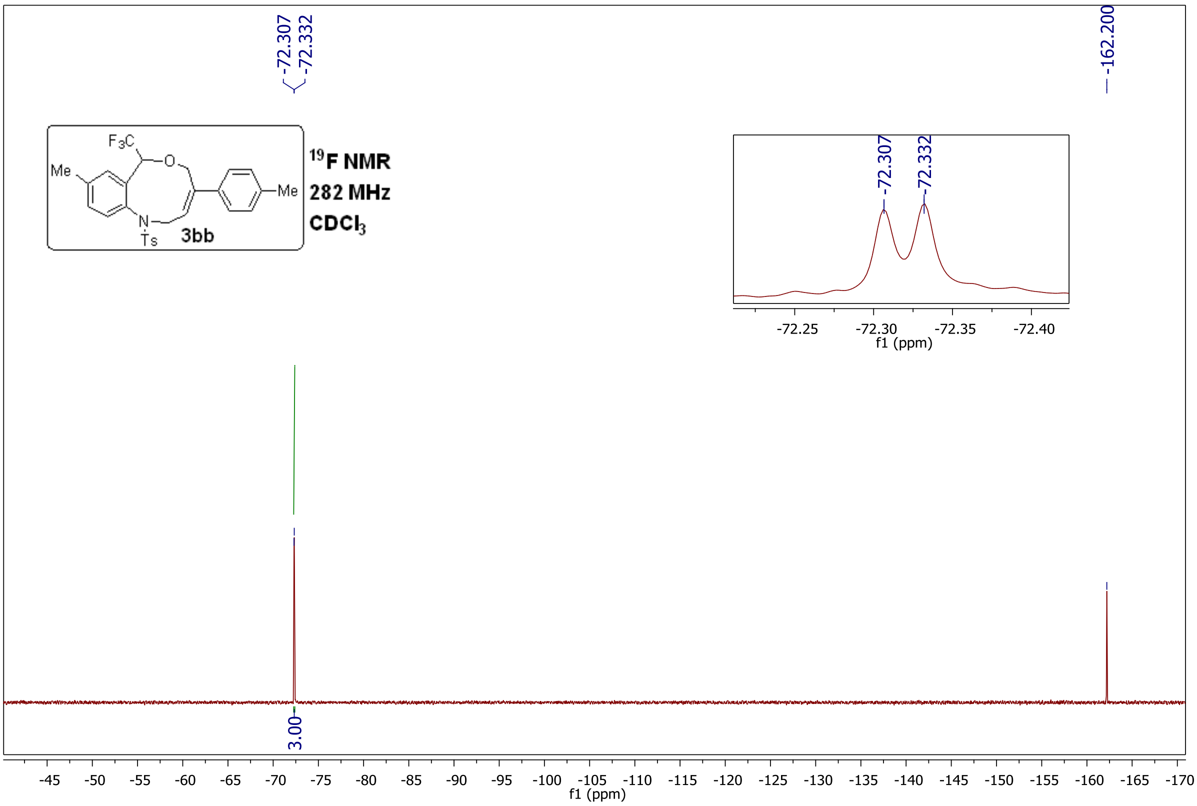

19F NMR
282 MHz
CDCl3
3bb
f1 (ppm)

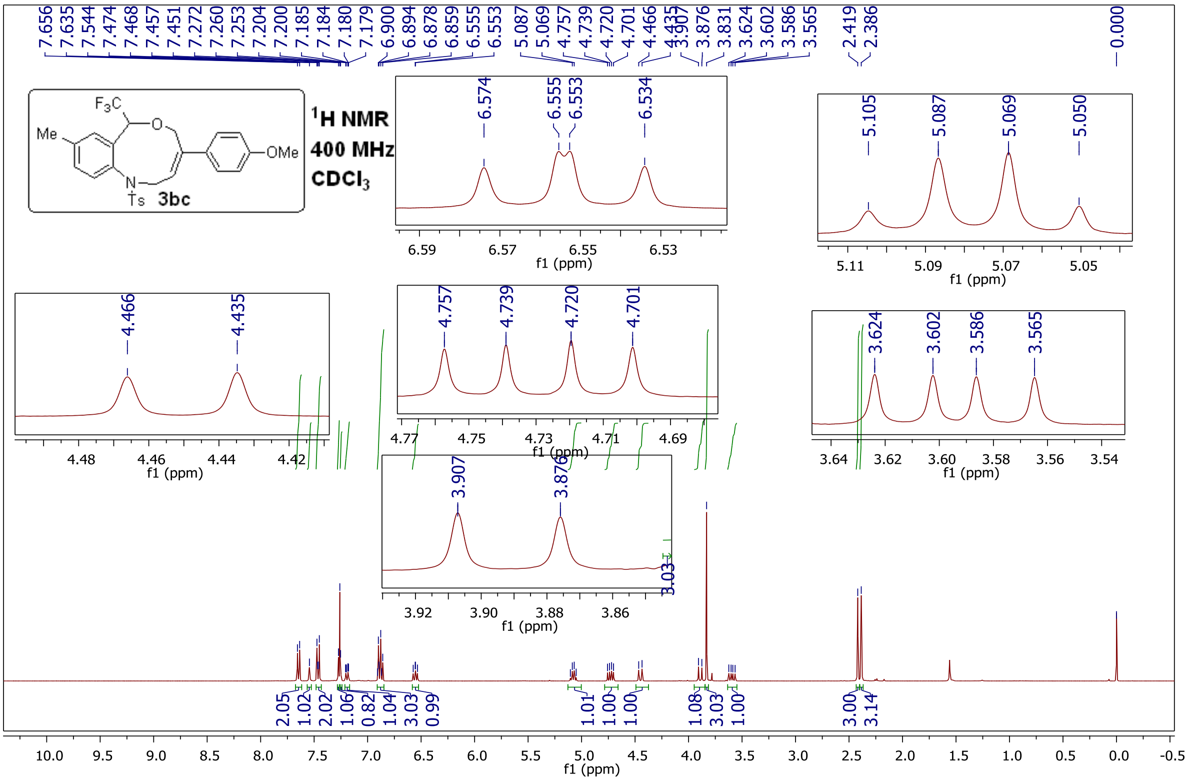
7.656 7.635 7.544 7.474 7.468 7.457 7.451 7.272 7.260 7.253 7.204 7.200 7.185 7.184 7.180 7.179 6.900 6.894 6.878 6.859 6.555 6.553 5.087 5.069 4.757 4.739 4.720 4.701 4.466 4.435 3.907 3.876 3.831 3.624 3.602 3.586 3.565 2.419 2.386 0.000
F3C
O
Me
OMe
N
Ts
3bc
1H NMR
400 MHz
CDCl3
2.05 1.02 2.02 1.06 0.82 1.04 3.03 0.99 1.01 1.00 1.00 1.08 3.03 1.00 3.00 3.14
f1 (ppm)

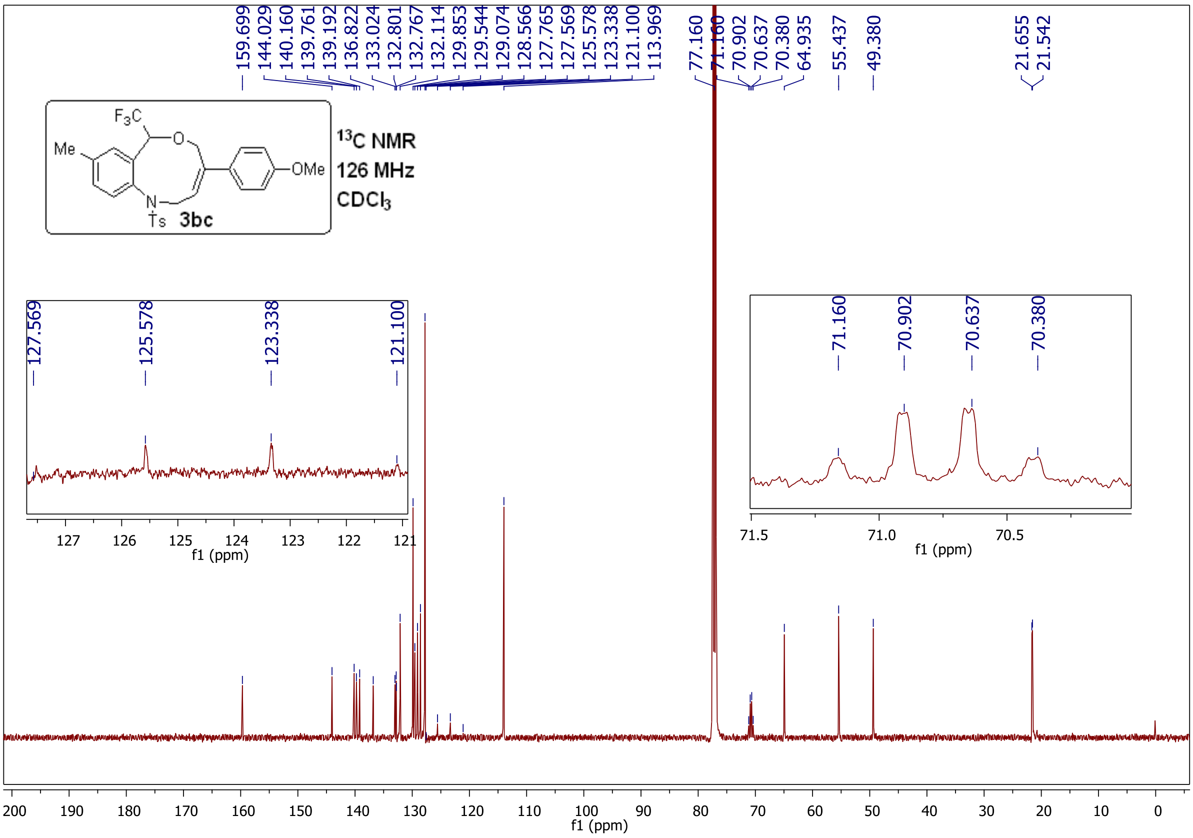
159.699 144.029 140.160 139.761 139.192 136.822 133.024 132.801 132.767 132.114 129.853 129.544 129.074 128.566 127.765 127.569 125.578 123.338 121.100 113.969 77.160 71.160 70.902 70.637 70.380 64.935 55.437 49.380 21.655 21.542
F3C
O
Me
OMe
N
Ts
3bc
13C NMR
126 MHz
CDCl3
f1 (ppm)

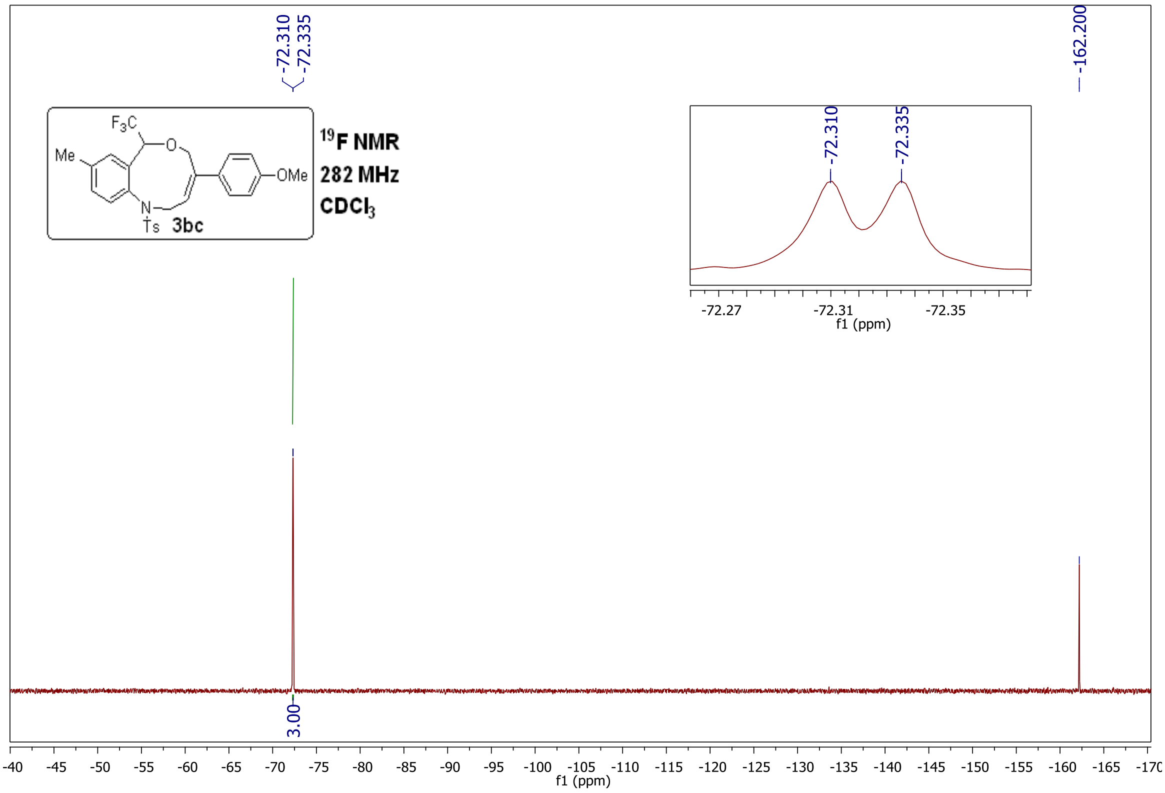

19F NMR
282 MHz
CDCl3
3bc

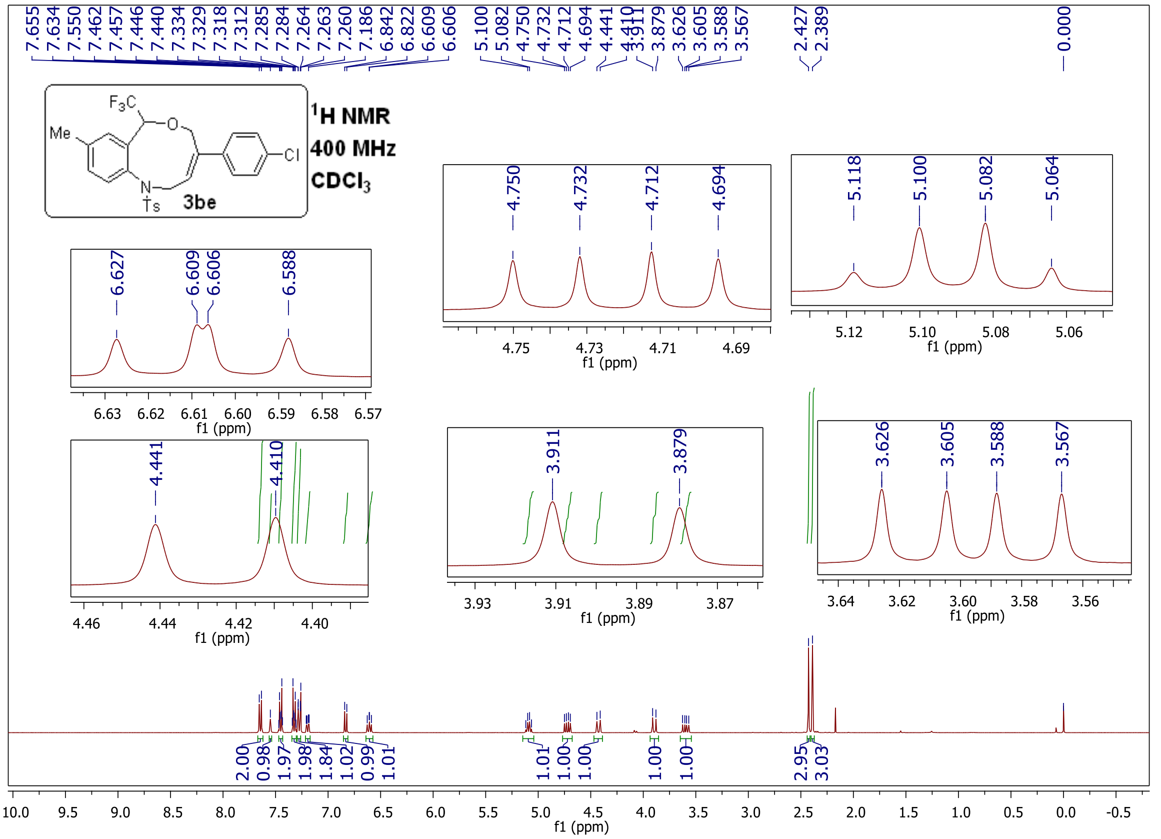

1H NMR
400 MHz
CDCl3
3be

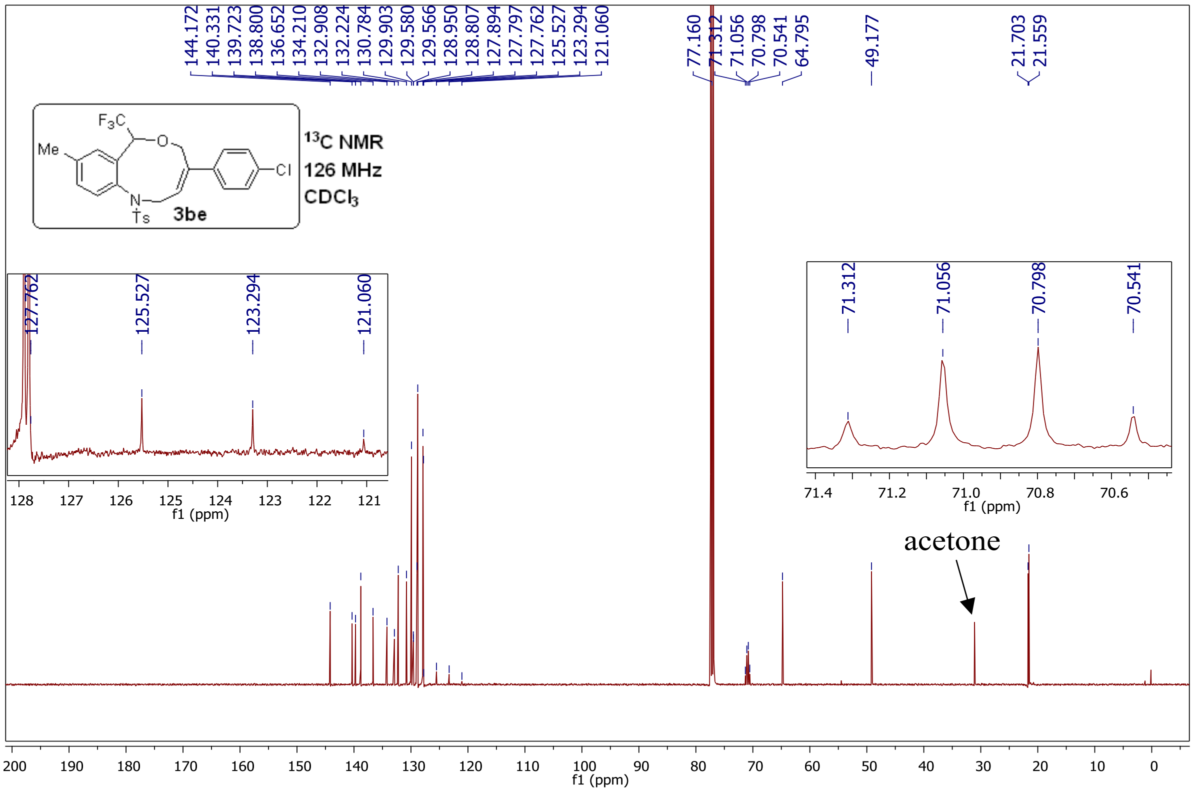

13C NMR
126 MHz
CDCl3
3be
acetone
f1 (ppm)

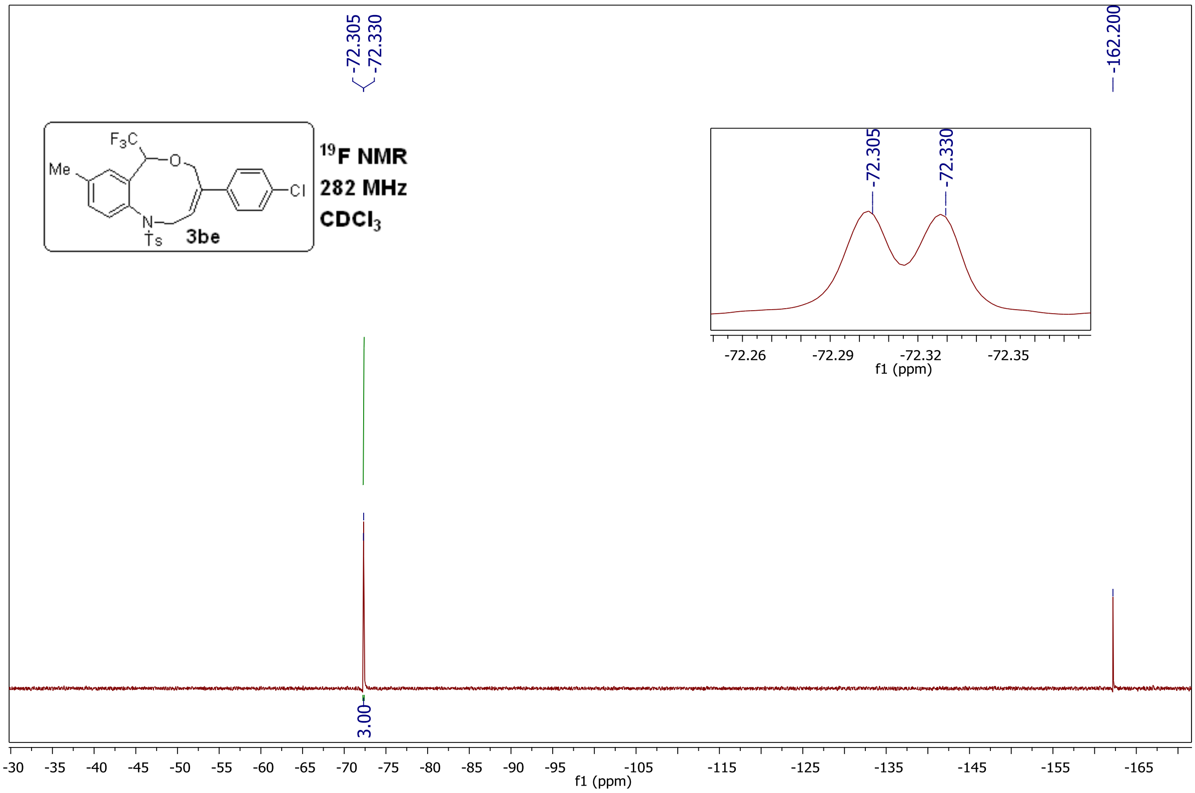

19F NMR
282 MHz
CDCl3
3be
-72.305
-72.330
-162.200
3.00
f1 (ppm)

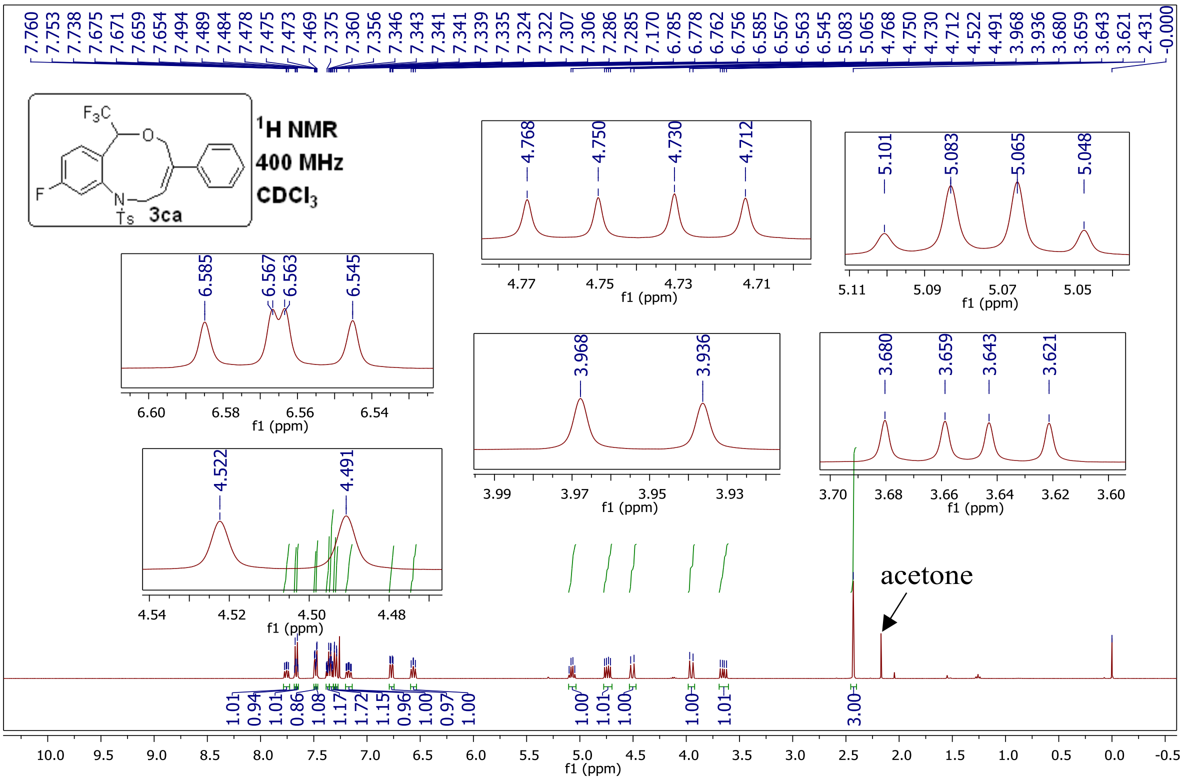
7.760 7.753 7.738 7.675 7.671 7.659 7.654 7.494 7.489 7.484 7.478 7.475 7.473 7.469 7.375 7.360 7.356 7.346 7.343 7.341 7.341 7.339 7.335 7.324 7.322 7.307 7.306 7.286 7.285 7.170 6.785 6.778 6.762 6.756 6.585 6.567 6.563 6.545 5.083 5.065 4.768 4.750 4.730 4.712 4.522 4.491 3.968 3.936 3.680 3.659 3.643 3.621 2.431 -0.000
F3C
O
F
N
Ts
3ca
1H NMR
400 MHz
CDCl3
acetone
1.01 0.94 1.01 0.86 1.08 1.17 1.72 1.15 0.96 1.00 0.97 1.00
1.00 1.01 1.00
1.00 1.01
3.00
f1 (ppm)

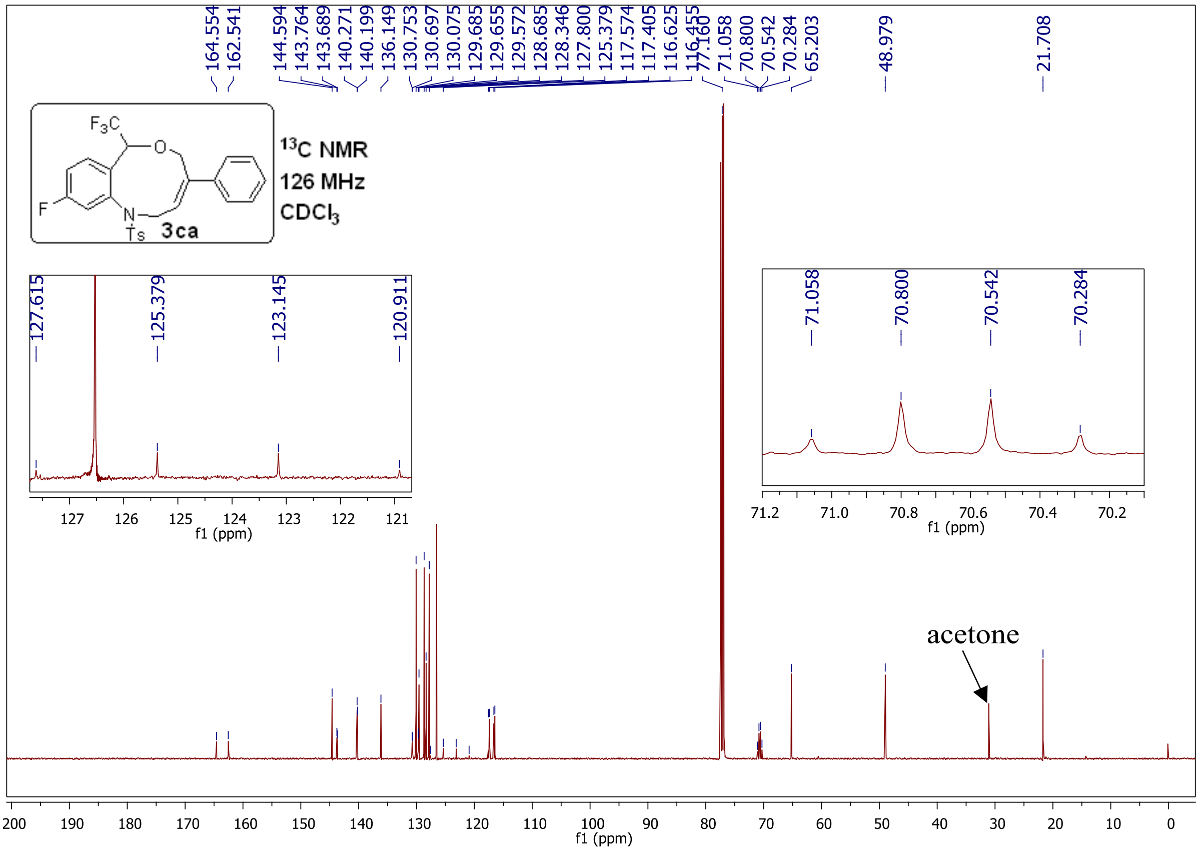
164.554 162.541 144.594 143.764 143.689 140.271 140.199 136.149 130.753 130.697 130.075 129.685 129.655 129.572 128.685 128.346 127.800 125.379 117.574 117.405 116.625 116.455 77.160 71.058 70.800 70.542 70.284 65.203 48.979 21.708
F3C
O
F
N
Ts
3ca
13C NMR
126 MHz
CDCl3
127.615 125.379 123.145 120.911
71.058 70.800 70.542 70.284
acetone
f1 (ppm)

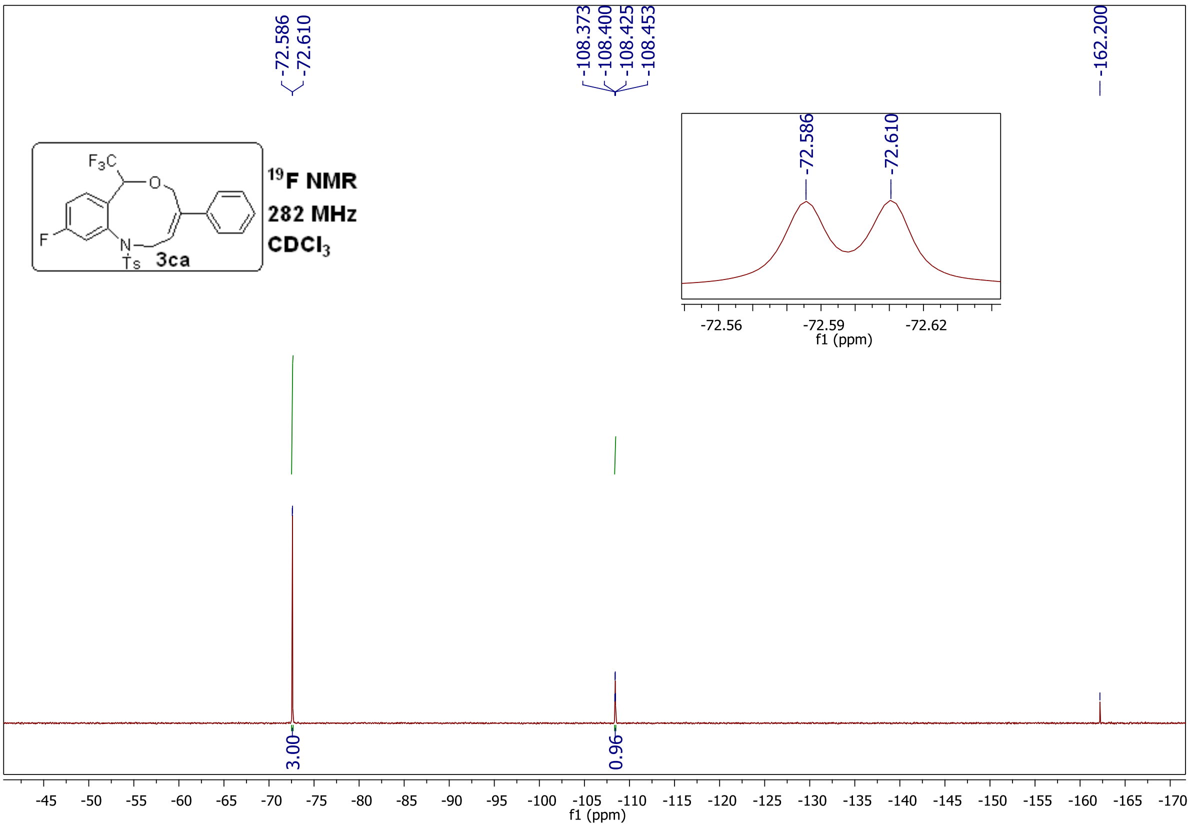

¹⁹F NMR
282 MHz
CDCl₃
3ca

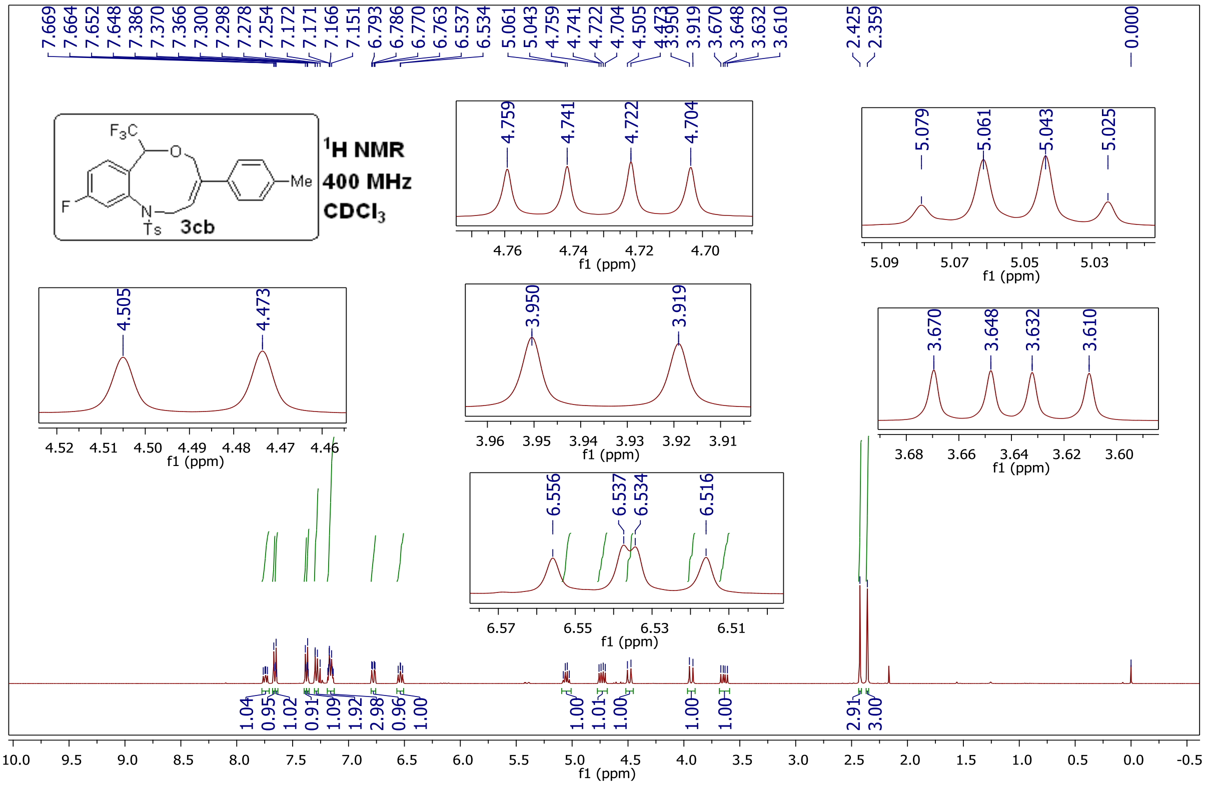

¹H NMR
400 MHz
CDCl₃
3cb

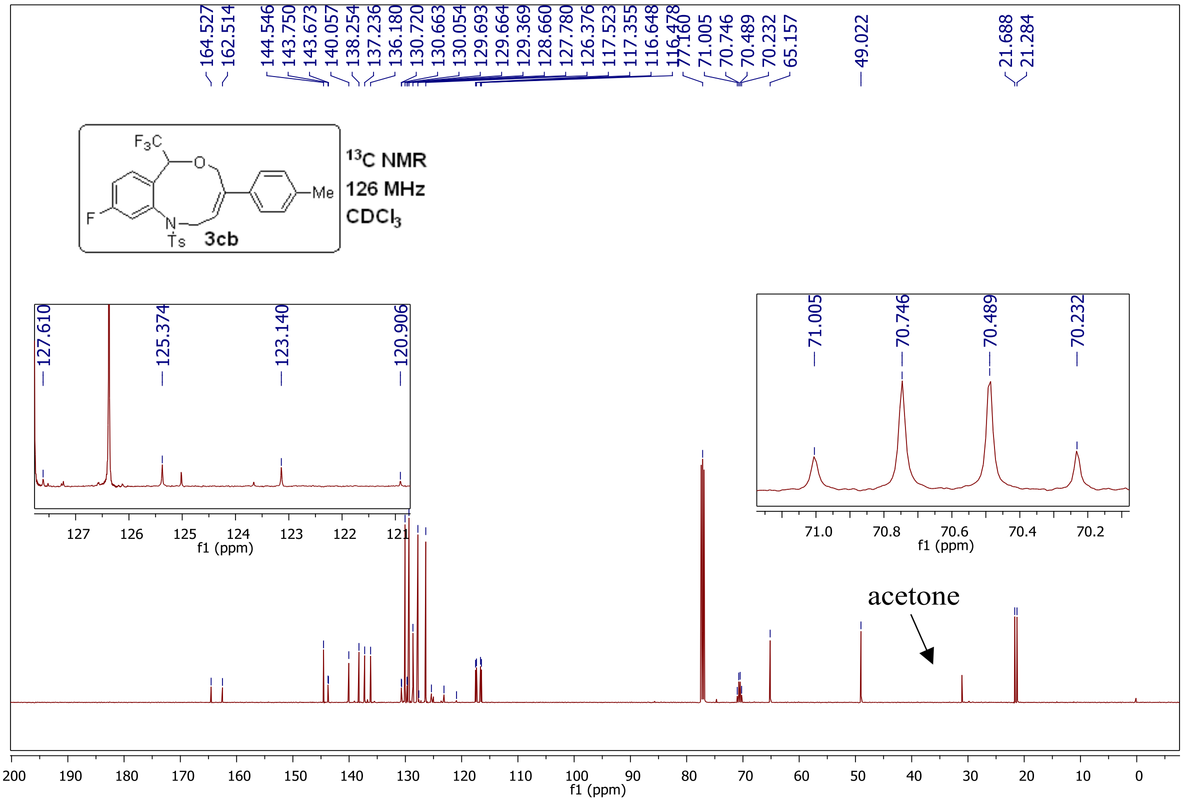
13C NMR
126 MHz
CDCl3
3cb
acetone
f1 (ppm)

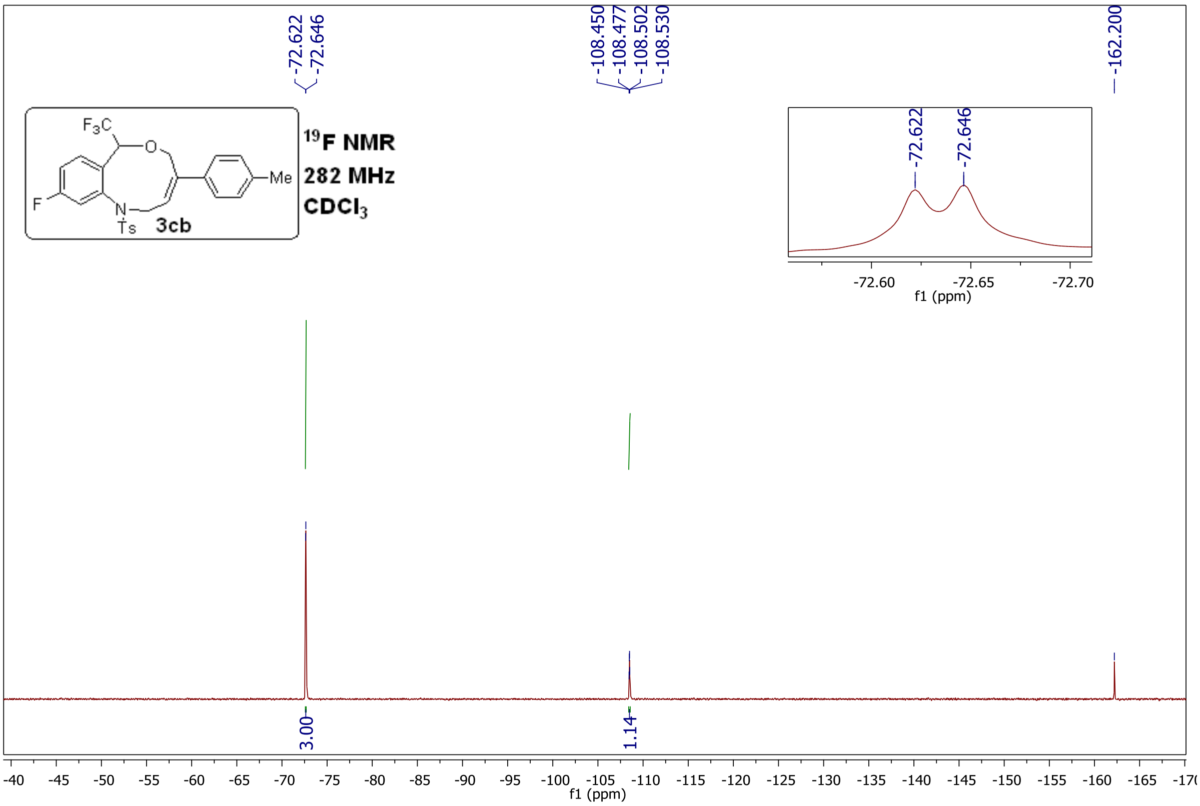
19F NMR
282 MHz
CDCl3
3cb
f1 (ppm)

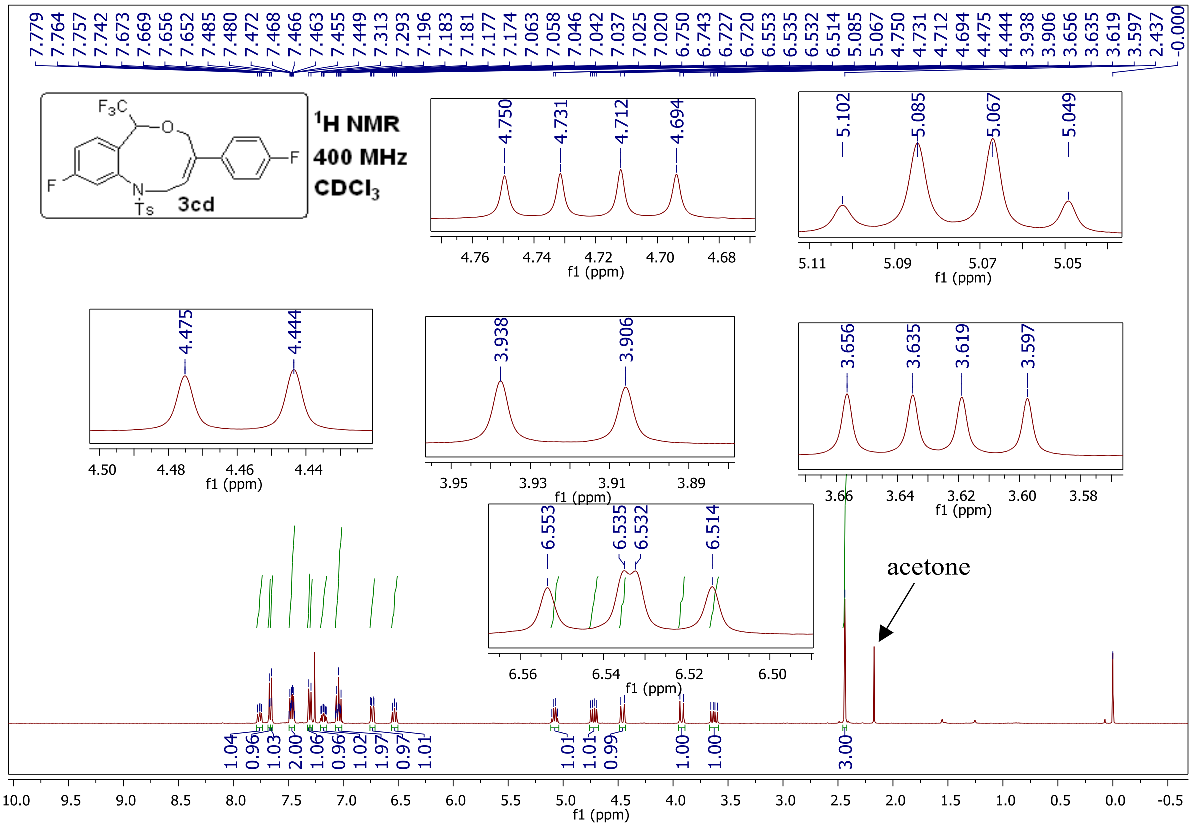
7.779 7.764 7.757 7.742 7.673 7.669 7.656 7.652 7.485 7.480 7.472 7.468 7.466 7.463 7.455 7.449 7.313 7.293 7.196 7.183 7.181 7.177 7.174 7.063 7.058 7.046 7.042 7.037 7.025 7.020 6.750 6.743 6.727 6.720 6.553 6.535 6.532 6.514 5.085 5.067 4.750 4.731 4.712 4.694 4.475 4.444 3.938 3.906 3.656 3.635 3.619 3.597 2.437 -0.000
F3C
O
F
F
N
Ts
3cd
1H NMR
400 MHz
CDCl3
acetone
1.04 0.96 1.03 2.00 1.06 0.96 1.02 1.97 0.97 1.01
1.01 1.01 0.99 1.00 1.00 3.00
f1 (ppm)

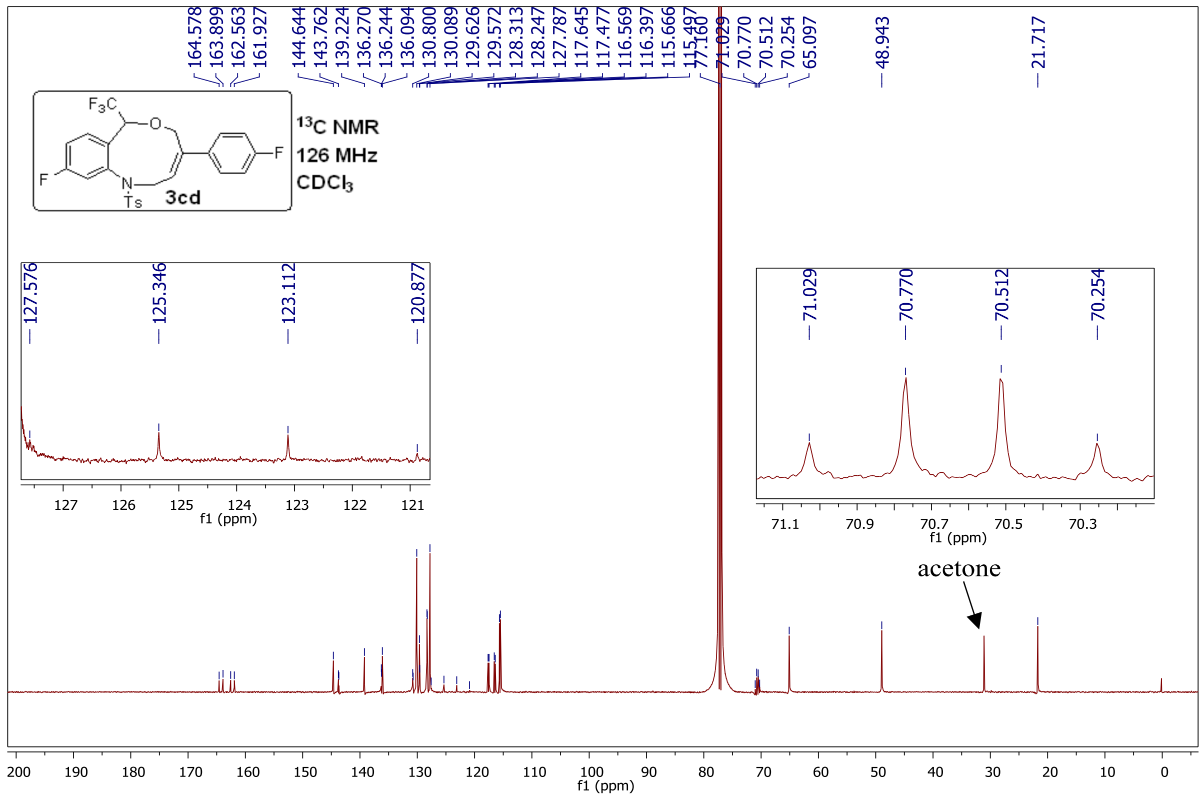
164.578 163.899 162.563 161.927 144.644 143.762 139.224 136.270 136.244 136.094 130.800 130.089 129.626 129.572 128.313 128.247 127.787 117.645 117.477 116.569 116.397 115.666 115.497 77.160 71.029 70.770 70.512 70.254 65.097 48.943 21.717
F3C
O
F
F
N
Ts
3cd
13C NMR
126 MHz
CDCl3
127.576 125.346 123.112 120.877
71.029 70.770 70.512 70.254
acetone
f1 (ppm)

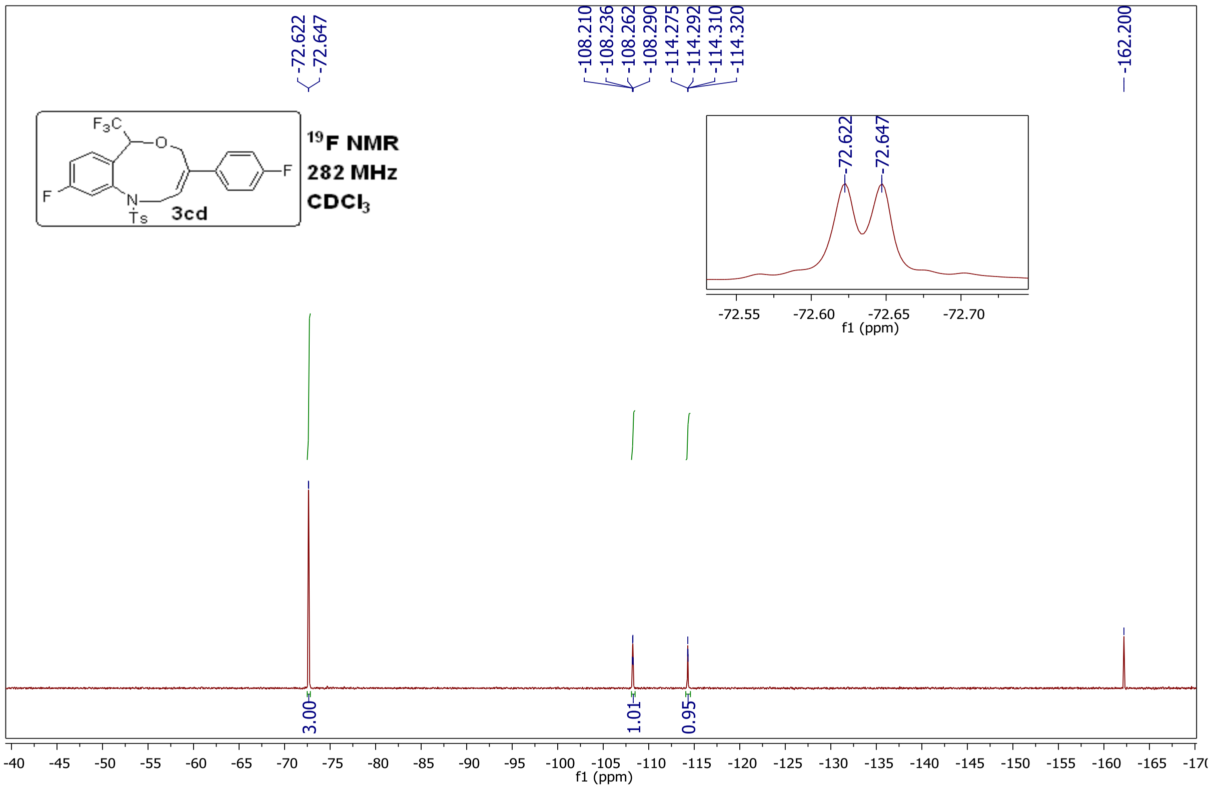
19F NMR
282 MHz
CDCl3
3cd
f1 (ppm)

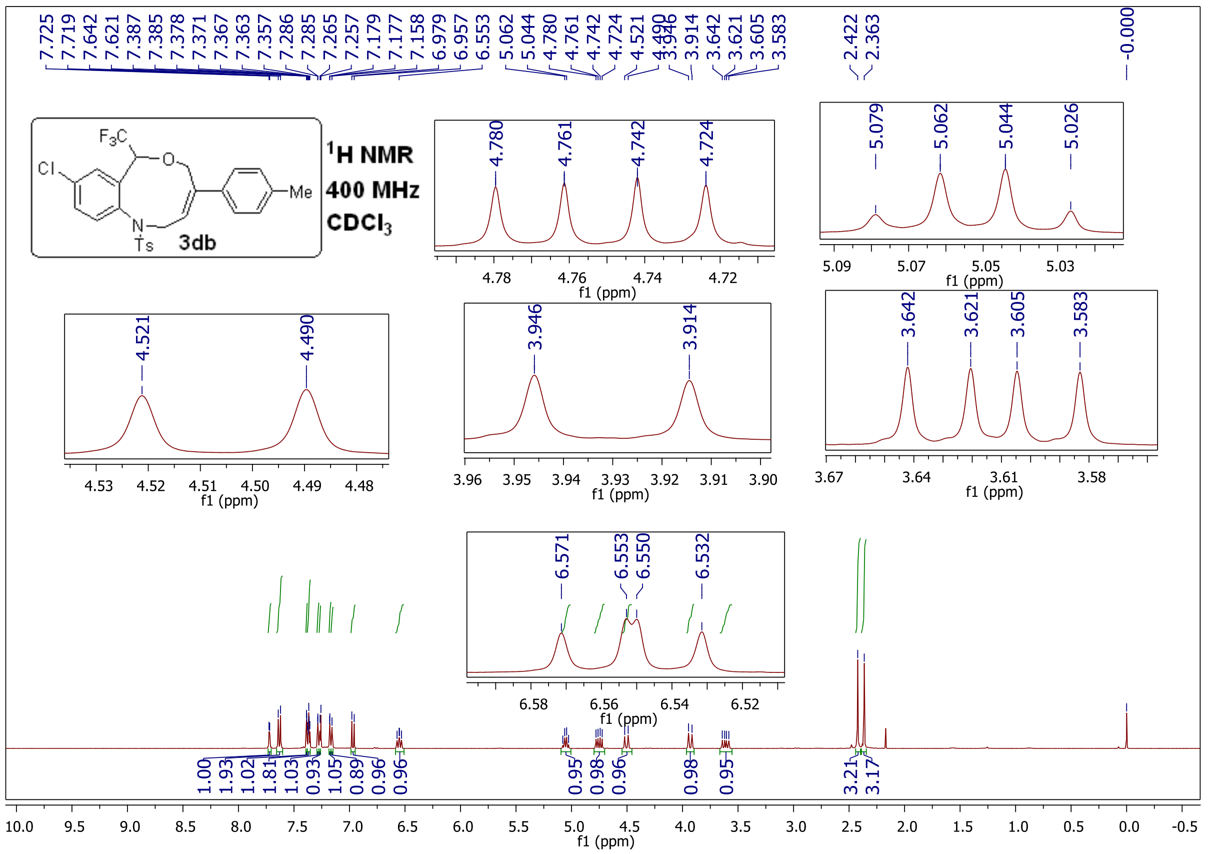
1H NMR
400 MHz
CDCl3
3db
f1 (ppm)

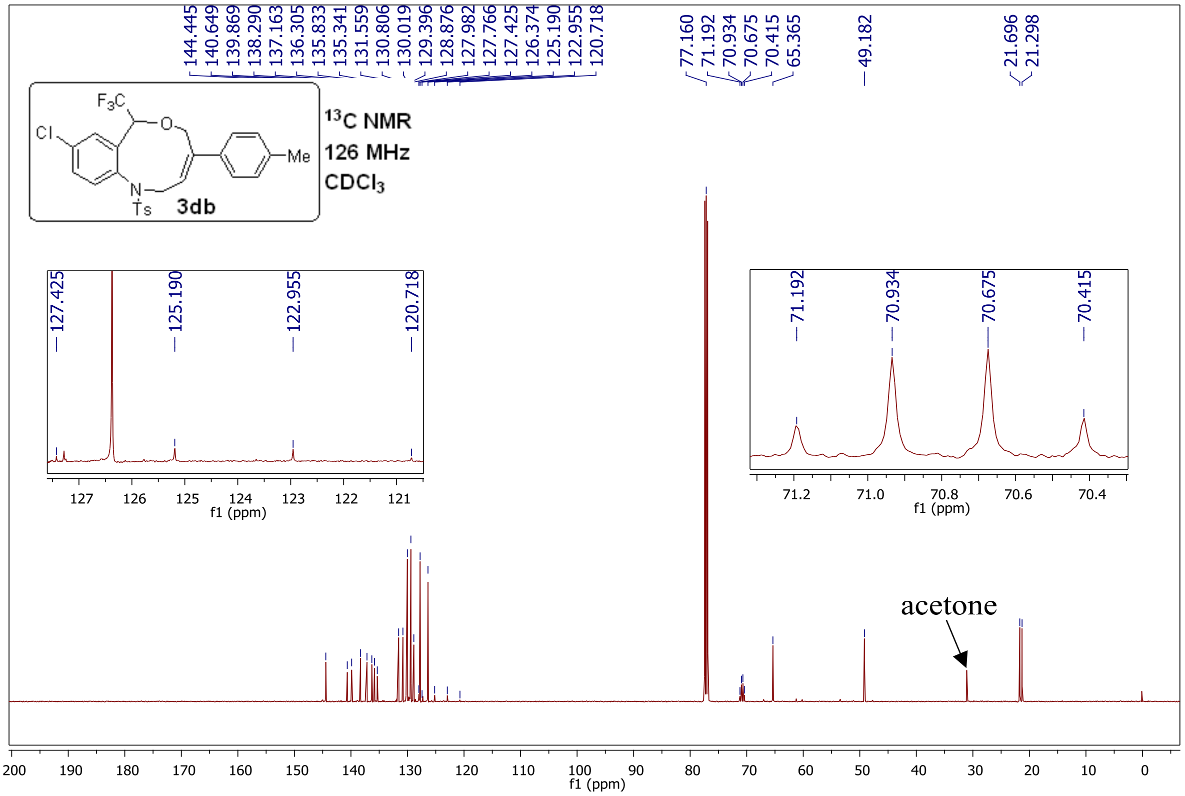
144.445
140.649
139.869
138.290
137.163
136.305
135.833
135.341
131.559
130.806
130.019
129.396
128.876
127.982
127.766
127.425
126.374
125.190
122.955
120.718
77.160
71.192
70.934
70.675
70.415
65.365
49.182
21.696
21.298
F3C
Cl
O
N
Ts
Me
3db
13C NMR
126 MHz
CDCl3
acetone
f1 (ppm)

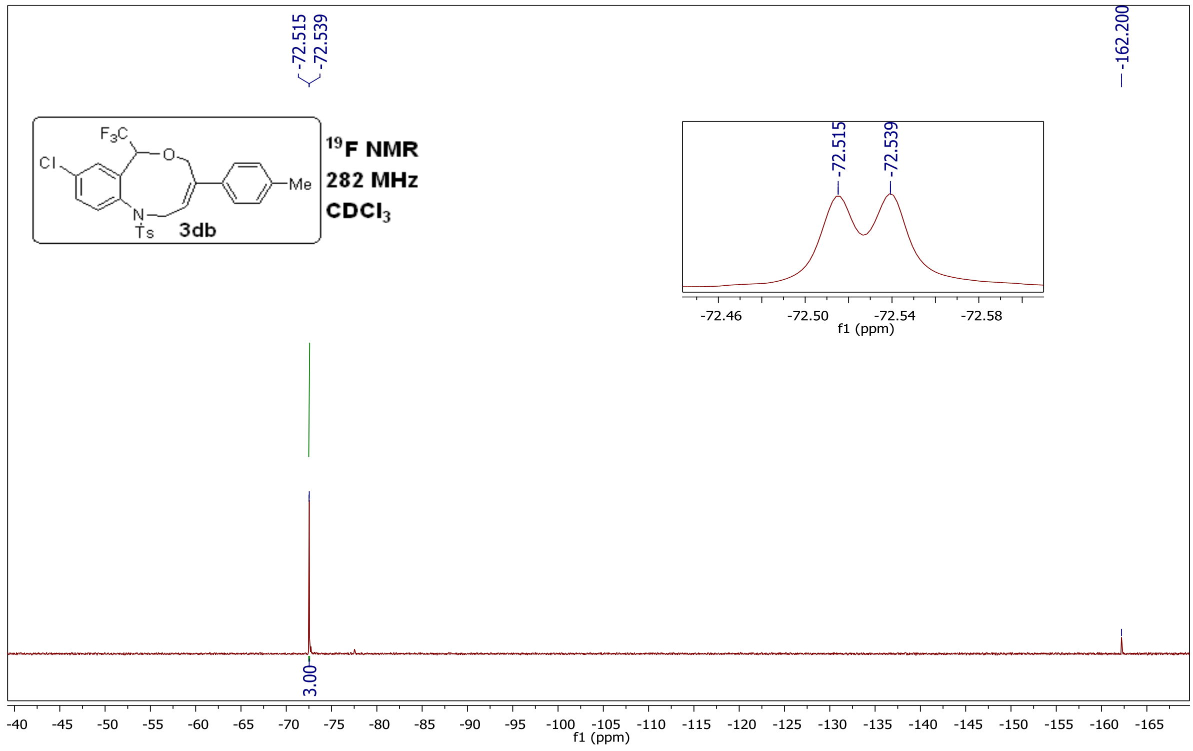
-72.515
-72.539
-162.200
F3C
Cl
O
N
Ts
Me
3db
19F NMR
282 MHz
CDCl3
3.00
f1 (ppm)

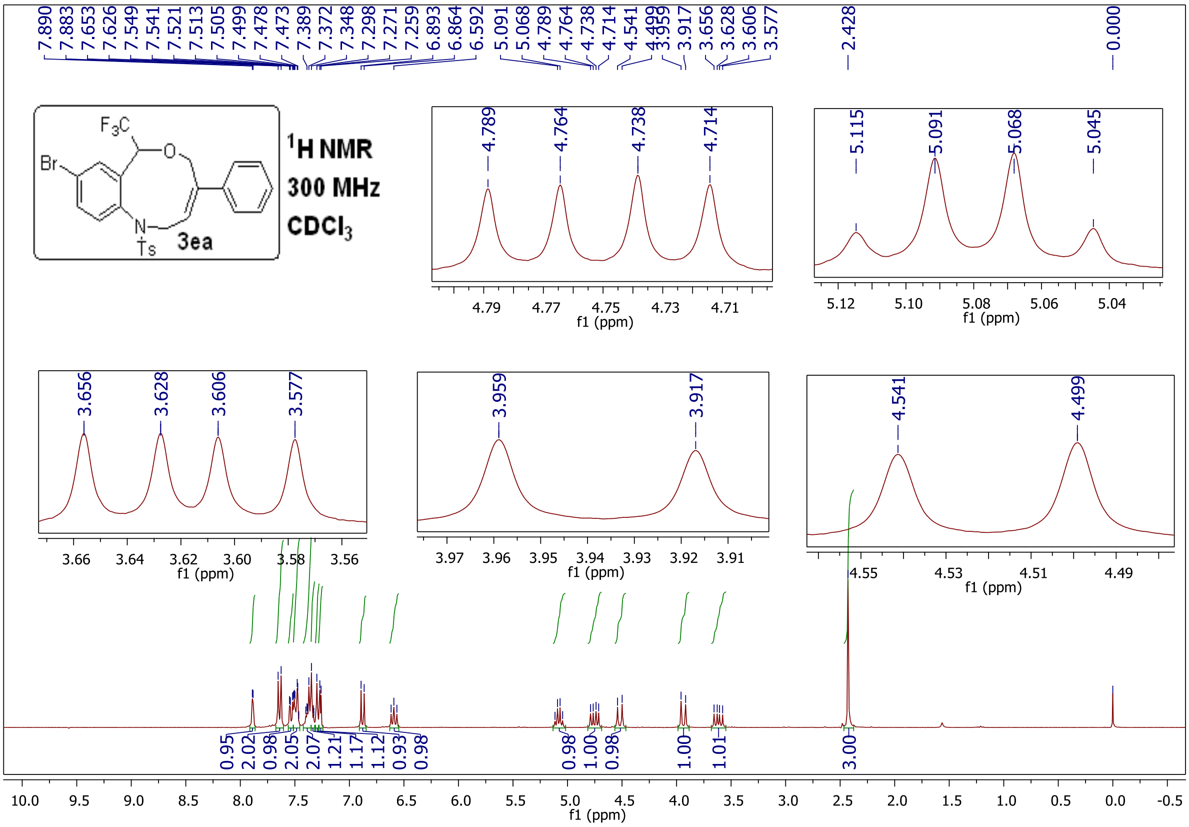
7.890 7.883 7.653 7.626 7.549 7.541 7.521 7.513 7.505 7.499 7.478 7.473 7.389 7.372 7.348 7.298 7.271 7.259 6.893 6.864 6.592 5.091 5.068 4.789 4.764 4.738 4.714 4.541 4.499 3.959 3.917 3.656 3.628 3.606 3.577 2.428 0.000
F3C
O
Br
N
Ts
3ea
1H NMR
300 MHz
CDCl3
0.95 2.02 0.98 2.05 2.07 1.21 1.17 1.12 0.93 0.98 0.98 1.00 0.98 1.00 1.01 3.00
f1 (ppm)

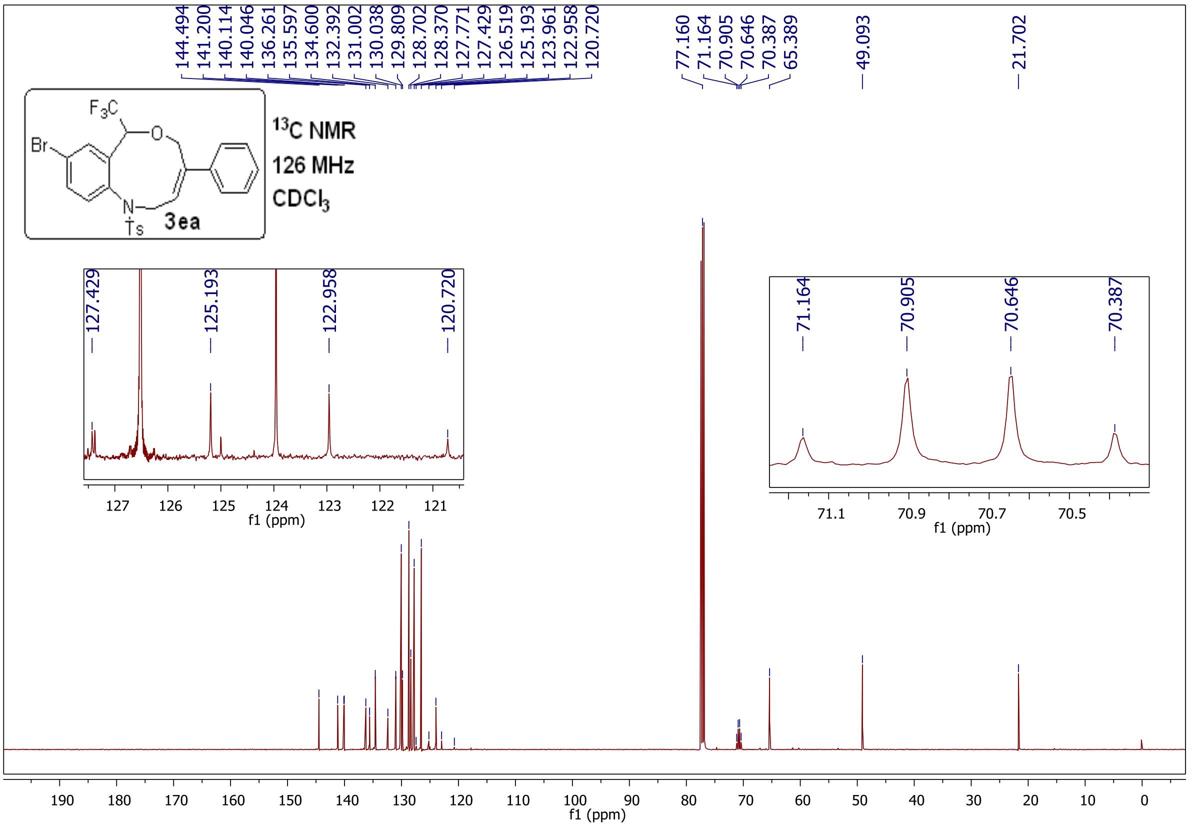
144.494 141.200 140.114 140.046 136.261 135.597 134.600 132.392 131.002 130.038 129.809 128.702 128.370 127.771 127.429 126.519 125.193 123.961 122.958 120.720 77.160 71.164 70.905 70.646 70.387 65.389 49.093 21.702
F3C
O
Br
N
Ts
3ea
13C NMR
126 MHz
CDCl3
f1 (ppm)

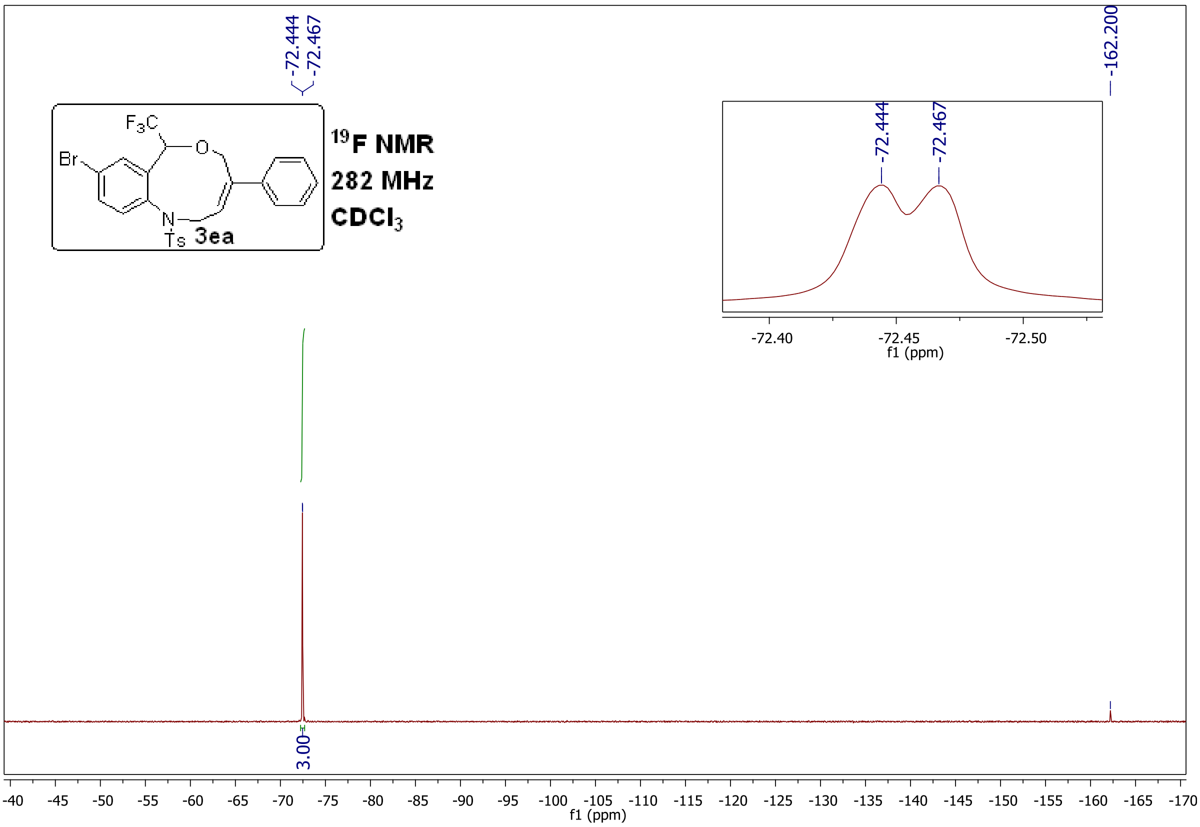

3ea
19F NMR
282 MHz
CDCl3
-72.444
-72.467
-162.200
3.00
f1 (ppm)

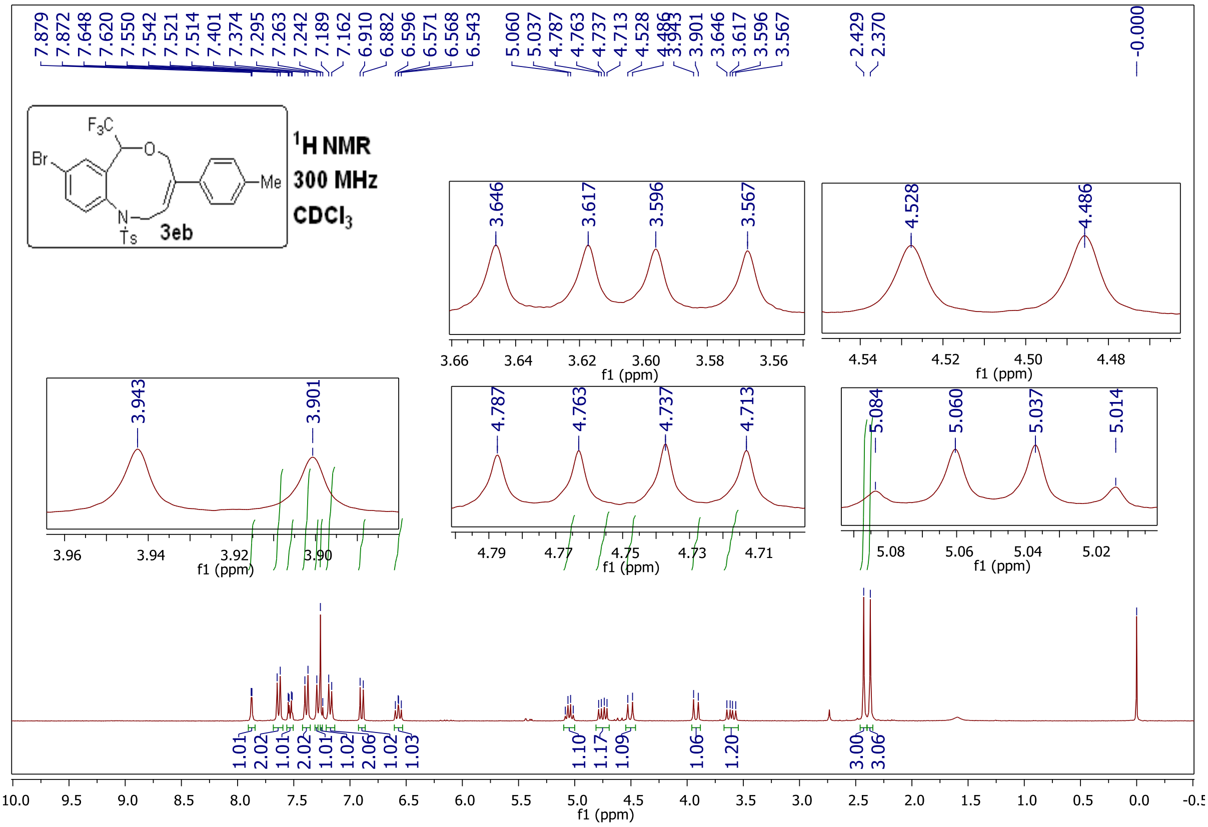

3eb
1H NMR
300 MHz
CDCl3
f1 (ppm)

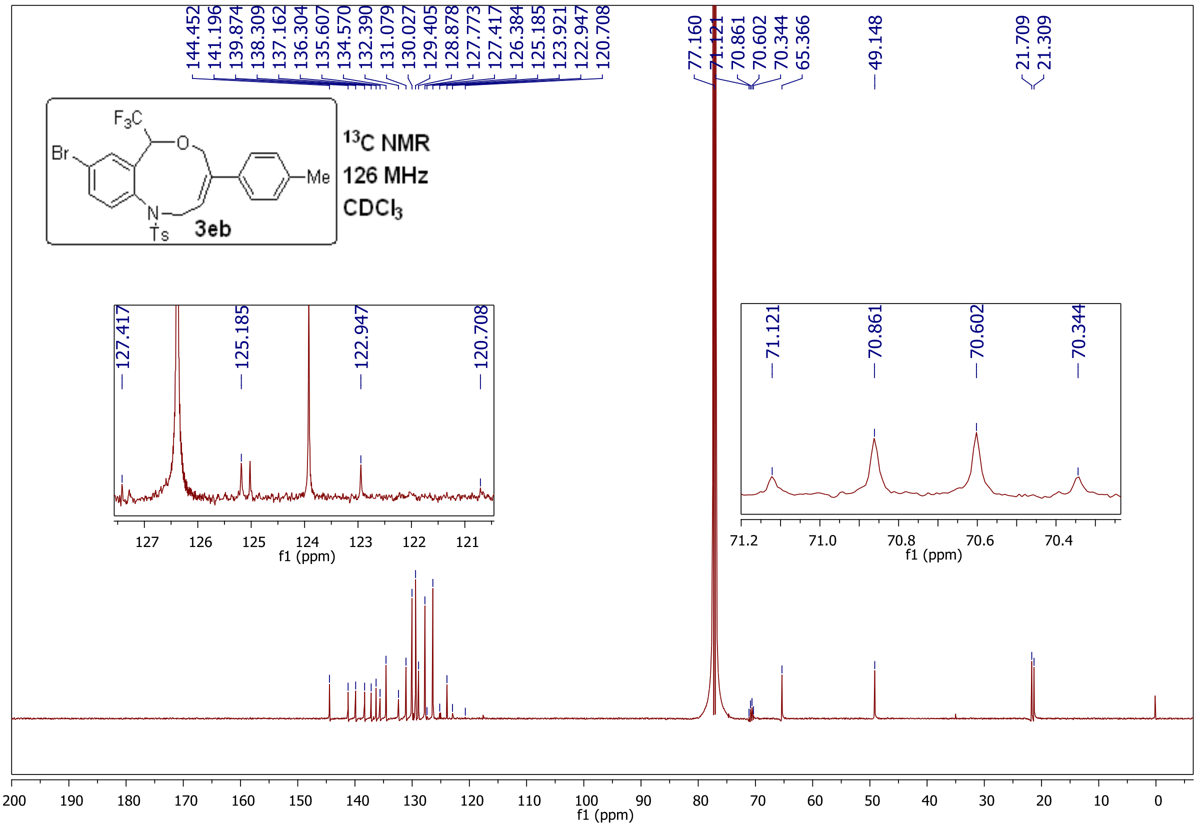
144.452
141.196
139.874
138.309
137.162
136.304
135.607
134.570
132.390
131.079
130.027
129.405
128.878
127.773
127.417
126.384
125.185
123.921
122.947
120.708
77.160
71.121
70.861
70.602
70.344
65.366
49.148
21.709
21.309
F3C
O
Br
Me
N
Ts
3eb
13C NMR
126 MHz
CDCl3
f1 (ppm)

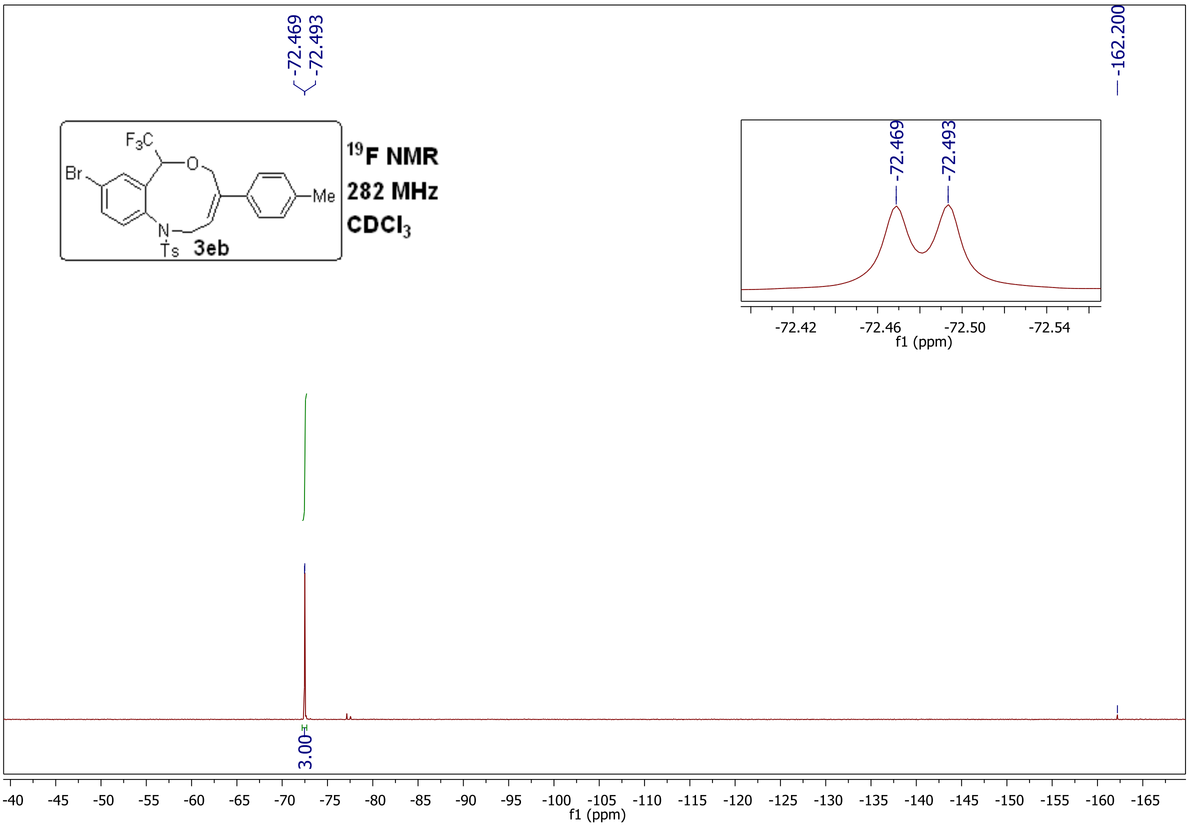
-72.469
-72.493
-162.200
F3C
O
Br
Me
N
Ts
3eb
19F NMR
282 MHz
CDCl3
3.00
f1 (ppm)

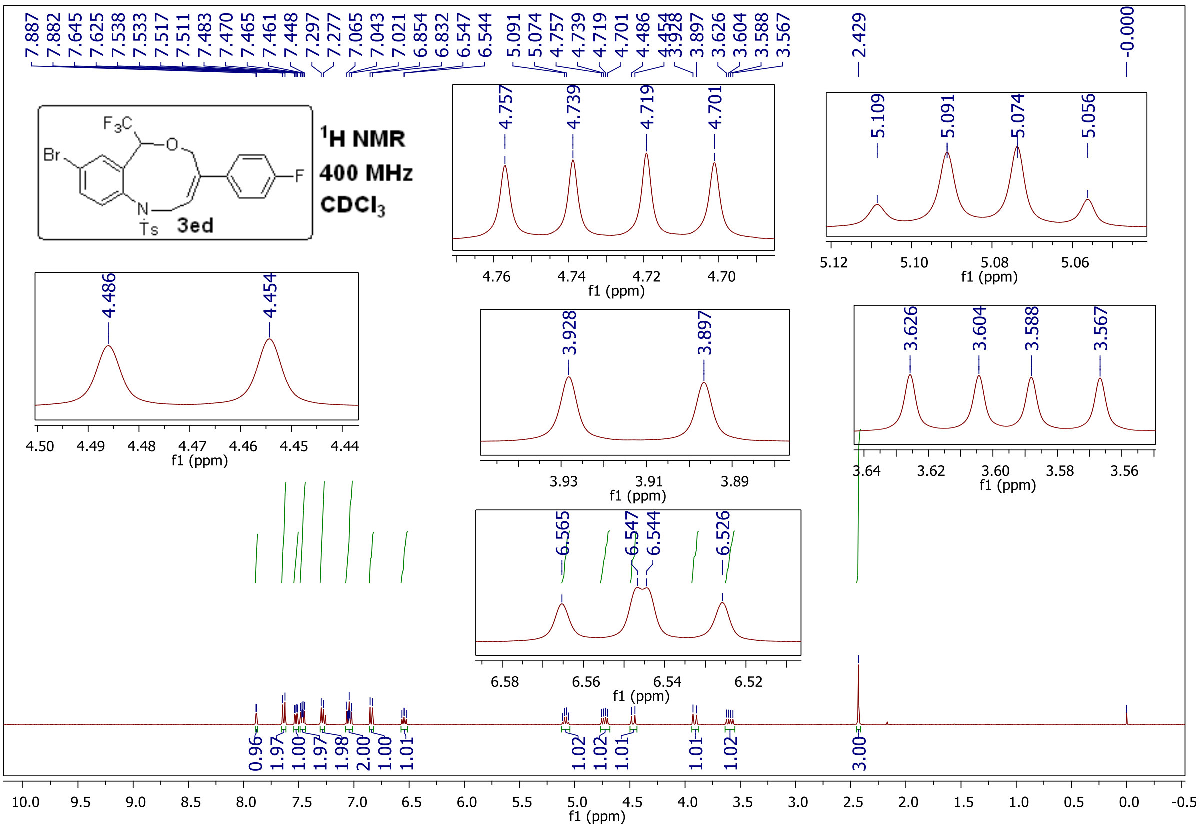

1H NMR
400 MHz
CDCl3
3ed
f1 (ppm)

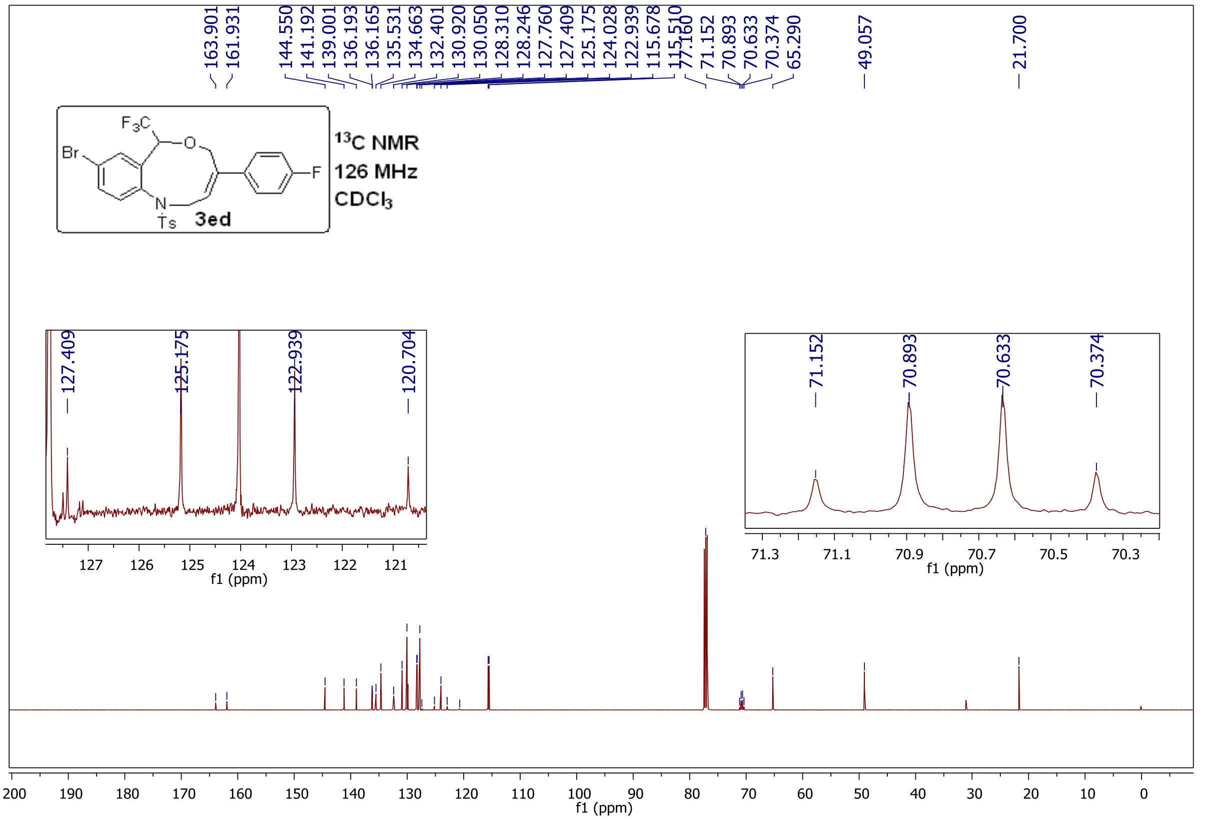

13C NMR
126 MHz
CDCl3
3ed
f1 (ppm)

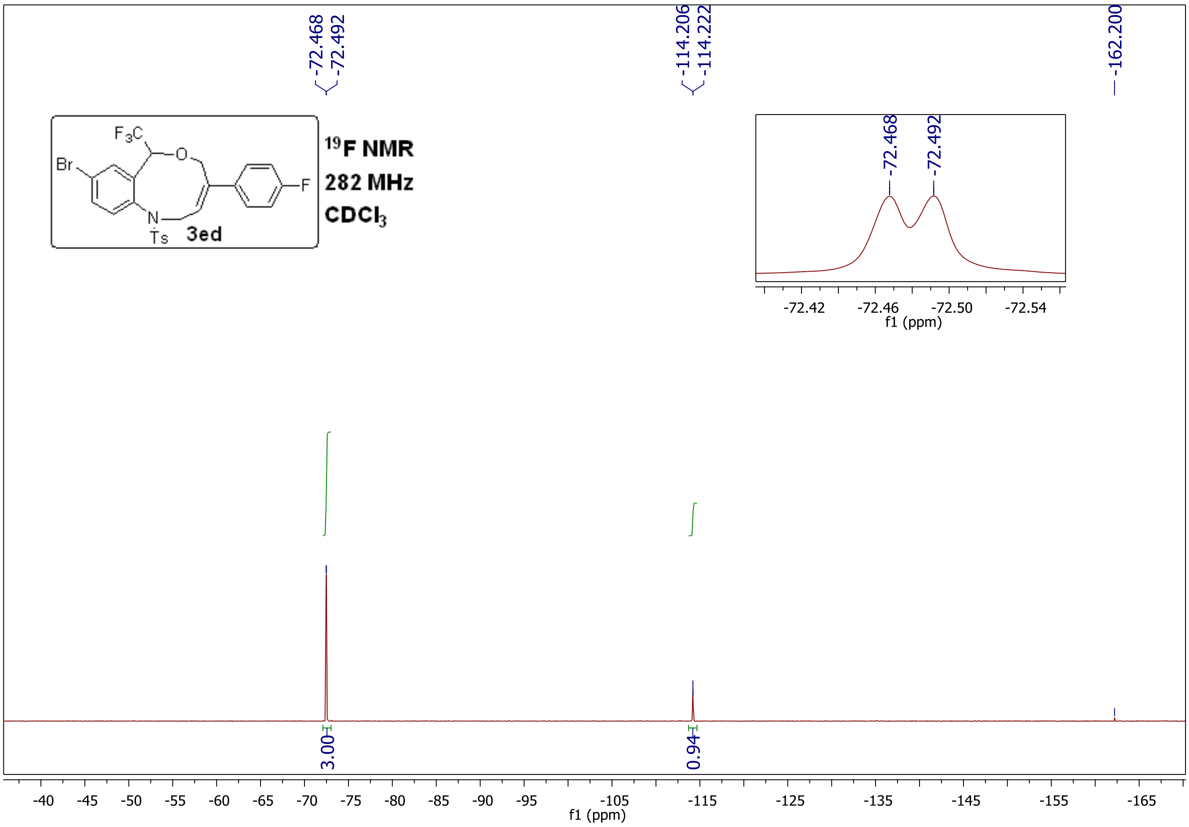

-72.468
-72.492
-114.206
-114.222
-162.200
3ed
19F NMR
282 MHz
CDCl3
3.00
0.94
f1 (ppm)

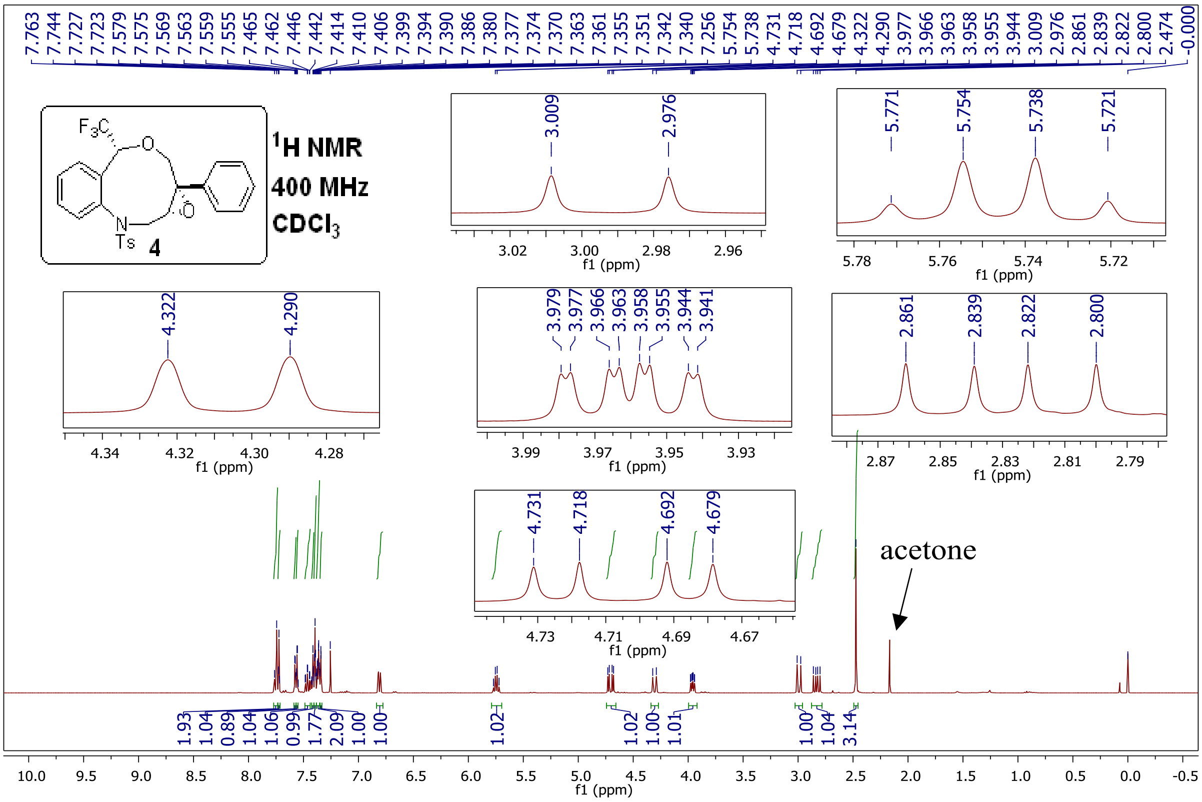

4
1H NMR
400 MHz
CDCl3
acetone
f1 (ppm)

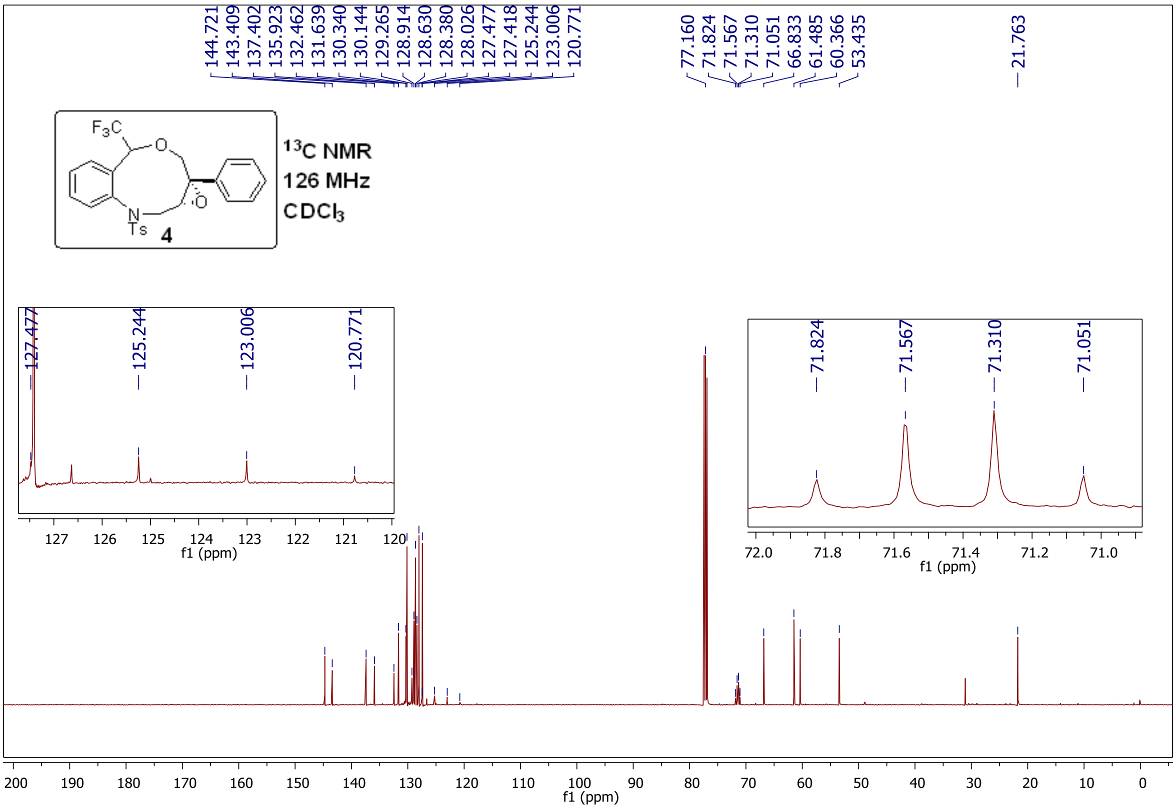
144.721
143.409
137.402
135.923
132.462
131.639
130.340
130.144
129.265
128.914
128.630
128.380
128.026
127.477
127.418
125.244
123.006
120.771
77.160
71.824
71.567
71.310
71.051
66.833
61.485
60.366
53.435
21.763
F_3C
O
N
Ts
4
O
^{13}C NMR
126 MHz
$CDCl_3$
f1 (ppm)

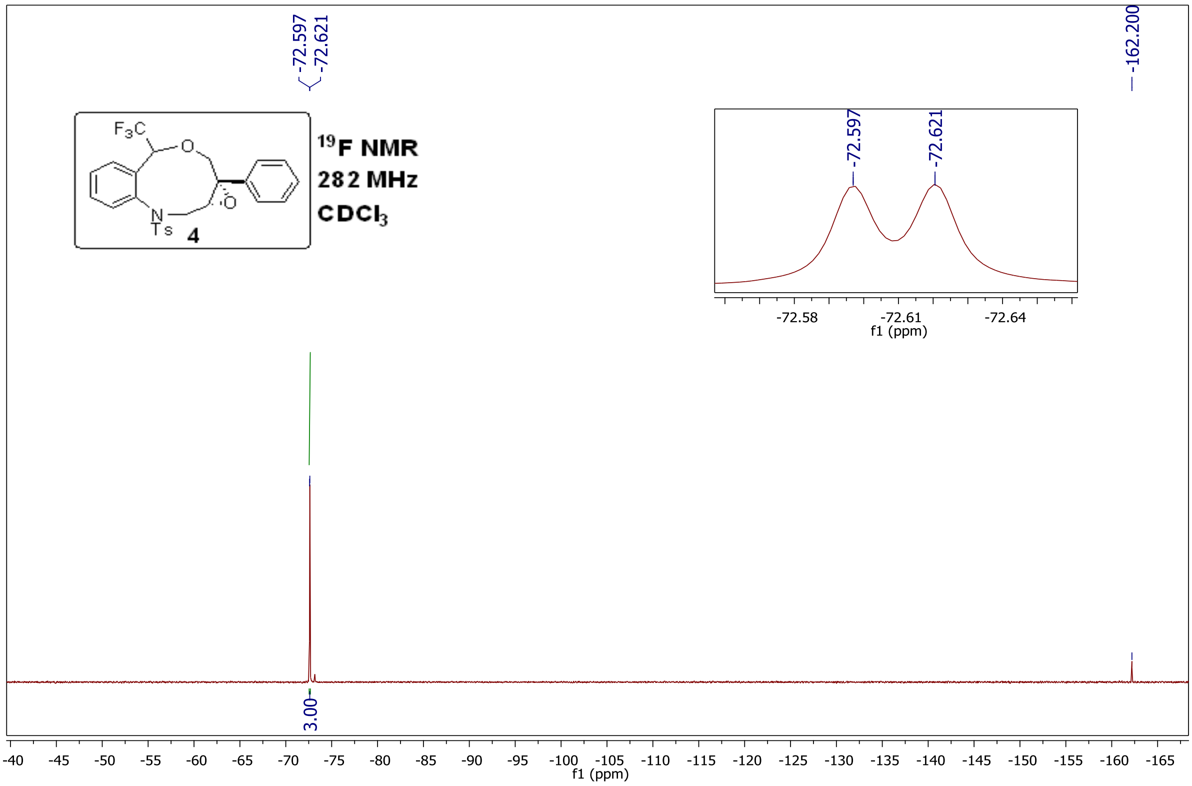
-72.597
-72.621
-162.200
F_3C
O
N
Ts
4
O
^{19}F NMR
282 MHz
$CDCl_3$
3.00
f1 (ppm)

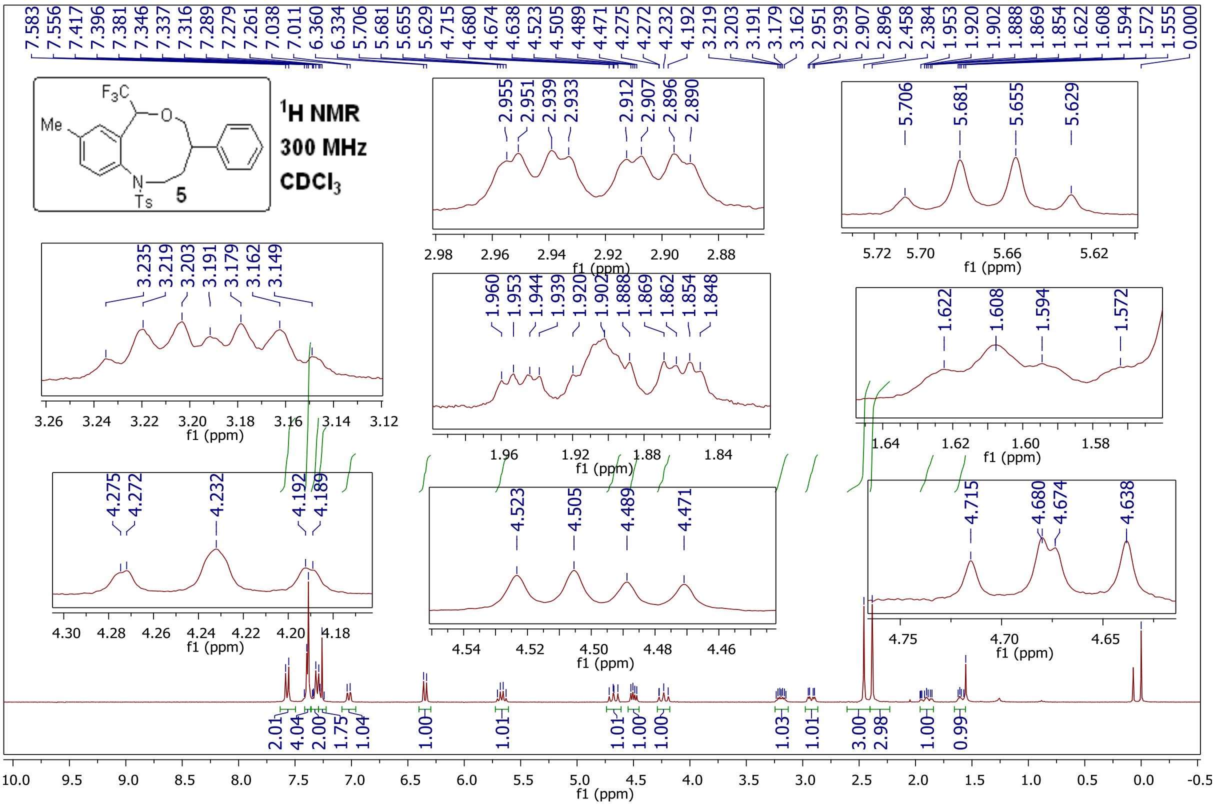
7.583 7.556 7.417 7.396 7.381 7.346 7.337 7.316 7.289 7.279 7.261 7.038 7.011 6.360 6.334 5.706 5.681 5.655 5.629 4.715 4.680 4.674 4.638 4.523 4.505 4.489 4.471 4.275 4.272 4.232 4.192 3.219 3.203 3.191 3.179 3.162 2.951 2.939 2.907 2.896 2.458 2.384 1.953 1.920 1.902 1.888 1.869 1.854 1.622 1.608 1.594 1.572 1.555 0.000
F_3C
Me
O
N
Ts
5
^{1}H NMR
300 MHz
$CDCl_3$
2.955 2.951 2.939 2.933 2.912 2.907 2.896 2.890
5.706 5.681 5.655 5.629
3.235 3.219 3.203 3.191 3.179 3.162 3.149
1.960 1.953 1.944 1.939 1.920 1.902 1.888 1.869 1.862 1.854 1.848
1.622 1.608 1.594 1.572
4.275 4.272 4.232 4.192 4.189
4.523 4.505 4.489 4.471
4.715 4.680 4.674 4.638
2.01 4.04 2.00 1.75 1.04 1.00 1.01 1.01 1.00 1.00 1.03 1.01 3.00 2.98 1.00 0.99
f1 (ppm)

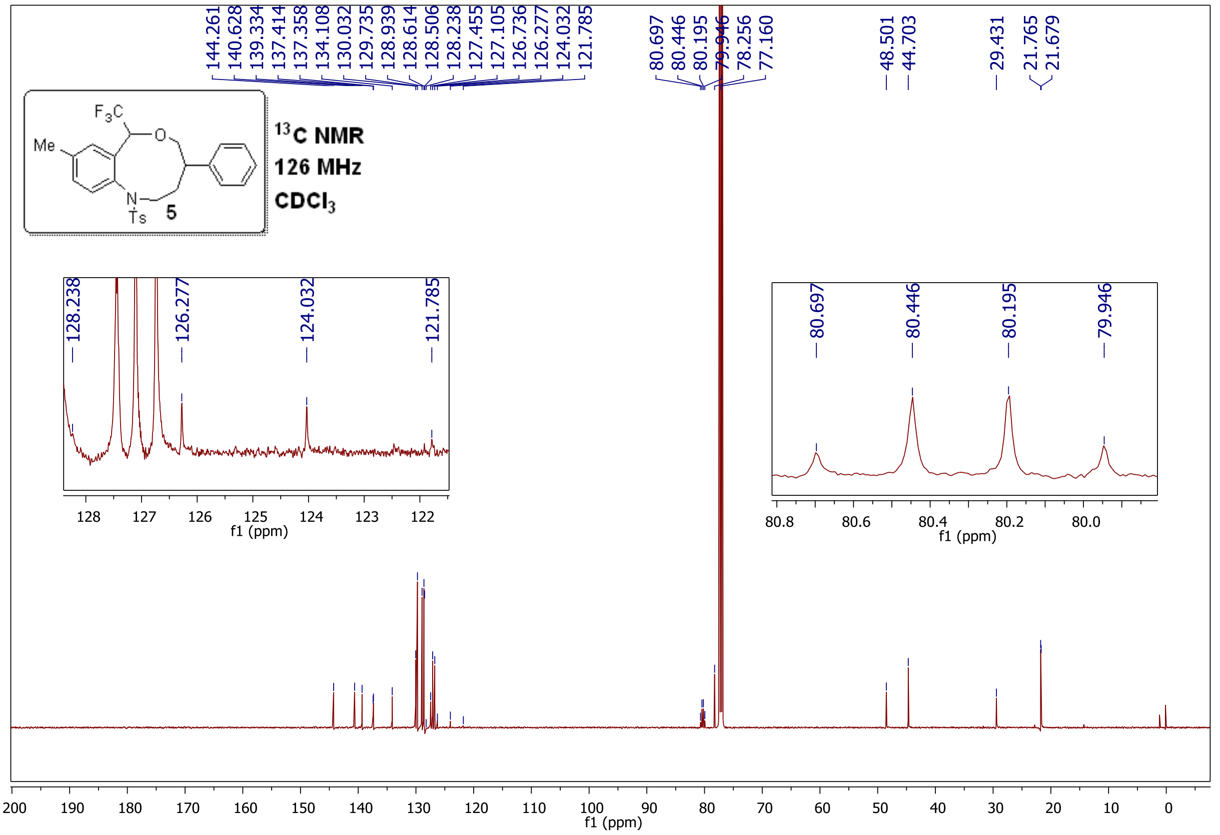
144.261 140.628 139.334 137.414 137.358 134.108 130.032 129.735 128.939 128.614 128.506 128.238 127.455 127.105 126.736 126.277 124.032 121.785
80.697 80.446 80.195 79.946 78.256 77.160
48.501 44.703
29.431 21.765 21.679
F_3C
Me
O
N
Ts
5
^{13}C NMR
126 MHz
$CDCl_3$
128.238 126.277 124.032 121.785
80.697 80.446 80.195 79.946
f1 (ppm)

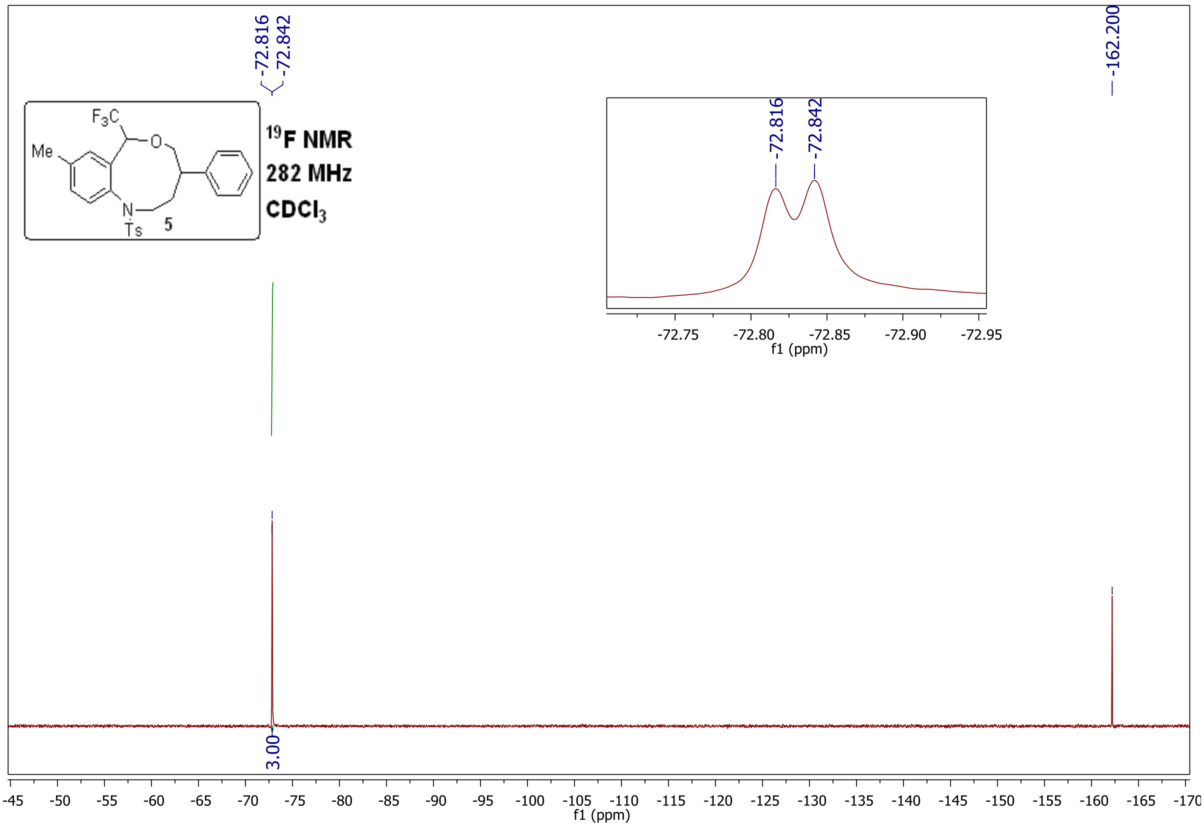

-72.816
-72.842
-162.200
^{19}F NMR
282 MHz
CDCl$_3$
5
3.00
f1 (ppm)

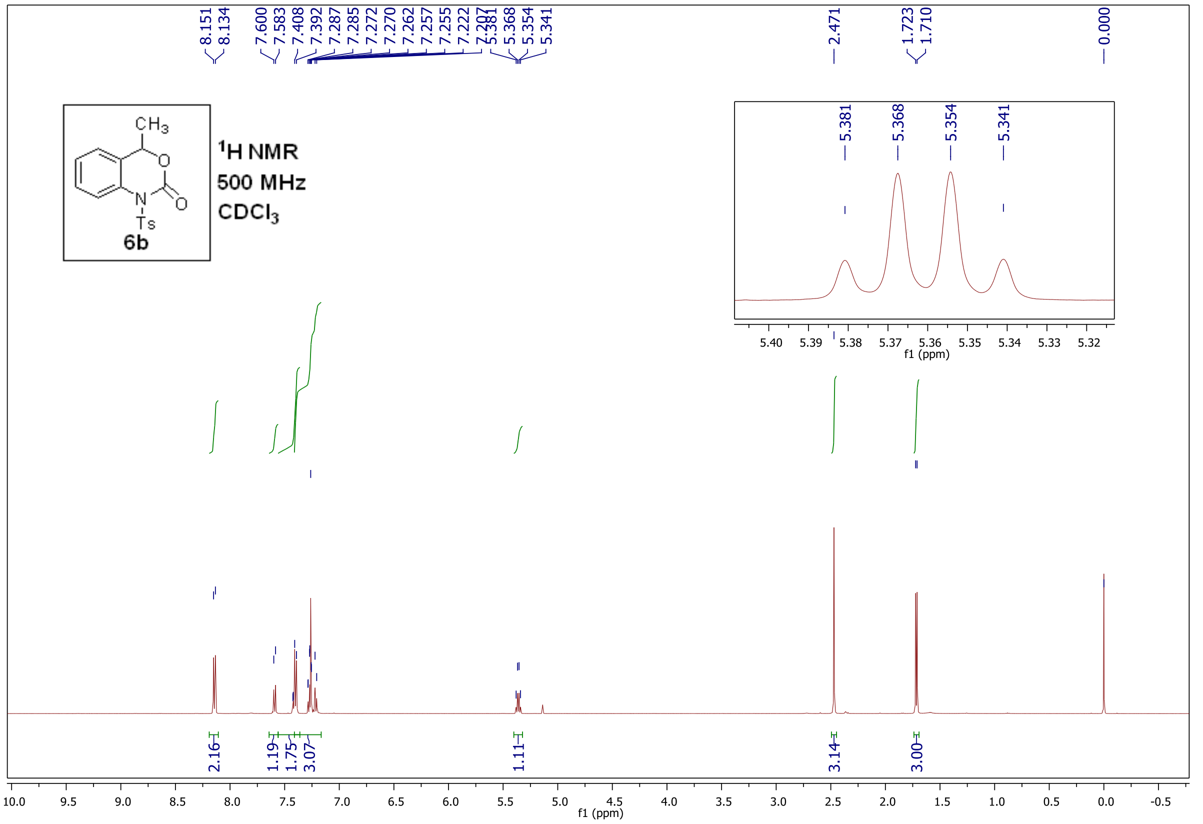

^{1}H NMR
500 MHz
CDCl$_3$
6b
8.151
8.134
7.600
7.583
7.408
7.392
7.287
7.285
7.272
7.270
7.262
7.257
7.255
7.222
5.381
5.368
5.354
5.341
2.471
1.723
1.710
0.000
2.16
1.19
1.75
3.07
1.11
3.14
3.00
f1 (ppm)

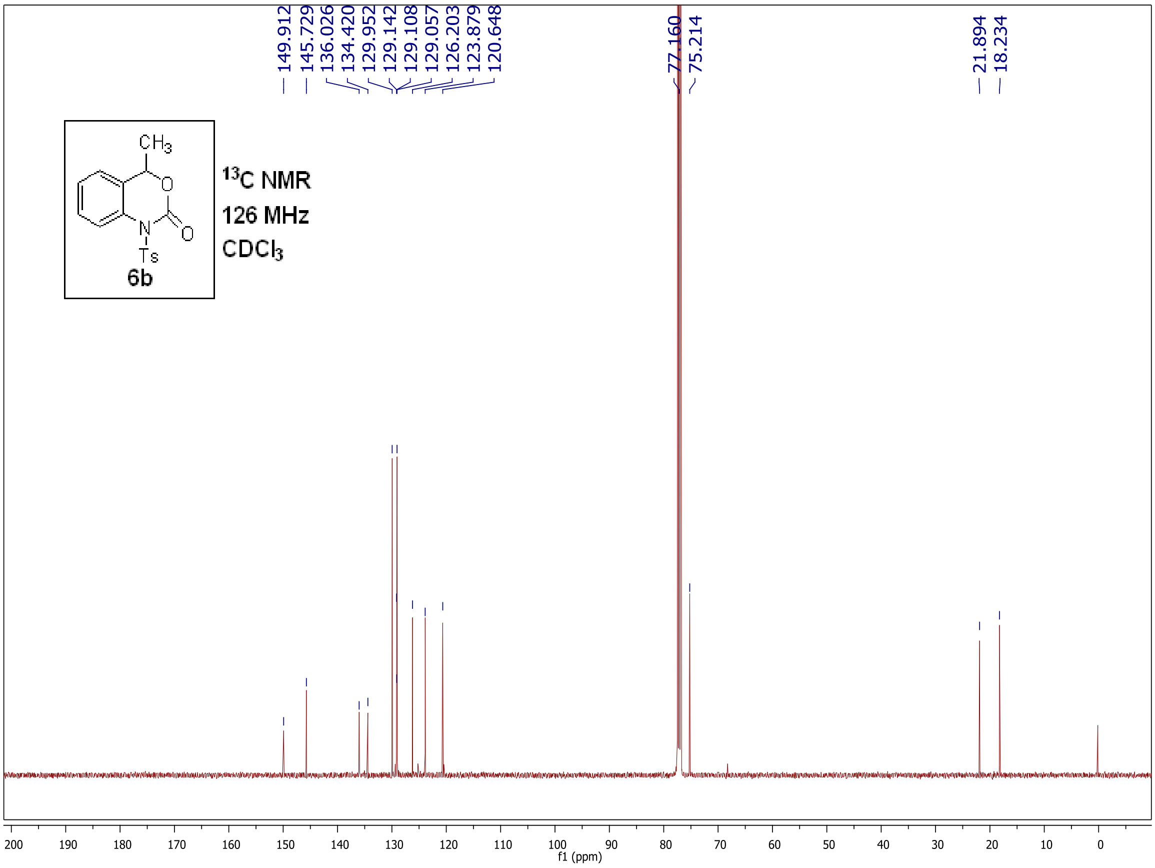
149.912
145.729
136.026
134.420
129.952
129.142
129.108
129.057
126.203
123.879
120.648
77.160
75.214
21.894
18.234
CH3
O
N
O
Ts
6b
13C NMR
126 MHz
CDCl3
f1 (ppm)

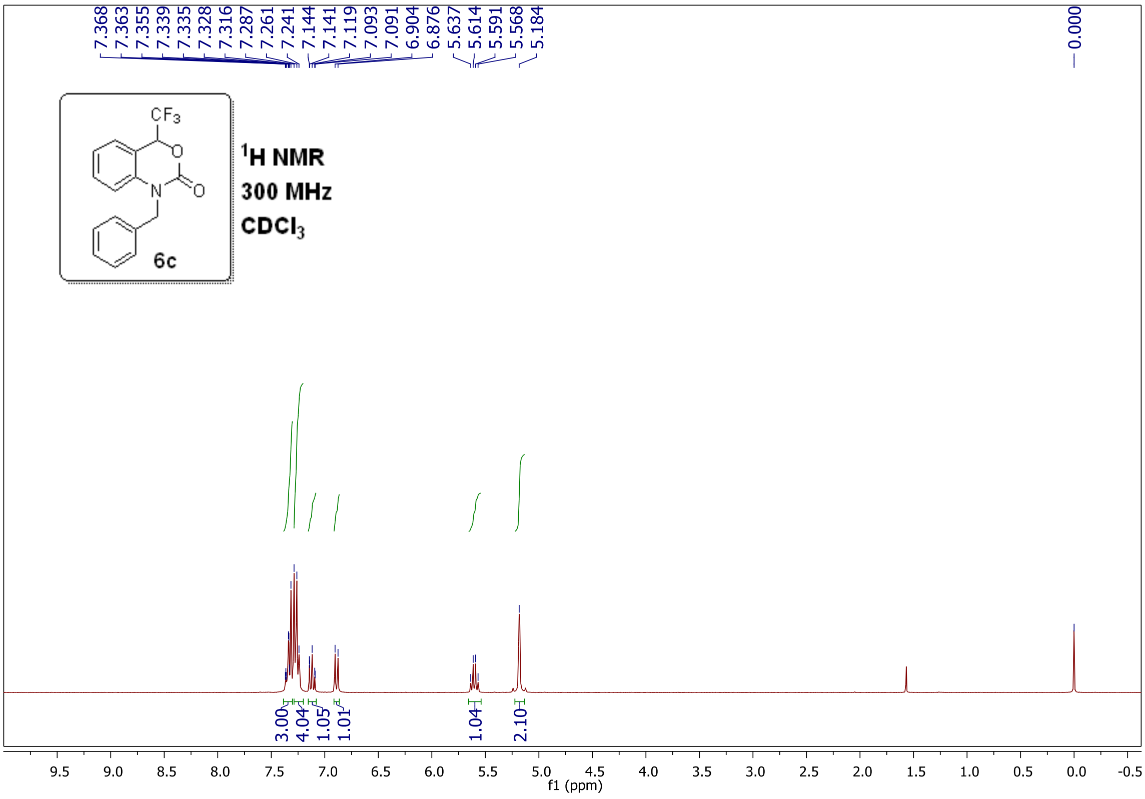
7.368
7.363
7.355
7.339
7.335
7.328
7.316
7.287
7.261
7.241
7.144
7.141
7.119
7.093
7.091
6.904
6.876
5.637
5.614
5.591
5.568
5.184
0.000
CF3
O
N
O
6c
1H NMR
300 MHz
CDCl3
3.00
4.04
1.05
1.01
1.04
2.10
f1 (ppm)

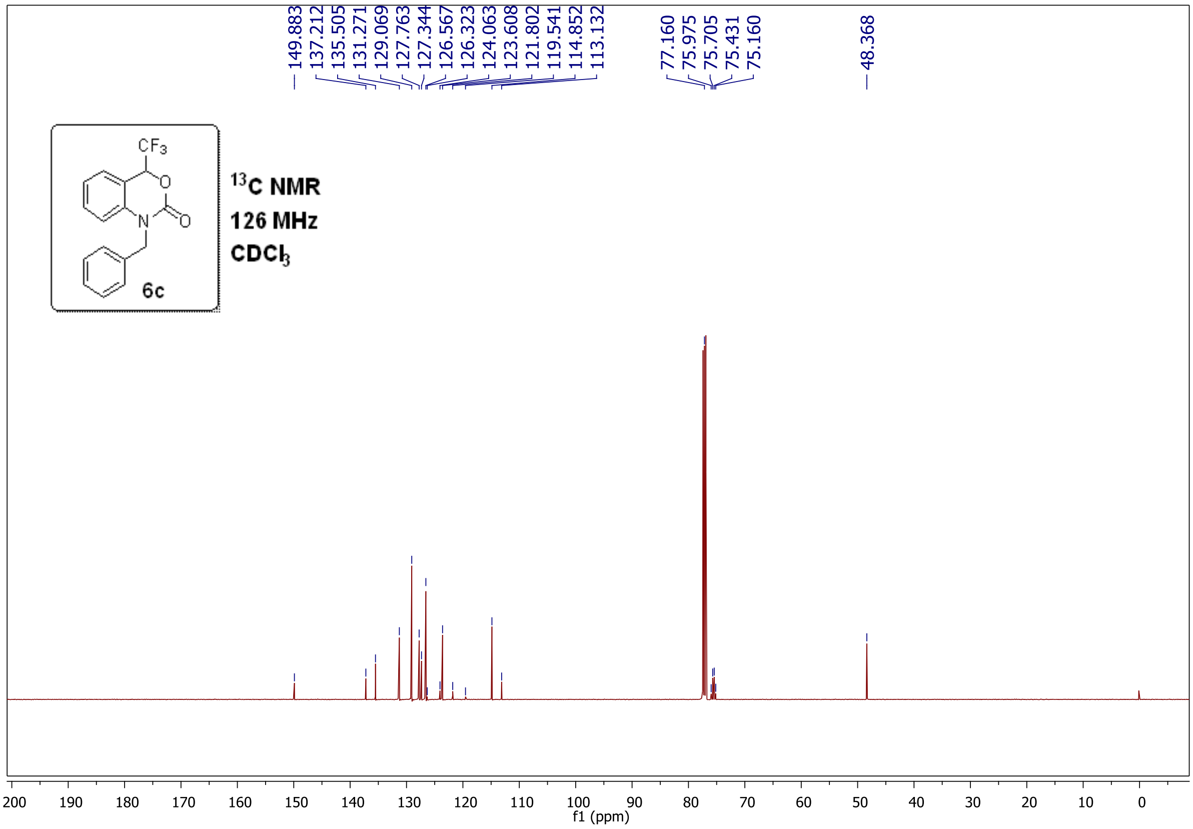
149.883
137.212
135.505
131.271
129.069
127.763
127.344
126.567
126.323
124.063
123.608
121.802
119.541
114.852
113.132
77.160
75.975
75.705
75.431
75.160
48.368
6c
13C NMR
126 MHz
CDCl3
f1 (ppm)

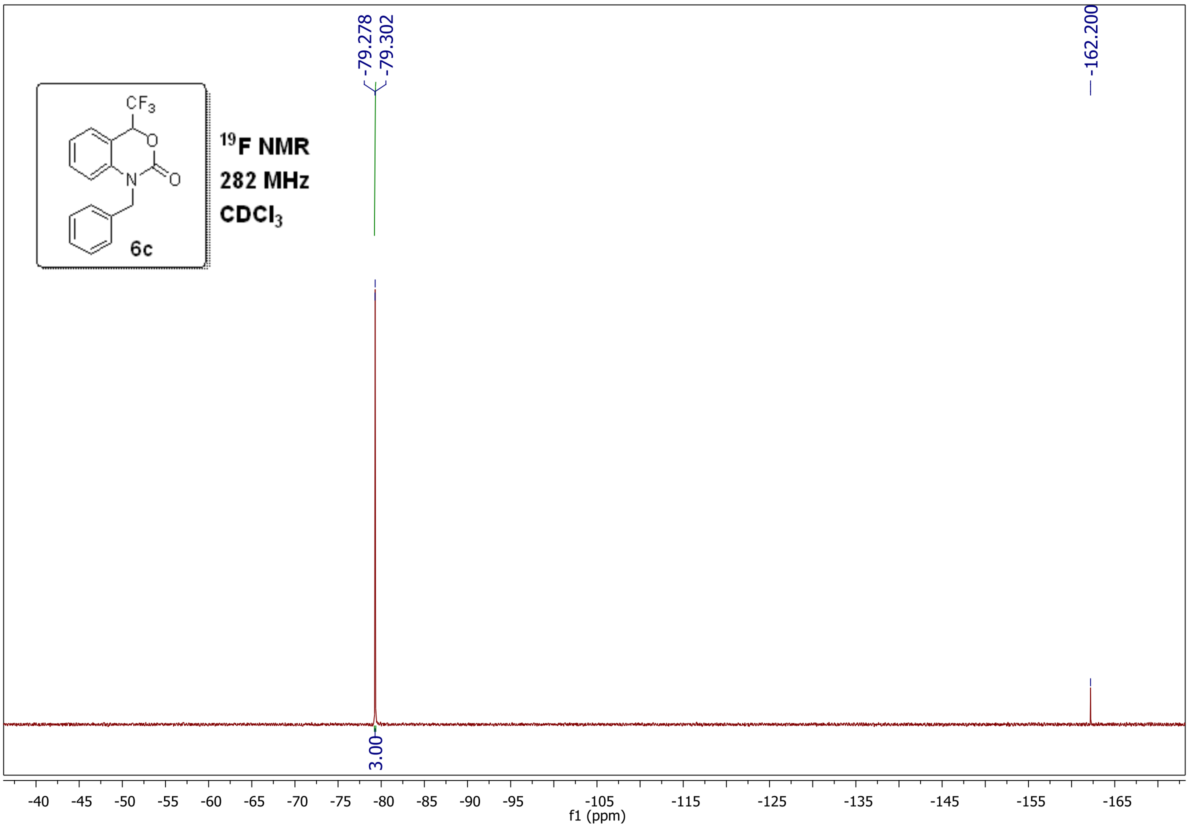
-79.278
-79.302
-162.200
6c
19F NMR
282 MHz
CDCl3
3.00
f1 (ppm)
